# Benzothiazole–Triazole–Indazole Conjugates: Design, Characterization, and Preliminary *In Vitro* MTT-Based Antiproliferative Evaluation

**DOI:** 10.3390/molecules31193452

**Published:** 2026-09-28

**Authors:** Fatima E. Laghchioua, Maurizio Viale, Renata Silva, Fernando Remião, José A. S. Cavaleiro, M. Amparo F. Faustino, El Mostapha Rakib, Maria Graça P. M. S. Neves, Nuno M. M. Moura

**Affiliations:** 1 Laboratory of Molecular Chemistry, Materials and Catalysis, Faculty of Sciences and Technics, Sultan Moulay Slimane University, Beni-Mellal BP 523, Morocco; laghchiouaa@gmail.com; 2 U.O. Bioterapie, IRCCS Azienda Ospedaliera Metropolitana (IRCCS AOM), L.go R. Benzi, 10, 16132 Genova, Italy; maurizio.viale@aomliguria.it; 3 UCIBIO-Applied Molecular Biosciences Unit, Laboratory of Toxicology, Department of Biological Sciences, Faculty of Pharmacy, University of Porto, Rua Jorge Viterbo Ferreira, 228, 4050-313 Porto, Portugal; rsilva@ff.up.pt (R.S.); remiao@ff.up.pt (F.R.); 4 LAQV-REQUIMTE, Department of Chemistry, University of Aveiro, 3810-193 Aveiro, Portugal; jcavaleiro@ua.pt (J.A.S.C.); faustino@ua.pt (M.A.F.F.); gneves@ua.pt (M.G.P.M.S.N.); 5 Higher School of Technology, Sultan Moulay Slimane University, Fquih Ben Salah BP 336, Morocco

**Keywords:** benzothiazole, indazole, 1,2,3-triazole, CuAAC, antiproliferative activity, SwissADME

## Abstract

A new library of benzothiazole–triazole–indazole conjugates (**5a**–**s**) was efficiently synthesized via a Cu(I)-catalyzed azide–alkyne cycloaddition (CuAAC) strategy. The approach involved the preparation of alkynylated indazoles and benzothiazole–azide derivatives, followed by click-chemistry cycloaddition to provide triazole-linked heterocyclic hybrids in good-to-excellent yields (72–95%), demonstrating the robustness of the modular synthetic design. *In silico* evaluation using SwissADME indicated that several compounds display physicochemical profiles potentially compatible with early-stage hit exploration, although the relatively high TPSA values suggest that membrane permeability may be limited for some derivatives. The antiproliferative activity of conjugates **5a**–**s** was assessed against A2780 (ovarian), A549 (lung), and MDA-MB-231 (breast) cancer cell lines using the MTT assay. Several conjugates exhibited greater *in vitro* antiproliferative activity toward A2780 cells, with **5r** emerging as the most active derivative of the series (IC_50_ = 15.7 ± 1.6 μM). In addition, **5b** showed notable antiproliferative activity against the MDA-MB-231 cell line (IC_50_ = 10.8 ± 2.9 μM), highlighting cell-line-dependent antiproliferative profiles. Preliminary structure–activity relationship analysis suggested that electronic effects alone do not fully account for the observed antiproliferative activity, and that substituent position, steric factors, and cell-line-dependent factors may also play important roles in modulating biological response. Given the micromolar activity, the MTT-based nature of the assay, and the absence of non-tumoral cell line data, these compounds are best regarded as chemical starting points for further optimization and mechanistic investigation.

## 1. Introduction

The dynamic nature of the pharmaceutical industry has stimulated the emergence of numerous innovative technologies and approaches for the development of new chemical entities. In the last twenty years, special attention has been given to pericyclic reactions, particularly cycloadditions, which have become increasingly significant and noteworthy among the scientific community [1,2,3]. 1,3-Dipolar cycloaddition reactions, commonly referred to as Huisgen cycloadditions, are particularly notable in both chemical and biological sectors, as they simplify and improve synthetic processes [4,5,6]. This approach is a cornerstone in the concept introduced by Sharpless in 2001, concerning click chemistry [7], which emphasizes high-yielding, selective, and modular reactions for the rapid synthesis of diverse molecular architectures [5,8,9,10].

The discovery that, in the presence of copper(I), the reaction involving organic azides and terminal alkynes exclusively affords 1,4-regioisomers of 1,2,3-triazoles in high yields had a significant impact on the field of click chemistry [11,12,13]. This variant of the Huisgen reaction, referred to as the copper-catalyzed azide–alkyne cycloaddition (CuAAC), is well-established through its utilization across diverse realms including drug discovery, bioconjugation, polymer and materials science, and related areas such as supramolecular chemistry [14,15,16]. Extensive research has been carried out on triazoles and their derivatives, confirming the pharmacological importance of this heterocyclic nucleus [17,18]. Although not naturally occurring, synthetic molecules containing 1,2,3-triazole moieties exhibit diverse biological activities and serve as attractive linkers due to their metabolic stability and ability to form hydrogen bonds, factors that can positively influence binding to biomolecular targets and enhance solubility [8,19,20,21,22,23,24]. Thus, in medicinal chemistry, the 1,2,3-triazole ring is often used not only as a synthetic connection point, but also as a stable linker capable of modulating molecular geometry, polarity, and potential interactions with biological targets.

Benzothiazole and indazole are both privileged heterocyclic scaffolds in medicinal chemistry. Benzothiazole derivatives have been widely investigated as bioactive molecules, including antiproliferative agents, because this ring system can participate in hydrophobic, π–π, and hydrogen-bonding interactions with biological targets. Indazole derivatives are also important in anticancer-oriented drug discovery, particularly as kinase inhibitors and other targeted agents. Molecular hybridization of benzothiazole and indazole fragments through a 1,2,3-triazole linker may therefore provide structurally diverse conjugates in which the triazole unit acts both as a stable linker and as a potential interaction motif. This type of hybridization strategy has been increasingly used in the design of heterocyclic compounds with antiproliferative activity, since the biological response of such systems can be strongly influenced by the nature of the fused heterocyclic cores, linker identity, substituent position, and physicochemical properties [25,26,27]. This design was intended to generate a first-generation library suitable for preliminary structure–activity relationship analysis rather than fully optimized drug candidates.

Recent studies on related heterocyclic systems have further shown that benzothiazole-, indazole-, and triazole-containing compounds may display relevant antiproliferative profiles, although their potency and selectivity are highly dependent on the cell model, substitution pattern, and additional pharmacophoric elements [28,29,30]. In comparison with these previous systems, the present work explores a new benzothiazole–triazole–indazole framework prepared through a modular CuAAC strategy, allowing variation in both the indazole and benzothiazole fragments. This provides a useful platform for preliminary evaluation of how substituent position and scaffold combination influence cell-line-dependent antiproliferative activity.

The versatility of benzothiazoles [31,32,33,34,35,36,37,38,39,40,41,42,43,44,45] and indazoles [46,47,48,49,50,51,52,53,54,55,56,57,58,59], not only in terms of their chemical reactivity but also in terms of their wide-ranging applications across different fields, motivated us to propose a straightforward strategy to conjugate both nuclei using a CuAAC approach. The drug-likeness of the compounds was evaluated *in silico* to identify derivatives with physicochemical profiles that may be compatible with further optimization. In addition, considering our interest in developing new *N*-heterocycles with antiproliferative potential [27,60], the activity of the new conjugates was assessed against three human cancer cell lines: A2780 (ovarian carcinoma), A549 (lung adenocarcinoma), and MDA-MB-231 (triple-negative breast adenocarcinoma). These cell lines were selected as representative models of ovarian, lung, and triple-negative breast cancers, given their clinical relevance and frequent use in preliminary *in vitro* anticancer screening. Since no non-tumoral cell line or mechanistic assay was included at this stage, the biological data are interpreted as preliminary MTT-based antiproliferative activity and cell-line-dependent response, rather than as evidence of tumor selectivity, cytotoxicity, safety, or a defined mechanism of action.

## 2. Results and Discussion

### 2.1. Synthesis and Structural Characterization

The conjugation of indazoles to benzothiazoles via the CuAAC approach required prior functionalization of the indazole scaffolds with an alkynyl unit to afford intermediates **2a**–**d** (Scheme 1), as well as functionalization of the benzothiazole derivatives with an azide moiety to provide intermediates **4a**–**g** (Scheme 2). The subsequent CuAAC reaction between these components afforded the 19 new target benzothiazole-triazole-indazole conjugates **5a**–**s** (Scheme 3). The indazole scaffolds **2a**–**d**, bearing the alkynyl unit, were obtained by reacting the adequate indazoles **1a**–**d** with propargyl bromide in the presence of K_2_CO_3_ in DMF (Scheme 1). These alkylations were performed under reflux for the nitroindazoles **1a**–**c** and at room temperature for **1d**, affording the desired derivatives in yields ranging from 74% to 90% after 1.5 h and 1 h, respectively. The initial indazoles **1a**–**d** were obtained according to literature procedures [27,61,62,63,64]. The nitroindazoles **1a**–**c** were obtained following the protocol reported by Noelting, involving diazotization of the adequate 2-methyl-nitroanilines [62,63], while 5-methoxy-1*H*-indazole-3-carbaldehyde **1d** was obtained by reacting 5-methoxyindole in DMF with aqueous NaNO_2_ and hydrochloric acid [64].

The structures of indazole derivatives **1d** and **2a**–**d** were confirmed by ^1^H and ^13^C NMR spectroscopy and when available are consistent with those previously reported in the literature (see Experimental Section and Figures S1–S10). The resonances at δ 10.17 ppm (^1^H NMR) and at 187.9 ppm (^13^C NMR) corroborate the successful formylation of 5-methoxyindole. The ^1^H NMR of compounds **2a**–**d** confirmed the presence of the extra propynyl unit; the most relevant features are the presence of a doublet at *ca.* 5.5 ppm due to the resonance of methylene protons and of a triplet at *ca.* 3.5 ppm due to terminal alkynyl hydrogen. In the ^13^C NMR spectra, the acetylenic carbons were observed at *ca* 76.8–78.5 ppm, while the *N*–CH_2_ carbon was not clearly observed, likely due to overlap with the DMSO-*d*_6_ solvent signal.

The second component, the benzothiazole derivatives **4a**–**g** bearing the azide function were obtained by reacting the *N*-(benzo[*d*]thiazol-2-yl)-2-chloroacetamide **3a**–**g** with sodium azide in DMF at room temperature (Scheme 2). After column purification, the new benzothiazole–azide derivatives **4a**–**g** were isolated in yields ranging from 75% to 95%. Precursors **3a**–**g** were obtained from the reaction of the adequate aminobenzothiazole with chloroacetyl chloride in the presence of triethylamine (TEA) in tetrahydrofuran (THF) at 0 °C, affording the required benzothiazole–acetamide derivatives in excellent yields ranging from 83% to 91% [65,66]. The aminobenzothiazole scaffolds were obtained in excellent yields (>80%) by reacting the corresponding anilines with potassium thiocyanate in the presence of bromine, following the literature procedures with minor modifications [65,67,68].

The ^1^H NMR spectra of the new derivatives **4a**–**g** are consistent with the proposed structures. The most relevant features include the presence of a singlet in the range δ 4.2–4.3 ppm due to the resonance of the methylene protons and a broad peak between ca. δ 12.4 and δ 12.7 ppm due to the resonance of the *N*H proton. The peaks between *ca* δ 8.3 and δ 7.0 ppm were assigned to the resonance of aromatic protons of benzothiazole core. The ^13^C NMR spectra of **4a**–**g** showed a signal in the range of δ 51.1 to δ 51.4 ppm due to the resonance of the methylene carbon from the azidoacetamide moiety, while the resonances of benzothiazole carbons appear between δ 121.1 and δ 148.9 ppm. The mass spectra of these scaffolds showed the expected *m*/*z* value corresponding to the [M + H]^+^ ion (Figures S11–S30 in the Supplementary Materials).

The CuAAC cycloaddition between the terminal alkynes **2a**–**d** and the azido derivatives **4a**–**g** in the presence of copper(I) afforded the 19 new conjugates **5a**–**s** (Scheme 3)**.** These CuAAC reactions took place in the presence of sodium ascorbate and 12 mol% of CuSO_4_, in DMF. All the reactions were carried out at room temperature for 4 h, until the full consumption of the starting materials was confirmed by TLC. After work-up, the target benzothiazole–triazole–indazole derivatives **5a**–**s** were isolated in yields ranging from 72% to 95% after recrystallization from DMSO/ethanol (see Experimental Section).

The structures of the final conjugates **5** were assigned using ^1^H NMR and, where obtainable, ^13^C NMR, COSY experiments, ESI-MS, and HRMS for most derivatives (see Experimental Section and Figures S31–S105 in Supplementary Materials). The ESI-MS spectra showed molecular-ion peaks consistent with the proposed molecular formulas for all final conjugates. HRMS analyses were successfully obtained for 15 of the 19 conjugates and agreed with the calculated exact masses. For **5a**, **5h**, **5l** and **5o** satisfactory HRMS data could not be obtained, most probably because of poor ionization response and/or decomposition under the ionization conditions. For compounds **5b** and **5c** satisfactory ^13^C NMR spectra could not be acquired despite repeated attempts, mainly due to their low solubility in the available deuterated solvents.

The apparent homogeneity of the target conjugates was assessed by TLC, which showed a single major spot and was further supported by the internal consistency of the ^1^H NMR data and, where obtainable, the ^13^C NMR data provided in the supporting information. No major unassigned resonances were observed in the ^1^H NMR spectra, except for residual solvent/water signals or explicitly noted residual ethanol in compound **5j**. Although these observations support sample homogeneity, they do not constitute a quantitative purity determination. HPLC analyses could not be performed owing to limited sample availability and poor solubility of some conjugates.

The formation of the CuAAC products was supported by the disappearance of the terminal alkyne proton signal observed in the indazole precursors and by the appearance of a singlet at *ca*. δ 8.6 ppm in the ^1^H NMR spectra, assigned to the triazole proton. The spectra also showed two characteristic singlets corresponding to the methylene groups connecting the triazole ring to the indazole and acetamide fragments at *ca*. δ 5.5 and δ 5.9 ppm. No additional triazole proton signals attributable to a significant amount of a second regioisomer were observed in the ^1^H NMR spectra. The 1,4-disubstituted 1,2,3-triazole regiochemistry was assigned based on the well-established regioselectivity of Cu(I)-catalyzed azide–alkyne cycloaddition under the applied conditions, together with the diagnostic NMR features and mass spectrometric data. Thus, the combination of the diagnostic ^1^H NMR features, the absence of signals attributable to a second regioisomer, and the ESI-MS and available HRMS data is consistent with the assigned structures of conjugates **5a**–**s**.

### 2.2. In Silico Analysis

The predicted physicochemical properties, lipophilicity, water solubility, drug-likeness, medicinal chemistry parameters and selected ADME-related descriptors of the compounds were obtained using the SwissADME online platform [69,70]. The tool was used to predict the topological polar surface area (TPSA), molecular weight (MW), *n*-octanol/water partition coefficient (XLOGP3), number of rotatable bonds (*n*-rot), number of hydrogen bond acceptors (n-ON), and number of hydrogen bond donors (*n*-OHNH) for the starting precursors **riluzole**, **2b**, **4b** and for all target adducts **5a**–**s**. **Riluzole**, a benzothiazole-containing drug approved for the treatment of amyotrophic lateral sclerosis, was included as a physicochemical reference point given its structural relationship to the benzothiazole core of the synthesized conjugates.

The results of the *in silico* analysis are shown in Table S1 (see Supplementary Materials). The synthesized compounds **5a**–**s** presented molecular weights ranging from 434.43 to 531.47, XLOGP3 values between 2.36 and 3.77, 7–11 hydrogen bond acceptors, and one hydrogen bond donor. The number of rotatable bonds varied between 7 and 10, while TPSA values ranged from 145.06 to 210.40. Three conjugates did not violate any of Lipinski’s rules, eleven violated only one rule, and five violated two rules, accounting for all 19 final compounds. Overall, these results indicate that part of the series falls within, or close to, conventional drug-likeness space, supporting their consideration as preliminary chemical structures for further optimization. Nevertheless, the relatively high TPSA values and a high number of hydrogen-bond acceptors observed for several derivatives, suggest that passive membrane permeability and oral absorption may require structural refinement in future analogs. Therefore, the SwissADME results should be interpreted as preliminary physicochemical guidance for hit selection and optimization, rather than as direct evidence of favorable pharmacokinetic behavior. Importantly, many approved anticancer drugs deviate from strict Lipinski criteria, indicating that these parameters should be considered as useful guiding tools rather than absolute exclusion criteria in early-stage anticancer research.

### 2.3. Biological Studies: Inhibition of Cell Proliferation

The antiproliferative activity of the newly synthesized compounds **5a**–**s** (see Scheme 3) was evaluated against three human cancer cell lines, namely A2780 ovarian carcinoma, A549 lung adenocarcinoma, and MDA-MB-231 triple-negative breast carcinoma, using the MTT assay. The primary objective of the screening was to establish an intra-series ranking of the newly synthesized hybrids and to identify structural features associated with antiproliferative activity across the three tumor cell lines of distinct tissue origin.

The biological evaluation was performed in two steps. In the first step, all nineteen compounds were screened at a single concentration of 30 µM to identify derivatives producing at least 50% inhibition of proliferation relative to vehicle-treated controls (see Experimental Section and Table S2 in Supplementary Materials). Compounds meeting this criterion were selected for second-stage concentration–response evaluation over the concentration range of 0.096–60 µM, and IC_50_ values were determined from the corresponding concentration–response curves by nonlinear regression whenever reliable curve fitting was possible (see Experimental Section and Table S3 in the Supplementary Materials).

Table 1 summarizes the resulting IC_50_ values (half-maximal inhibitory concentrations) for compounds subjected to second-stage evaluation, together with compounds classified as IC_50_ > 30 µM for SAR ranking purposes. Compounds with fitted IC_50_ values above 30 µM, or compounds that failed to reach 50% inhibition at the 30 μM screening concentration, were not considered active under the present screening criteria. Compound **5q** gave a fitted IC_50_ value slightly above this threshold (33.9 ± 1.7 µM) and was therefore considered borderline rather than active. The values correspond to 72 h exposure; a time point commonly used in preliminary MTT-based antiproliferative screening because it allows cell proliferation over several cell divisions.

The results show that these compounds were, in general, more active against A2780 than against the other two cell lines. In A2780 cells, compounds **5a** and **5c**–**e**, as well as **5l**, **5p** and **5r,** showed comparatively better inhibitory activities than the remaining derivatives.

Within the subseries **5a**–**e**, bearing the nitro group at position four of the indazole unit, the best activity against A2780 was found for the derivatives **5e** (IC_50_ = 18.2 ± 6.2 μM), bearing a chlorine substituent at position five of the benzothiazole core (R^1^ = Cl), followed by the unsubstituted analog **5a** (20.9 ± 4.1 μM). Derivatives **5c** (R^1^ = OCF_3_) and **5d** (R^1^ = F) also showed moderate activity, with IC_50_ values of 28.2 ± 0.8 μM and 25.1 ± 3.7 μM, respectively. Interestingly, compound **5b** (R^1^ = OCH_3_) was inactive in A2780 cells under the present screening conditions, but exhibited measurable antiproliferative activity against MDA-MB-231 cells, with an IC_50_ = 10.8 ± 2.9 µM. These results suggest that, within this subset, antiproliferative activity is not directly correlated with the electronic nature (electron-withdrawing or electron-donating) of the benzothiazole substituents. Thus, steric effects, polarity, and/or specific interactions with relevant biological targets may play a more important role than electronic factors alone, although these possibilities remain hypothetical in the absence of mechanistic studies.

For compounds **5f**–**k**, all IC_50_ exceeded 30 μM in all tested cell lines, regardless of the electronic nature of the substituents at the benzothiazole unit. This trend suggests that the presence of a nitro group at position five of the indazole unit is less favorable for antiproliferative activity in this series.

Interestingly, compound **5l** (IC_50_ = 24.1 ± 5.8 μM), bearing a nitro group at position six of the indazole unit, showed activity comparable to the analog **5a** (IC_50_ = 20.9 ± 4.1 μM), indicating that shifting the nitro group from position four to six is well tolerated in the unsubstituted molecule. However, this positional change was not generally favorable in the OCF_3_-substituted molecule, as the corresponding compound **5m** (IC_50_ > 30 μM) displayed reduced activity when compared with **5c** (IC_50_ = 28.2 ± 0.8 μM, borderline value).

For the final subseries **5n**–**s**, bearing methoxy and formyl substituents in the indazole moiety, the most active derivatives against A2780 were **5r** (R^1^ = F), followed by **5p** (R^1^ = OCF_3_) while **5q** (R^1^ = NO_2_) gave a borderline value above the screening cut-off (IC_50_ = 33.9 ± 1.7 μM). Notably, compound **5r** was the most active derivative of the entire library against A2780 cells with an IC_50_ = 15.7 ± 1.6 μM, whereas **5p** showed weak activity (IC_50_ = 29.8 ± 0.4 μM), close to the operational threshold. Compound **5s** (R^1^ = Cl) was inactive (IC_50_ > 30 μM). The antiproliferative activity of compounds **5p** and **5r**, bearing electron-withdrawing groups (OCF_3_ and F), contrasted with the borderline activity of **5q** (NO_2_) and the inactivity of **5s** (Cl), suggests that the activity in this subseries does not appear to be directly correlated with the electron-withdrawing strength of the R^1^ substituent.

For the MDA-MB-231 cell line, only compound **5b** showed measurable antiproliferative activity (IC_50_ = 10.8 ± 2.9 μM) under the present assay conditions, while all the other derivatives had IC_50_ values above 30 μM. Also, all compounds were inactive in A549 cells (IC_50_ > 30 μM), indicating limited activity toward this lung carcinoma line.

Although no reference anticancer drug was tested in parallel under the experimental conditions used, the literature-reported cisplatin IC_50_ values obtained using MTT-based assays in the same cell lines provide just an approximate contextual comparison. Reported values include approximately 2.5 μM in A2780 cells [71], 9 ± 1.6 μM in A549 cells [72], and 63.1 ± 1.2 μM in MDA-MB-231 cells [73], although direct comparison should be made cautiously because assay conditions may differ between studies. In this context, the most active conjugates, **5r** in A2780 (IC_50_ = 15.7 ± 1.6 μM) and **5b** in MDA-MB-231 (IC_50_ = 10.8 ± 2.9 μM), showed measurable to moderate antiproliferative activity and, as unoptimized first-generation hybrid compounds, provide an encouraging starting point for further structural optimization.

The differential sensitivity across A2780, A549, and MDA-MB-231 cells may reflect differences in cell-line-specific biology and/or intracellular compound exposure. These cell lines have distinct genetic profiles, molecular characteristics, and cellular pathway dependencies, which can influence their response to small molecules [74]. In addition, compound effectiveness can depend on the presence and levels of specific cellular targets, as well as on differences in drug resistance and drug disposition mechanisms. In this regard, distinct sensitivities to targeted anticancer agents across cell-line panels have been reported; for example, Ogasawara and colleagues observed variable responses to lenvatinib across human liver cancer cell lines, which correlated with differential expression of growth-related factors (e.g., FGFR1–4, FGF19, FRS2α, and RET) [75].

In our panel, the consistent inactivity in A549 cells (IC_50_ > 30 μM) and the almost complete inactivity in MDA-MB-231 cells (except **5b**) may reflect limited intracellular exposure and/or resistance mechanisms in these models. SwissADME predictions provide a useful qualitative context for this behavior: the conjugates are relatively large and polar molecules (MW 434–531 g/mol; TPSA 145–210 Å^2^) with multiple hydrogen-bond acceptors, features generally associated with reduced passive membrane permeability. Moreover, SwissADME predicts that several derivatives may behave as P-gp substrates (Table S1). Since P-gp is a major efflux transporter implicated in multidrug resistance [67,68,69], transporter-mediated efflux may represent one possible explanation for reduced intracellular accumulation and, consequently, lower apparent potency in efflux-competent cells; however, this hypothesis requires experimental validation. Cell-line-specific differences in uptake mechanisms and metabolic capacity may further influence intracellular compound levels and biological response. More broadly, A549 and MDA-MB-231 are often reported to display comparatively resistant phenotypes in drug screening studies, and their lower responsiveness here may also reflect cell-line-specific genetic background and resistance mechanisms.

Within A2780, the higher activity of **5r** (IC_50_ = 15.7 ± 1.6 μM) may be partly consistent with its comparatively lower polarity within the series (TPSA = 145.06 Å^2^) combined with moderate lipophilicity (XLOGP3 = 2.63), which could favor improved cellular penetration relative to more polar analogs. Conversely, derivatives with higher TPSA values would be expected to incur a greater permeability penalty, which may contribute to weaker antiproliferative effects. However, given the limited number of active compounds and the absence of mechanistic studies, this observation should be regarded as a working hypothesis rather than evidence of a causal relationship. Importantly, the strong dependence of activity on nitro-group position, together with the absence of a clear electronic-substituent trend on the benzothiazole ring, suggests that intrinsic structure–activity requirements (e.g., regioisomer-dependent presentation of functional groups and three-dimensional polarity distribution) may contribute to potency. Finally, the cell-line-dependent activity of **5b** in MDA-MB-231 cells is not readily rationalized by bulk physicochemical descriptors alone, suggesting that cell-line-specific factors and/or target-specific effects may contribute to this behavior. Given the relevance of MDA-MB-231 as a triple-negative breast cancer model, this result is noteworthy and warrants further mechanistic studies.

The activities observed for compounds **5r** (IC_50_ = 15.7 ± 1.6 μM) and **5b** (IC_50_ = 10.8 ± 2.9 μM) are consistent with the moderate antiproliferative activity commonly reported for early-stage heterocyclic hit compounds. Although no reference anticancer drug was evaluated within the present study, these values place the most active compounds of the series in a range that warrants further investigation and optimization.

Because the biological readout is based on the MTT assay, the results describe antiproliferative effects under the assay conditions and do not, by themselves, distinguish cytostatic from cytotoxic outcomes or identify a specific molecular target. In addition, because non-tumoral cell lines were not included in the present study, no conclusions regarding tumor selectivity or safety profile can be drawn. Nonetheless, the combined SAR and *in silico* profiling support future optimization toward reduced polarity and transporter liability while preserving structural features associated with A2780 activity, particularly the favorable nitro-group placement and substituent patterns observed for **5r** and **5e**. Importantly, the screening identified several active members within the synthesized library and revealed preliminary structure–activity relationships that may guide the design of improved analogs. Accordingly, the present findings provide a useful basis for future biological characterization and optimization of this class of benzothiazole–triazole–indazole conjugates.

## 3. Conclusions

This work reports the efficient synthesis of a new series of benzothiazole–triazole–indazole conjugates through a modular CuAAC strategy. In this approach, the indazole derivatives acted as the alkyne component, while the benzothiazole derivatives served as the azide components, affording the target compounds in good to excellent yields (72–95%). *In silico* analysis indicated that part of these derivatives display physicochemical profiles compatible with early-stage drug-likeness criteria, with minimal violations of Lipinski’s rules of five.

Biological evaluation revealed that several compounds exhibit cell-line-dependent antiproliferative activity, particularly against A2780 ovarian carcinoma cells. Benzothiazole–triazole–indazole conjugate **5r** was the most active compound in A2780 cells (IC_50_ = 15.7 ± 1.6 µM) under the MTT assay conditions, while compound **5b** displayed greater activity against the MDA-MB-231 cell line (IC_50_ = 10.8 ± 2.9 µM). Structure–activity relationship analysis indicated that the position of the nitro group on the indazole ring strongly influences antiproliferative activity, while electronic effects of benzothiazole substituents do not fully account for the observed potency.

Overall, these benzothiazole–triazole–indazole hybrids represent preliminary hit-like scaffolds for further anticancer-oriented optimization. Further studies will be required to address selectivity against non-tumoral cell lines, confirm the antiproliferative effects using complementary viability/proliferation assays, and perform mechanistic studies to elucidate the molecular target(s) and mode of action of the most active derivatives. Additional studies on intracellular exposure determinants, including uptake, efflux, and metabolism, would also be important for future optimization.

## 4. Experimental Section

### 4.1. General Remarks

Nuclear magnetic resonance (NMR) spectra were recorded using the following instruments: Bruker AC 300.13 MHz or 500.13 MHz for ^1^H NMR and 75.47 MHz or 125.76 MHz for ^13^C NMR (Bruker, Wissembourg, France); the spectra were recorded in deuterated chloroform (CDCl_3_) or dimethylsulfoxide (DMSO-*d*_6_) and using tetramethylsilane (TMS) as the internal reference, as indicated for each compound. Chemical shifts are given in δ parts per million (ppm) downfield from TMS; the coupling constants (*J*) in Hertz (Hz). Electrospray ionization mass spectra were acquired with a Micromass Q-Tof 2 (Micromass, Manchester, UK), operating in positive ion mode, equipped with a Z-spray source, an electrospray probe and a syringe pump. Source and desolvation temperatures were 80 °C and 150 °C, respectively. Capillary voltage was 3000 V. The spectra were acquired at a nominal resolution of 9000 and at cone voltages of 30 V. Nebulization and collision gases were N_2_ and Ar, respectively. Compound solutions in methanol were introduced at a 10 μL min^−1^ flow rate. High-resolution mass spectra (HRMS) were obtained in a Q-Exactive^®^ hybrid quadrupole Orbitrap^®^ mass spectrometer (Thermo Fisher Scientific, Bremen, Germany). The instrument was operated in positive mode, with a spray voltage at 3.0 kV, and interfaced with a heated electrospray ionization probe (HESI II). Spectra were analyzed using the acquisition software Xcalibur ver. 4.0 (Thermo Scientific, San Jose, CA, USA). Büchi-Tottoli apparatus (Büchi Labortechnik AG, Flawil, Switzerland) was used to measure melting points.

Column chromatography was carried out on SiO_2_ (silica gel 60 Merck 0.063–0.200 mm). Thin-layer chromatography (TLC) was carried out on silica gel 60 F 254 aluminum plates, Merck. Layer thickness 0.200 mm. Chromatograms were visualized under UV at 254 and 365 nm. All reagents were of analytical grade and were used as received without further purification. Solvents were dried according to the literature procedures [76]. The starting scaffolds **1a**–**c** and **3a**–**g** were prepared according to the literature procedures [27,61,62,63,64,65].

The HRMS analyses were successfully obtained for most of the final conjugates **5**. For compounds **5a**, **5h**, **5l**, and **5o**, satisfactory HRMS data could not be obtained despite repeated attempts, most likely because of poor ionization efficiency and/or partial decomposition under the ionization conditions. Structural assignment of these compounds was therefore supported by the available NMR and ESI-MS data.

### 4.2. Synthesis

#### 4.2.1. Synthesis of 5-Methoxy-1H-Indazole-3-Carbaldehyde

To a solution of NaNO_2_ (550 mg, 8 mmol) in 4 mL of distilled water and 3 mL of DMF at 0 °C, was added slowly HCl (1.33 mL of 2 M, 2.7 mmol). The resulting mixture was kept at 0 °C. A solution of 5-methoxy-indole (1 mmol, 1 equiv) in DMF (3 mL) was then added at 0 °C and the reaction mixture was stirred for 2 h and then extracted with ethyl acetate (3 × 20 mL). The combined organic extracts were washed with water, dried over anhydrous Na_2_SO_4_ and the solvent was evaporated. The resulting crude mixture was purified by column chromatography using hexane/EtOAc (8:2).

**5-methoxy-1*H*-indazole-3-carbaldehyde, 1d**

Yield: 82%. M.p.: 215.0–216.0 °C. ^1^H NMR (300 MHz, DMSO-*d*_6_): δ 14.09 (s, 1H, *N*H), 10.17 (s, 1H, CHO), 7.62 (dd, *J* = 9.0, 0.6 Hz, 1H), 7.50 (d, *J* = 2.4 Hz, 1H), 7.13 (dd, *J* = 9.0, 2.4 Hz, 1H), 3.83 (s, 3H, OCH_3_) ppm. ^13^C NMR (75 MHz, DMSO-*d*_6_): δ 187.9, 157.1, 143.4, 137.4, 121.7, 119.9, 112.8, 100.0, 55.8 ppm.

#### 4.2.2. Alkylation of Nitroindazoles **1a–c**

In a round-bottom flask, each nitroindazole **1a**–**c** (1 mmol) was dissolved in DMF (2 mL) and then potassium carbonate (1.5 mmol) was added. After leaving the mixture under stirring for 15 min, propargyl bromide (1.5 mmol) was added dropwise. The reaction mixture was maintained under stirring for 1.5 h under reflux. After this period, TLC control confirmed the disappearance of the starting material and the formation of a main product. The reaction was then poured into ice-water, the solid was filtered and dried. The resulting solids were purified by column chromatography using hexane/EtOAc (8:2).

**4-nitro-1-(prop-2-yn-1-yl)-1*H*-indazole, 2a**

Yield: 74%. M.p: 87–89 °C. ^1^H NMR (300 MHz, DMSO-*d*_6_): δ 8.56 (d, *J* = 1.0 Hz, 1H), 8.31 (dt, *J* = 8.4, 1.0 Hz, 1H), 8.22 (dd, *J* = 7.7, 0.7 Hz, 1H), 7.71 (dd, *J* = 8.4, 7.7 Hz, 1H), 5.52 (d, *J* = 2.5 Hz, 2H), 3.48 (t, *J* = 2.5 Hz, 1H) ppm. ^13^C NMR (75 MHz, DMSO-*d*_6_): δ 141.0, 140.3, 132.9, 126.8, 119.3, 118.4, 116.8, 78.5, 76.8 ppm.

**5-nitro-1-(prop-2-yn-1-yl)-1*H*-indazole, 2b**

Yield: 80%. M.p.: 82–84 °C. ^1^H NMR (300 MHz, DMSO-*d*_6_): δ 8.84 (dd, *J* = 2.2, 1.0 Hz, 1H), 8.44 (d, *J* = 1.0 Hz, 1H), 8.29 (dd, *J* = 9.3, 2.2 Hz, 1H), 7.94 (dt, *J* = 9.3, 1.0 Hz, 1H), 5.47 (d, *J* = 2.6 Hz, 2H), 3.48 (t, *J* = 2.6 Hz, 1H) ppm. ^13^C NMR (75 MHz, DMSO-*d*_6_): δ 142.4, 141.2, 137.3, 123.5, 121.8, 119.6, 111.0, 78.4, 76.6 ppm.

**6-nitro-1-(prop-2-yn-1-yl)-1H-indazole, 2c**

Yield: 75%. M.p.: 163.0–163.6 °C. ^1^H NMR (300 MHz, DMSO-*d*_6_): δ 8.83 (dt, *J* = 1.9, 1.0 Hz, 1H), 8.38 (d, *J* = 1.0 Hz, 1H), 8.05 (dd, *J* = 8.9, 1.0 Hz, 1H), 8.00 (dd, *J* = 8.9, 1.9 Hz, 1H), 5.56 (d, *J* = 2.5 Hz, 2H), 3.47 (t, *J* = 2.5 Hz, 1H) ppm. ^13^C NMR (75 MHz, DMSO-*d*_6_): δ 146.6, 138.1, 134.9, 127.5, 123.1, 115.9, 107.0, 78.5, 76.5 ppm.

#### 4.2.3. Alkylation of 5-Methoxy-1H-Indazole-3-Carbaldehyde

In a round-bottom flask, 5-methoxy-1*H*-indazole-3-carbaldehyde **1d** (1 mmol) was dissolved in DMF (2 mL) and then potassium carbonate (1.5 mmol) was added. After leaving the mixture under stirring for 15 min, propargyl bromide (1.5 mmol) was added dropwise. The reaction mixture was maintained under stirring for 1 h at room temperature and, after this period, poured into crushed ice (10 g). The resulting solid was filtered and purified by column chromatography using hexane/EtOAc (8:2).

**5-methoxy-1-(prop-2-yn-1-yl)-1*H*-indazole-3-carbaldehyde, 2d**

Yield: 90%. M.p.: 108.8–109.3 °C. ^1^H NMR (300 MHz, DMSO-*d*_6_): δ 10.14 (s, 1H), 7.82 (d, *J* = 9.2 Hz, 1H), 7.51 (d, *J* = 2.4 Hz, 1H), 7.23 (dd, *J* = 9.2, 2.4 Hz, 1H), 5.53 (d, *J* = 2.5 Hz, 2H), 3.85 (s, 3H), 3.55 (t, *J* = 2.5 Hz, 1H) ppm. ^13^C NMR (75 MHz DMSO-*d*_6_): δ 187.3, 157.5, 142.4, 136.5, 122.7, 120.1, 112.6, 100.4, 79.9, 77.3, 55.9 ppm.

#### 4.2.4. Synthesis of Azides **4a–g**

In a round-bottom flask, a mixture of the adequate 2-chloro-*N*-(6X-benzo[*d*]thiazol-2-yl)acetamide (0.05 g) and sodium azide (10 equiv) in 25 mL of DMF was maintained under stirring for 2 h. After this period, the resulting mixture was poured into crushed ice (10 g). The resulting solid was filtered and purified by column chromatography using hexane/EtOAc (8:2).

**2-azido-*N*-(benzo[*d*]thiazol-2-yl)acetamide, 4a**

Yield: 88%. M.p.: 181.6–182.5 °C. ^1^H NMR (300 MHz, DMSO-*d*_6_): δ 12.59 (s, 1H), 8.01 (dd, *J* = 7.7, 1.4 Hz, 1H), 7.77 (d, *J* = 7.7 Hz, 1H), 7.46 (td, *J* = 7.7, 1.4 Hz, 1H), 7.33 (td, *J* = 7.7, 1.4 Hz, 1H), 4.26 (s, 2H) ppm. ^13^C NMR (75 MHz, DMSO-*d*_6_): δ 168.3, 157.9, 148.9, 131.9, 126.7, 124.2, 122.3, 121.1, 51.2 ppm. MS-ESI(+): *m*/*z* 234.1 [M + H]^+^.

**2-azido-*N*-(6-(trifluoromethoxy)benzo[*d*]thiazol-2-yl)acetamide, 4b**

Yield: 95%. M.p.: 158.5–159.0 °C. ^1^H NMR (300 MHz, DMSO-*d*_6_): δ 12.71 (s, 1H), 8.15 (d, *J* = 1.4 Hz, 1H), 7.85 (d, *J* = 8.8 Hz, 1H), 7.44 (ddd, *J* = 8.8, 2.4, 1.4 Hz, 1H), 4.28 (s, 2H) ppm. ^13^C NMR (75 MHz, DMSO-*d*_6_): δ 168.6, 159.4, 147.9, 144.6, 133.1, 122.2, 120.5, 118.9, 115.5, 51.2 ppm.

**2-azido-*N*-(6-methoxybenzo[*d*]thiazol-2-yl)acetamide, 4c**

Yield: 75%; M.p.: 169.0–169.7 °C. ^1^H NMR (300 MHz, DMSO-*d*_6_): δ 12.46 (s, 1H), 7.66 (d, *J* = 8.8 Hz, 1H), 7.60 (d, *J* = 2.6 Hz, 1H), 7.04 (dd, *J* = 8.8, 2.6 Hz, 1H), 4.24 (s, 2H), 3.82 (s, 3H) ppm. ^13^C NMR (75 MHz, DMSO-*d*_6_): δ 167.9, 156.7, 143.0, 133.2, 129.9, 121.8, 115.5, 105.2, 57.0, 51.1 ppm. **MS-ESI(+)**: *m*/*z* 264.1 [M + H]^+^.

**2-azido-*N*-(6-nitrobenzo[*d*]thiazol-2-yl)acetamide, 4d**

Yield: 80%; M.p.: 189.2–190.9 °C. ^1^H NMR (300 MHz, DMSO-*d*_6_): δ 9.07 (d, *J* = 2.7 Hz, 1H), 8.29 (dd, *J* = 8.9, 2.7 Hz, 1H), 7.91 (d, *J* = 8.9 Hz, 1H), 4.30 (s, 2H) ppm. ^13^C NMR (75 MHz, DMSO-*d*_6_): δ 169.2, 163.7, 153.8, 143.5, 132.6, 122.30, 121.2, 119.4, 51.4 ppm. MS-ESI(+): *m*/*z* 279.1 [M + H]^+^.

**2-azido-*N*-(6-fluorobenzo[*d*]thiazol-2-yl)acetamide, 4e**

Yield: 83%. M.p.: 178.3–179.1 °C. ^1^H NMR (300 MHz, DMSO-*d*_6_): δ 12.61 (s, 1H), 7.92 (dd, *J* = 8.7, 2.7 Hz, 1H), 7.77 (dd, *J* = 9.0, 4.8 Hz, 1H), 7.30 (td, *J* = 9.0, 2.7 Hz, 1H), 4.26 (s, 2H) ppm. ^13^C NMR (75 MHz, DMSO-*d*_6_): δ 168.7, 159.0, 148.0, 133.8, 128.4, 127.2, 122.5, 122.2, 51.4 ppm. MS-ESI(+): *m*/*z* 252.1 [M + H]^+^.

**2-azido-*N*-(6-chlorobenzo[*d*]thiazol-2-yl)acetamide, 4f**

Yield: 85%. M.p.: 189.7–191.0 °C. ^1^H NMR (300 MHz, DMSO-*d*_6_): δ 12.71 (s, 1H), 8.16 (d, *J* = 2.2 Hz, 1H), 7.76 (d, *J* = 8.6 Hz, 1H), 7.47 (dd, *J* = 8.6, 2.2 Hz, 1H), 4.27 (s, 2H) ppm. ^13^C NMR (75 MHz, DMSO-*d*_6_): δ 168.5, 158.8, 147.8, 133.6, 128.2, 127.0, 122.3, 122.0, 51.3 ppm. MS-ESI(+): *m*/*z* 268.0 [M + H]^+^.

**2-azido-*N*-(6-bromobenzo[*d*]thiazol-2-yl)acetamide, 4g**

Yield: 90%. M.p.: 205.5–206.4 °C. ^1^H NMR (300 MHz, DMSO-*d*_6_) δ 12.69 (s, 1H), 8.29 (d, *J* = 2.2 Hz, 1H), 7.71 (d, *J* = 8.6 Hz, 1H), 7.59 (dd, *J* = 8.6, 2.2 Hz, 1H), 4.27 (s, 2H) ppm. ^13^C NMR (75 MHz, DMSO-*d*_6_) δ 168.5, 158.7, 148.0, 134.0, 129.8, 124.8, 122.8, 116.2, 51.2 ppm. MS-ESI(+): *m*/*z* 312.0 [M + H]^+^.

#### 4.2.5. Synthesis of Benzothiazole–Triazole–Indazole Conjugates **5a–s**

To a stirred solution of each azide (0.085 mmol) and the appropriate terminal alkyne (0.085 mmol) in DMF (2.0 mL) was added copper(II) sulfate (0.01mmol) and sodium ascorbate (0.02 mmol). The reaction mixture was maintained under stirring at room temperature until the TLC control showed the total consumption of the starting material (4 h). After completion, the reaction mixture was poured into ice-water and the resulting precipitate was collected by filtration and washed with water. The residues were purified by recrystallization from DMSO/ethanol.

***N*-(benzo[*d*]thiazol-2-yl)-2-(4-((4-nitro-1*H*-indazol-1-yl)methyl)-1*H*-1,2,3-triazol-1-yl)acetamide, 5a**

Yield: 89%. M.p.: 135.4–136.0 °C. ^1^H NMR (500 MHz, DMSO-*d*_6_): δ 12.86 (s, 1H), 8.55 (s, 1H), 8.42 (d, *J* = 8.2 Hz, 1H), 8.20–8.21 (m, 2H), 7.97 (d, *J* = 7.8 Hz, 1H), 7.77 (d, *J* = 7.8 Hz, 1H), 7.70 (t, *J* = 8.2 Hz, 1H), 7.45 (t, *J* = 7.8 Hz, 1H), 7.32 (t, *J* = 7.8 Hz, 1H), 5.92 (s, 2H), 5.49 (s, 2H) ppm. ^13^C NMR (125 MHz, DMSO-*d*_6_): δ 166.3, 162.9, 157.8, 148.9, 142.9, 141.4, 140.2, 132.6, 131.9, 126.8, 126.5, 126.0, 124.4, 122.3, 119.2, 118.9, 116.8, 55.4, 52.2 ppm. MS-ESI(+): *m*/*z* calc for C_19_H_14_N_8_O_3_S: 435.1 [M + H]^+^ found 435.2.

***N*-(6-methoxybenzo[*d*]thiazol-2-yl)-2-(4-((4-nitro-1*H*-indazol-1-yl)methyl)-1*H*-1,2,3-triazol-1-yl)acetamide, 5b**

Yield: 79%. M.p.: 233.4–233.9 °C. ^1^H NMR (300 MHz, DMSO-*d*_6_): δ 12.76 (s, 1H), 8.55 (s, 1H), 8.45 (d, *J* = 8.4 Hz, 1H), 8.24–8.18 (m, 2H), 7.75–7.63 (m, 2H), 7.58 (d, *J* = 2.6 Hz, 1H), 7.04 (dd, *J* = 8.8, 2.6 Hz, 1H), 5.94 (s, 2H), 5.48 (s, 2H), 3.80 (s, 3H) ppm. ^13^C NMR: not obtained despite repeated attempts because of limited solubility in the available deuterated solvents. MS-ESI(+): *m*/*z* 465.2 [M + H]^+^. HRMS-ESI(+): *m*/*z* calc for C_20_H_17_N_8_O_4_S: 465.1088 [M + H]^+^ found 465.1091.

**2-(4-((4-nitro-*1H*-indazol-1-yl)methyl)-1*H*-1,2,3-triazol-1-yl)-*N*-(6-(trifluoromethoxy)benzo[*d*]thiazol-2-yl)acetamide, 5c**

Yield: 95%; m.p.: 139.7–140.2 °C. ^1^H NMR (300 MHz, DMSO-*d*_6_): δ 12.98 (s, 1H), 8.55 (s, 1H), 8.45 (d, *J* = 8.3 Hz, 1H), 8.29–8.18 (m, 2H), 8.14 (s, 1H), 7.87 (d, *J* = 7.5 Hz, 1H), 7.71 (t, *J* = 8.3 Hz, 1H), 7.44 (d, *J* = 8.3 Hz, 1H), 5.94 (s, 2H), 5.53 (s, 2H) ppm. ^13^C NMR: not obtained despite repeated attempts because of limited solubility in the available deuterated solvents. MS-ESI(+): *m*/*z* 519.1 [M + H]^+^. HRMS-ESI(+): *m*/*z* calc for C_20_H_14_N_8_O_4_F_3_S: 519.0805 [M + H]^+^ found 519.0806.

***N*-(6-fluorobenzo[*d*]thiazol-2-yl)-2-(4-((4-nitro-1*H*-indazol-1-yl)methyl)-1*H*-1,2,3-triazol-1-yl)acetamide, 5d**

Yield: 76%. M.p.: 250.6–251.1 °C. ^1^H NMR (500 MHz, DMSO-*d*_6_): δ 12.89 (s, 1H), 8.55 (s, 1H), 8.43 (d, *J* = 8.3 Hz, 1H), 8.23–8.19 (m, 2H), 7.90 (dd, *J* = 8.9, 2.8 Hz, 1H), 7.79 (dd, *J* = 8.9, 4.8 Hz, 1H), 7.70 (t, *J* = 8.3 Hz, 1H), 7.31 (td, *J* = 8.9, 2.8 Hz, 1H), 5.93 (s, 2H), 5.49 (s, 2H) ppm. ^13^C NMR (125 MHz, DMSO-*d*_6_): δ 166.2, 160.2, 158.3, 157.9, 145.6, 142.9, 141.4, 140.2, 133.2, 132.6, 126.6, 126.0, 122.4, 119.2, 118.8, 116.7, 115.0, 114.8, 108.9, 108.7, 52.0, 44.7 ppm. MS-ESI(+): *m*/*z* 453.2 [M + H]^+^. HRMS-ESI(+): *m*/*z* calc for C_19_H_14_N_8_O_3_FS: 453.0888 [M + H]^+^ found 453.0894.

***N*-(6-chlorobenzo[*d*]thiazol-2-yl)-2-(4-((4-nitro-1*H*-indazol-1-yl)methyl)-1*H*-1,2,3-triazol-1-yl)acetamide, 5e**

Yield: 81%; M.p.: 236.1–236.5 °C. ^1^H NMR (300 MHz, DMSO-*d*_6_): δ 12.95 (s, 1H), 8.55 (s, 1H), 8.43 (d, *J* = 8.3 Hz, 1H), 8.24–8.10 (m, 3H), 7.77 (broad d, 1H), 7.70 (t, *J* = 8.3 Hz, 1H), 7.47 (broad d, 1H), 5.93 (s, 2H), 5.50 (s, 2H) ppm. ^13^C NMR (125 MHz, DMSO-*d*_6_): δ 162.9, 140.2, 133.7, 129.0, 126.5, 126.2, 122.7, 119.2, 118.9, 116.8, 44.7, 36.3 ppm. MS-ESI(+): *m*/*z* 469.3 [M + H]^+^. HRMS-ESI(+): *m*/*z* calc for C_19_H_14_N_8_O_3_ClS: 469.0593 [M + H]^+^ found 469.0594.

***N*-(6-methoxybenzo[*d*]thiazol-2-yl)-2-(4-((5-nitro-1*H*-indazol-1-yl)methyl)-1*H*-1,2,3-triazol-1-yl)acetamide, 5f**

Yield: 77%; M.p.: 223.0–223.6 °C. ^1^H NMR (500 MHz, DMSO-*d*_6_): δ 12.72 (s, 1H), 8.85 (d, *J* = 2.5 Hz, 1H), 8.44 (s, 1H), 8.27 (dd, *J* = 9.3, 2.5 Hz, 1H), 8.19 (s, 1H), 8.03 (d, *J* = 9.3 Hz, 1H), 7.66 (d, *J* = 8.8 Hz, 1H), 7.55 (d, *J* = 2.8 Hz, 1H), 7.04 (dd, *J* = 8.8, 2.8 Hz, 1H), 5.85 (s, 2H), 5.47 (s, 2H), 3.78 (s, 3H) ppm. ^13^C NMR (125 MHz, DMSO-*d*_6_): δ 165.9, 156.8, 155.8, 142.8, 142.3, 141.6, 137.1, 133.2, 126.0, 123.5, 121.8, 121.6, 119.6, 115.7, 111.4, 105.1, 56.0, 52.0, 44.5 ppm. MS-ESI(+): *m*/*z* 465.2 [M + H]^+^. HRMS-ESI(+): *m*/*z* calc for C_20_H_17_N_8_O_4_S: 465.1088 [M + H]^+^ found 465.1091.

**2-(4-((5-nitro-1*H*-indazol-1-yl)methyl)-1*H*-1,2,3-triazol-1-yl)-*N*-(6-(trifluoromethoxy)benzo[*d*]thiazol-2-yl)acetamide, 5g**

Yield: 90%; M.p.: 179.3–180.0 °C. ^1^H NMR (300 MHz, DMSO-*d*_6_): δ 12.98 (s, 1H), 8.85 (d, *J* = 2.2 Hz, 1H), 8.44 (s, 1H), 8.29 (dd, *J* = 9.3, 2.2 Hz, 1H), 8.21 (s, 1H), 8.14 (broad s, 1H), 8.07 (d, *J* = 9.3 Hz, 1H), 7.87 (d, *J* = 9.2 Hz, 1H), 7.44 (broad d, 1H), 5.89 (s, 2H), 5.52 (s, 2H) ppm. ^13^C NMR (125 MHz, DMSO-*d*_6_): δ 166.2, 160.2, 158.3, 157.9, 145.5, 142.3, 141.7, 137.2, 133.2, 126.1, 123.5, 122.4, 122.3, 121.6, 119.6, 115.1, 114.9, 111.4, 108.9, 108.6, 52.0, 44.5 ppm. MS-ESI(+): *m*/*z* 519.1 [M + H ]^+^. HRMS-ESI(+): *m*/*z* calc for C_20_H_14_N_8_O_4_F_3_S: 519.0805 [M + H]^+^ found 519.0810.

**2-(4-((5-nitro-1*H*-indazol-1-yl)methyl)-1*H*-1,2,3-triazol-1-yl)-*N*-(6-nitrobenzo[*d*]thiazol-2-yl)acetamide, 5h**

Yield: 82%. M.p.: 247.9–248.3 °C. ^1^H NMR (500 MHz, DMSO-*d*_6_): δ 13.31 (s, 1H), 9.08 (s, 1H), 8.85 (d, *J* = 2.5 Hz, 1H), 8.44 (s, 1H), 8.32–8.26 (m, 2H), 8.21 (s, 1H), 8.06 (d, *J* = 9.3 Hz, 1H), 7.97–7.91 (m, 1H), 5.89 (s, 2H), 5.55 (s, 2H) ppm. ^13^C NMR (125 MHz, DMSO-*d*_6_): δ 162.5, 147.6, 143.6, 142.7, 142.3, 141.7, 137.0, 125.9, 123.5, 122.3, 121.6, 121.3, 119.7, 119.6, 111.5, 52.4, 44.6 ppm. MS-ESI(+): *m*/*z* calc for C_19_H_12_LiN_9_O_5_S: 485.1 [M + Li-H]^+●^ found 485.6.

***N*-(6-fluorobenzo[*d*]thiazol-2-yl)-2-(4-((5-nitro-1*H*-indazol-1-yl)methyl)-1*H*-1,2,3-triazol-1-yl)acetamide, 5i**

Yield: 77%. M.p.: 130.1–130.5 °C. ^1^H NMR (500 MHz, DMSO-*d*_6_): δ 12.93 (s, 1H), 8.84 (d, *J* = 2.5 Hz, 1H), 8.44 (s, 1H), 8.27 (dd, *J* = 9.3, 2.5 Hz, 1H), 8.20 (s, 1H), 8.03 (d, *J* = 9.3 Hz, 1H), 7.88 (dd, *J* = 8.9, 2.9 Hz, 1H), 7.78 (dd, *J* = 8.9, 4.7 Hz, 1H), 7.30 (td, *J* = 8.9, 2.9 Hz, 1H), 5.85 (s, 2H), 5.49 (s, 2H) ppm. ^13^C NMR (125 MHz, DMSO-*d*_6_): δ 166.2, 160.2, 158.3, 157.9, 145.5, 142.3, 141.7, 137.2, 133.1, 126.1, 123.5, 122.4 (d, *J* = 10.0 Hz), 121.6, 119.6, 114.9 (d, *J* = 24.5 Hz), 111.4, 108.7 (d, *J* = 27.2 Hz), 52.0, 44.5 ppm. MS-ESI(+): *m*/*z* 453.2 [M + H ]^+^. HRMS-ESI(+): *m*/*z* calc for C_19_H_14_N_8_O_3_FS: 453.0888 [M + H]^+^ found 453.0878.

***N*-(6-chlorobenzo[*d*]thiazol-2-yl)-2-(4-((5-nitro-1*H*-indazol-1-yl)methyl)-1*H*-1,2,3-triazol-1-yl)acetamide, 5j**

Yield: 72%. M.p.: 193.6–194.6 °C. ^1^H NMR (500 MHz, DMSO-*d*_6_): δ 12.95 (s, 1H), 8.85 (d, *J* = 2.3 Hz, 1H), 8.45 (s, 1H), 8.29 (dd, *J* = 9.2, 2.3 Hz, 1H), 8.21 (s, 1H), 8.12–8.16 (m, 1H), 8.06 (d, *J* = 9.2 Hz, 1H), 7.75–7.80 (m, 1H), 7.47 (dd, *J* = 6.1, 2.4 Hz, 1H), 5.88 (s, 2H), 5.51 (s, 2H) ppm. ^13^C NMR (125 MHz, DMSO-*d*_6_): δ 166.8, 162.8, 158.7, 147.9, 142.3, 137.5, 133.6, 128.4, 127.0, 126.2, 122.5, 122.0, 121.6, 119.6, 111.5, 52.1, 44.6 ppm. MS-ESI(+): *m*/*z* 469.3 [M + H]^+^. HRMS-ESI(+): *m*/*z* calc for C_19_H_14_N_8_O_3_ClS: 469.0593 [M + H]^+^ found 469.0584. Minor additional signals (δ ≈ 4.4, 3.4, and 1.1 ppm) correspond to residual ethanol from recrystallization, consistent with the limited solubility of this compound; they fall outside the integrated structural assignment.

***N*-(6-bromobenzo[*d*]thiazol-2-yl)-2-(4-((5-nitro-1*H*-indazol-1-yl)methyl)-1*H*-1,2,3-triazol-1-yl)acetamide, 5k**

Yield: 81%. M.p.: 230.7–231.3 °C. ^1^H NMR (300 MHz, DMSO-*d*_6_): δ 12.96 (s, 1H), 8.85 (d, *J* = 2.3 Hz, 1H), 8.44 (s, 1H), 8.24–8.32 (m, 2H), 8.20 (s, 1H), 8.06 (d, *J* = 9.2 Hz, 1H), 7.66–7.77 (m, 1H), 7.55–7.64 (m, 1H), 5.89 (s, 2H), 5.51 (s, 2H) ppm. ^13^C NMR (125 MHz, DMSO-*d*_6_): δ 166.2, 157.8, 148.9, 142.8, 142.3, 141.6, 137.1, 131.9, 126.8, 126.0, 124.3, 123.4, 122.3, 121.6, 121.3, 119.6, 111.5, 52.1, 44.6. MS-ESI(+): *m*/*z* 513.2 [M + H]^+^. HRMS-ESI(+): *m*/*z* calc for C_19_H_14_N_8_O_3_BrS: 513.0087 [M + H]^+^ found 513.0094.

***N*-(benzo[*d*]thiazol-2-yl)-2-(4-((6-nitro-1*H*-indazol-1-yl)methyl)-1*H*-1,2,3-triazol-1-yl)acetamide, 5l**

Yield: 83%. M.p.: 244.4–245.1 °C. ^1^H NMR (300 MHz, DMSO-*d*_6_): δ 12.84 (s, 1H), 8.97 (s, 1H), 8.36 (s, 1H), 8.21 (s, 1H), 8.06–7.95 (m, 3H), 7.78 (broad doublet, 1H), 7.46 (t, *J* = 7.9 Hz, 1H), 7.32 (t, *J* = 7.9 Hz, 1H), 5.98 (s, 2H), 5.51 (s, 2H) ppm. ^13^C NMR (75 MHz, DMSO-*d*_6_): δ 164.7, 146.7, 145.0, 123.0, 122.3, 120.4, 115.7, 115.1, 107.2, 52.5, 44.4 ppm. MS-ESI(+): *m*/*z* calc for C_19_H_15_N_8_O_3_S 435.1 [M + H]^+^ found 435.2.

**2-(4-((6-nitro-1*H*-indazol-1-yl)methyl)-1*H*-1,2,3-triazol-1-yl)-*N*-(6-(trifluoromethoxy)benzo[*d*]thiazol-2-yl)acetamide, 5m**

Yield: 92%. M.p.: 191.1–191.9 °C. ^1^H NMR (300 MHz, DMSO-*d*_6_): δ 12.97 (s, 1H), 8.96 (s, 1H), 8.36 (s, 1H), 8.21 (s, 1H), 8.13 (bs, 1H), 8.06–8.02 (m, 1H), 8.01 (d, *J* = 1.9 Hz, 1H), 7.87 (broad d, 1H), 7.45 (broad d, 1H), 5.98 (s, 2H), 5.53 (s, 1H) ppm. ^13^C NMR (75 MHz, DMSO-*d*_6_): δ 163.5, 159.5, 147.7, 146.5, 144.9, 142.4, 122.9, 122.3, 120.6, 115.7, 115.4, 107.4, 52.3, 44.6, 36.6 ppm. MS-ESI(+): *m*/*z* 519.1 [M + H]^+^. HRMS-ESI(+): *m*/*z* calc for C_20_H_14_N_8_O_4_F_3_S: 519.0805 [M + H]^+^ found 519.0806.

***N*-(benzo[*d*]thiazol-2-yl)-2-(4-((3-formyl-5-methoxy-1*H*-indazol-1-yl)methyl)-1*H*-1,2,3-triazol-1-yl)acetamide, 5n**

Yield: 95%. M.p.: 203.3–203.8 °C. ^1^H NMR (300 MHz, DMSO-*d*_6_): δ 12.85 (s, 1H), 10.14 (s, 1H), 8.26 (s, 1H), 7.99 (d, *J* = 7.5 Hz, 1H), 7.94 (d, *J* = 9.4 Hz, 1H), 7.78 (d, *J* = 7.5 Hz, 1H), 7.52 (d, *J* = 2.4 Hz, 1H), 7.46 (t, *J* = 7.5 Hz, 1H), 7.33 (t, *J* = 7.5 Hz, 1H), 7.23 (dd, *J* = 9.4, 2.4 Hz, 1H), 5.94 (s, 2H), 5.52 (s, 2H), 3.85 (s, 3H) ppm. ^13^C NMR (75 MHz, DMSO-*d*_6_): δ 187.2, 166.4, 157.5, 148.9, 142.2, 136.9, 131.9, 129.0, 126.8, 126.2, 124.3, 122.7, 122.3, 119.9, 113.0, 100.3, 55.9, 52.2, 45.5 ppm. MS-ESI(+): *m*/*z* 448.2 [M + H]^+^. HRMS-ESI(+): *m*/*z* calc for C_21_H_18_N_7_O_3_S: 448.1186 [M + H]^+^ found 448.1189.

**2-(4-((3-formyl-5-methoxy-1*H*-indazol-1-yl)methyl)-1*H*-1,2,3-triazol-1-yl)-*N*-(6-methoxybenzo[*d*]thiazol-2-yl)acetamide, 5o**

Yield: 75%. M.p.: 217.6–218.2 °C. ^1^H NMR (300 MHz, DMSO-*d*_6_): δ 12.72 (s, 1H), 10.14 (s, 1H), 8.26 (s, 1H), 7.94 (d, *J* = 9.2 Hz, 1H), 7.67 (d, *J* = 8.8 Hz, 1H), 7.58 (d, *J* = 2.7 Hz, 1H), 7.52 (d, *J* = 2.4 Hz, 1H), 7.22 (dd, *J* = 9.2, 2.4 Hz, 1H), 7.05 (dd, *J* = 8.8, 2.7 Hz, 1H), 5.94 (s, 2H), 5.49 (s, 2H), 3.85 (s, 3H), 3.80 (s, 3H) ppm. ^13^C NMR (75 MHz, DMSO-*d*_6_): δ 187.3, 165.9, 157.5, 156.8, 155.8, 142.2, 136.8, 133.2, 126.2, 122.7, 121.8, 119.9, 115.7, 112.9, 107.4, 105.1, 100.3, 56.0, 55.9, 52.1, 45.5 ppm. MS-ESI(+): *m*/*z* calc for C_22_H_20_N_7_O_4_S: 478.1 [M + H]^+^ found 478.2.

**2-(4-((3-formyl-5-methoxy-1*H*-indazol-1-yl)methyl)-1*H*-1,2,3-triazol-1-yl)-*N*-(6-(trifluoromethoxy)benzo[*d*]thiazol-2-yl)acetamide, 5p**

Yield: 94%. M.p.: 168.1–168.7 °C. ^1^H NMR (300 MHz, DMSO-*d*_6_): δ 12.98 (s, 1H), 10.14 (s, 1H), 8.26 (s, 1H), 8.14 (bs, 1H), 7.94 (d, *J* = 9.2 Hz, 1H), 7.87 (d, *J* = 8.7 Hz, 1H), 7.52 (d, *J* = 2.5 Hz, 1H), 7.44 (dd, *J* = 8.7, 1.8 Hz, 1H), 7.22 (dd, *J* = 9.2, 2.5 Hz, 1H), 5.94 (s, 2H), 5.54 (s, 2H), 3.85 (s, 3H) ppm. ^13^C NMR (75 MHz, DMSO-*d*_6_): δ 187.3, 166.5, 159.4, 157.5, 147.9, 144.7, 144.1, 142.2, 136.8, 133.1, 126.2, 122.7, 122.3 (d, *J* = 5.5 Hz), 120.5, 119.9, 118.9, 115.6, 113.0, 100.3, 55.9, 52.1, 45.5 ppm. MS-ESI(+): *m*/*z* 532.1 [M + H]^+^. HRMS-ESI(+): *m*/*z* calc for C_22_H_16_N_7_O_4_F_3_S: 531.0937 [M]^+●^ found 531.0955.

**2-(4-((3-formyl-5-methoxy-1*H*-indazol-1-yl)methyl)-1*H*-1,2,3-triazol-1-yl)-*N*-(6-nitrobenzo[*d*]thiazol-2-yl)acetamide, 5q**

Yield: 85%. M.p.: 227.1–227.9 °C. ^1^H NMR (300 MHz, DMSO-*d*_6_): δ 13.27 (s, 1H), 10.14 (s, 1H), 9.08 (s, 1H), 8.34–8.23 (m, 2H), 7.94 (d, *J* = 9.3 Hz, 2H), 7.52 (d, *J* = 2.4 Hz, 1H), 7.23 (dd, *J* = 9.3, 2.4 Hz, 1H), 5.95 (s, 2H), 5.57 (s, 2H), 3.85 (s, 3H) ppm. ^13^C NMR (75 MHz, DMSO-*d*_6_): δ 187.3, 167.7, 166.0, 160.9, 157.5, 143.6, 142.2, 136.8, 130.3, 128.8, 126.3, 122.7, 122.3, 119.9, 119.6, 112.9, 100.3, 55.9, 52.9, 45.9 ppm. MS-ESI(+): *m*/*z* 493.2 [M + H]^+^. HRMS-ESI(+): *m*/*z* calc for C_21_H_17_N_8_O_5_S: 493.1036 [M + H]^+^ found 493.1037.

***N*-(6-fluorobenzo[d]thiazol-2-yl)-2-(4-((3-formyl-5-methoxy-1*H*-indazol-1-yl)methyl)-1*H*-1,2,3-triazol-1-yl)acetamide, 5r**

Yield: 83%. M.p.: 195.0–195.9 °C. ^1^H NMR (300 MHz, DMSO-*d*_6_): δ 12.88 (s, 1H), 10.14 (s, 1H), 8.26 (s, 1H), 7.98–7.74 (m, 3H), 7.51 (d, *J* = 2.5 Hz, 1H), 7.31 (broad t, 1H), 7.22 (dd, *J* = 9.2, 2.5Hz, 1H), 5.94 (s, 2H), 5.53 (s, 2H), 3.85 (s, 3H) ppm. ^13^C NMR (75 MHz, DMSO-*d*_6_): δ 187.3, 166.2, 157.5, 142.3, 136.9, 133.1, 126.4, 122.6, 119.9, 116.3, 112.9, 108.7, 105.4, 100.3, 55.98, 52.2, 45.5 ppm. MS-ESI(+): *m*/*z* 466.2 [M + H]^+^. HRMS-ESI(+): *m*/*z* calc for C_21_H_17_N_7_O_3_FS: 466.1092 [M + H]^+^ found 466.1098.

***N*** **-(6-chlorobenzo[*d*]thiazol-2-yl)-2-(4-((3-formyl-5-methoxy-1*H*-indazol-1-yl)methyl)-1*H*-1,2,3-triazol-1-yl)acetamide, 5s**

Yield: 82%. M.p.: 215.8–216.5 °C. ^1^H NMR (300 MHz, DMSO-*d*_6_): δ 12.94 (s, 1H), 10.14 (s, 1H), 8.26 (s, 1H), 8.14 (broad s, 1H), 7.94 (d, *J* = 9.2 Hz, 1H), 7.78 (broad s, 1H), 7.51 (d, *J* = 2.5 Hz, 1H), 7.48 (broad d, 1H), 7.22 (dd, *J* = 9.2, 2.5 Hz, 1H), 5.94 (s, 2H), 5.52 (s, 2H), 3.85 (s, 3H) ppm. ^13^C NMR (75 MHz, DMSO-*d*_6_): δ 187.2, 157.5, 144.9, 142.2, 136.9, 133.7, 128.4, 127.1, 126.2, 122.7, 122.1, 119.9, 113.0, 100.3, 55.9, 52.4, 45.5 ppm. MS-ESI(+): *m*/*z* 481.4 [M]^+●^. HRMS-ESI(+): *m*/*z* calc for C_21_H_16_N_7_O_3_ClS: 482.0797 [M + H]^+^ found 482.0785.

### 4.3. Biological Assays

#### 4.3.1. Compounds Preparation for the Biological Assay and Culture Conditions

All compounds were prepared using two different dissolution procedures:

(a) DMSO/Fetal Bovine Serum-based preparation. Compounds were initially dissolved in DMSO [Merck Life Science S.r.l., Milano (MI), Italy] at a concentration of 100 mM, using final volumes ranging from 5 to 30 μL depending on compound solubility. The resulting solutions were subsequently diluted with fetal bovine serum (FBS) to a final volume of 500 μL, yielding a compound concentration of 1 mM. From these stock solutions, 10× working solutions were prepared by further dilution in FBS containing the appropriate amount of DMSO. This procedure allowed the preparation of 10× solutions corresponding to the final experimental concentrations. The final concentrations of the compounds tested on cells ranged from 0.01 to 100 μM, while the final DMSO concentration ranged from 0.1% to 0.6%, depending on the solubility of the individual compounds.
(b) DMSO/cremophor–ethanol/saline-based preparation. Compounds were initially dissolved in DMSO as described above. The resulting solutions were then further diluted in normal saline containing 1% cremophor/ethanol (1:1), rather than FBS, together with the appropriate DMSO concentration. The final DMSO concentration was adjusted to 0.1% or 0.2%, while the cremophor/ethanol concentration was maintained at 0.1%. Appropriate vehicle-matched controls containing the corresponding final concentrations of DMSO and/or cremophor/ethanol were included in all experiments.

All compounds, prepared according to the procedures described above, were evaluated in the A2780 human ovarian carcinoma cell line (Interlab Cell Line Collection, ICLC, Genova, Italy; HTL98008), cultured in RPMI medium supplemented with 1% glutamine, 1% penicillin–streptomycin, and 10% FBS (complete medium; Euroclone SpA, Pero, MI, Italy); the A549 human lung carcinoma cell line (ICLC, Genova, Italy; HTL03001), cultured in complete RPMI medium (Euroclone SpA); and the MDA-MB-231 human breast carcinoma cell line, kindly provided by Dr. Antonio Daga (IRCCS Ospedale Policlinico San Martino, Genova, Italy), cultured in DMEM supplemented with 1% glutamine, 1% non-essential amino acids, 1% penicillin–streptomycin, and 10% FBS (Euroclone SpA). Cells were maintained at 37 °C in a humidified atmosphere containing 5% CO_2_. All cell lines were routinely subcultured at split ratios of 1:10 to 1:20 twice weekly, using routine trypsinization procedures for cell detachment and maintenance.

#### 4.3.2. Determination of Antiproliferative Activity (MTT Assay)

Antiproliferative activity was evaluated using the MTT assay. A2780, A549, and MDA-MB-231 cells were seeded in 96-well flat-bottom microplates at densities optimized for each cell line (2700, 1250, and 2900 cells/well, respectively) to maintain exponential growth during the assay. Following seeding, the plates were centrifuged at 275× *g* for 2 min to facilitate cell attachment. After 6–8 h of adhesion, cells were treated with 20 µL of compound solution.

The biological evaluation was performed in two steps. First, all compounds were screened at 30 µM to identify derivatives producing at least 50% inhibition of cell proliferation relative to the corresponding vehicle-treated control. Compounds meeting this criterion were subsequently evaluated over a concentration range of 0.01–100 µM to generate concentration–response curves and determine IC_50_ values whenever reliable curve fitting was possible.

Following a 72 h treatment period, cell viability was assessed using the MTT assay according to previously reported procedures [77,78]. Briefly, 50 μL of MTT solution (2 mg/mL; Sigma-Aldrich, St. Louis, MO, USA) were added to each well and the plates were incubated for 4 h at 37 °C. After centrifugation (275× *g*, 2 min), the supernatant was removed and the resulting formazan crystals were dissolved in 100 μL of DMSO under shaking for 30 min at room temperature and a brief shaking. Absorbance was measured at 540 nm using a 400 ATC microplate reader (SLT Labinstruments, Salzburg, Austria). Vehicle-treated controls were included in all experiments and used as reference for the calculation of percentage growth inhibition.

The half-maximal inhibitory concentration (IC_50_) values were determined by nonlinear regression analysis of the corresponding concentration–response (percent growth inhibition versus compound concentration) curves using either Excel or Cricket Graph 1.0 software. Based on our previous experience and the pharmacological activity observed for this class of compounds, IC_50_ values below 30 μM were considered indicative of biologically relevant antiproliferative activity. Values are expressed as mean ± SD from three to seven independent experiments, depending on the compound and cell line, as indicated in Table 1.

#### 4.3.3. Data Analysis and Activity Classification

Because the primary objective of the biological evaluation was intra-series ranking and preliminary SAR analysis rather than pairwise statistical comparison between all compounds, no formal multiple-comparison statistical testing was applied to the IC_50_ values and therefore reported as mean ± SD obtained from independent experiments.

For SAR ranking and hit prioritization, 30 µM was adopted as the operational activity threshold. Therefore, compounds with fitted or estimated IC_50_ values above 30 µM were not considered active under the present screening criteria and are reported as IC_50_ > 30 µM for ranking purposes. Borderline fitted values slightly above this threshold are indicated in Table 1.

## Figures and Tables

**Scheme 1:**
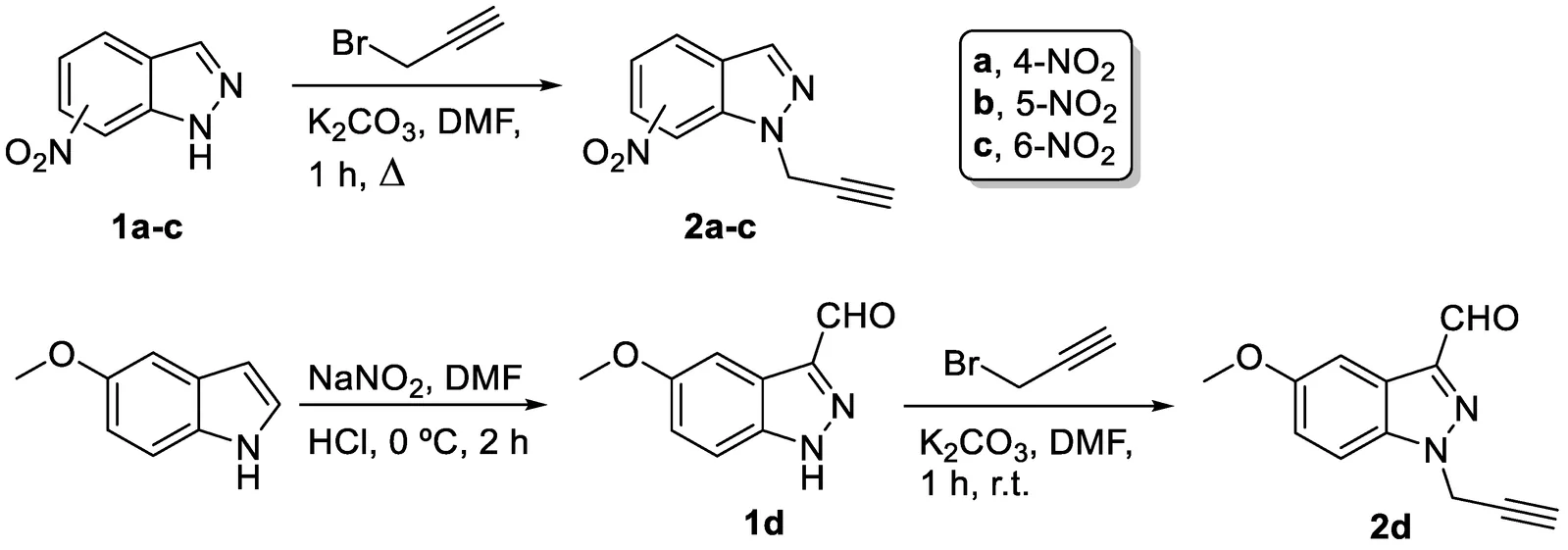
Functionalization of the indazole core with alkynyl units to obtain indazole–alkynyl scaffolds **2a**–**d**.

**Scheme 2:**
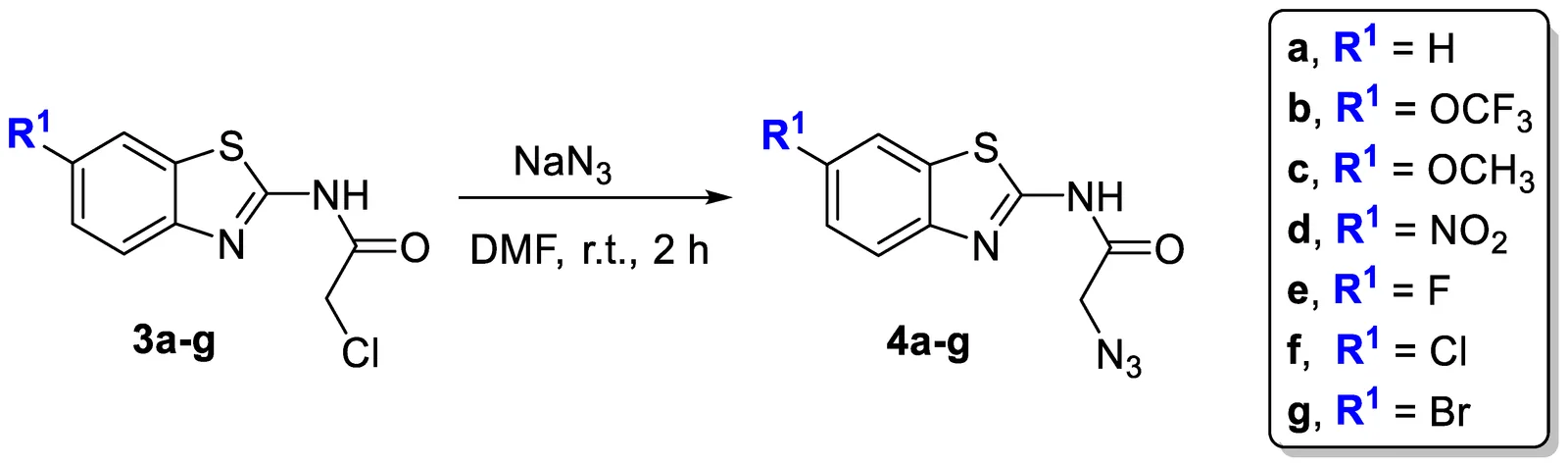
Preparation of benzothiazole–azide precursors **4a**–**g**.

**Scheme 3:**
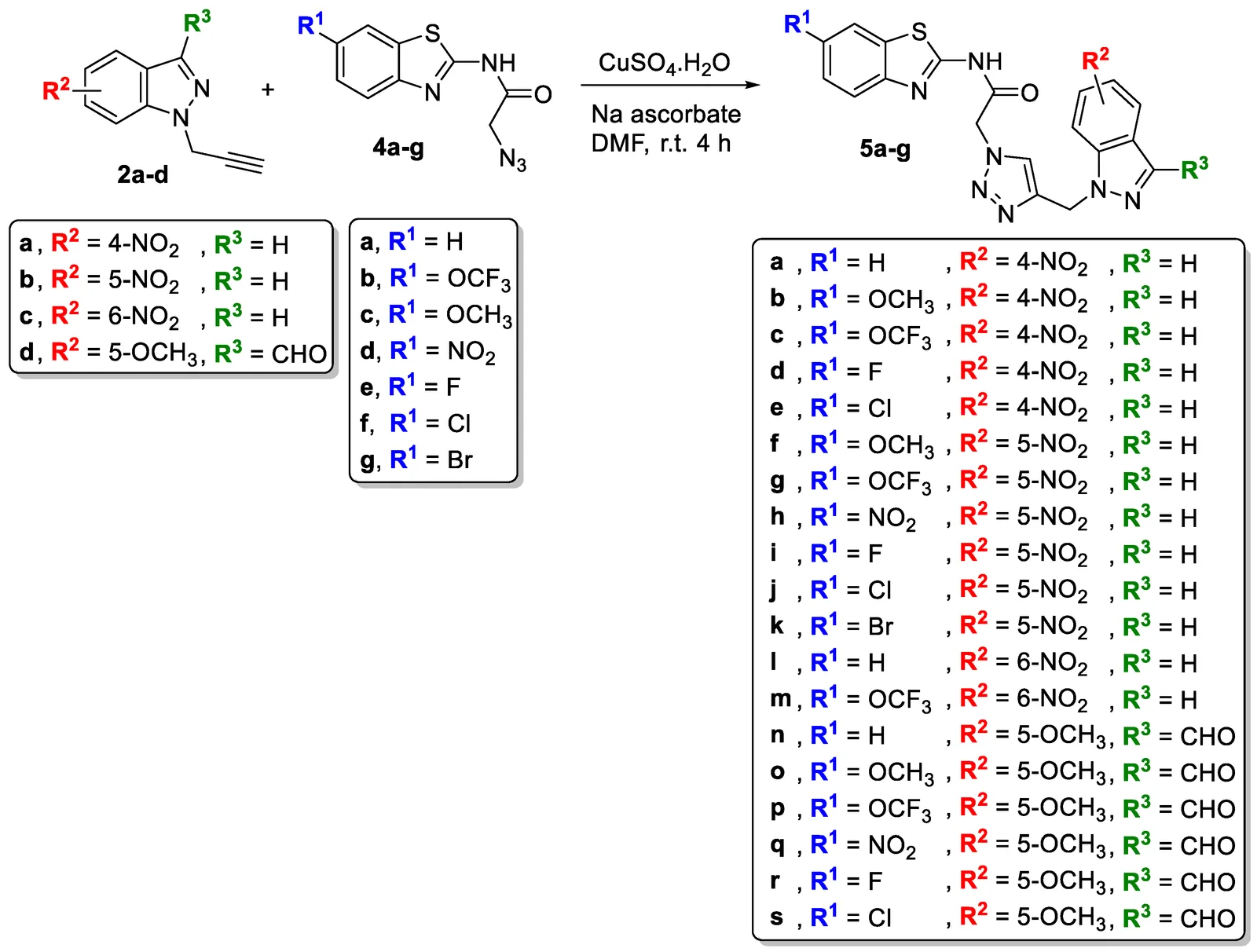
Synthesis of benzothiazole–triazole–indazole conjugates **5a**–**s**.

**Table 1:** Concentrations of compounds inhibiting 50% proliferation, IC_50_, in A2780, A549 and MDA-MB-231 cells after 72 h treatment.

Compd	IC_50_ ± SD ^a^ (μM)		
	A2780	A549	MDA-MB-231
**5a**	20.9 ± 4.1	>30	>30
**5b**	>30	>30	10.8 ± 2.9
**5c**	28.2 ± 0.8	>30	>30
**5d**	25.1 ± 3.7	>30	>30
**5e**	18.2 ± 6.2	>30	>30
**5f**	>30	>30	>30
**5g**	>30	>30	>30
**5h**	>30	>30	>30
**5i**	>30	>30	>30
**5j**	>30	>30	>30
**5k**	>30	>30	>30
**5l**	24.1 ± 5.8	>30	>30
**5m**	>30	>30	>30
**5n**	>30	>30	>30
**5o**	>30	>30	>30
**5p**	29.8 ± 0.4	>30	>30
**5q**	33.9 ± 1.7 ^b^	>30	>30
**5r**	15.7 ± 1.6	>30	>30
**5s**	>30	>30	>30

a—Values (µM) are expressed as mean ± SD from 3 to 7 independent experiments, depending on the compound and cell line. b—Borderline fitted value slightly above the 30 µM operational activity threshold; not considered active for SAR ranking. Compounds reported as IC_50_ > 30 μM did not meet the operational activity threshold adopted for SAR ranking under the present assay conditions.

## Data Availability

The raw data supporting the conclusions of this article will be made available by the authors on request.

## Supplementary Materials

The following supporting information can be downloaded at https://www.mdpi.com/article/10.3390/molecules31193452/s1, Figure S1–S105: NMR and mass spectrometry spectra of compounds **1d**, **2a**–**d**, **4a**–**g** and **5a**–**s**; Table S1: Physicochemical properties, lipophilicity, water solubility, and pharmacokinetics, drug likeness and medicinal chemistry characteristics of compounds obtained in the online platform SwissADME. Table S2: Percent proliferation of A2780, A549 and MDA-MB-231 cells treated with 30 μM of each compound **5a**–**s**. Values are expressed as percentage of untreated control (100%) ± standard deviation. Table S3: Dose-response effect of compounds **5a**–**e**, **5l** and **5p**–**r**, on cell viability in A2780 ovarian carcinoma and MDA-MB-231 triple negative breast carcinoma cancer cells. Cells were treated with varying concentrations of the appropriate compound (0.096 to 60 μM) for 72 h. The values represent the percent cell proliferation referred to the untreated control (set to 100%). Data represents the mean ± standard deviation (SD) from independent experiments.

## References

[1] Seeman J.I. (2025). Woodward and Hoffmann. Hoffmann and Woodward. A Close Collaboration Had Yet to Begin. Chem. Rec..

[2] Müller T.J.J. (2025). Recent Advances in Ene Reactions with Carbon Enophiles. Adv. Synth. Catal..

[3] Sankararaman S. (2005). Pericyclic Reactions—A Textbook: Reactions, Applications and Theory.

[4] Lallana E., Riguera R., Fernandez-Megia E. (2011). Reliable and Efficient Procedures for the Conjugation of Biomolecules through Huisgen Azide–Alkyne Cycloadditions. Angew. Chem. Int. Ed..

[5] Breugst M., Reissig H. (2020). The Huisgen Reaction: Milestones of the 1,3-Dipolar Cycloaddition. Angew. Chem. Int. Ed..

[6] Kasana S., Nigam V., Singh S., Kurmi B.D., Patel P. (2024). A New Insight into the Huisgen Reaction: Heterogeneous Copper Catalyzed Azide-Alkyne Cycloaddition for the Synthesis of 1,4-Disubstituted Triazole (From 2018–2023). Chem. Biodivers..

[7] Rostovtsev V.V., Green L.G., Fokin V.V., Sharpless K.B. (2002). A Stepwise Huisgen Cycloaddition Process: Copper(I)-Catalyzed Regioselective “Ligation” of Azides and Terminal Alkynes. Angew. Chem. Int. Ed..

[8] Moura N.M.M., Tomé A.C., Black D.S., Cossy J., Stevens C.V. (2022). 1,2,3-Triazoles. Comprehensive Heterocyclic Chemistry IV.

[9] Barlow T.M.A., Tourwé D., Ballet S. (2017). Cyclisation To Form Small, Medium and Large Rings by Use of Catalysed and Uncatalysed Azide–Alkyne Cycloadditions (AACs). Eur. J. Org. Chem..

[10] Gogoi P., Deori K., Sarma D. (2025). Regioselective Synthesis of 1,4-Disubstituted 1,2,3-Triazoles: Advances in Click Chemistry and Multicomponent Reaction Engineering. Synlett.

[11] Yadav M.S., Rajkhowa S., Singh S.K., Jaiswal M.K., Tiwari V.K. (2024). Multicomponent Click Reaction: An Indispensable Tool for Easy Access of Functionalized 1,2,3-Triazoles. ChemistrySelect.

[12] Tashrifi Z., Khanaposhtani M.M., Bahadorikhalili S., Larijani B., Mahdavi M. (2024). Intramolecular Click Cycloaddition Reactions: Synthesis of 1,2,3-Triazoles. Curr. Org. Synth..

[13] Nemallapudi B.R., Guda D.R., Ummadi N., Avula B., Zyryanov G.V., Reddy C.S., Gundala S. (2022). New Methods for Synthesis of 1,2,3-Triazoles: A Review. Polycycl. Aromat. Compd..

[14] Bhatia S., Biharee A., Mondal S., Kulkarni S., Kumar R., Thareja S. (2025). Mechanistic analysis of diverse click reactions along with their applications. Eur. J. Med. Chem..

[15] Jayashree S., Sheyara R.T.B., Anbarasu S., Samanta D. (2025). Copper-catalyzed and metal-free azide-alkyne ‘click’ reactions: From synthesis and functionalization of small molecules to macromolecules. J. Macromol. Sci. Part A.

[16] Dai J., Xue X., Jiang X., Liu X., Zhan P. (2025). Advances in click chemistry for drug discovery and development. Expert Opin. Drug Discov..

[17] Ragab S.S. (2025). Signature of click chemistry in advanced techniques for cancer therapeutics. RSC Adv..

[18] Nwe K., Brechbiel M.W. (2009). Growing Applications of “Click Chemistry” for Bioconjugation in Contemporary Biomedical Research. Cancer Biother. Radiopharm..

[19] Matin M.M., Matin P., Rahman M.R., Ben Hadda T., Almalki F.A., Mahmud S., Ghoneim M.M., Alruwaily M., Alshehri S. (2022). Triazoles and Their Derivatives: Chemistry, Synthesis, and Therapeutic Applications. Front. Mol. Biosci..

[20] Manoharan A., Jayan J., Rangarajan T.M., Bose K., Benny F., Ipe R.S., Kumar S., Kukreti N., Abdelgawad M.A., Ghoneim M.M. (2023). “Click Chemistry”: An Emerging Tool for Developing a New Class of Structural Motifs against Various Neurodegenerative Disorders. ACS Omega.

[21] Aly A.A., Hassan A.A., Mohamed N.K., Abd El-Aal A.S., Balboul B.A.A., Nayl A.A., Bräse S., Ramadan M. (2025). Synthesis, Reactions, and Biological Applications of 1,2,4-triazoles: A Review. Curr. Org. Chem..

[22] Serafini M., Pirali T., Tron G., Meanwell N., Lolli M. (2021). Click 1,2,3-triazoles in drug discovery and development: From the flask to the clinic?. Advances in Heterocyclic Chemistry.

[23] Forezi L.d.S.M., Cardoso M.F.C., Gonzaga D.T.G., da Silva F.d.C., Ferreira V.F. (2018). Alternative Routes to the Click Method for the Synthesis of 1,2,3-Triazoles, an Important Heterocycle in Medicinal Chemistry. Curr. Top. Med. Chem..

[24] Agalave S.G., Maujan S.R., Pore V.S. (2011). Click Chemistry: 1,2,3-Triazoles as Pharmacophores. Chem.—An Asian J..

[25] Bozorov K., Zhao J., Aisa H.A. (2019). 1,2,3-Triazole-containing hybrids as leads in medicinal chemistry: A recent overview. Bioorg. Med. Chem..

[26] Aouad M., Soliman M., Alharbi M., Bardaweel S., Sahu P., Ali A., Messali M., Rezki N., Al-Soud Y. (2018). Design, Synthesis and Anticancer Screening of Novel Benzothiazole-Piperazine-1,2,3-Triazole Hybrids. Molecules.

[27] Eddahmi M., Moura N.M.M., Bouissane L., Amiri O., Faustino M.A.F., Cavaleiro J.A.S., Mendes R.F., Paz F.A.A., Neves M.G.P.M.S., Rakib E.M. (2019). A Suitable Functionalization of Nitroindazoles with Triazolyl and Pyrazolyl Moieties via Cycloaddition Reactions. Molecules.

[28] Cao Y., Yang Y., Ampomah-Wireko M., Obaid Arhema Frejat F., Zhai H., Zhang S., Wang H., Yang P., Yuan Q., Wu G. (2022). Novel indazole skeleton derivatives containing 1,2,3-triazole as potential anti-prostate cancer drugs. Bioorg. Med. Chem. Lett..

[29] Wu B.-W., Huang W.-J., Liu Y.-H., Liu Q.-G., Song J., Hu T., Chen P., Zhang S.-Y. (2024). Design, synthesis and biological evaluation of 1,2,3-triazole benzothiazole derivatives as tubulin polymerization inhibitors with potent anti-esophageal cancer activities. Eur. J. Med. Chem..

[30] Aljuhani A., Nafie M.S., Albujuq N.R., Alsehli M., Bardaweel S.K., Darwish K.M., Alraqa S.Y., Aouad M.R., Rezki N. (2025). Discovery of new benzothiazole-1,2,3-triazole hybrid-based hydrazone/thiosemicarbazone derivatives as potent EGFR inhibitors with cytotoxicity against cancer. RSC Adv..

[31] Mohamed-Ezzat R.A., Elgemeie G.H. (2025). Synthetic strategies towards benzothiazole-based compounds of therapeutic potency: Experimental, investigational and approved drugs. RSC Adv..

[32] Padilla-Martínez I.I., Cruz A., García-Báez E.V., Mendieta-Wejebe J.E., Rosales-Hernández M.C. (2025). Condensation Reactions of 2-Aminothiophenoles to Afford 2-Substituted Benzothiazoles of Biological Interest: A Review (2020–2024). Int. J. Mol. Sci..

[33] Asif M., Imran M. (2021). A Mini-Review on Pharmacological Importance of Benzothiazole Scaffold. Mini-Rev. Org. Chem..

[34] Kale A., Kakde R., Pawar S., Thombare R. (2021). Recent Development in Substituted Benzothiazole as an Anticonvulsant Agent. Mini-Rev. Med. Chem..

[35] Agarwal S., Gandhi D., Kalal P. (2017). Benzothiazole: A Versatile and Multitargeted Pharmacophore in the Field of Medicinal Chemistry. Lett. Org. Chem..

[36] Rouf A., Tanyeli C. (2015). Bioactive thiazole and benzothiazole derivatives. Eur. J. Med. Chem..

[37] Keri R.S., Patil M.R., Patil S.A., Budagumpi S. (2015). A comprehensive review in current developments of benzothiazole-based molecules in medicinal chemistry. Eur. J. Med. Chem..

[38] Doraghi F., Edareh M.H., Ghanbarlou M., Larijani B., Mahdavi M. (2025). Unconventional approaches for synthesis of 2-substituted benzothiazoles. Org. Biomol. Chem..

[39] Palchykov V.A. (2025). Visible light-mediated synthesis of 1,3-benzothiazoles: A comprehensive review. Coord. Chem. Rev..

[40] Zaghary W.A., Elgemeie G.H., Azzam R.A., Aljuhr S.A., Sakr T.M. (2025). Recent Developments in the Synthesis of Benzothiazoles and their Anti-cancer Mechanistic Discoveries. Curr. Pharm. Des..

[41] Kashyap M., Gupta S., Bansal Y., Bansal G. (2024). A critical analysis of design, binding pattern and SAR of benzo-fused heteronuclear compounds as VEGFR-2 inhibitors. Bioorg. Med. Chem..

[42] Aayishamma I., Purawarga Matada G.S., Pal R., Ghara A., Aishwarya N.V.S.S., Kumaraswamy B., Hosamani K.R., Manjushree B.V., Haripriya E. (2024). Benzothiazole a privileged scaffold for Cutting-Edges anticancer agents: Exploring drug design, structure-activity relationship, and docking studies. Eur. J. Med. Chem..

[43] Paoletti N., Supuran C.T. (2024). Benzothiazole derivatives in the design of antitumor agents. Arch. Pharm..

[44] Yadav K.P., Rahman M.A., Nishad S., Maurya S.K., Anas M., Mujahid M. (2023). Synthesis and biological activities of benzothiazole derivatives: A review. Intell. Pharm..

[45] Haroun M. (2022). Review on the Developments of Benzothiazole-containing Antimicrobial Agents. Curr. Top. Med. Chem..

[46] Puri S., Sawant S., Juvale K. (2023). A comprehensive review on the indazole based derivatives as targeted anticancer agents. J. Mol. Struct..

[47] Shang C., Hou Y., Meng T., Shi M., Cui G. (2021). The Anticancer Activity of Indazole Compounds: A Mini Review. Curr. Top. Med. Chem..

[48] Tandon N., Luxami V., Kant D., Tandon R., Paul K. (2021). Current progress, challenges and future prospects of indazoles as protein kinase inhibitors for the treatment of cancer. RSC Adv..

[49] Barresi E., Baglini E., Poggetti V., Castagnoli J., Giorgini D., Salerno S., Taliani S., Da Settimo F. (2024). Indole-Based Compounds in the Development of Anti-Neurodegenerative Agents. Molecules.

[50] Pal D., Sahu P. (2022). Importance of Indazole against Neurological Disorders. Curr. Top. Med. Chem..

[51] Er-raqioui R., Roudani S., El Houssni I., Gumede N.J., Sert Y., Mendes R.F., Chernyshov D., Paz F.A.A., Cavaleiro J.A.S., Faustino M.d.A.F. (2025). Synthesis, Characterization, Antimicrobial Activity and Molecular Modeling Studies of Novel Indazole-Benzimidazole Hybrids. Antibiotics.

[52] Paul A., Guria T., Roy P., Maity A. (2022). Recent Development of Heterocyclic Compounds with Indazole Moiety as Potential Antiparasitic Agents. Curr. Top. Med. Chem..

[53] Gandham S.K., Kudale A.A., Allaka T.R., Chepuri K., Jha A. (2024). New indazole–1,2,3–triazoles as potent antimicrobial agents: Design, synthesis, molecular modeling and in silico ADME profiles. J. Mol. Struct..

[54] Nanda S.S., Yi D.K., Panda O.P., Chigurupati S., Mohapatra T.K., Hossain M.I. (2022). Impact of Indazole Scaffold as Antibacterial and Antifungal Agent. Curr. Top. Med. Chem..

[55] Parveen H., Wani M.Y., Mukhtar S., Ahmad A. (2024). Synthesis, in vitro and in silico screening of novel ferrocenyl substituted cyclohexenone and indazole derivatives as effective antimycobacterial agents. J. Mol. Struct..

[56] Taha M., Gilani S.J., Kazmi I., Rahim F., Adalat B., Ullah H., Nawaz F., Wadood A., Ali Z., Shah S.A.A. (2024). Synthesis, biological evaluation and molecular docking study of indazole based schiff base analogues as new anti-diabetic inhibitors. J. Mol. Struct..

[57] Rodríguez-Villar K., Yépez-Mulia L., Cortés-Gines M., Aguilera-Perdomo J.D., Quintana-Salazar E.A., Olascoaga Del Angel K.S., Cortés-Benítez F., Palacios-Espinosa J.F., Soria-Arteche O., Pérez-Villanueva J. (2021). Synthesis, Antiprotozoal Activity, and Cheminformatic Analysis of 2-Phenyl-2H-Indazole Derivatives. Molecules.

[58] Kumar V., Sirbaiya A.K., Nematullah M., Haider M.F., Rahman M.A. (2024). Synthesis of 1,3-substituted 1H-indazole derivatives and evaluation of anti-inflammatory activity in Sprague Dawley rats. Intell. Pharm..

[59] He M., Wang J., Deng W., Han X., Wang X., Pang L., Huang J., Lan P., Wang T., Wang Z. (2024). Exploring novel indazole derivatives as ASK1 inhibitors: Design, synthesis, biological evaluation and docking studies. Bioorg. Chem..

[60] Laghchioua F.E., Kouakou A., Eddahmi M., Viale M., Monticone M., Gangemi R., Maric I., El Ammari L., Saadi M., Baltas M. (2020). Antiproliferative and apoptotic activity of new indazole derivatives as potential anticancer agents. Arch. Pharm..

[61] Eddahmi M., Moura N.M.M., Bouissane L., Faustino M.A.F., Cavaleiro J.A.S., Paz F.A.A., Mendes R.F., Figueiredo J., Carvalho J., Cruz C. (2019). Synthesis and Biological Evaluation of New Functionalized Nitroindazolylacetonitrile Derivatives. ChemistrySelect.

[62] Noelting E. (1904). Ueber Bildung von Indazolen aus nitrirten orthomethylirten Aminen. Berichte Dtsch. Chem. Ges..

[63] Abbassi N., Rakib E.M., Bouissane L., Hannioui A., Khouili M., El Malki A., Benchidmi M., Essassi E.M. (2011). Studies on the Reduction of the Nitro Group in 4-Nitroindazoles by Anhydrous SnCl 2 in Different Alcohols. Synth. Commun..

[64] Chevalier A., Ouahrouch A., Arnaud A., Gallavardin T., Franck X. (2018). An optimized procedure for direct access to 1 H -indazole-3-carboxaldehyde derivatives by nitrosation of indoles. RSC Adv..

[65] Laghchioua F.E., da Silva C.F.M., Pinto D.C.G.A., Cavaleiro J.A.S., Mendes R.F., Paz F.A.A., Faustino M.A.F., Rakib E.M., Neves M.G.P.M.S., Pereira F. (2024). Design of Promising Thiazoloindazole-Based Acetylcholinesterase Inhibitors Guided by Molecular Docking and Experimental Insights. ACS Chem. Neurosci..

[66] Laghchioua F.E., Gomes A.T.P.C., Mendes K., Mendes R.F., Paz F.A.A., Videira P.A., Faustino M.A.F., Cavaleiro J.A.S., Rakib E.M., Neves M.G.P.M.S. (2026). Benzothiazole–Triphenylphosphonium Conjugates: Synthesis, In Silico Analysis, and Exploratory In Vitro Effects on HT-29 Colorectal Cancer Cell Viability. ChemMedChem.

[67] Salih O.M., Al-Sha’er M.A., Basheer H.A. (2024). Novel 2-Aminobenzothiazole Derivatives: Docking, Synthesis, and Biological Evaluation as Anticancer Agents. ACS Omega.

[68] Javahershenas R., Han J., Kazemi M., Jervis P.J. (2024). Recent Advances in the Application of 2-Aminobenzothiazole to the Multicomponent Synthesis of Heterocycles. ChemistryOpen.

[69] Daina A., Michielin O., Zoete V. (2017). SwissADME: A free web tool to evaluate pharmacokinetics, drug-likeness and medicinal chemistry friendliness of small molecules. Sci. Rep..

[70] Daina A., Michielin O., Zoete V. (2014). iLOGP: A Simple, Robust, and Efficient Description of n -Octanol/Water Partition Coefficient for Drug Design Using the GB/SA Approach. J. Chem. Inf. Model..

[71] Ghini V., Magherini F., Massai L., Messori L., Turano P. (2022). Comparative NMR metabolomics of the responses of A2780 human ovarian cancer cells to clinically established Pt-based drugs. Dalt. Trans..

[72] Ray R., Al Khashali H., Haddad B., Wareham J., Coleman K.-L., Alomari D., Ranzenberger R., Guthrie J., Heyl D., Evans H.G. (2022). Regulation of Cisplatin Resistance in Lung Cancer Cells by Nicotine, BDNF, and a β-Adrenergic Receptor Blocker. Int. J. Mol. Sci..

[73] Maciel L.L.F., Silva M.B., Moreira R.O., Cardoso A.P., Fernandes C., Horn A., de Aquino Almeida J.C., Kanashiro M.M. (2022). In Vitro and In Vivo Relevant Antineoplastic Activity of Platinum(II) Complexes toward Triple-Negative MDA-MB-231 Breast Cancer Cell Line. Pharmaceutics.

[74] Ben-David U., Siranosian B., Ha G., Tang H., Oren Y., Hinohara K., Strathdee C.A., Dempster J., Lyons N.J., Burns R. (2018). Genetic and transcriptional evolution alters cancer cell line drug response. Nature.

[75] Ogasawara S., Mihara Y., Kondo R., Kusano H., Akiba J.H. (2019). Yano Antiproliferative Effect of Lenvatinib on Human Liver Cancer Cell Lines In Vitro and In Vivo. Anticancer Res..

[76] Armarego W.L.F., Chai C. (2013). Purification of Laboratory Chemicals.

[77] Amaro A., Angelini G., Mirisola V., Esposito A.I., Reverberi D., Matis S., Maffei M., Giaretti W., Viale M., Gangemi R. (2016). A highly invasive subpopulation of MDA-MB-231 breast cancer cells shows accelerated growth, differential chemoresistance, features of apocrine tumors and reduced tumorigenicity in vivo. Oncotarget.

[78] Hussain R.F., Nouri A.M.E., Oliver R.T.D. (1993). A new approach for measurement of cytotoxicity using colorimetric assay. J. Immunol. Methods.

