# Supplementary material for: Benzothiazole–Triazole–Indazole Conjugates: Design, Characterization, and Preliminary In Vitro MTT-Based Antiproliferative Evaluation
Source: Molecules. 2026 Sep 28;31(19):3452. doi: 10.3390/molecules31193452 (PMC13666027; doi:10.3390/molecules31193452)
Supplement: Supplementary file 1 [file molecules-31-03452-s001.zip › molecules-4485538-supplementary.pdf]

Supporting Information

Benzothiazole–Triazole–Indazole Conjugates: Design, Characterization, and Preliminry *In Vitro* MTT-Based Antiproliferative Evaluation

Fatima Ezzahra Laghchioua,[1] Maurizio Viale,[2] Renata Silva,[3] Fernando Remião,[3] José A. S. Cavaleiro,[4] M. Amparo F. Faustino,[4] El Mostapha Rakib,[1,5,*] Maria da Graça P. M. S. Neves,[4] Nuno M. M. Moura[4,*]

[1] Laboratory of Molecular Chemistry, Materials and Catalysis, Faculty of Sciences and Technics, Sultan Moulay Slimane University, Beni-Mellal, BP 523, Morocco

[2] U.O. Bioterapie, IRCCS Azienda Ospedaliera Metropolitana (IRCCS AOM), L.go R. Benzi, 10, 16132 Genova, Italy

[3] UCIBIO-Applied Molecular Biosciences Unit, Laboratory of Toxicology, Department of Biological Sciences, Faculty of Pharmacy, University of Porto, Rua Jorge Viterbo Ferreira, 228, 4050-313 Porto, Portugal

[4] LAQV-REQUIMTE, Department of Chemistry, University of Aveiro, 3810-193 Aveiro, Portugal

[5] Higher School of Technology, Sultan Moulay Slimane University, BP 336, Fkih Ben Salah, Morocco

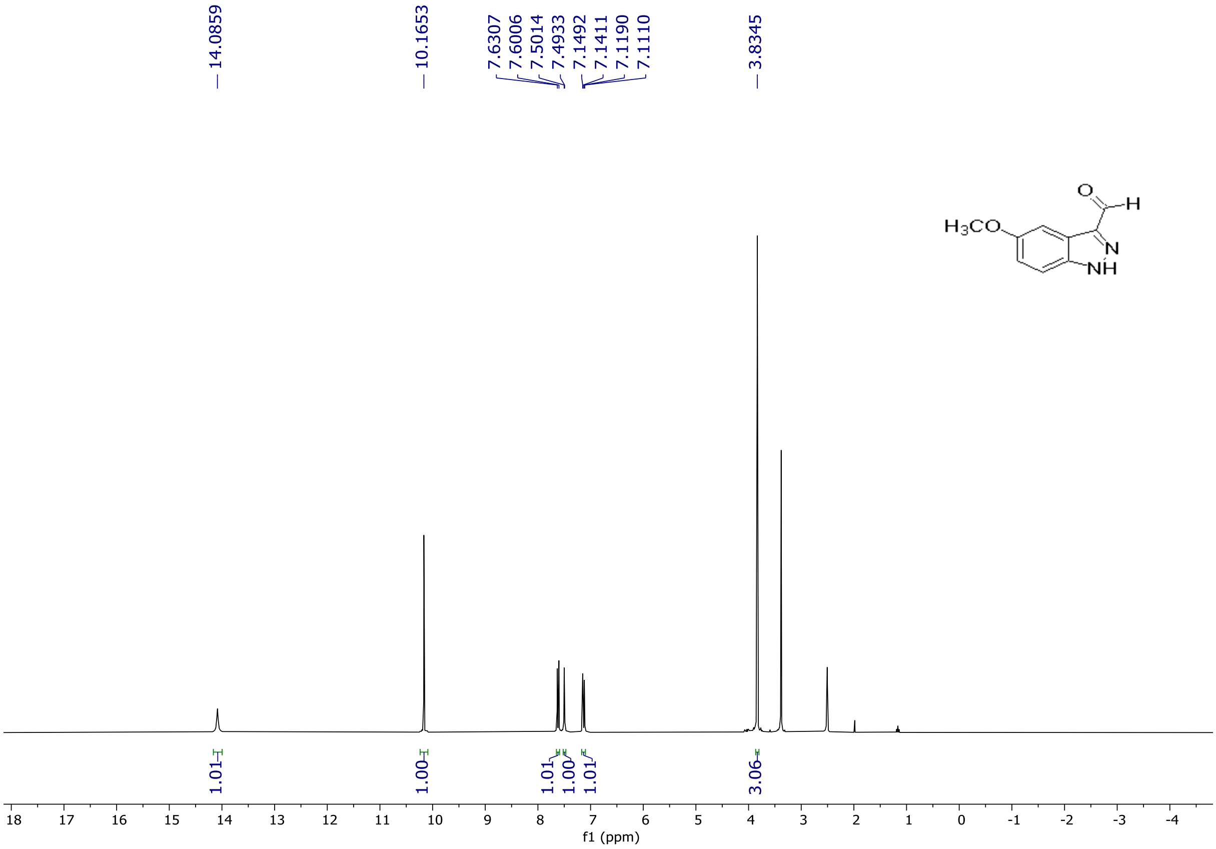

Figure S1. ^{1}H NMR spectrum of compound **1d** (300.13 MHz, DMSO-d_6).

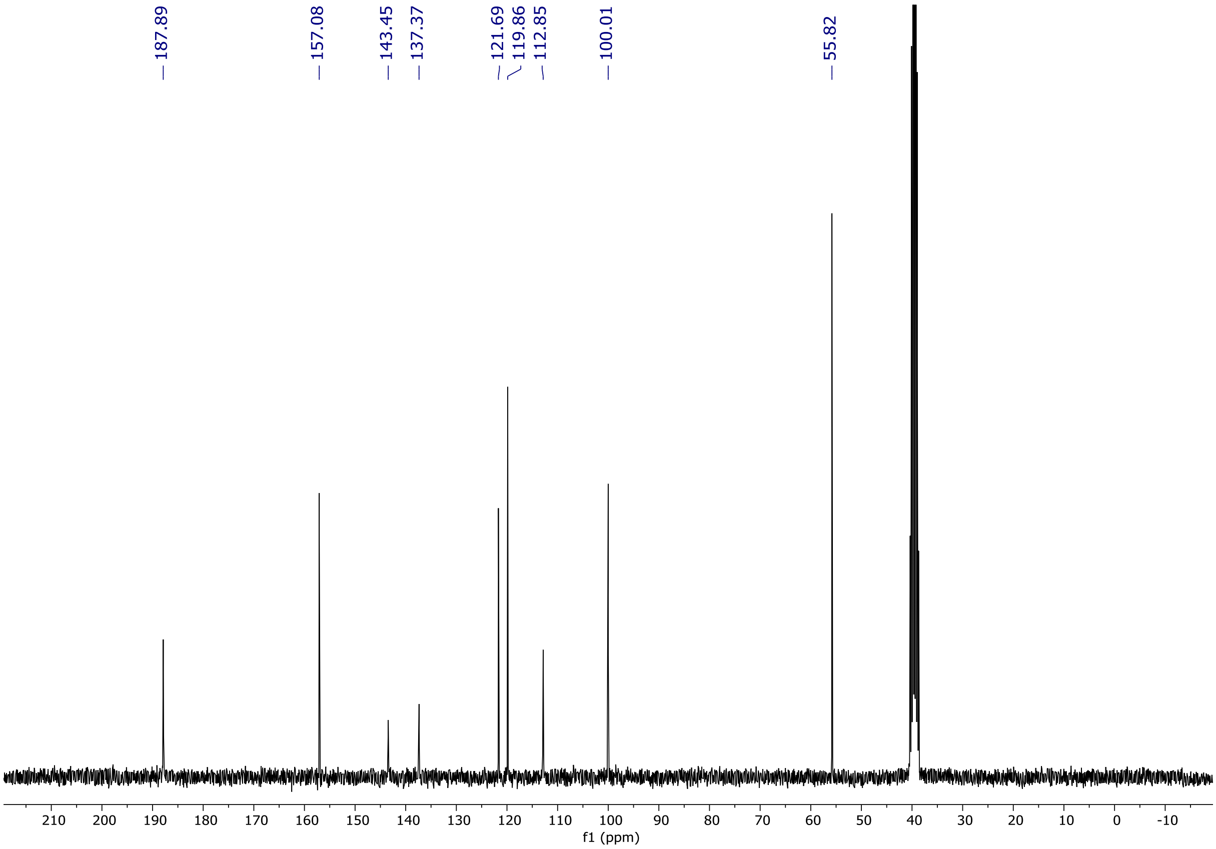

Figure S2. ^{13}C NMR spectrum of compound **1d** (75.47 MHz, DMSO-d_6).

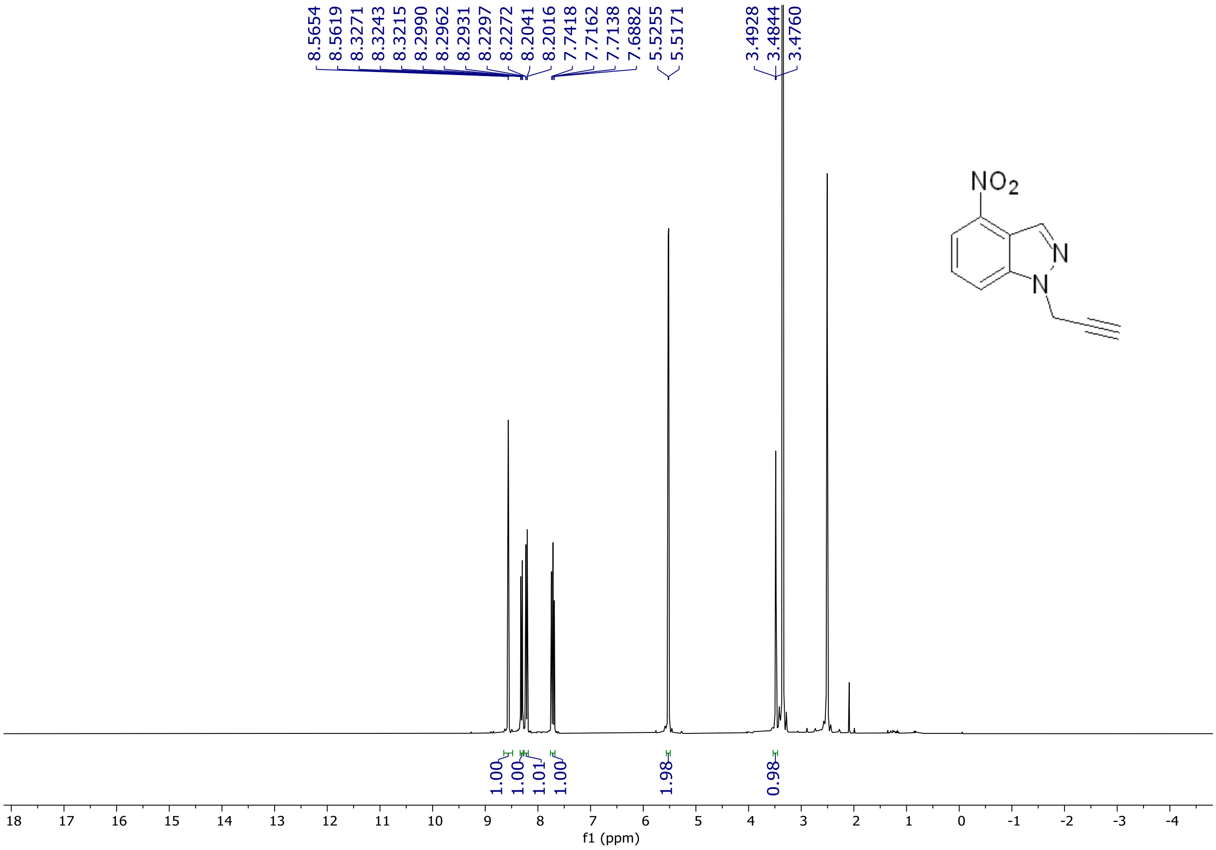

Figure S3. ^{1}H NMR spectrum of compound **2a** (300.13 MHz, DMSO-d_6).

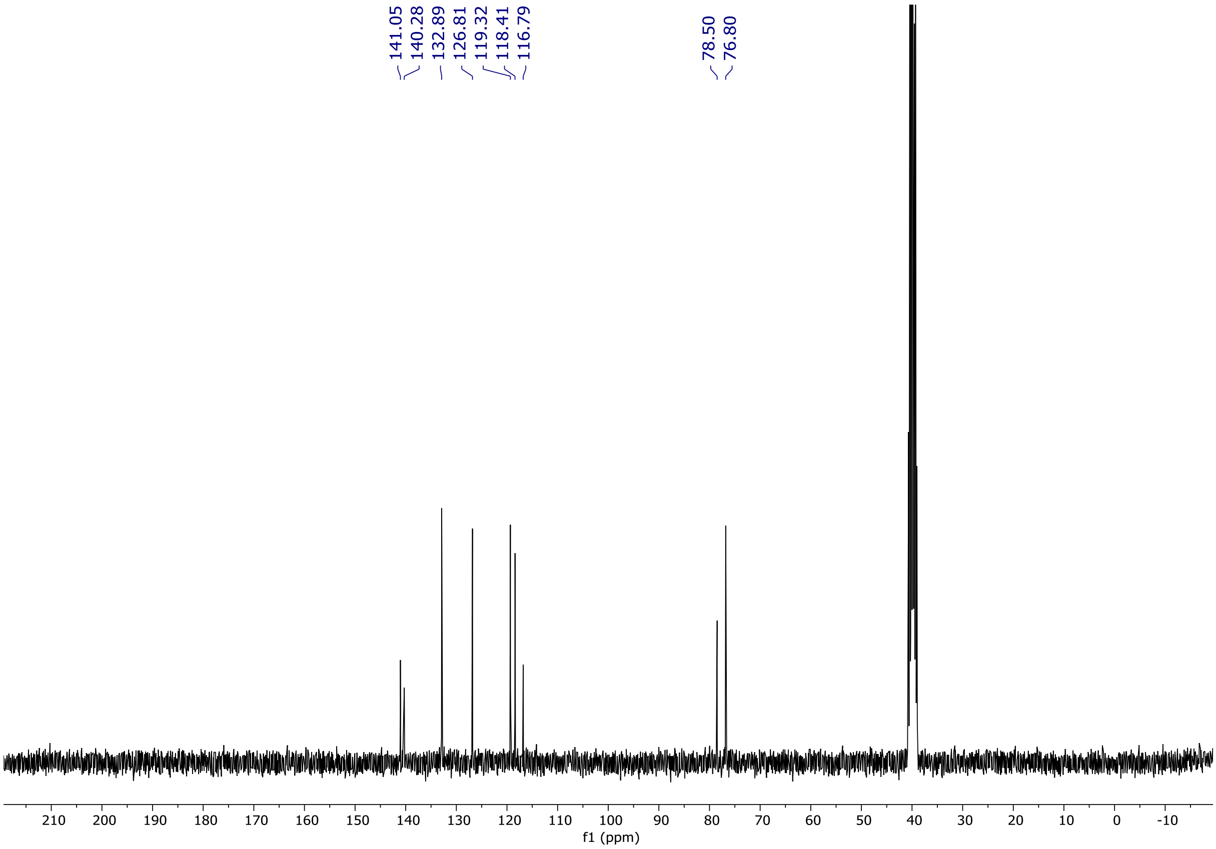

Figure S4. ^{13}C NMR spectrum of compound **2a** (75.47 MHz, DMSO-d_6).

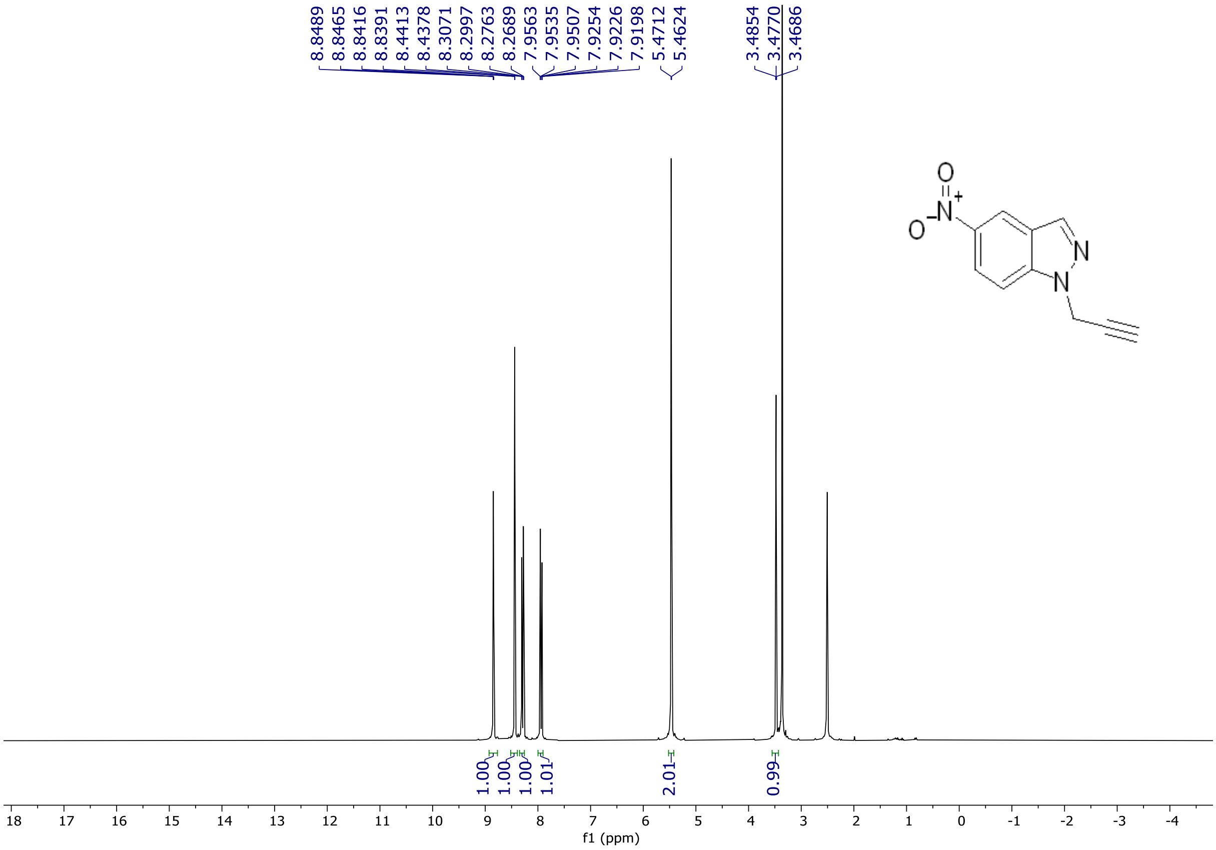

Figure S5. ^{1}H NMR spectrum of compound **2b** (300.13 MHz, DMSO-d_6).

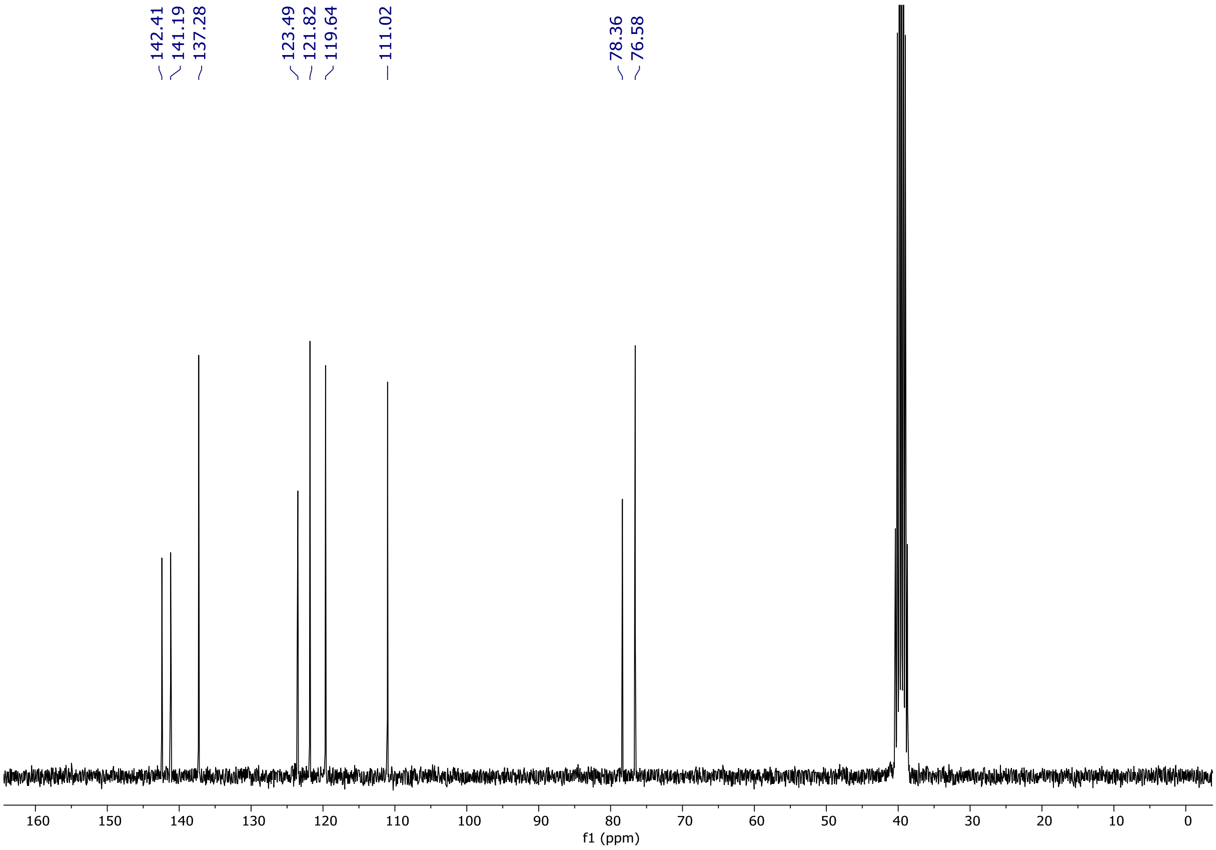

Figure S6. ^{13}C NMR spectrum of compound **2b** (75.47 MHz, DMSO-d_6).

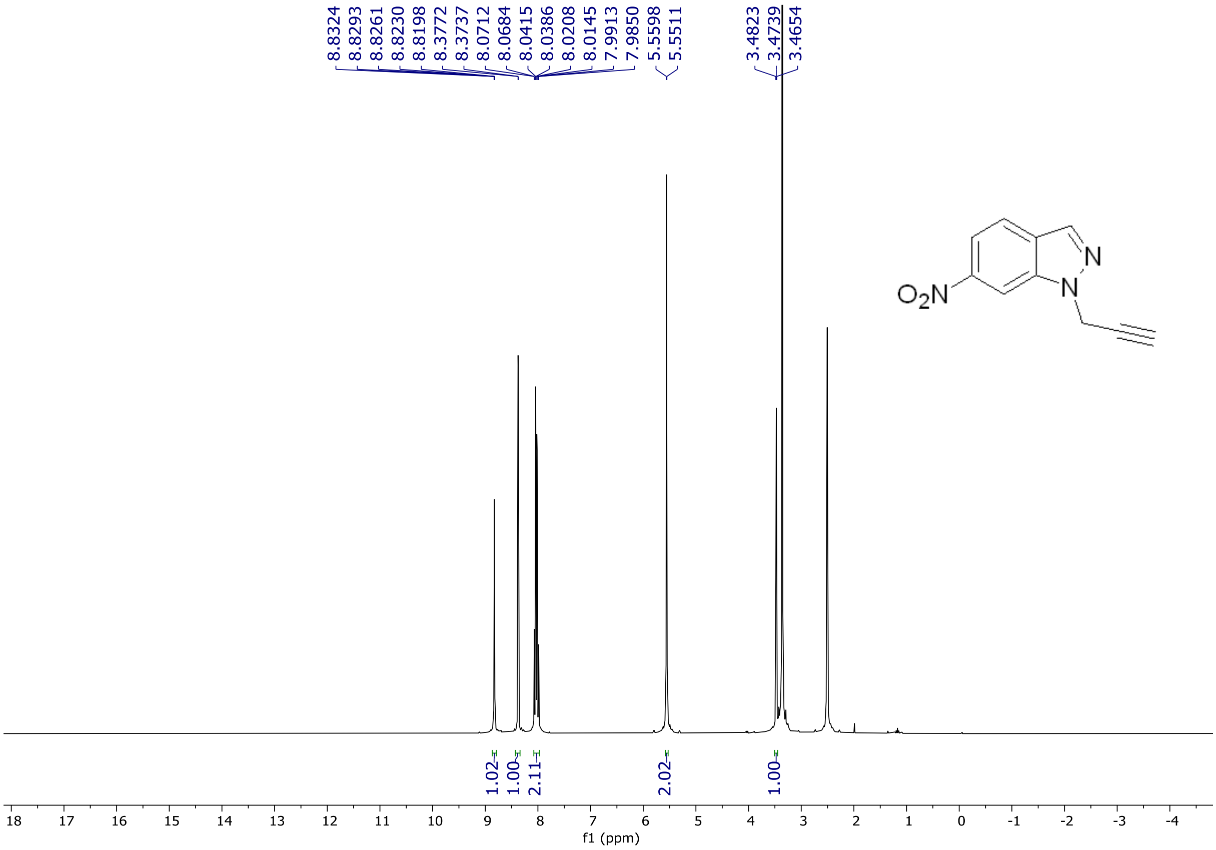

Figure S7. ^{1}H NMR spectrum of compound **2c** (300.13 MHz, DMSO-d_6).

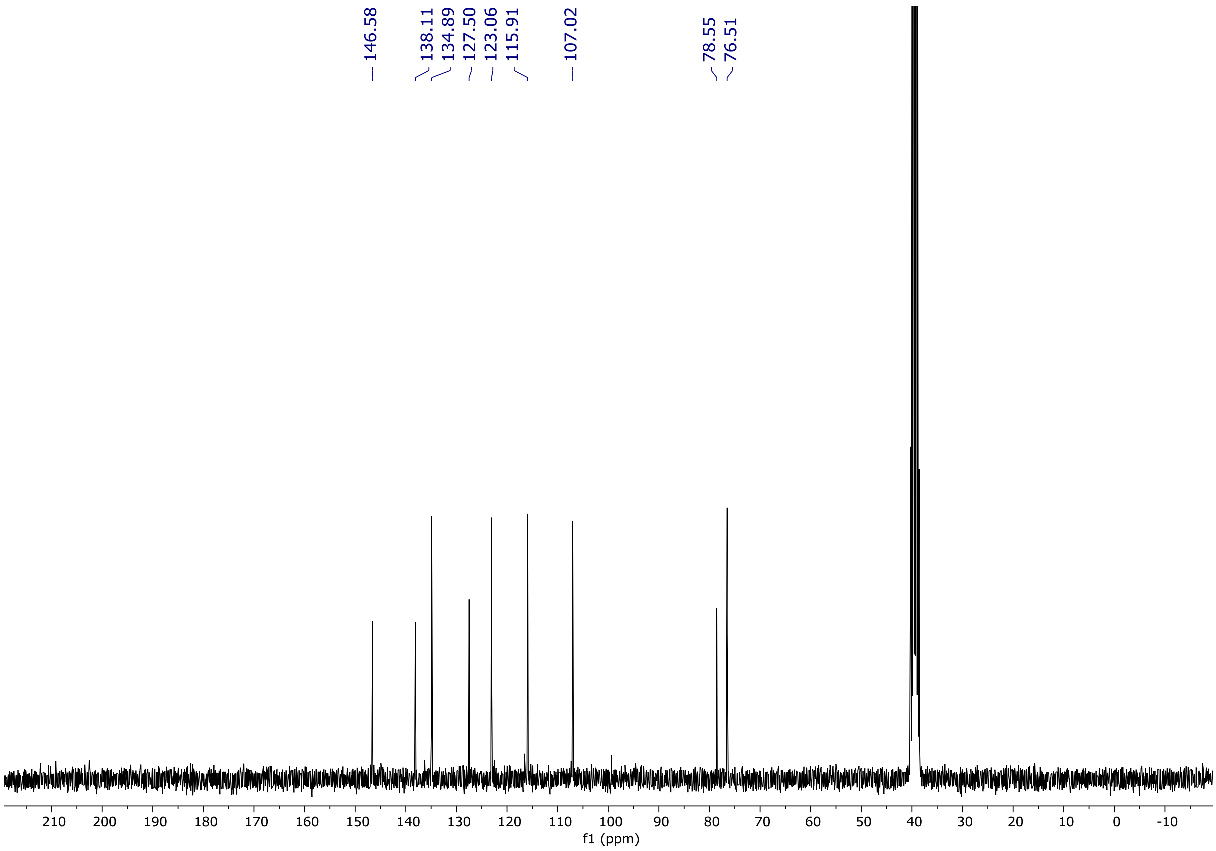

Figure S8. ^{13}C NMR spectrum of compound **2c** (75.47 MHz, DMSO-d_6).

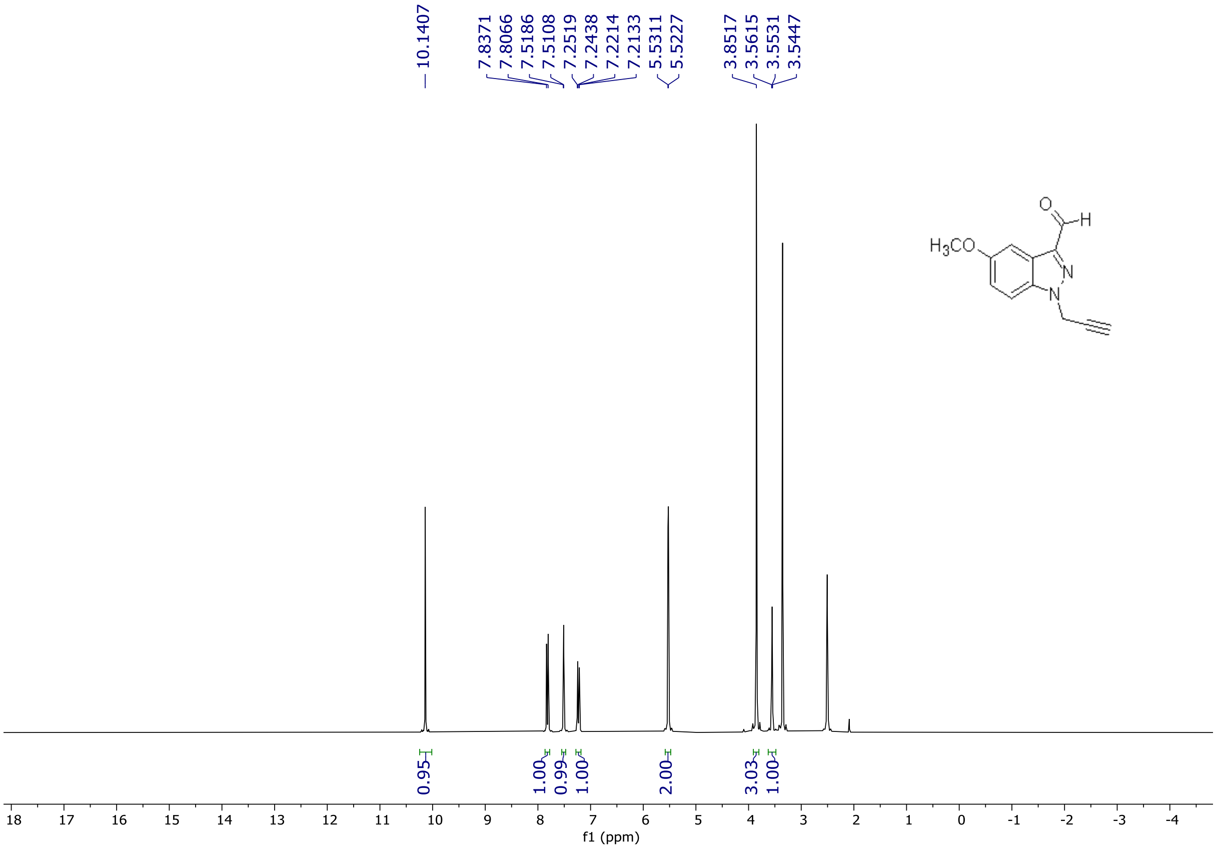

Figure S9. ^{1}H NMR spectrum of compound 2**d** (300.13 MHz, DMSO-d_6).

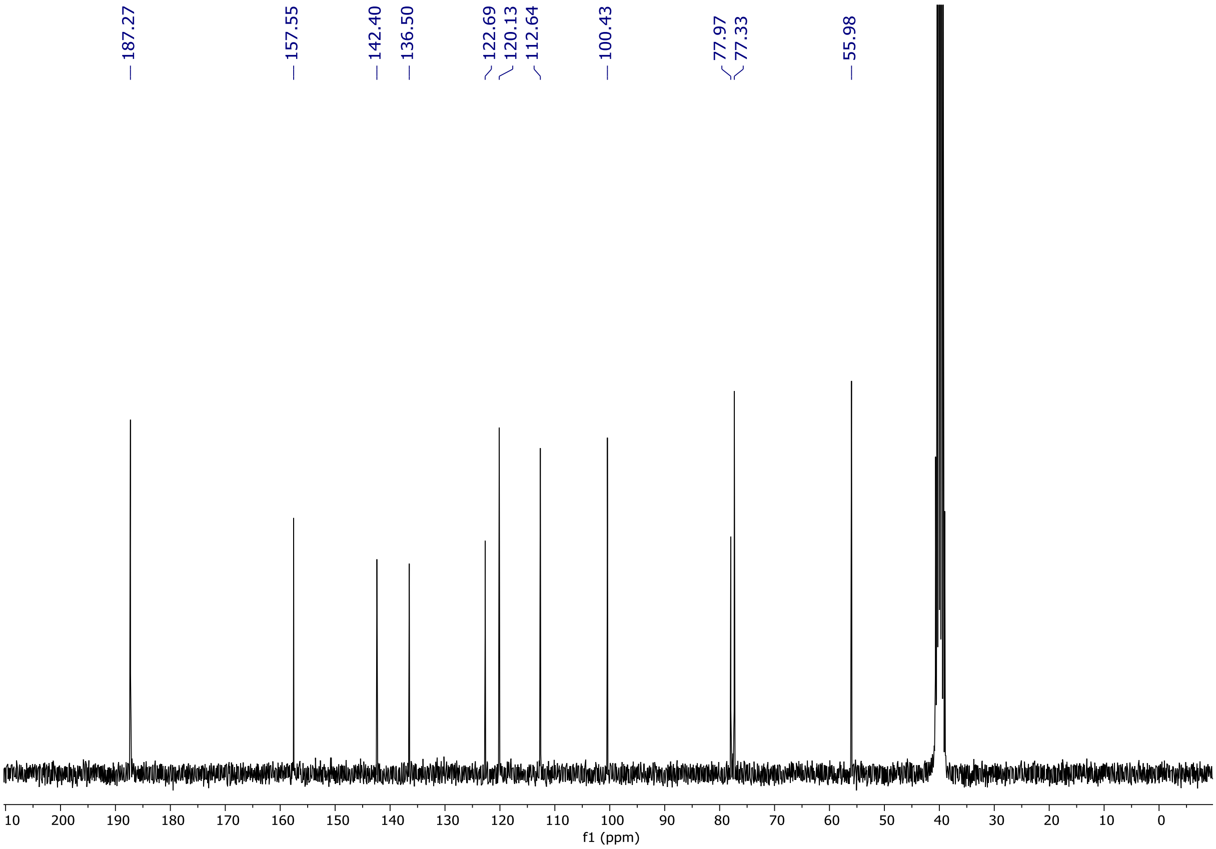

Figure S10. ^{13}C NMR spectrum of compound **2d** (75.47 MHz, DMSO-d_6).

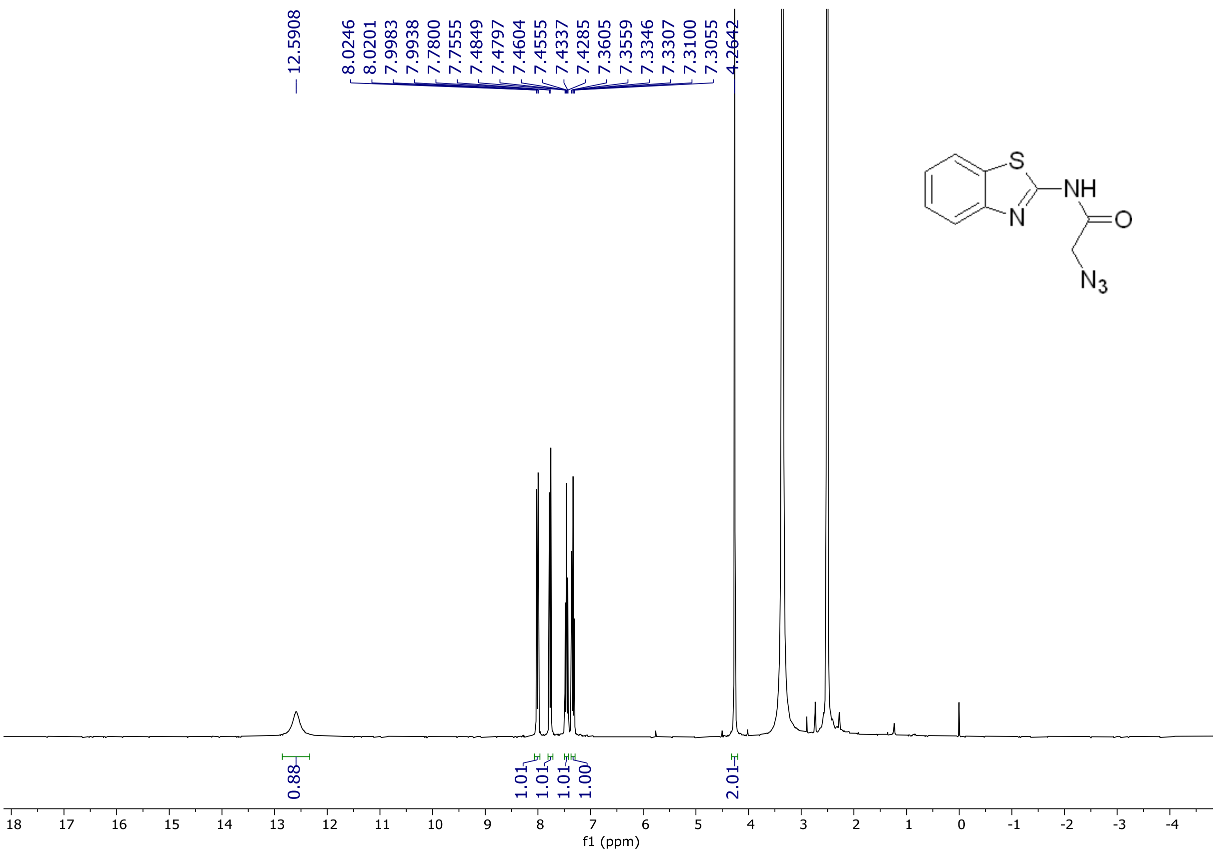

Figure S11. ^{1}H NMR spectrum of compound **4a** (300.13 MHz, DMSO-*d*$_6$).

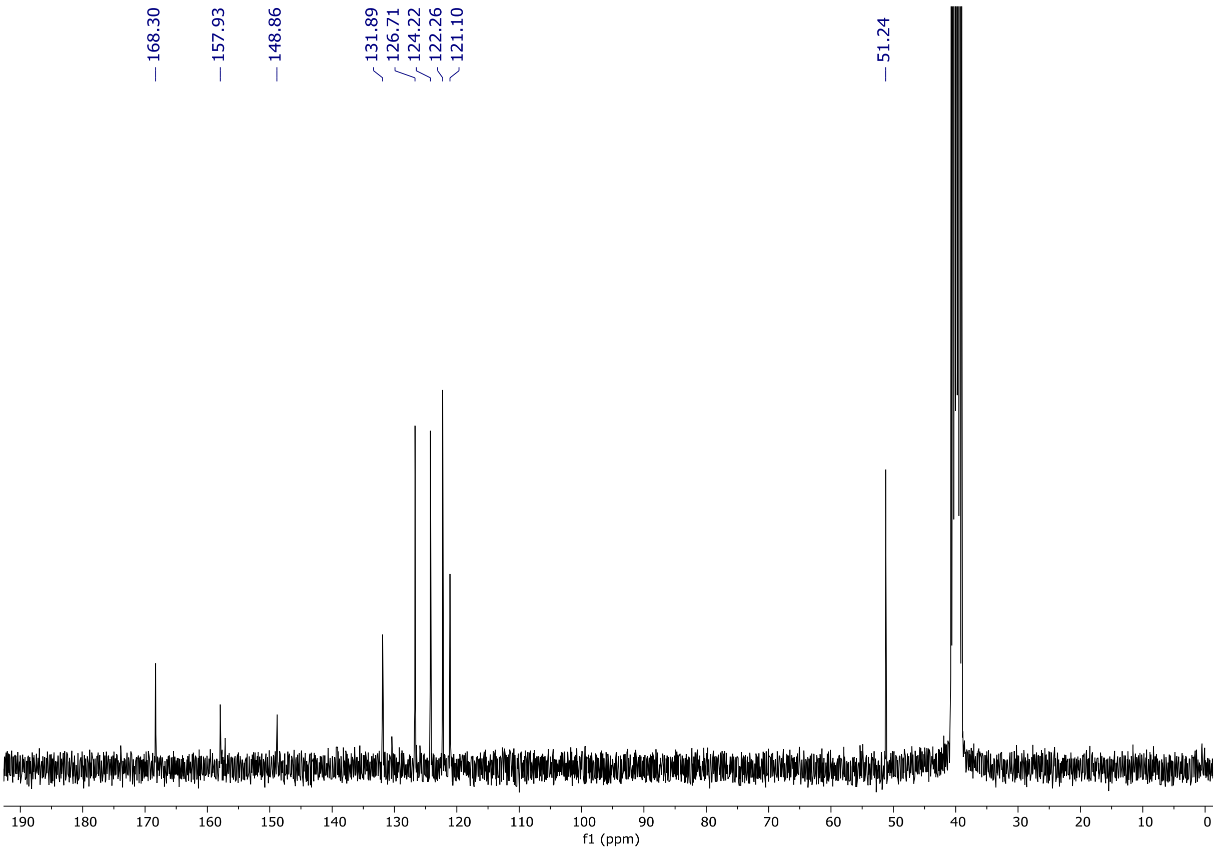

Figure S12. ^{13}C NMR spectrum of compound **4a** (75.47 MHz, DMSO-*d*$_6$).

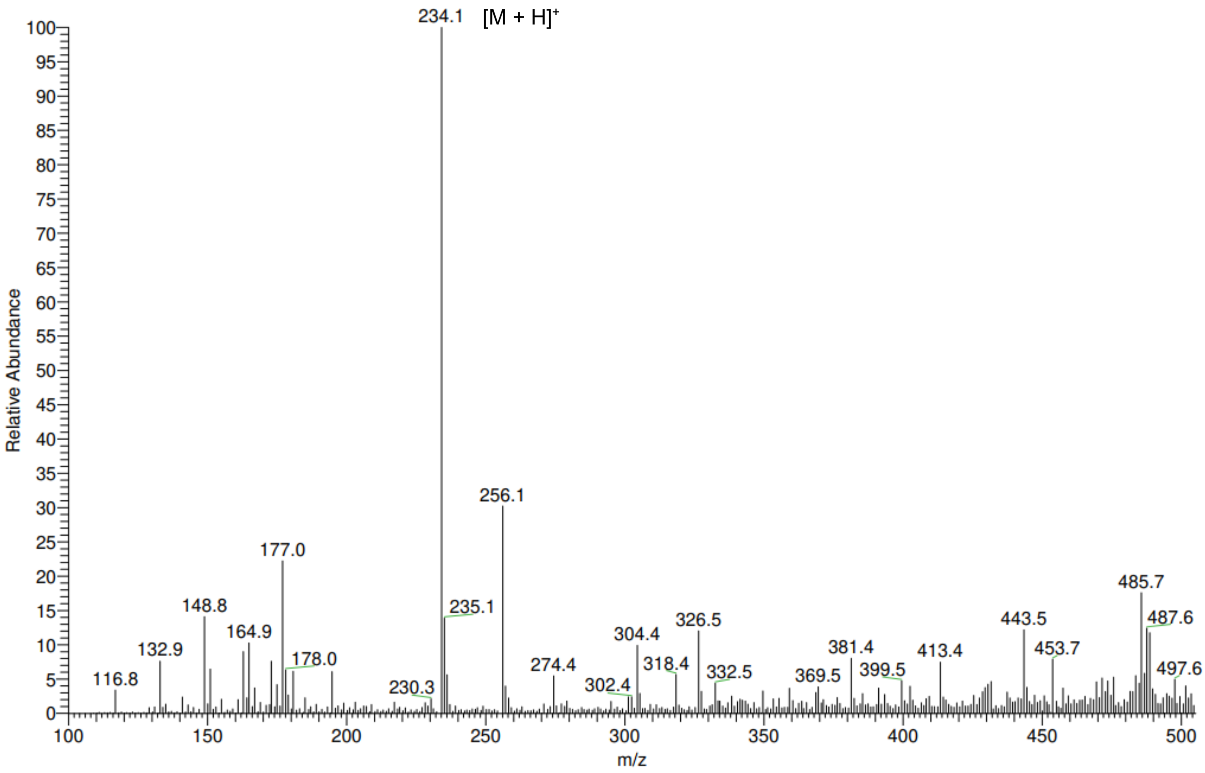

Figure S13. MS-ESI(+) spectrum of compound **4a**.

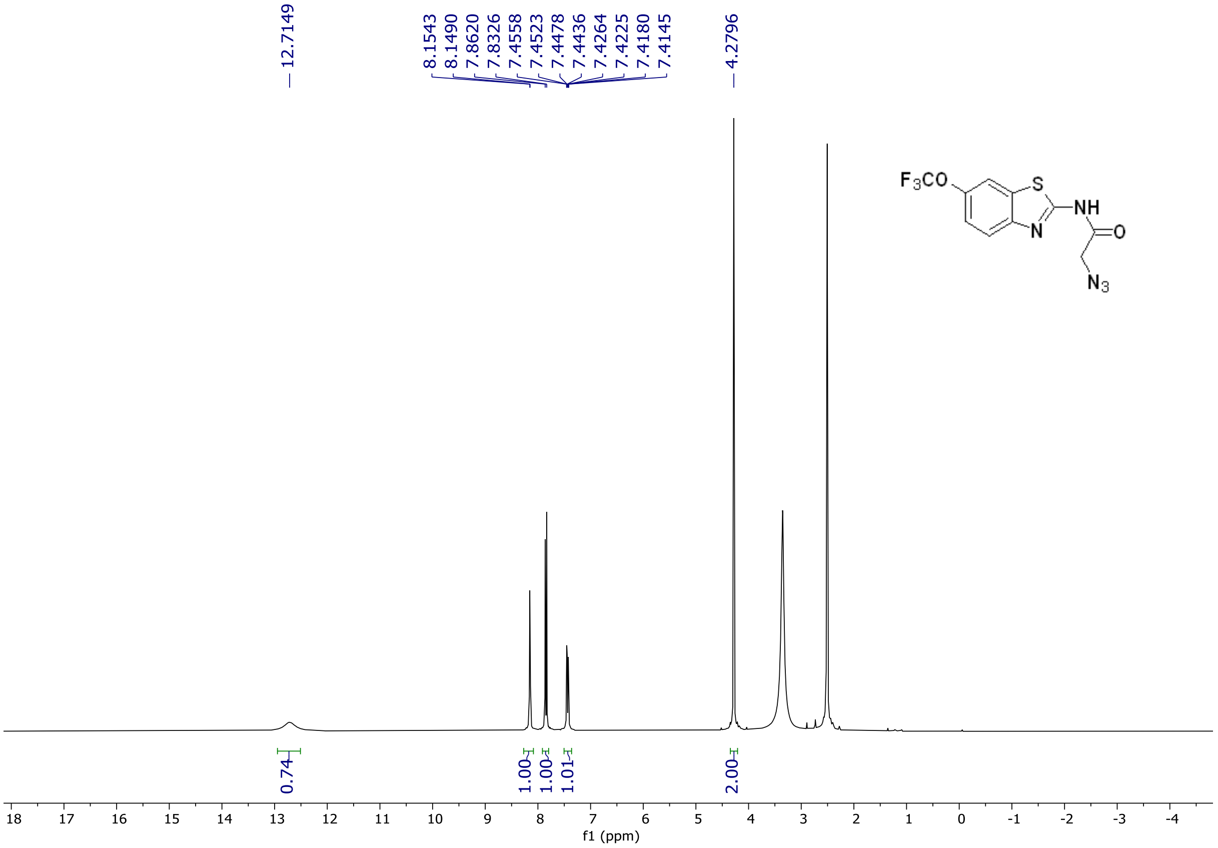

Figure S14. ^{1}H NMR spectrum of compound **4b** (300.13 MHz, DMSO-d_6).

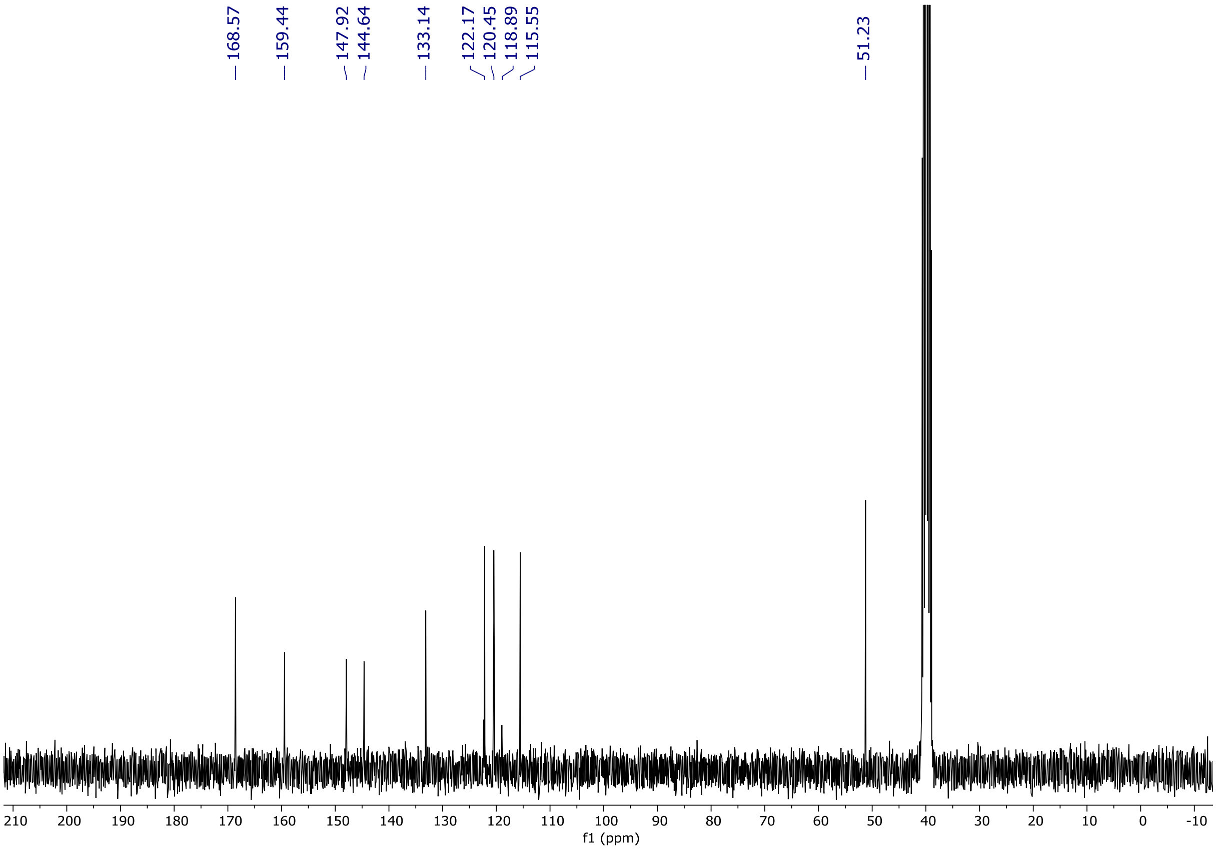

Figure S15. ^{13}C NMR spectrum of compound **4b** (75.47 MHz, DMSO-d_6).

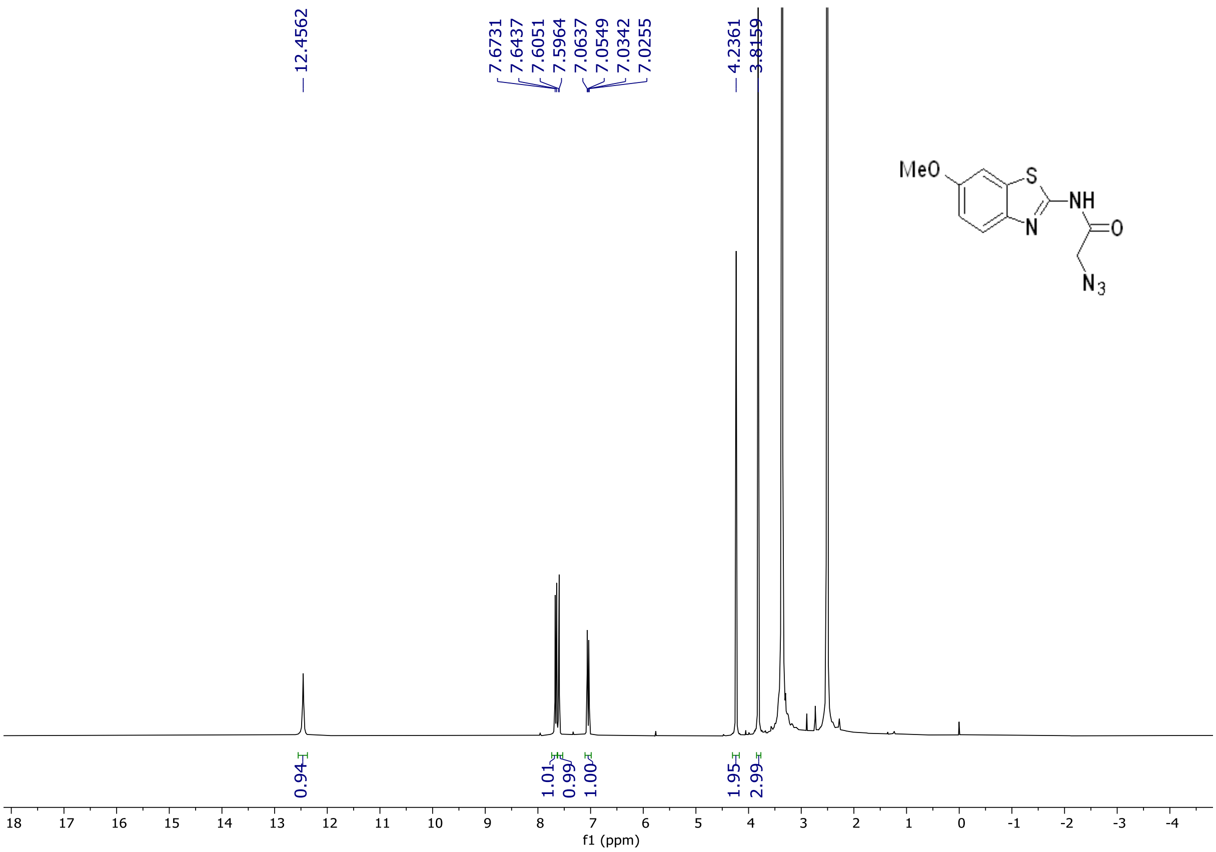

Figure S16. ^{1}H NMR spectrum of compound **4c** (300.13 MHz, DMSO-d_6).

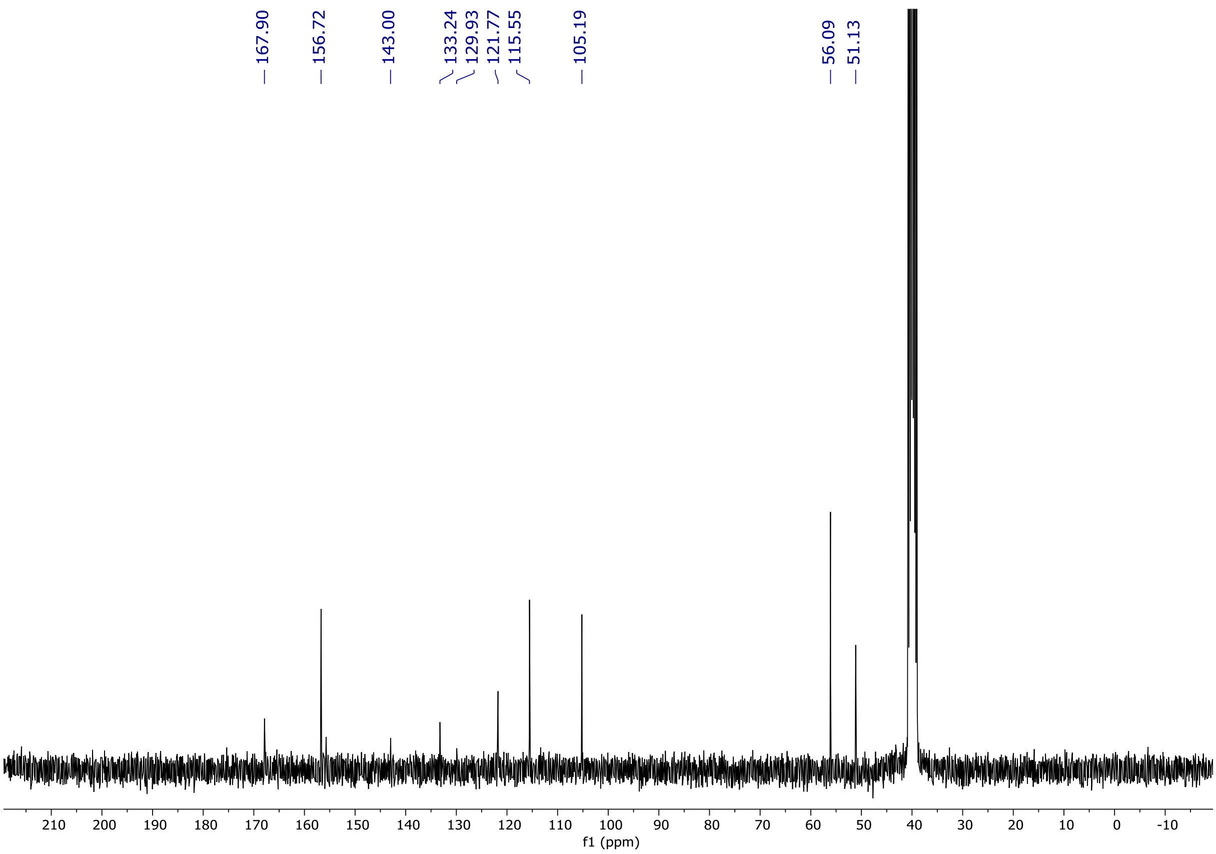

Figure S17. ^{13}C NMR spectrum of compound **4c** (75.47 MHz, DMSO-d_6).

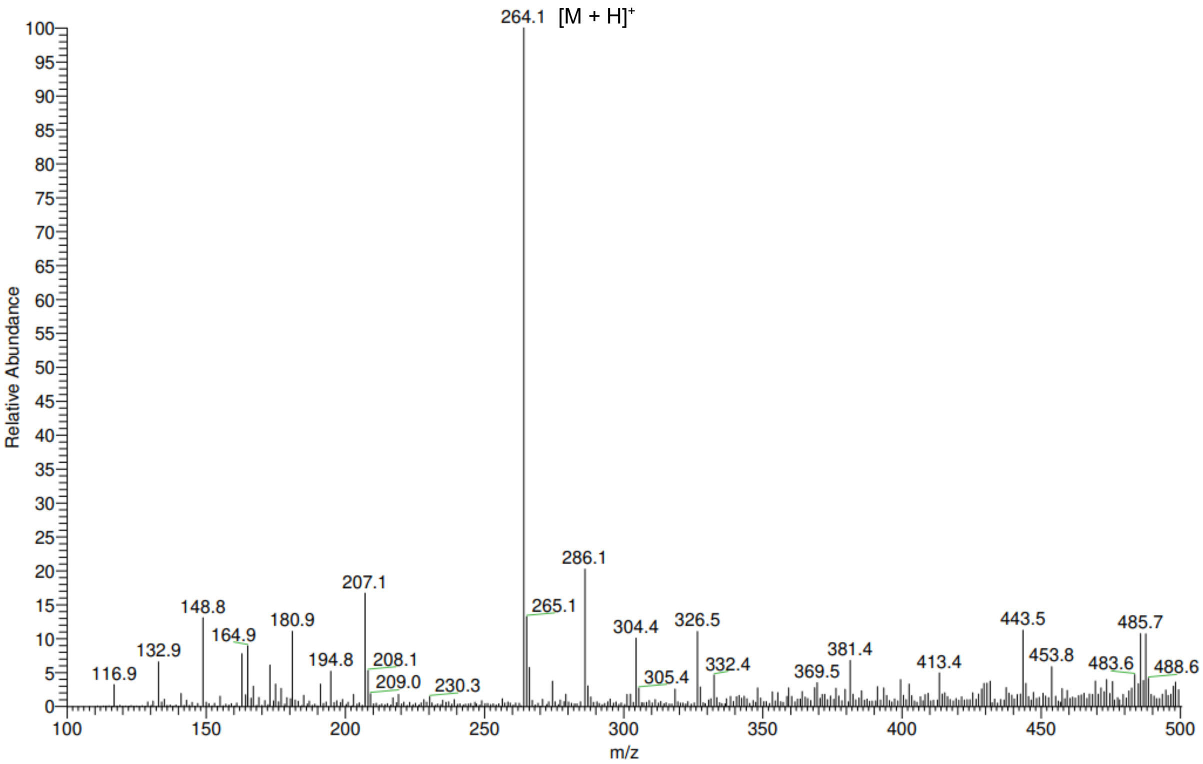

Figure S18. MS-ESI(+) spectrum of compound **4c**.

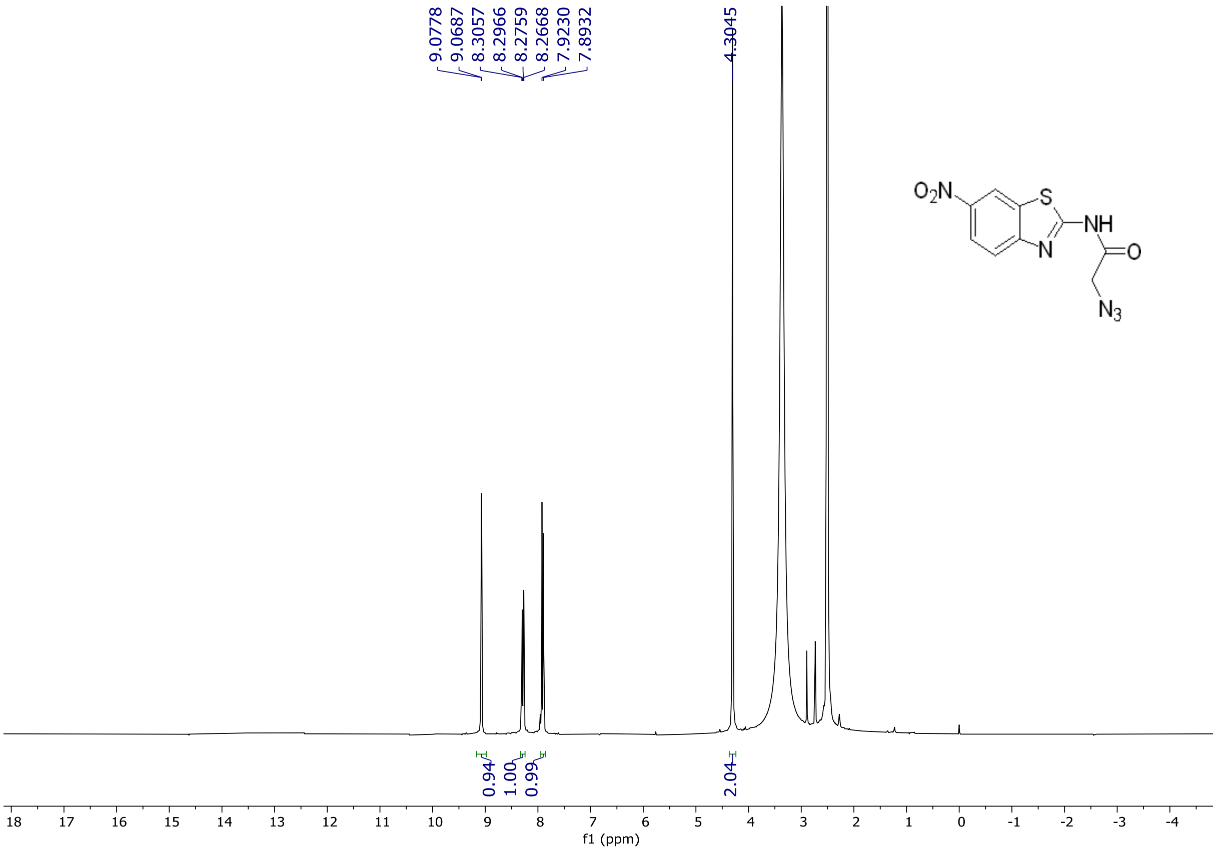

Figure S19. ^{1}H NMR spectrum of compound **4d** (300.13 MHz, DMSO-*d*$_6$).

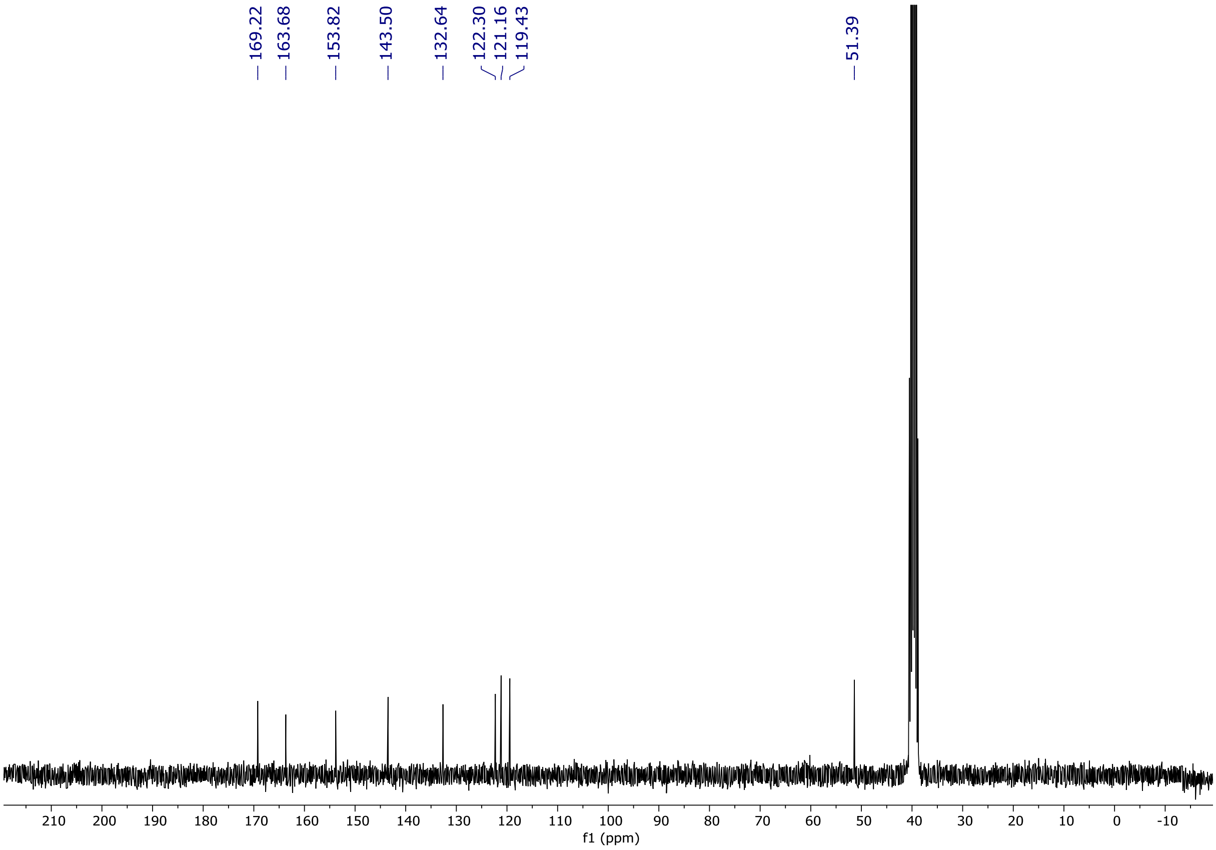

Figure S20. ^{13}C NMR spectrum of compound **4d** (75.47 MHz, DMSO-*d*$_6$).

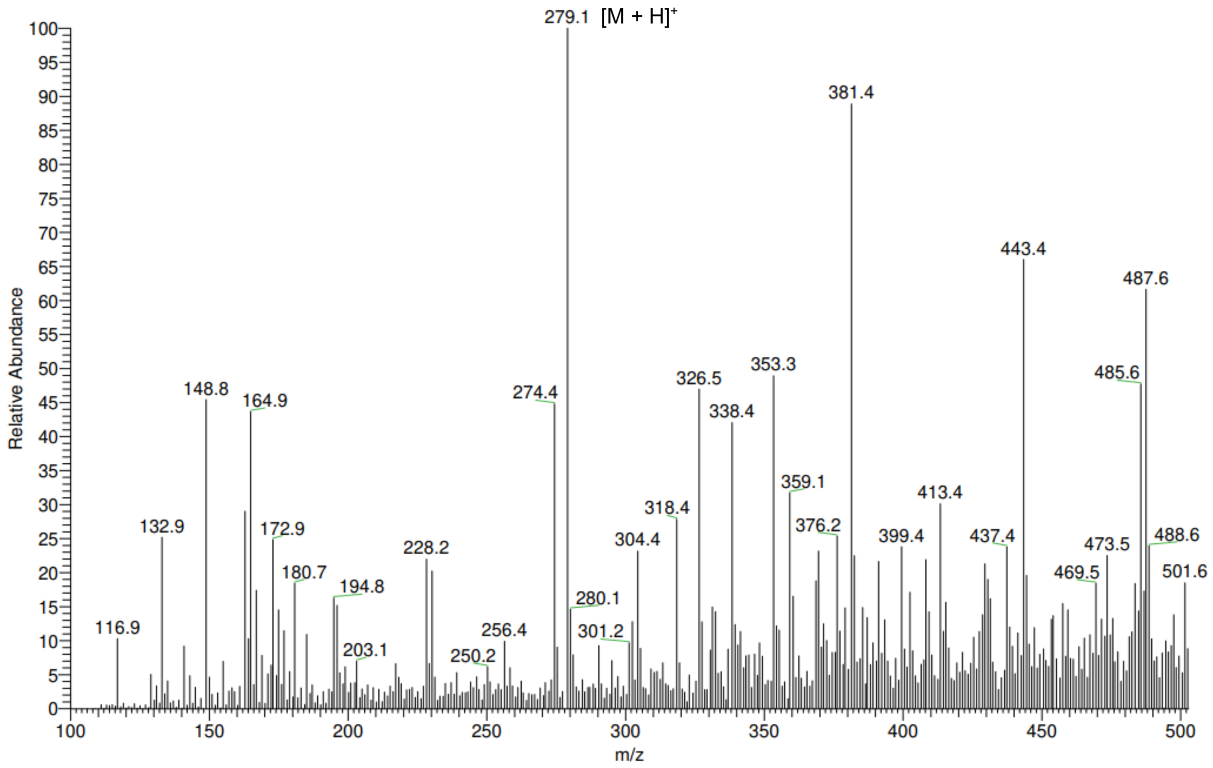

Figure S21. MS-ESI(+) spectrum of compound **4d**.

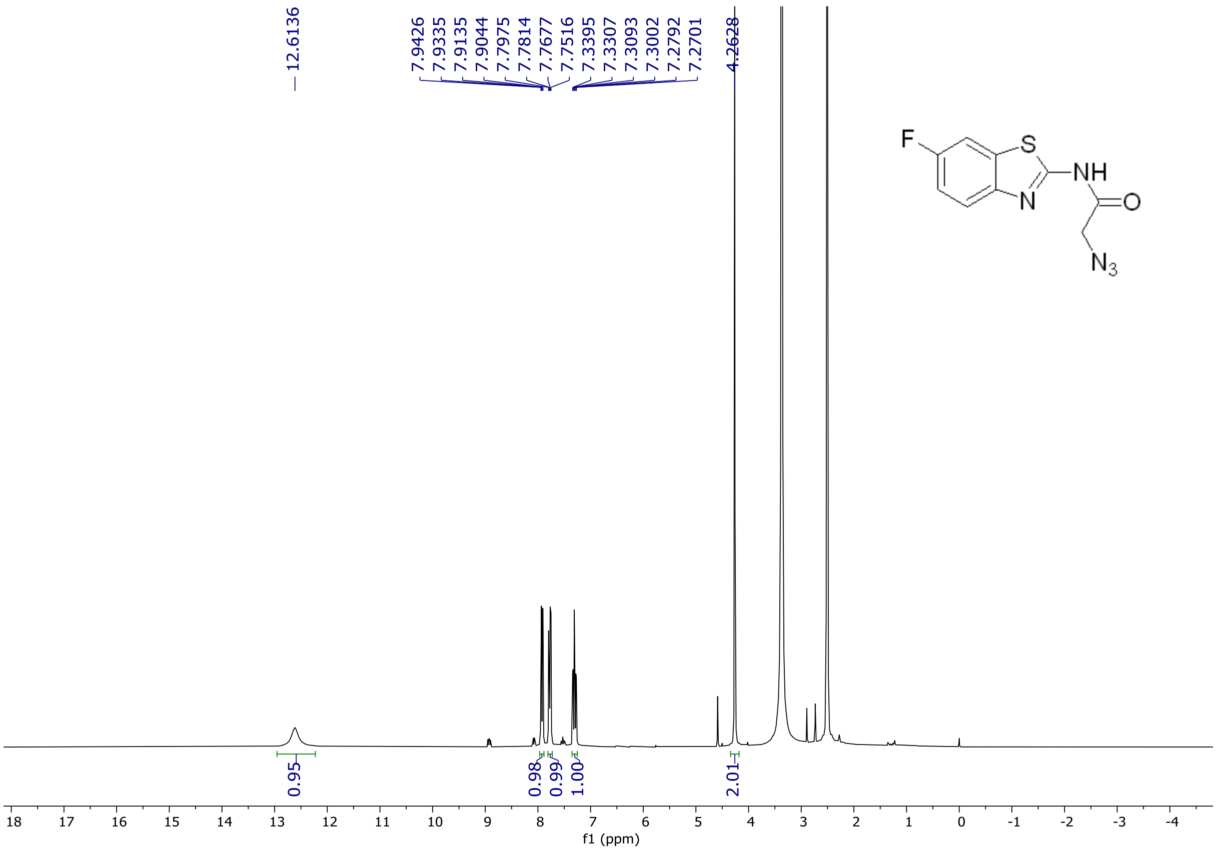

Figure S22. ^{1}H NMR spectrum of compound **4e** (300.13 MHz, DMSO-d_6).

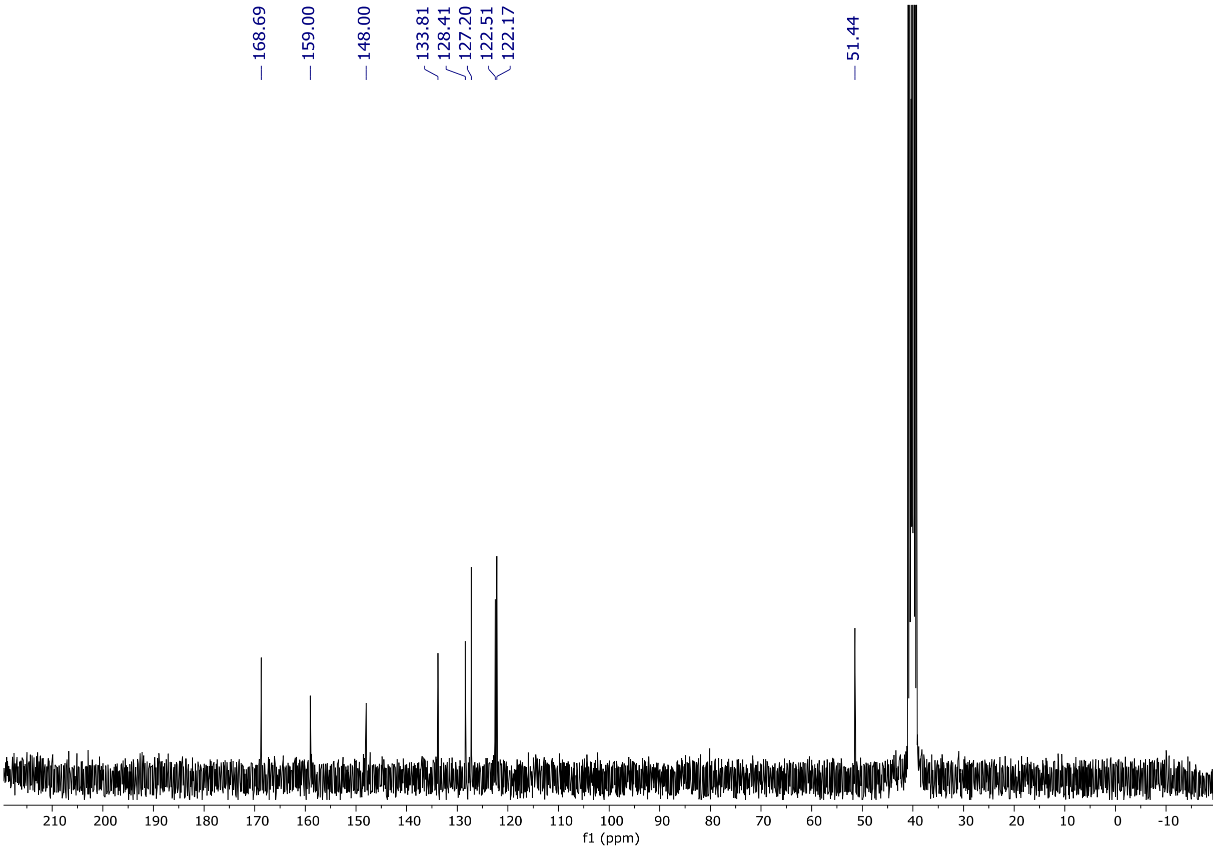

Figure S23. ^{13}C NMR spectrum of compound **4e** (75.47 MHz, DMSO-d_6).

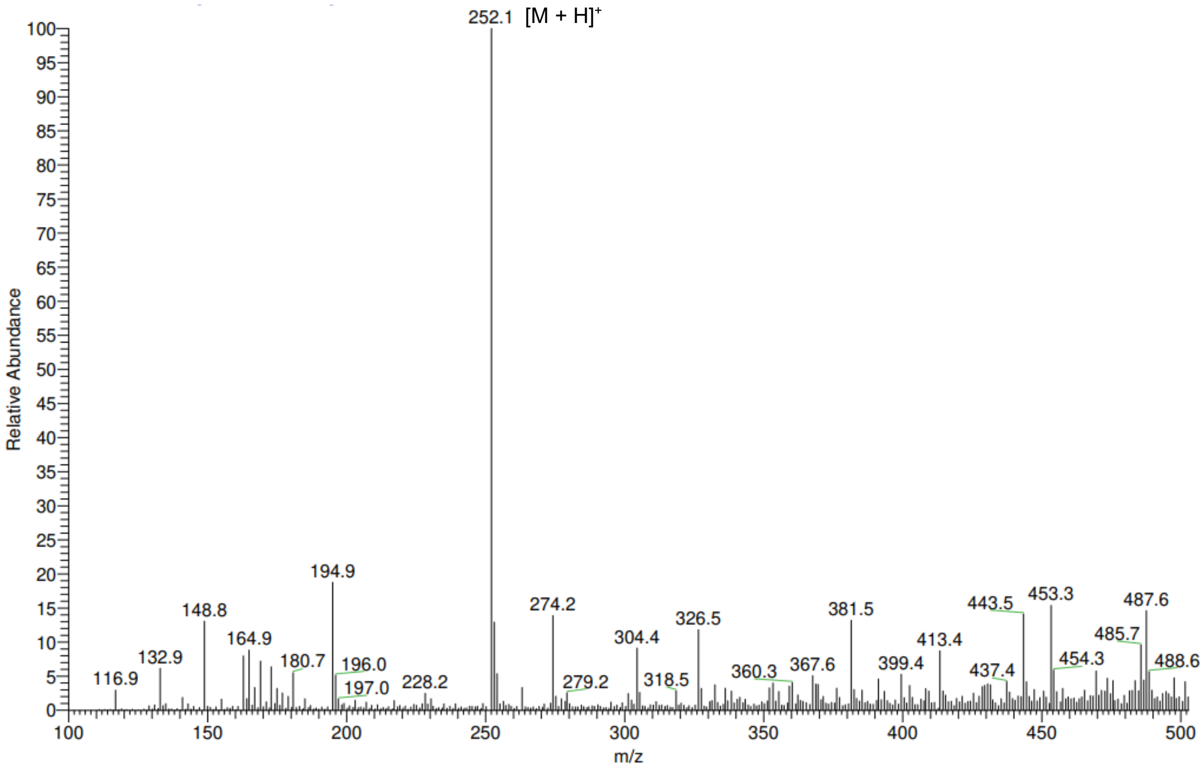

Figure S24. MS-ESI(+) spectrum of compound **4e**.

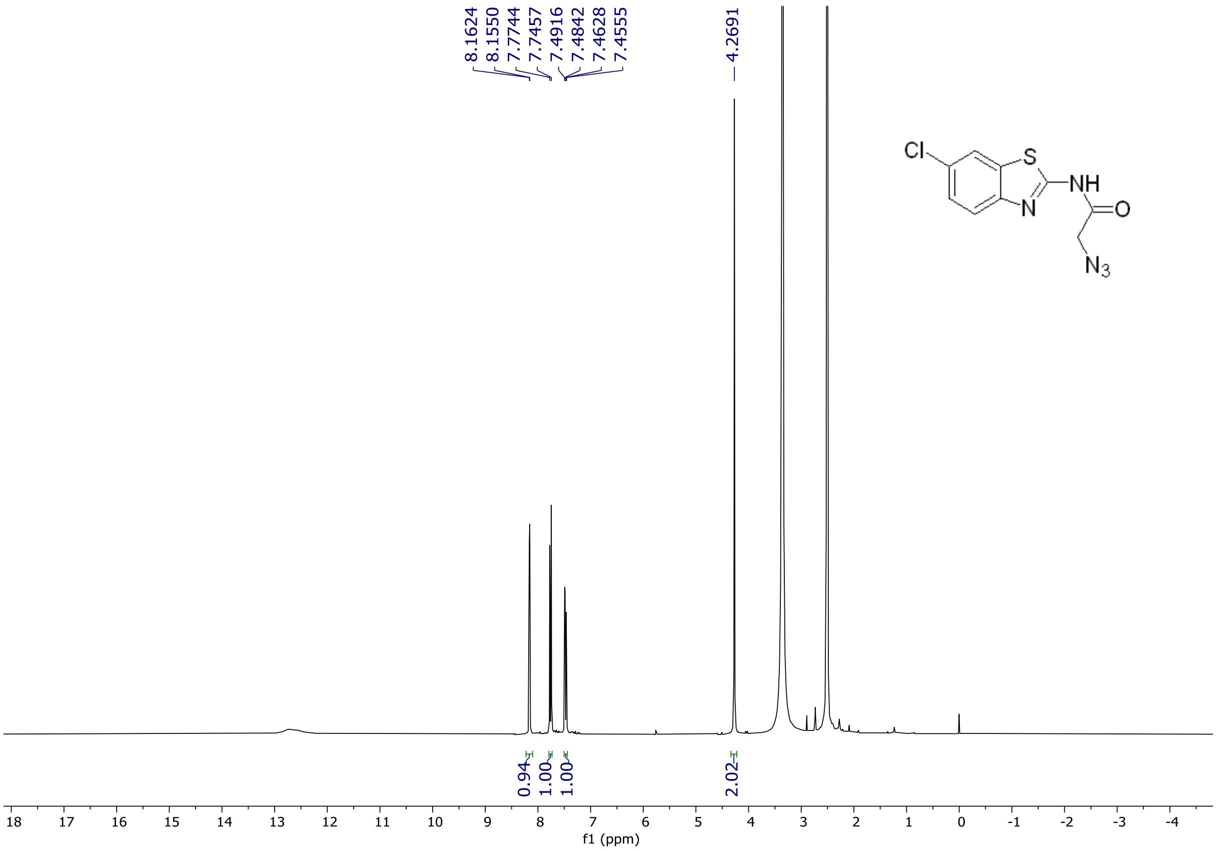

Figure S25. ^{1}H NMR spectrum of compound **4f** (300.13 MHz, DMSO-d_6).

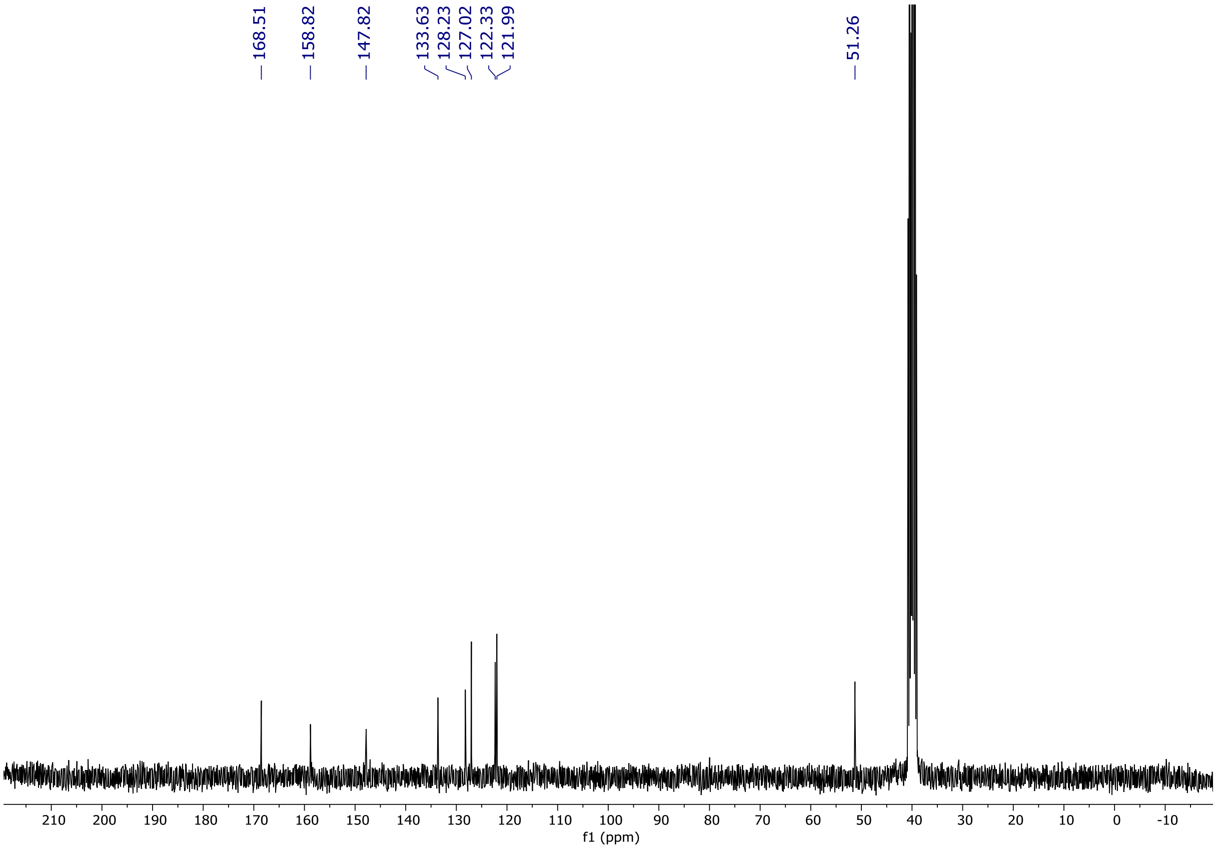

Figure S26. ^{13}C NMR spectrum of compound **4f** (75.47 MHz, DMSO-d_6).

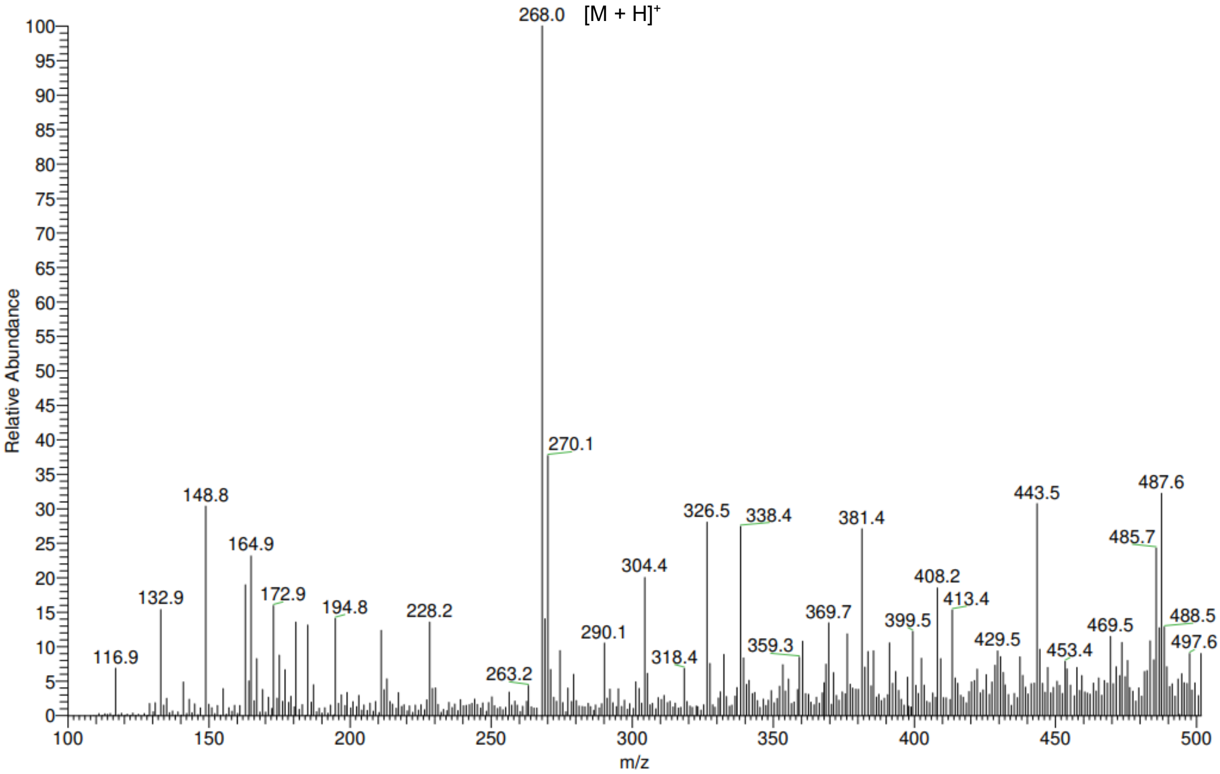

Figure S27. MS-ESI(+) spectrum of compound **4f**.

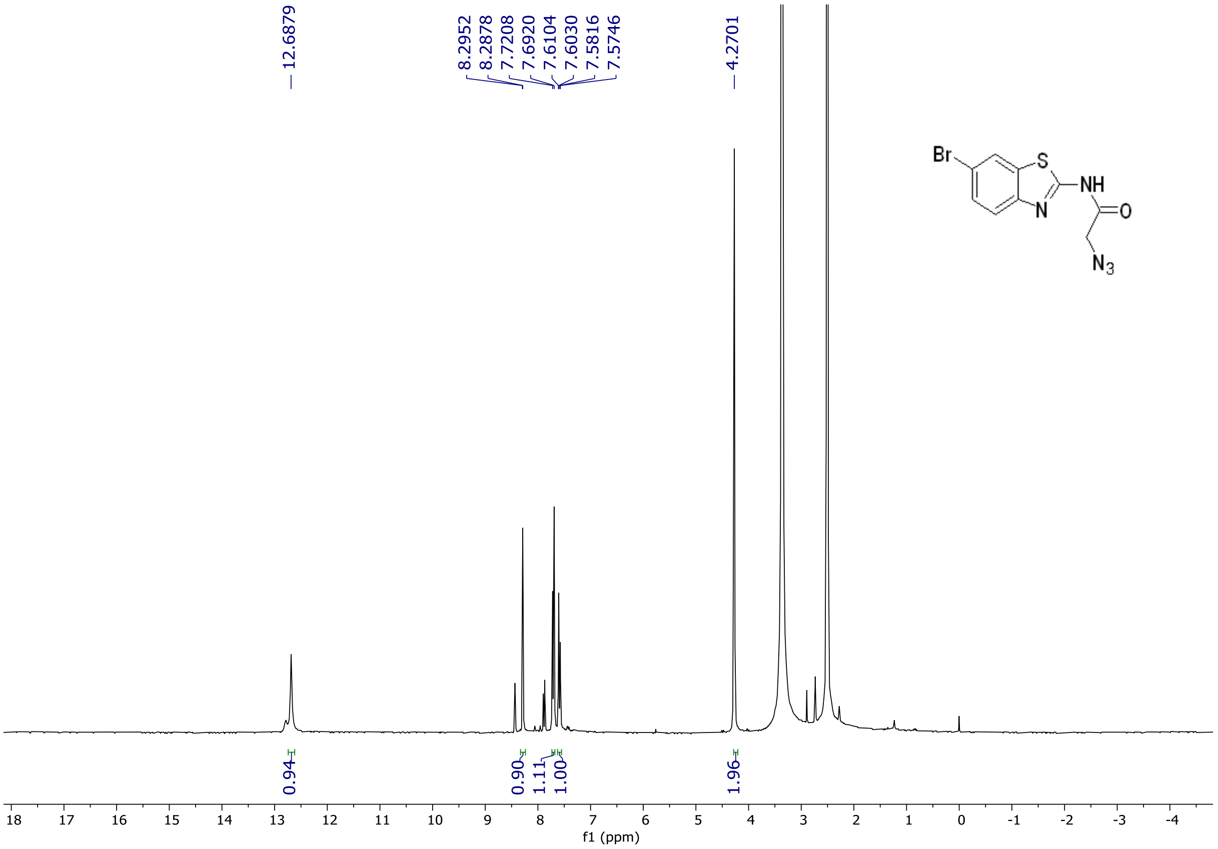

Figure S28. ^{1}H NMR spectrum of compound **4g** (300.13 MHz, DMSO-d_6).

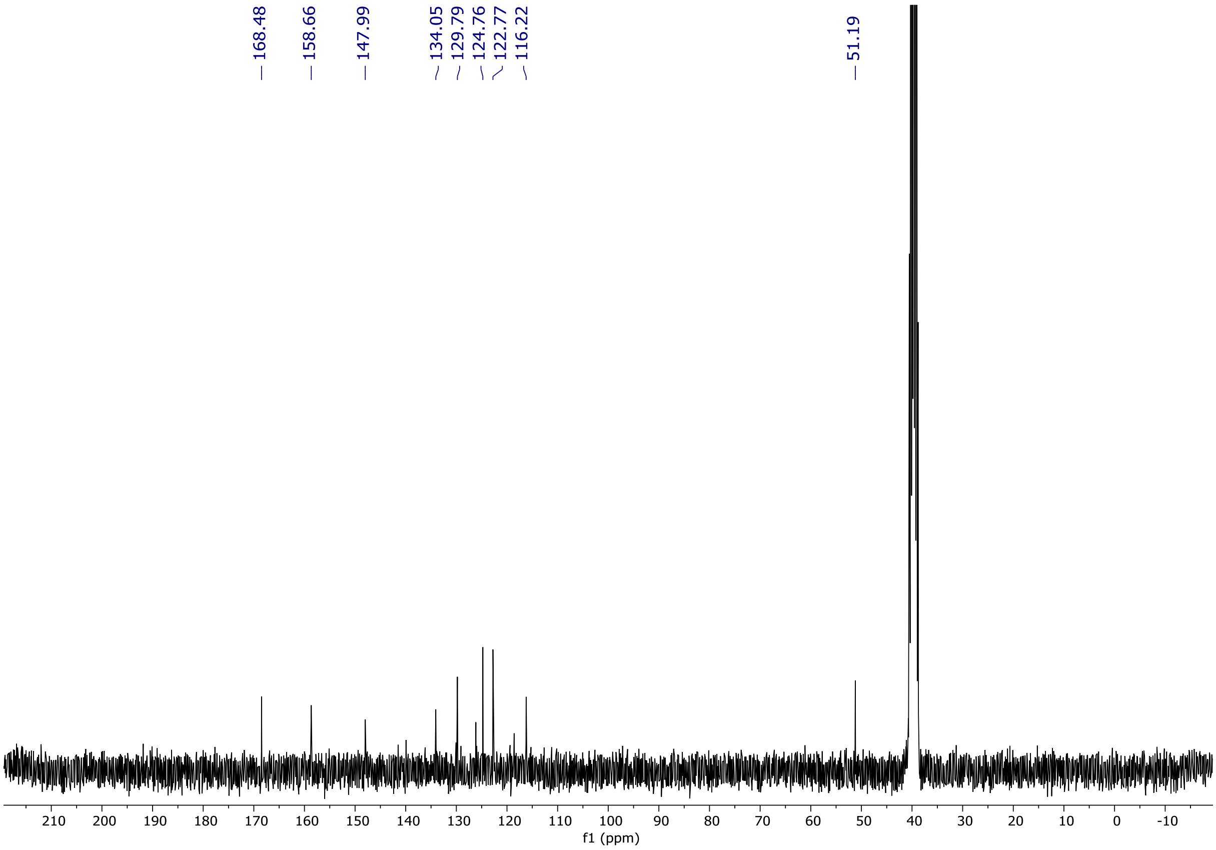

Figure S29. ^{13}C NMR spectrum of compound **4g** (75.47 MHz, DMSO-d_6).

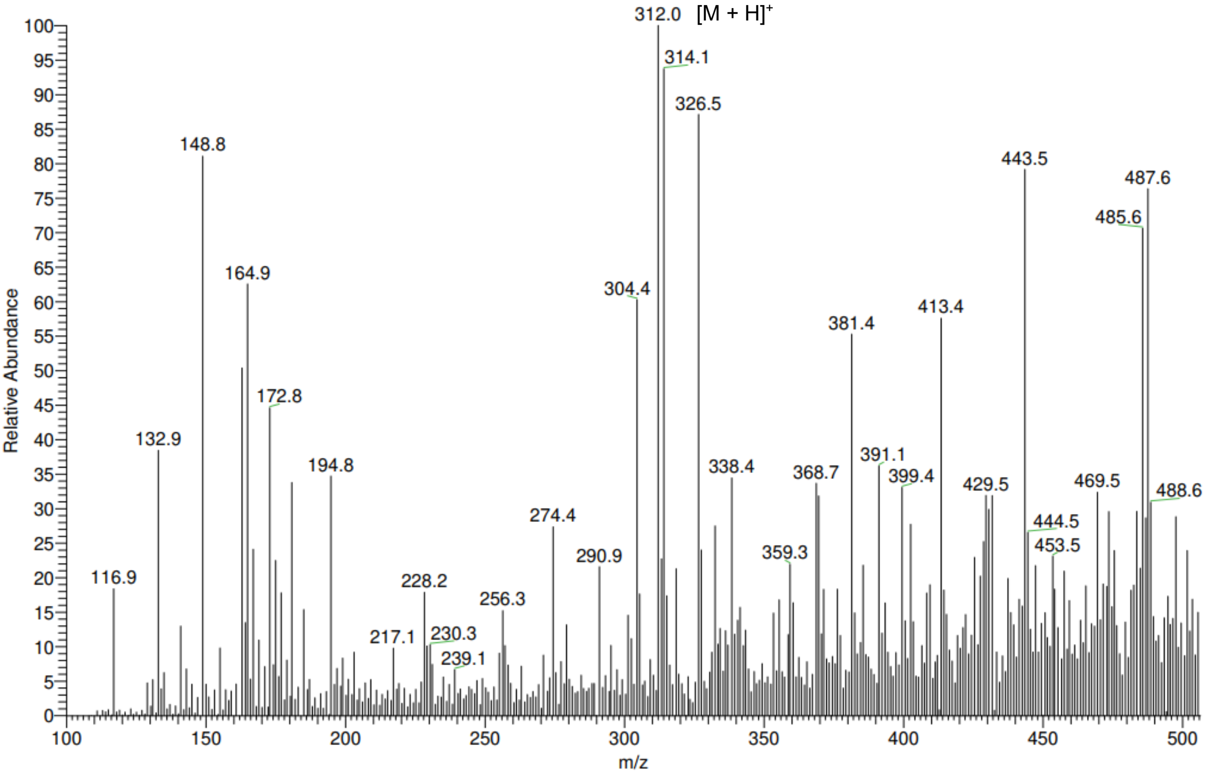

Figure S30. MS-ESI(+) spectrum of compound **4g**.

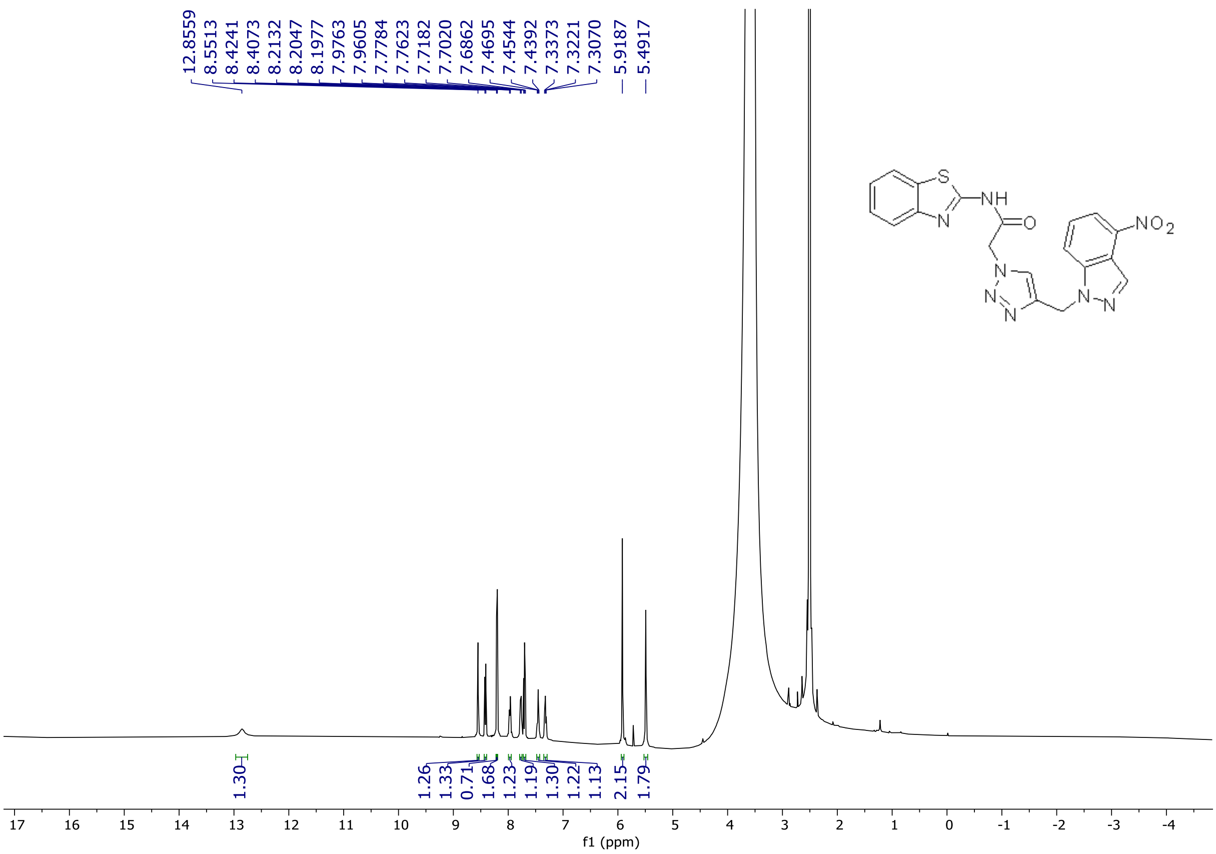

Figure S31. ^{1}H NMR spectrum of compound **5a** (500.13 MHz, DMSO-d_6).

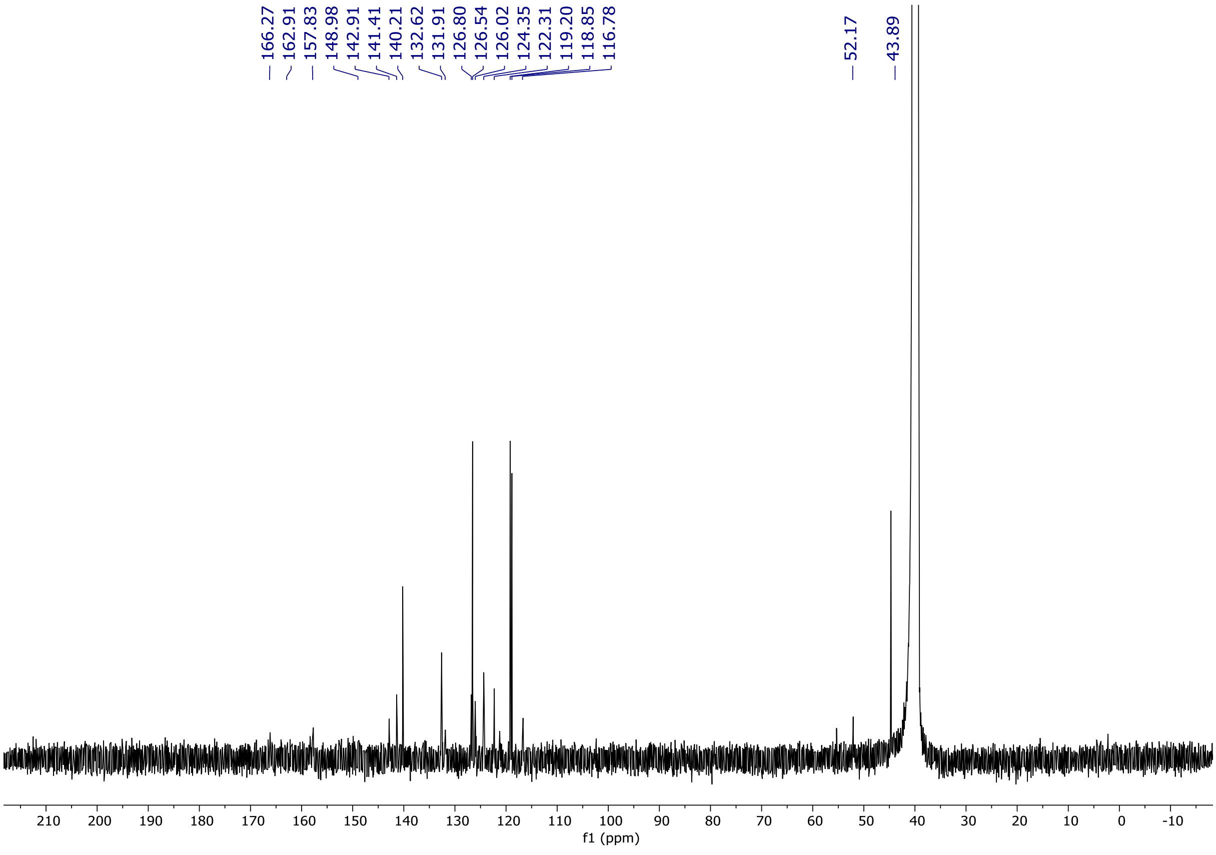

Figure S32. ^{13}C NMR spectrum of compound **5a** (125.76 MHz, DMSO-d_6).

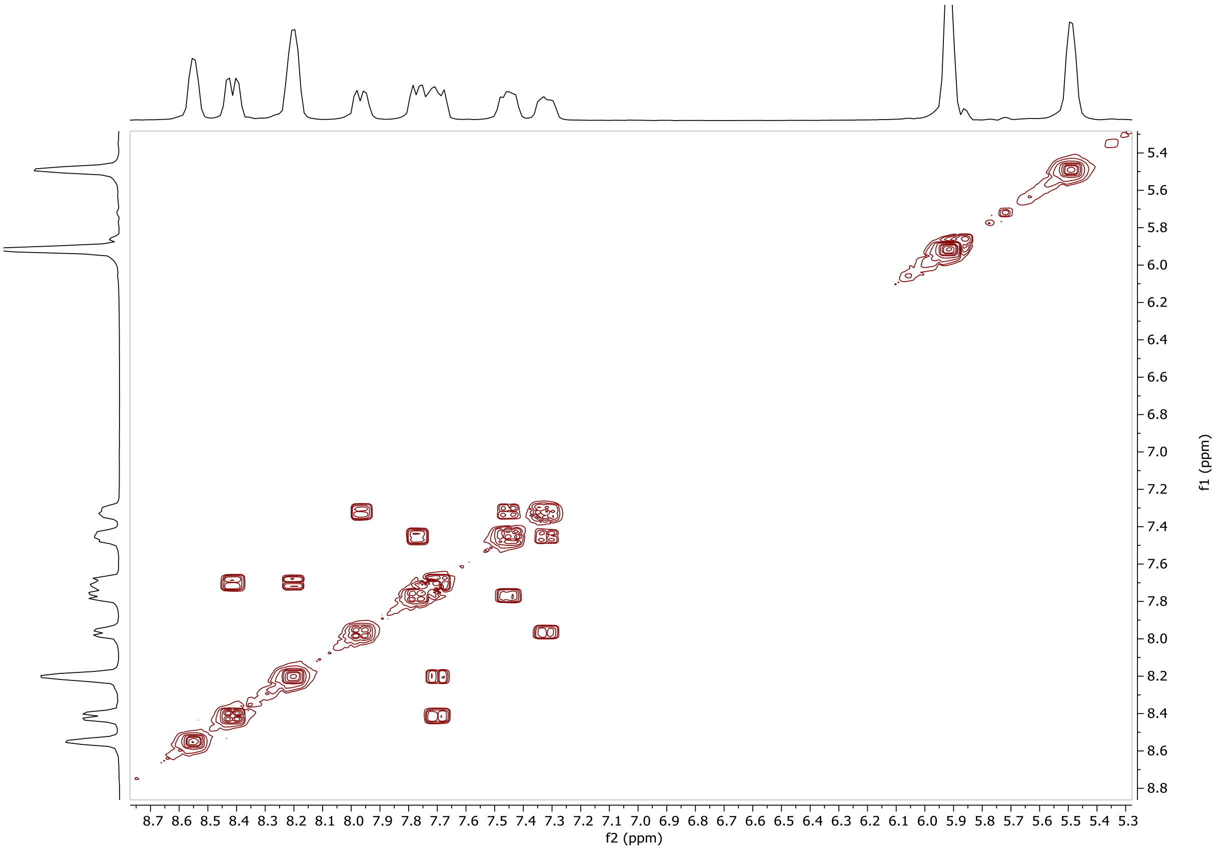

Figure S33. COSY($^1H/^1H$) spectrum of compound **5a** (300.13 MHz, DMSO-d_6).

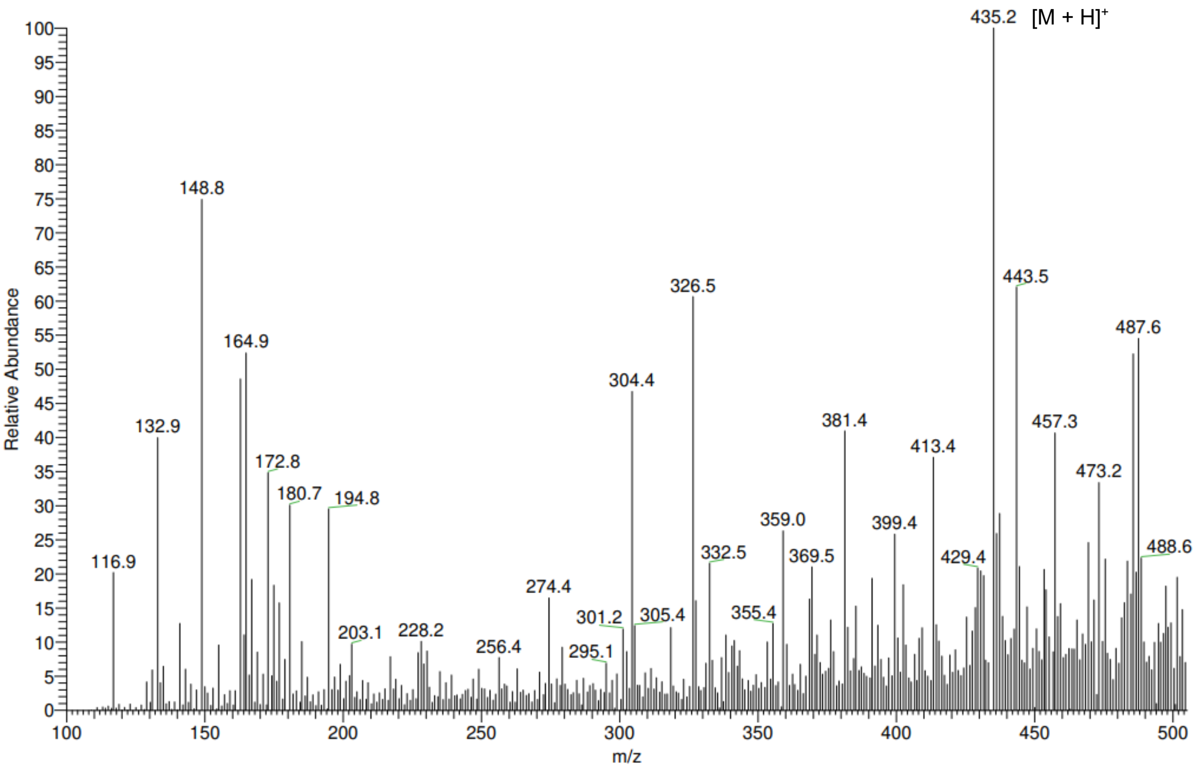

Figure S34. MS-ESI(+) spectrum of compound **5a**.

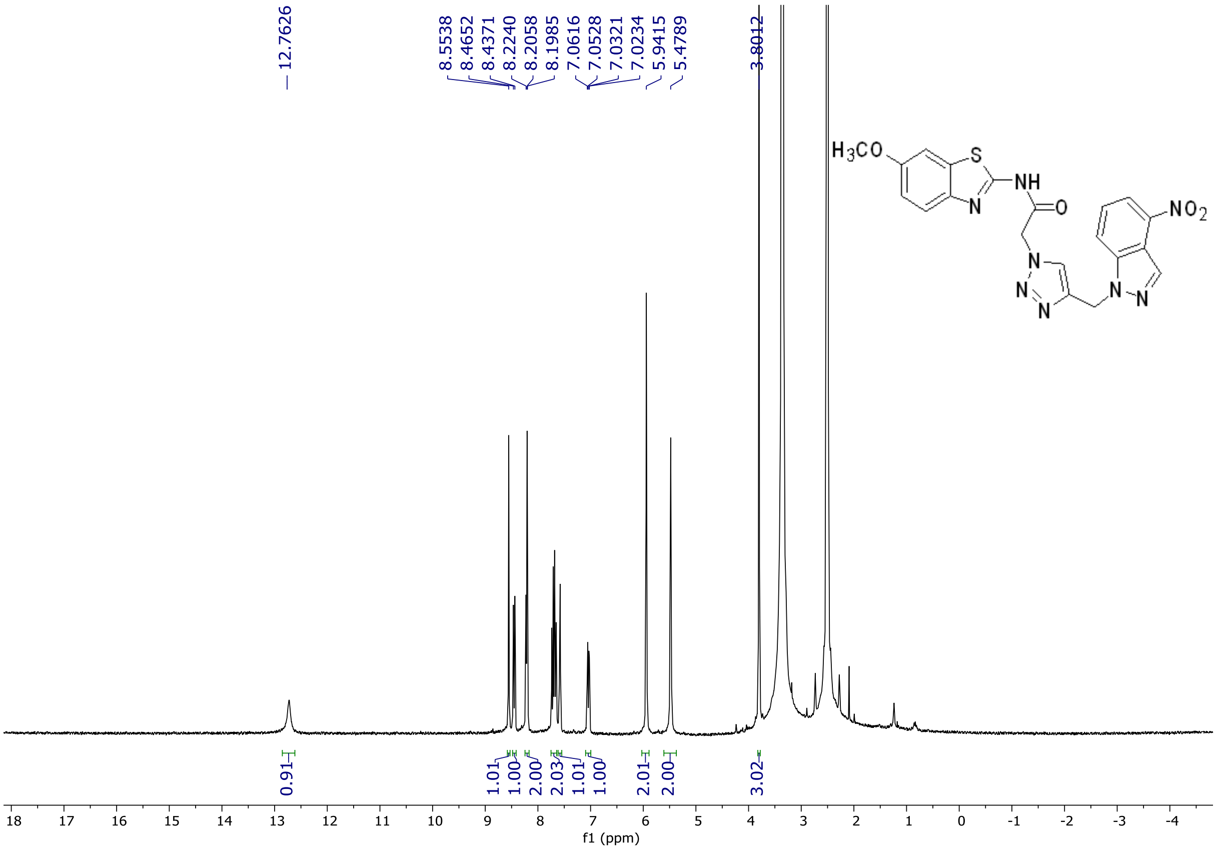

Figure S35. ^{1}H NMR spectrum of compound **5b** (500.13 MHz, DMSO-*d*$_6$).

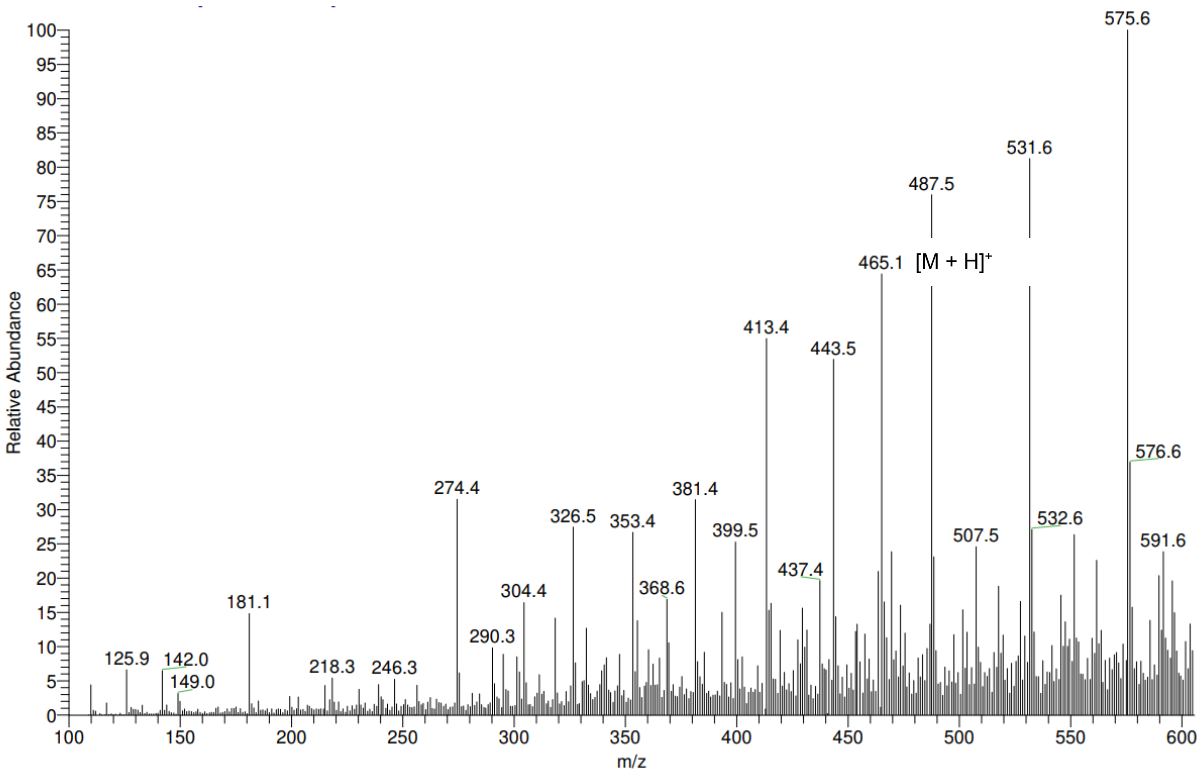

Figure S36. MS-ESI(+) spectrum of compound **5b**.

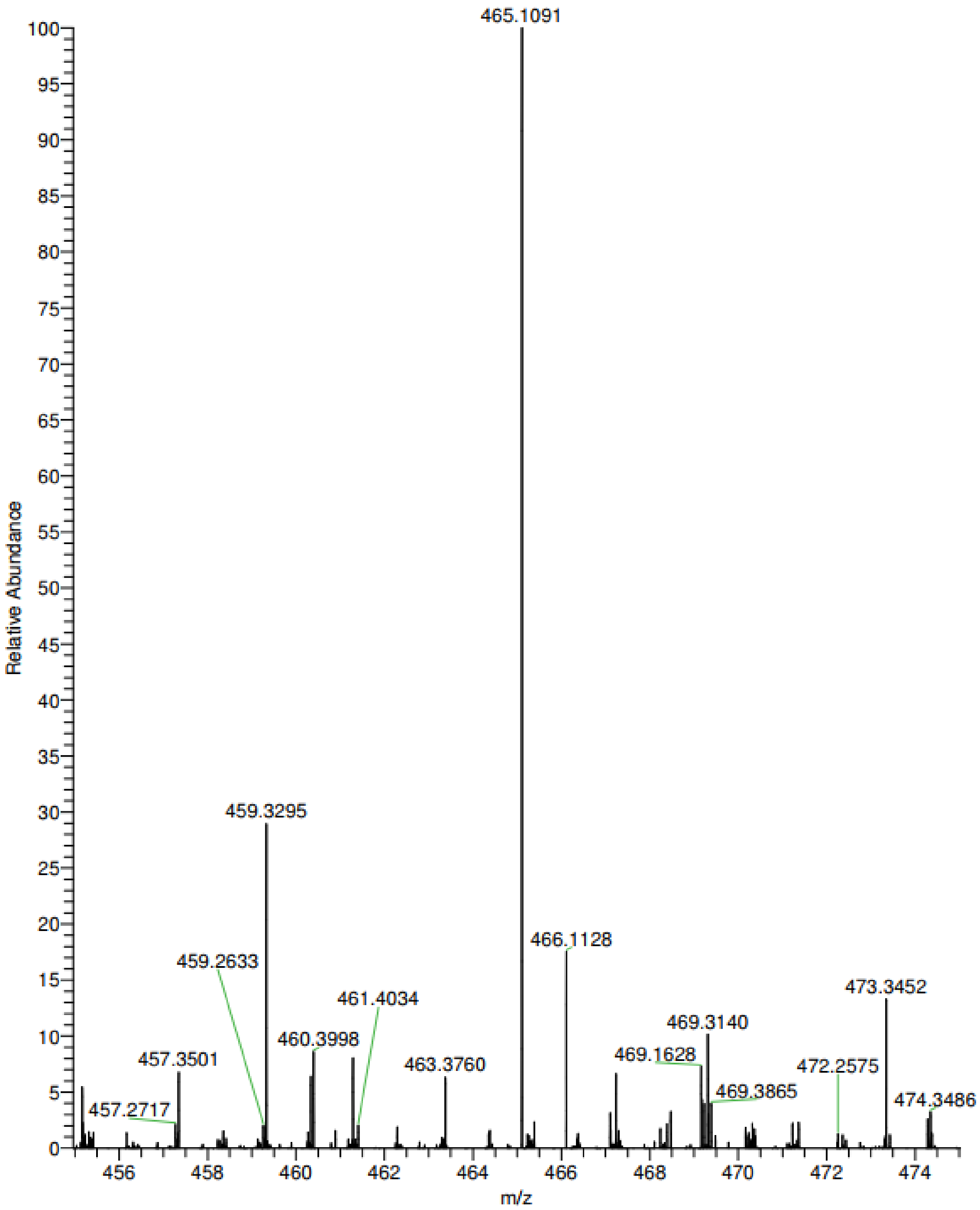

Figure S37. HRMS-ESI(+) spectrum of compound **5b**.

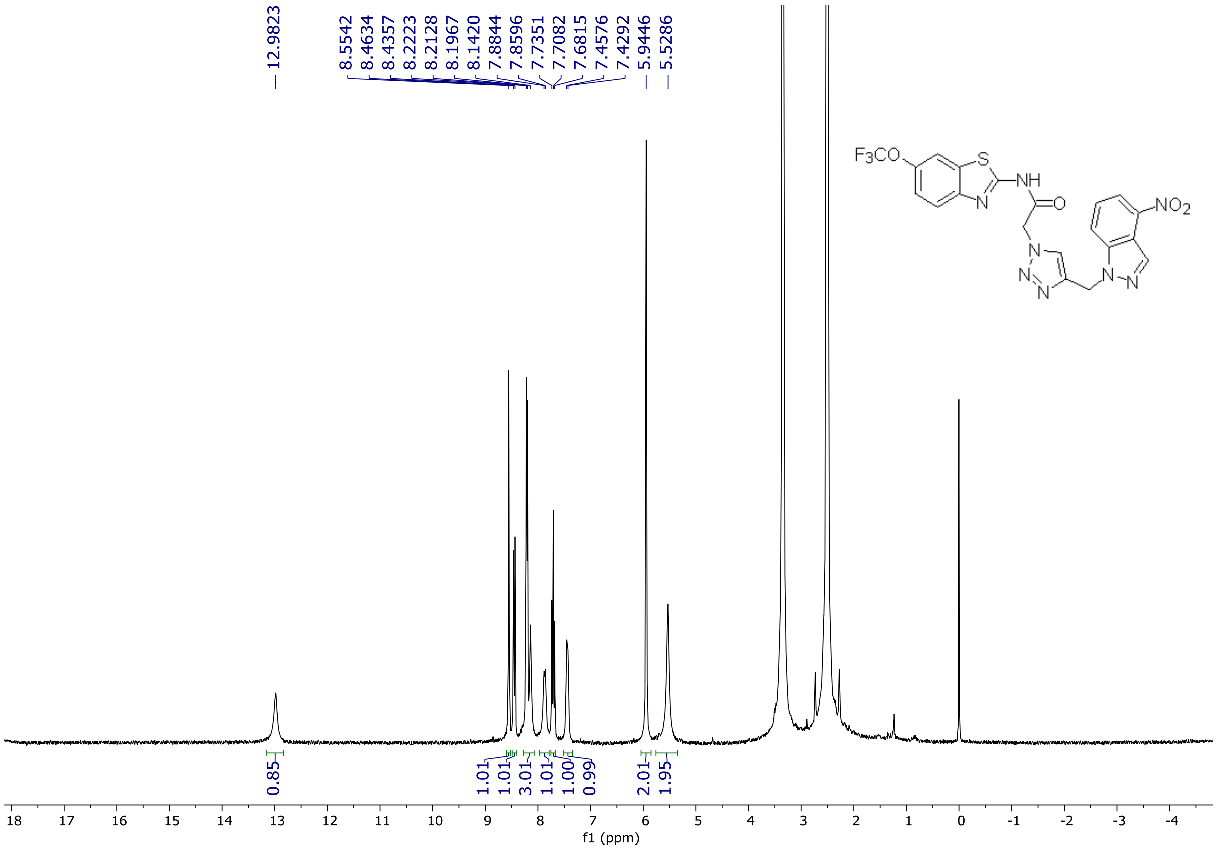

Figure S38. ^{1}H NMR spectrum of compound **5c** (500.13 MHz, DMSO-d_6).

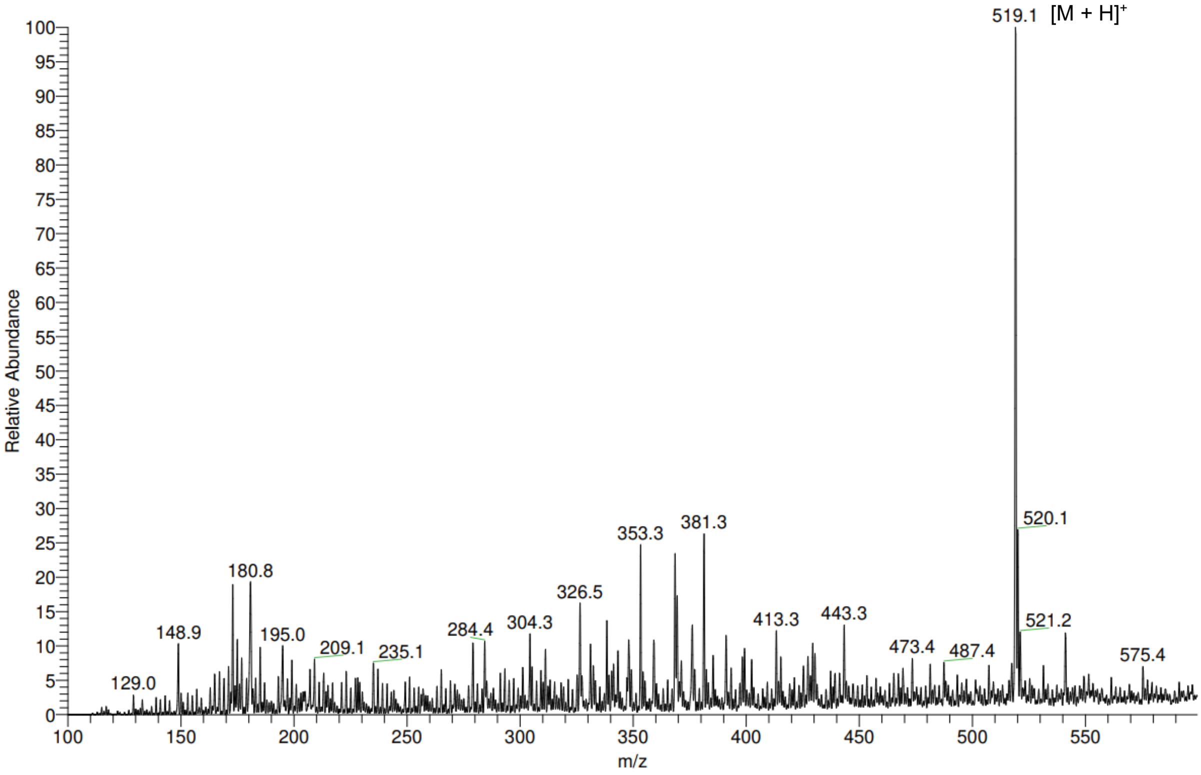

Figure S39. MS-ESI(+) spectrum of compound **5c**.

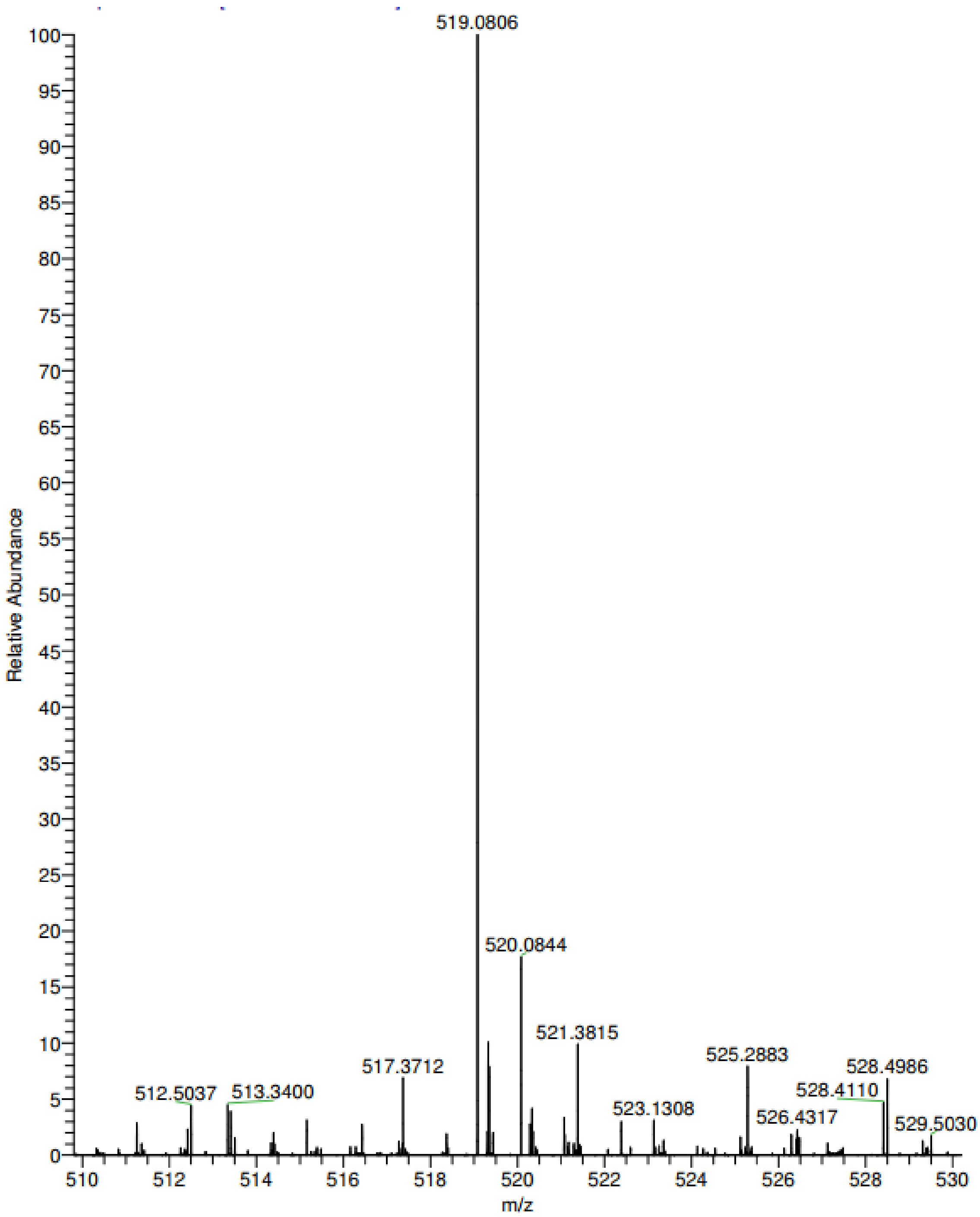

Figure S40. HRMS-ESI(+) spectrum of compound **5c**.

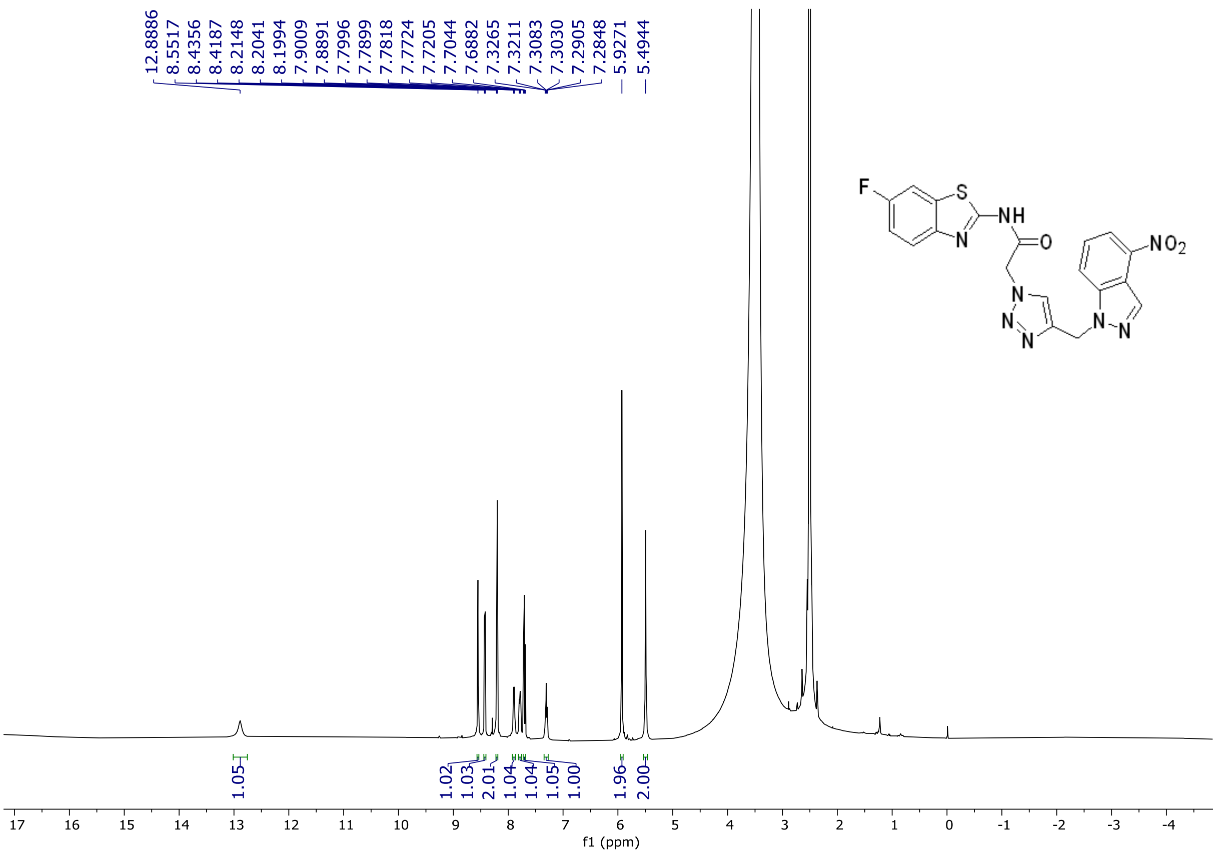

Figure S41. ^{1}H NMR spectrum of compound **5d** (500.13 MHz, DMSO-d_6).

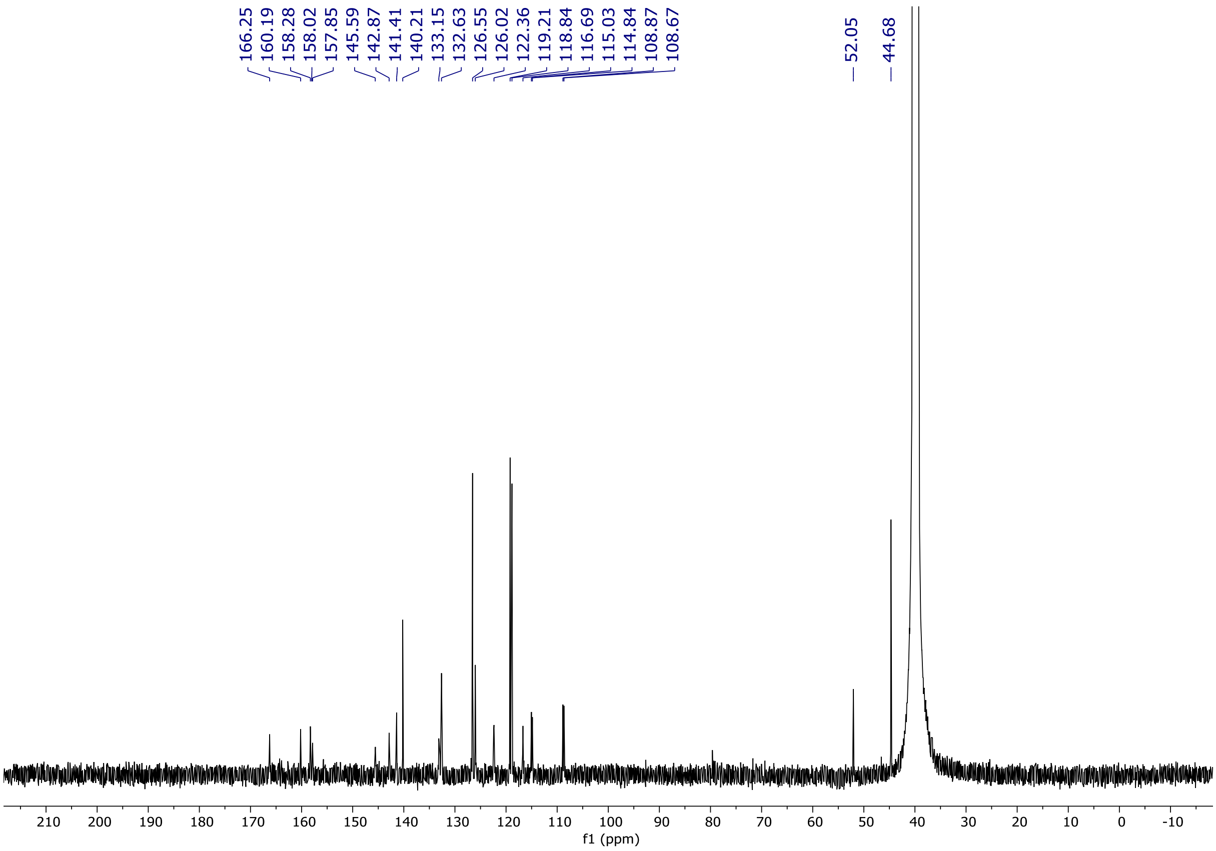

Figure S42. ^{13}C NMR spectrum of compound **5d** (125.76 MHz, DMSO-d_6).

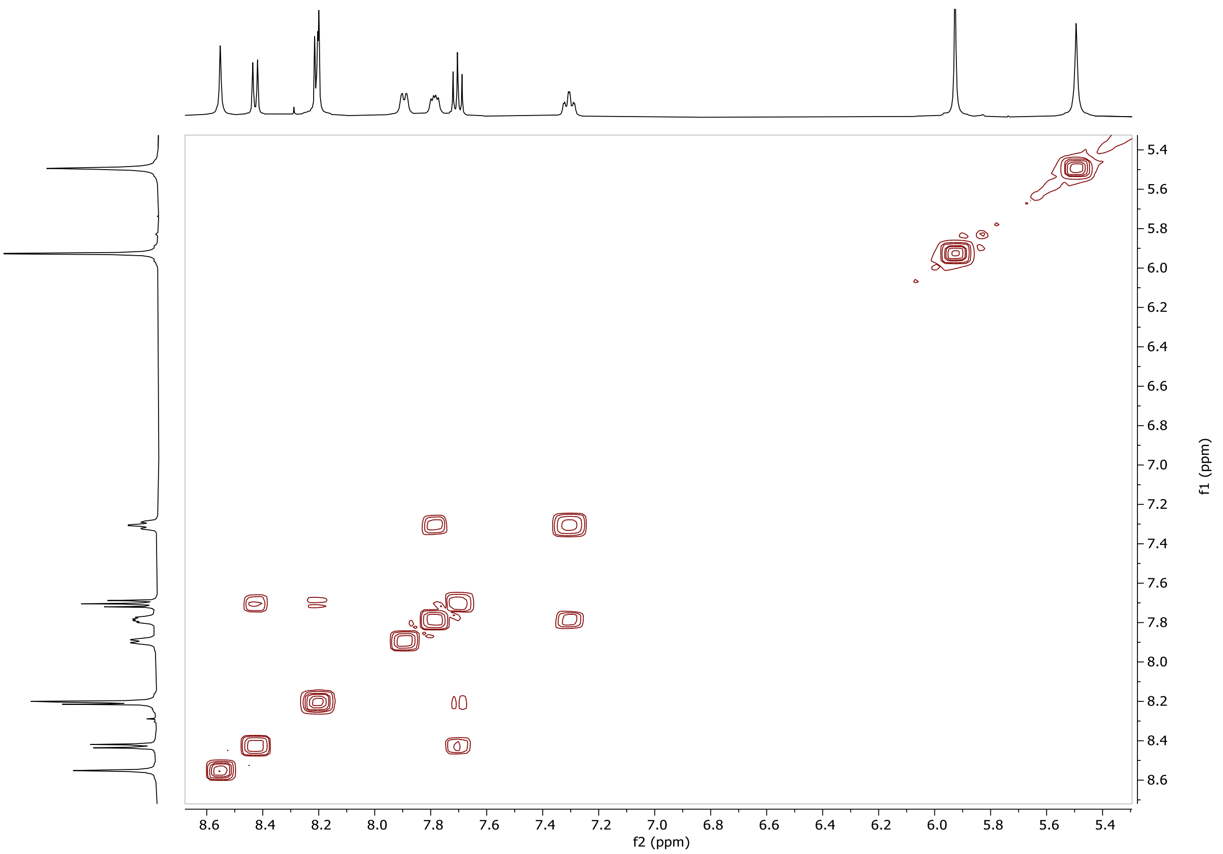

Figure S43. COSY($^1H/^1H$) spectrum of compound **5d** (500.13 MHz, DMSO-d_6).

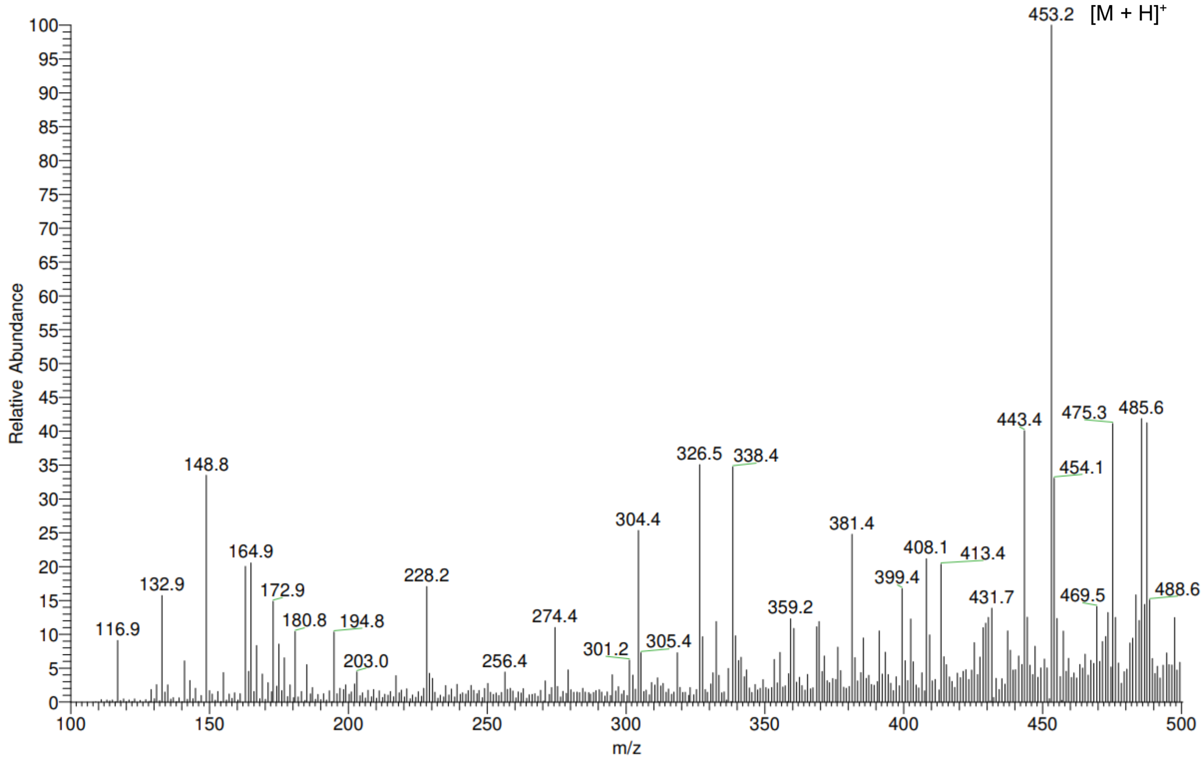

Figure S44. MS-ESI(+) spectrum of compound **5d**.

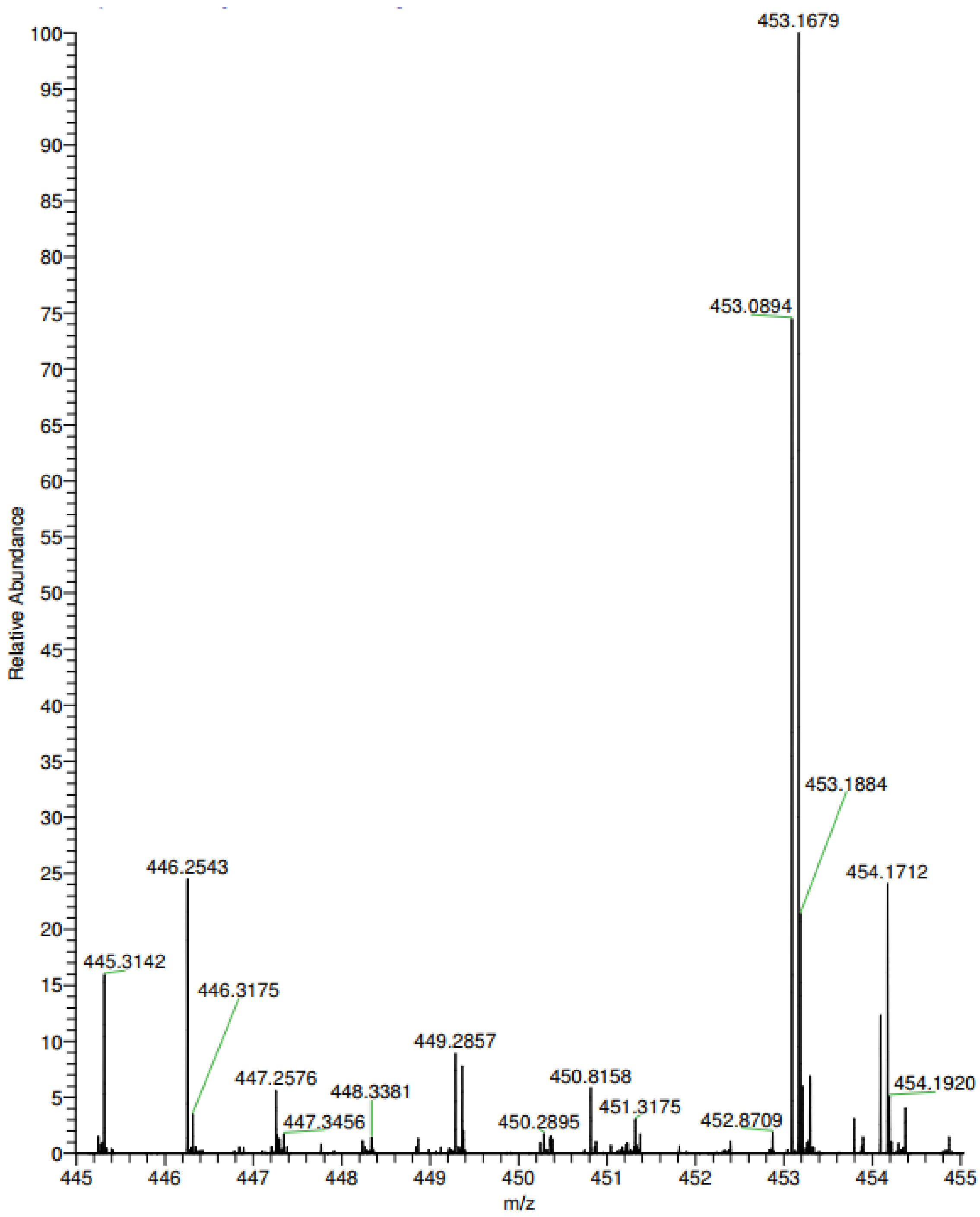

Figure S45. HRMS-ESI(+) spectrum of compound **5d**.

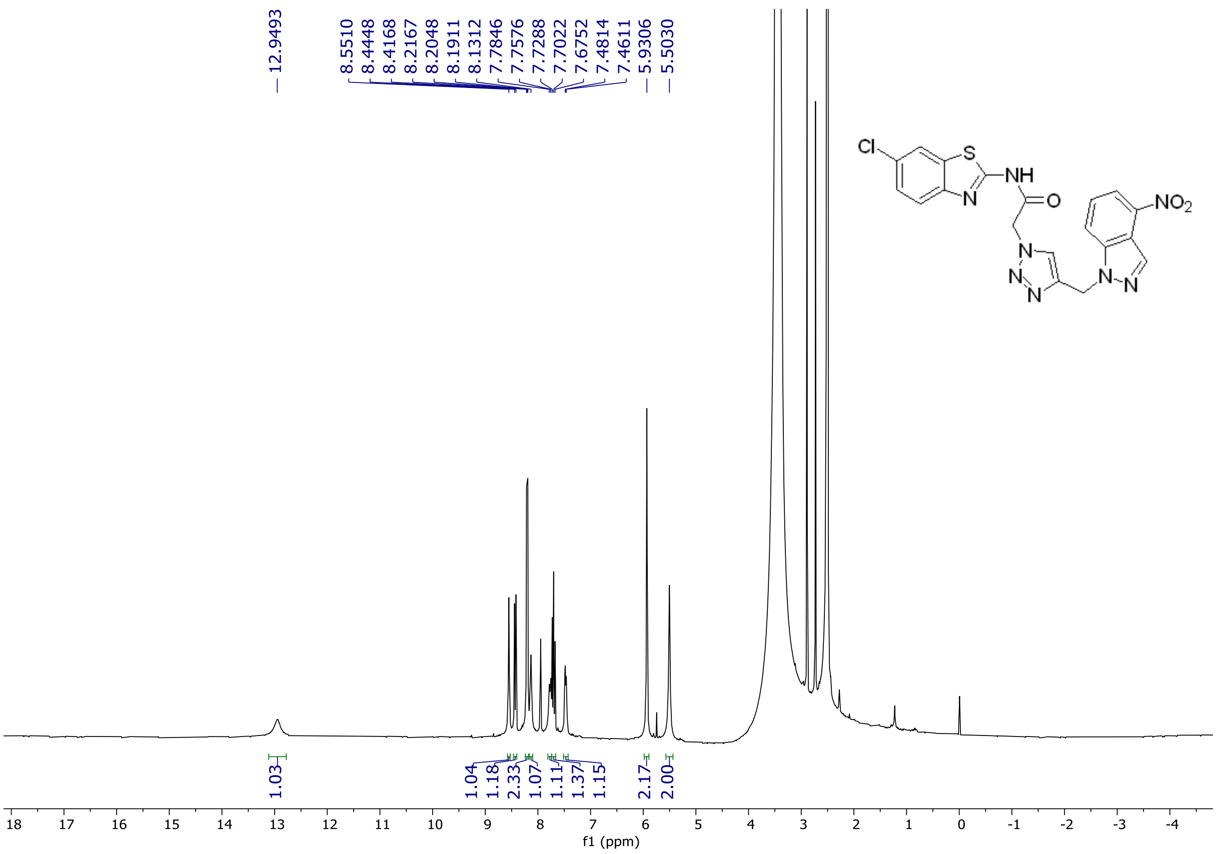

Figure S46. ^{1}H NMR spectrum of compound **5e** (300.13 MHz, DMSO-d_6).

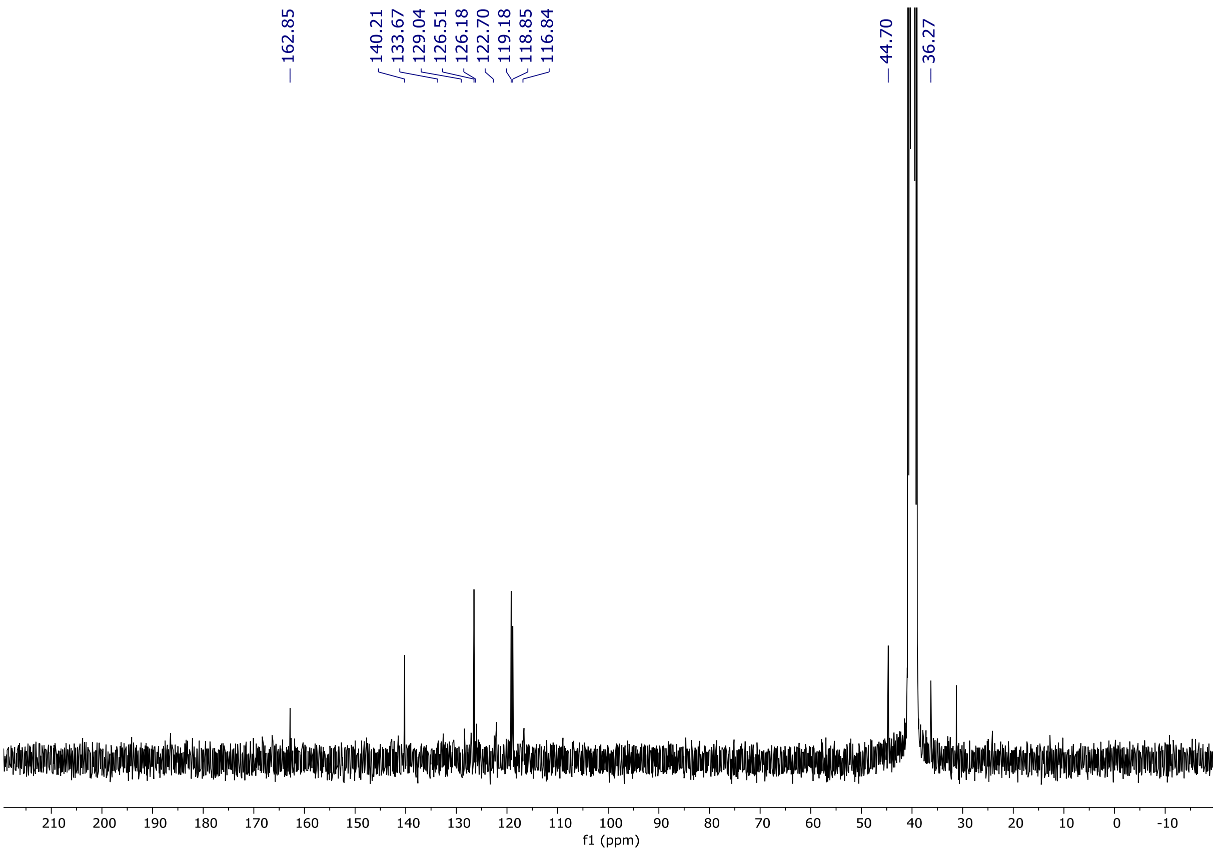

Figure S47. ^{13}C NMR spectrum of compound **5e** (125.76 MHz, DMSO-d_6).

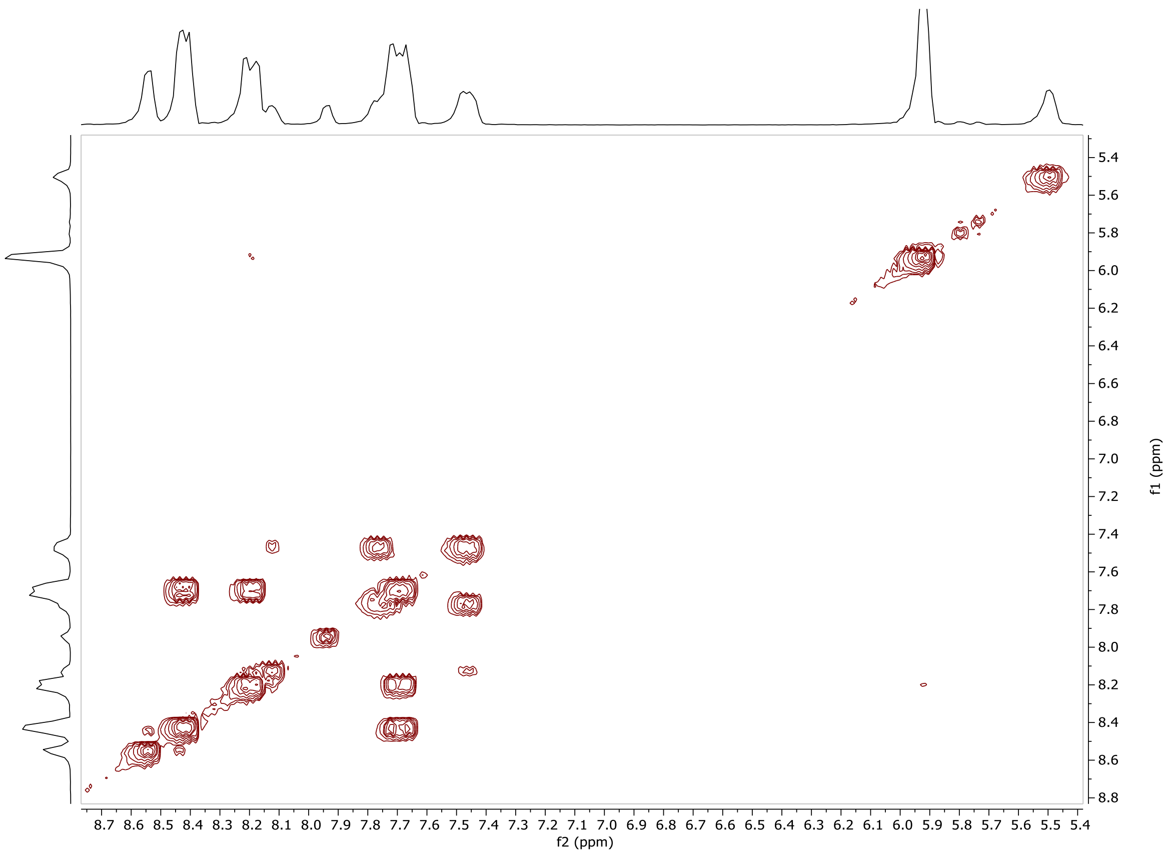

Figure S48. COSY($^1H/^1H$) spectrum of compound **5e** (500.13 MHz, DMSO-d_6).

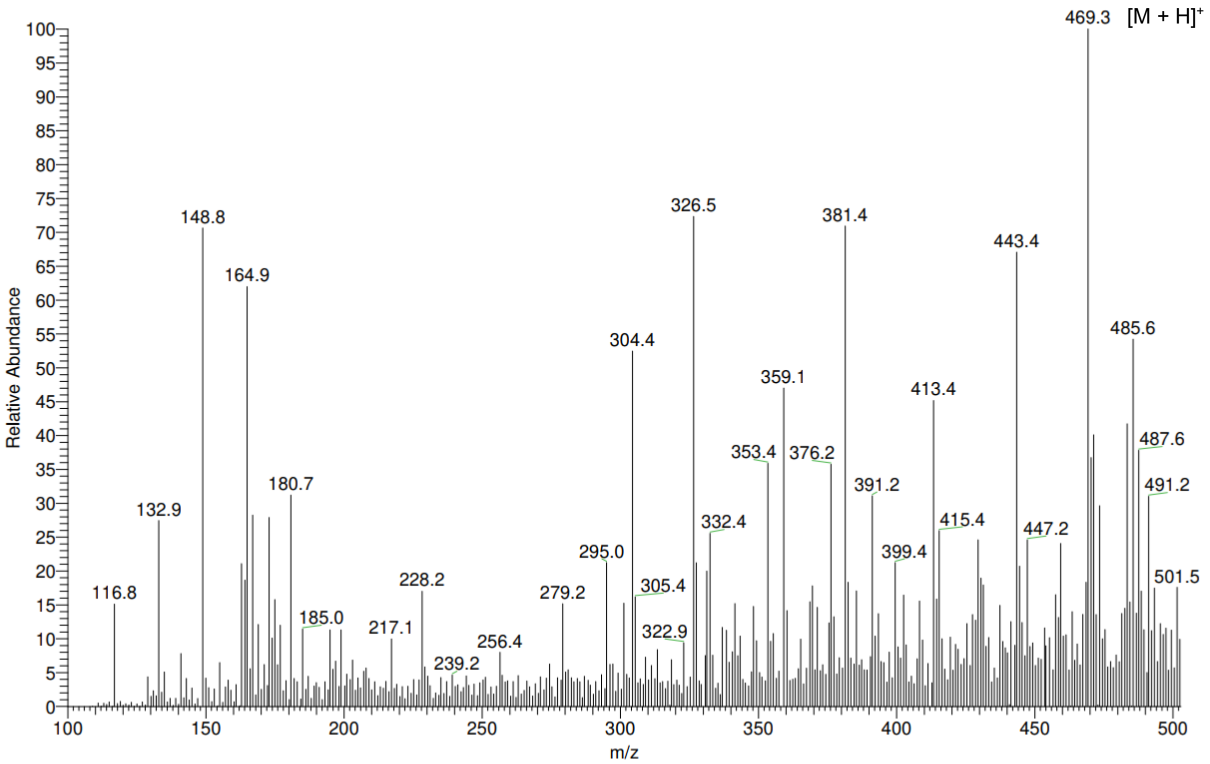

Figure S49. MS-ESI(+) spectrum of compound **5e**.

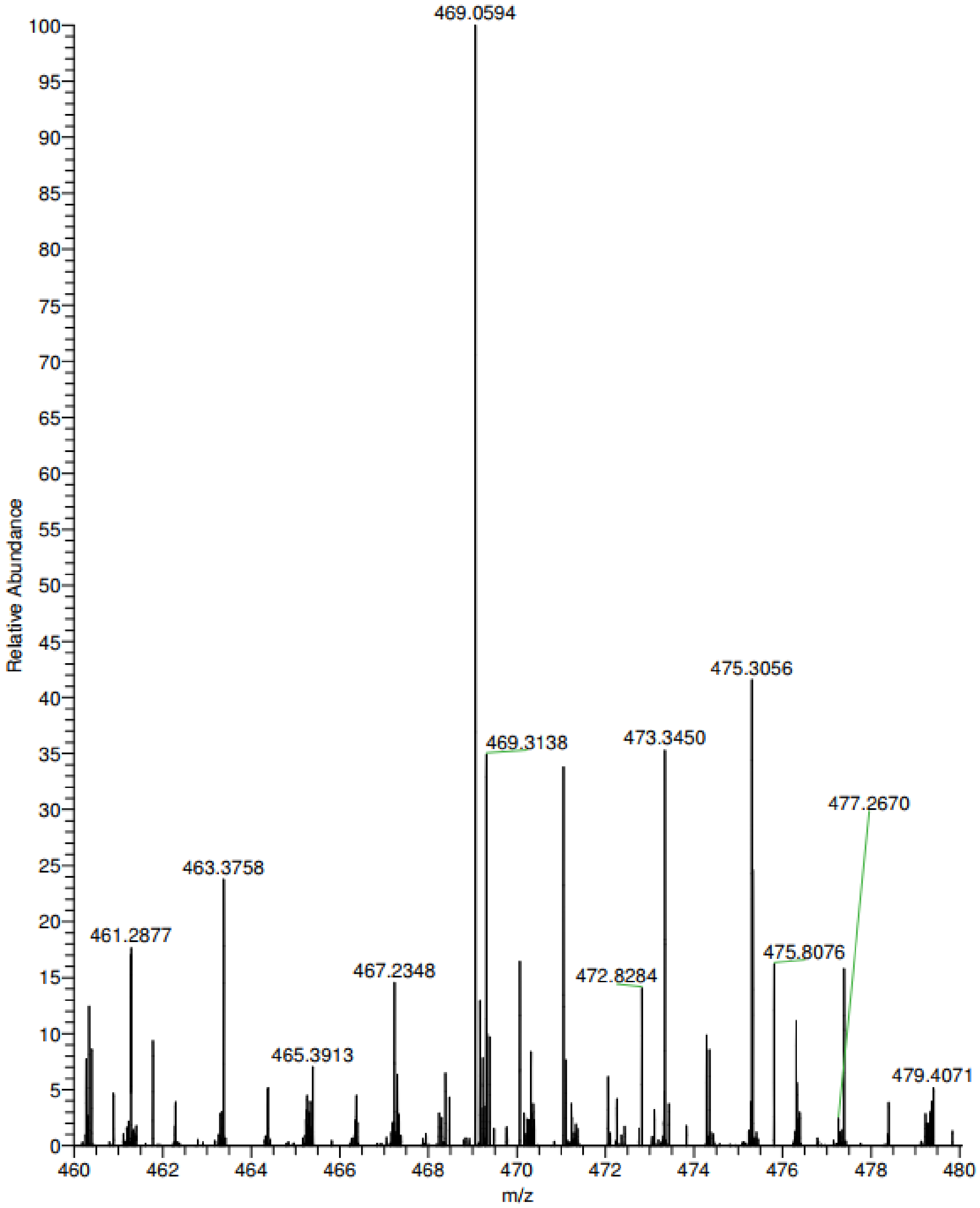

Figure S50. HRMS-ESI(+) spectrum of compound **5e**.

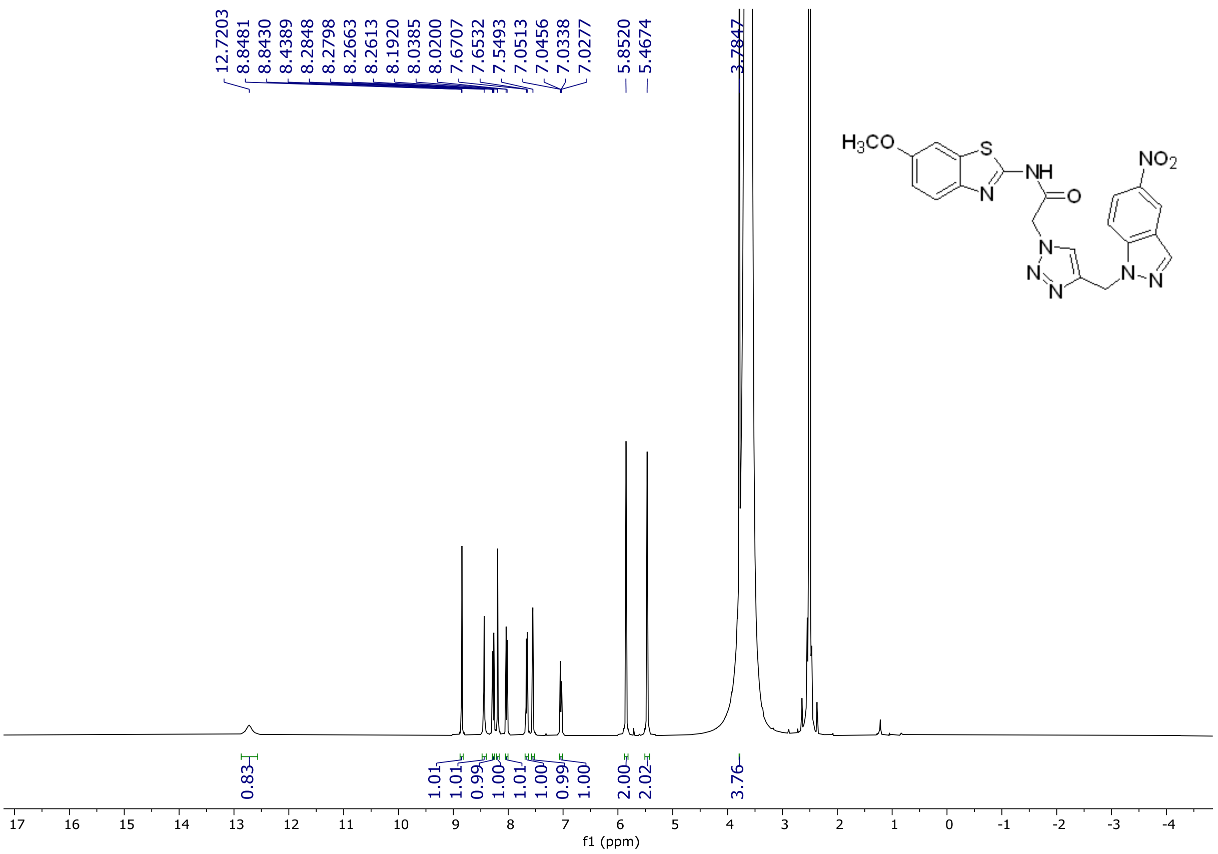

Figure S51. ^{1}H NMR spectrum of compound **5f** (500.13 MHz, DMSO-d_6).

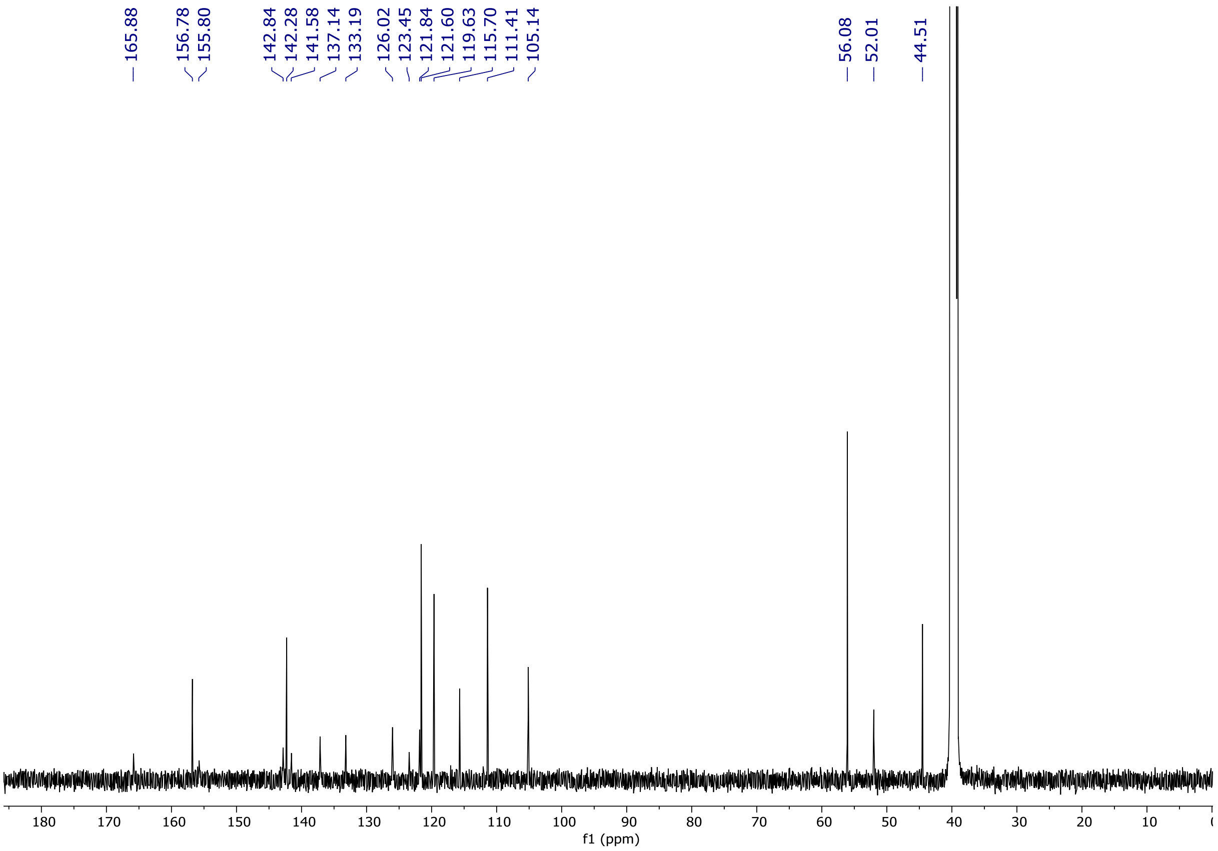

Figure S52. ^{13}C NMR spectrum of compound **5f** (125.76 MHz, DMSO-d_6).

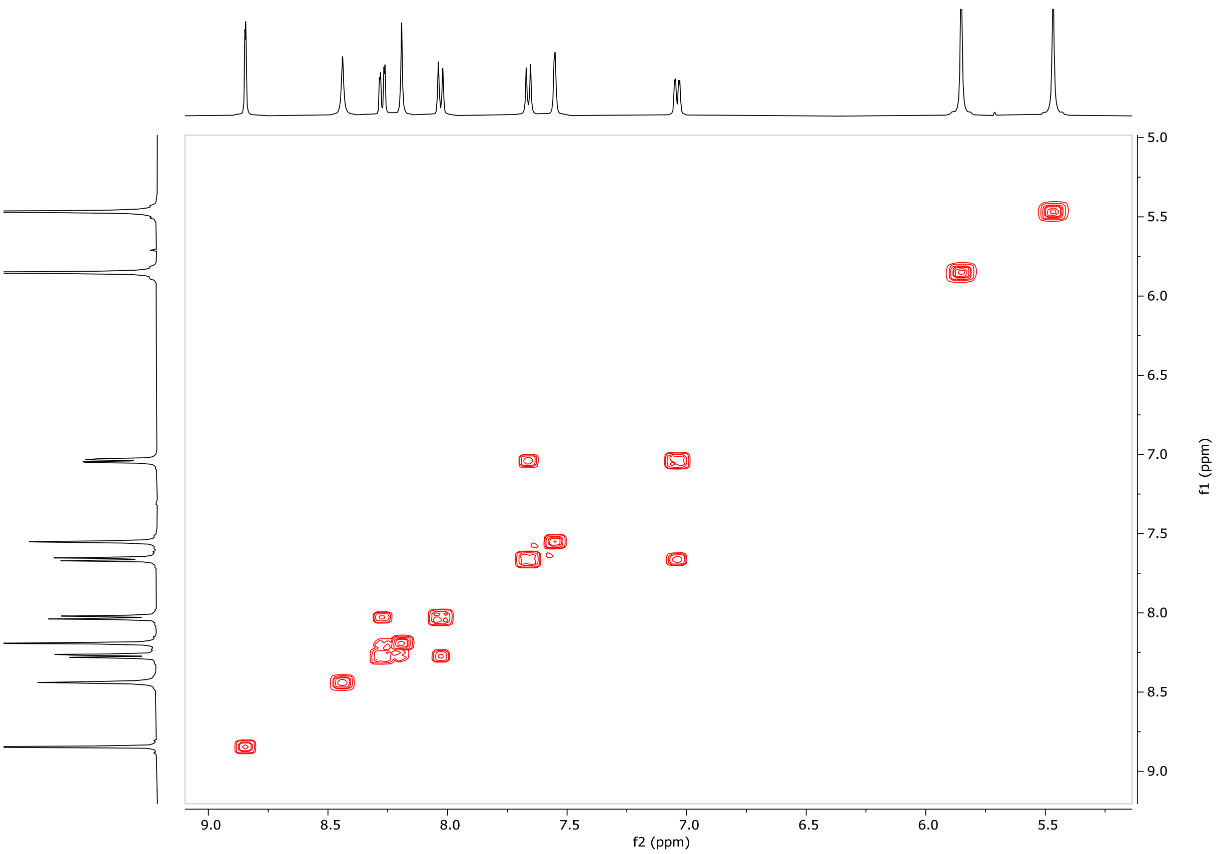

Figure S53. COSY($^1H/^1H$) spectrum of compound **5f** (500.13 MHz, DMSO-*d*$_6$).

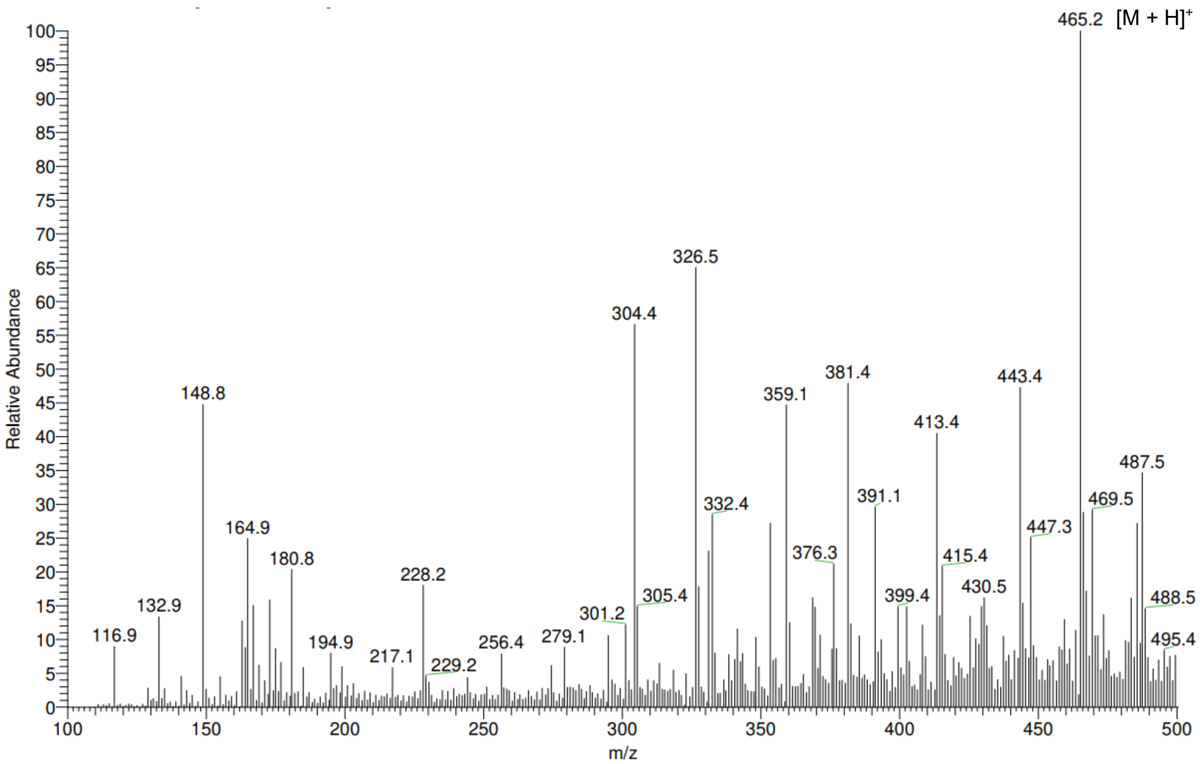

Figure S54. MS-ESI(+) spectrum of compound **5f**.

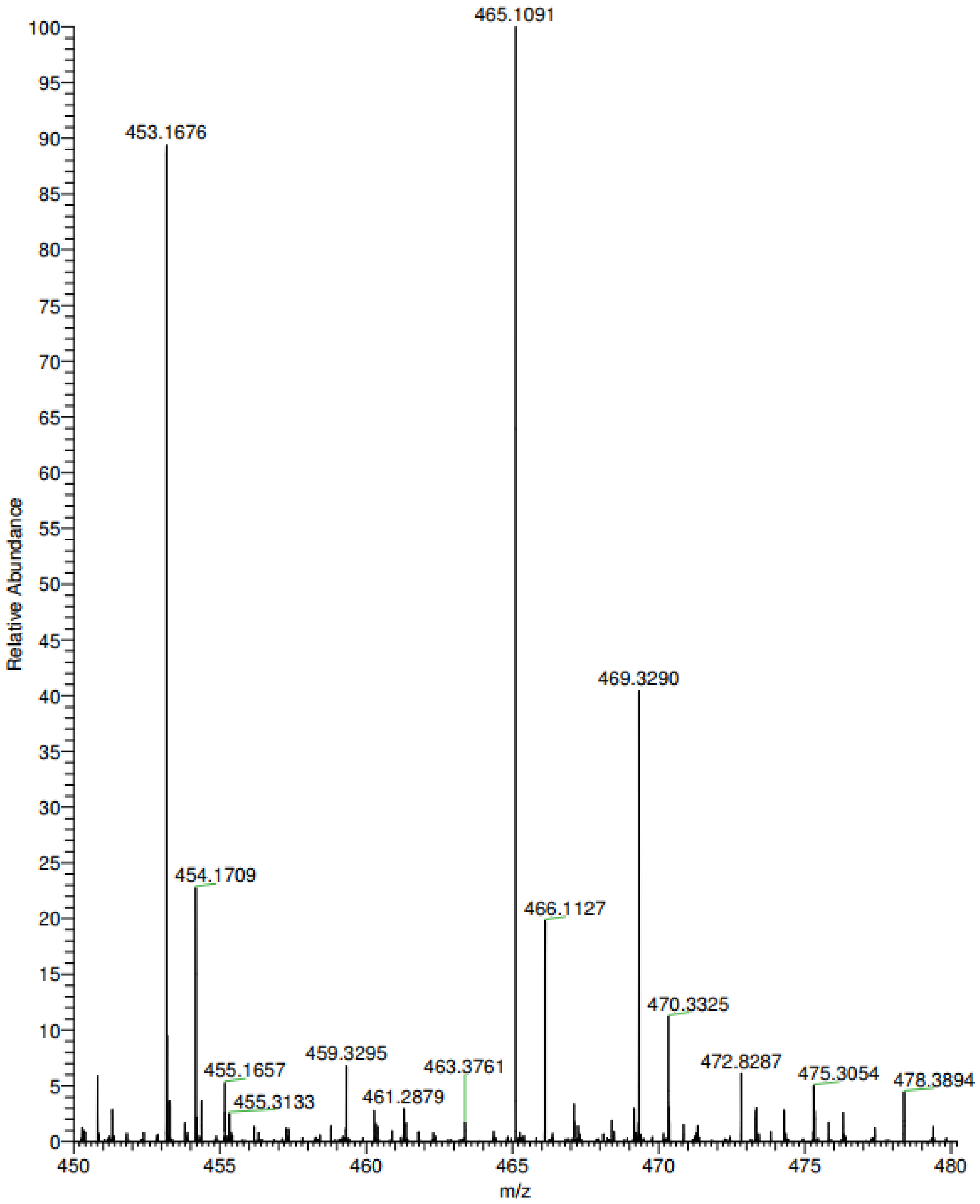

Figure S55. HRMS-ESI(+) spectrum of compound **5f**.

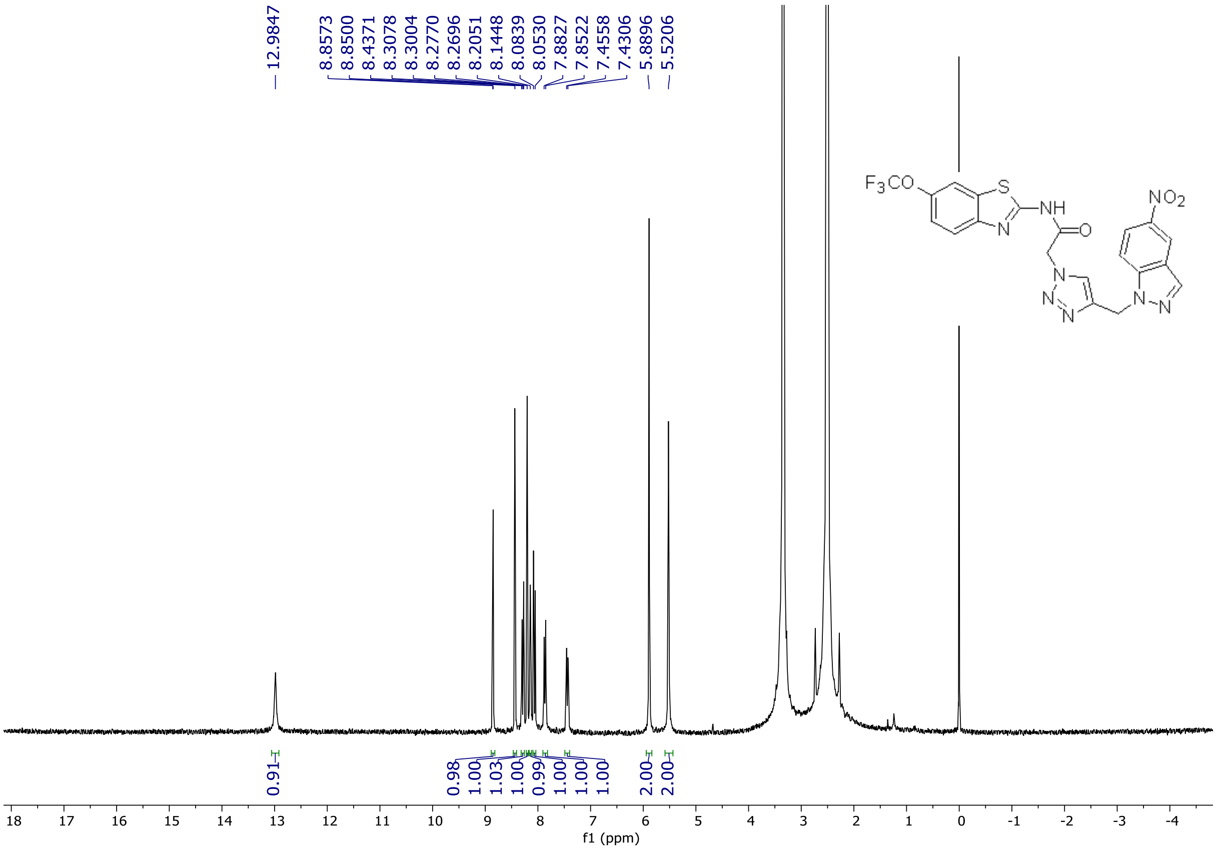

Figure S56. ^{1}H NMR spectrum of compound **5g** (300.13 MHz, DMSO-d_6).

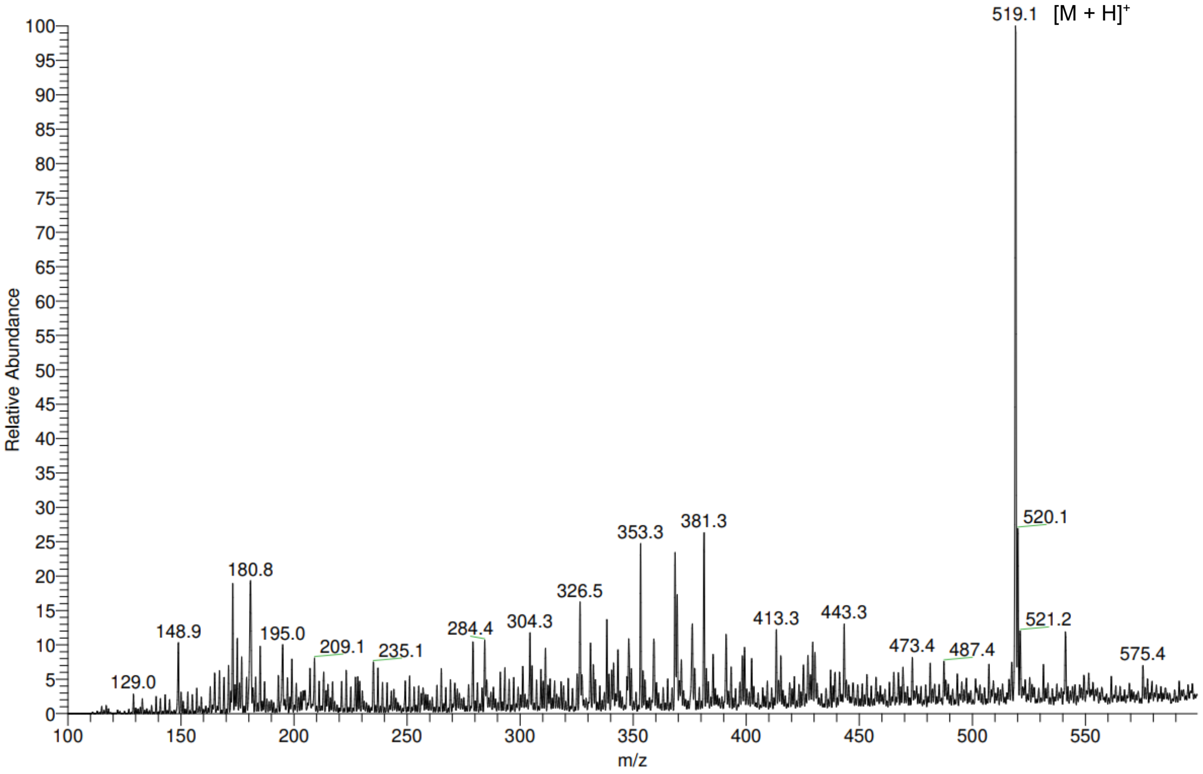

Figure S57. MS-ESI(+) spectrum of compound **5g**.

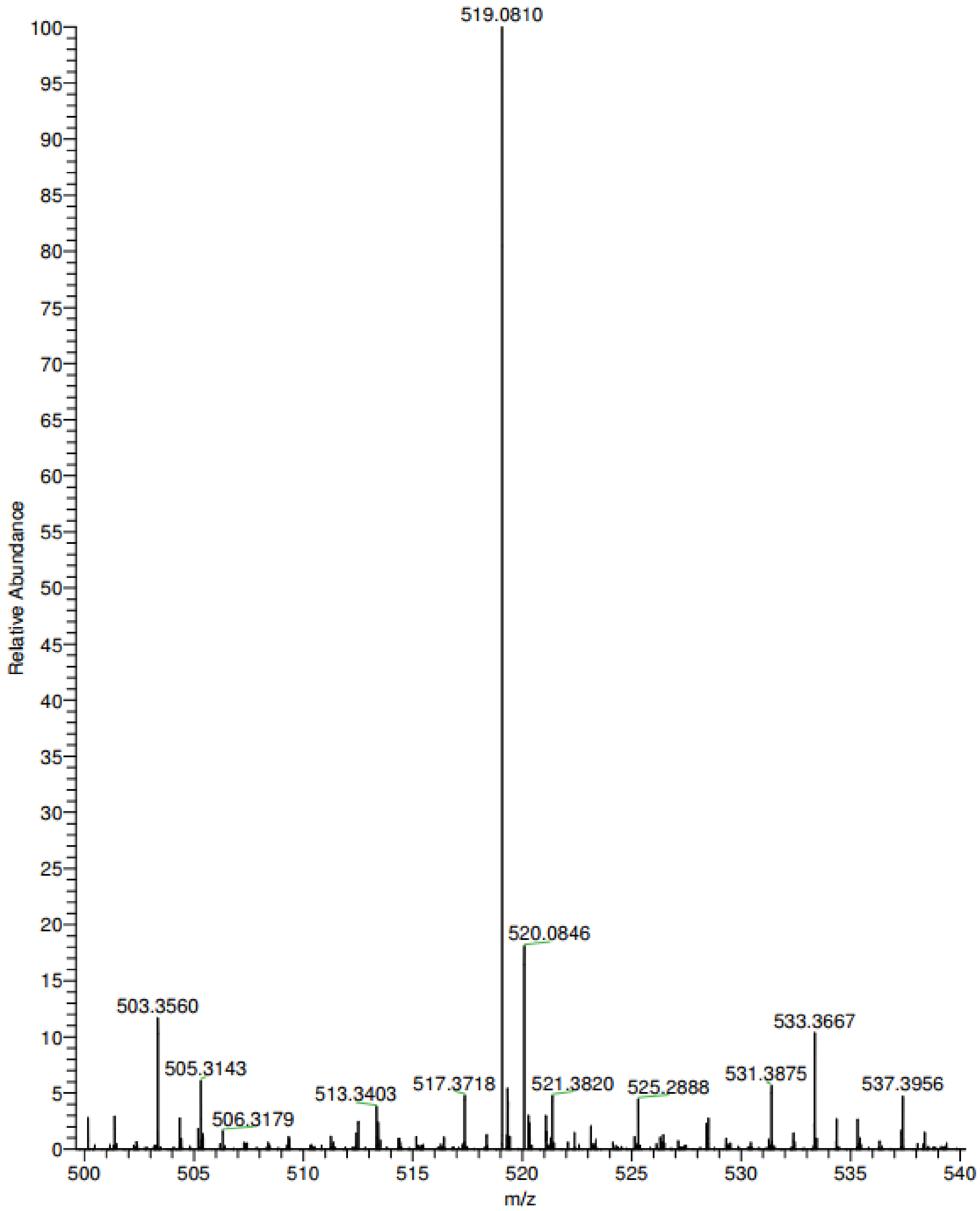

Figure S58. HRMS-ESI(+) spectrum of compound **5g**.

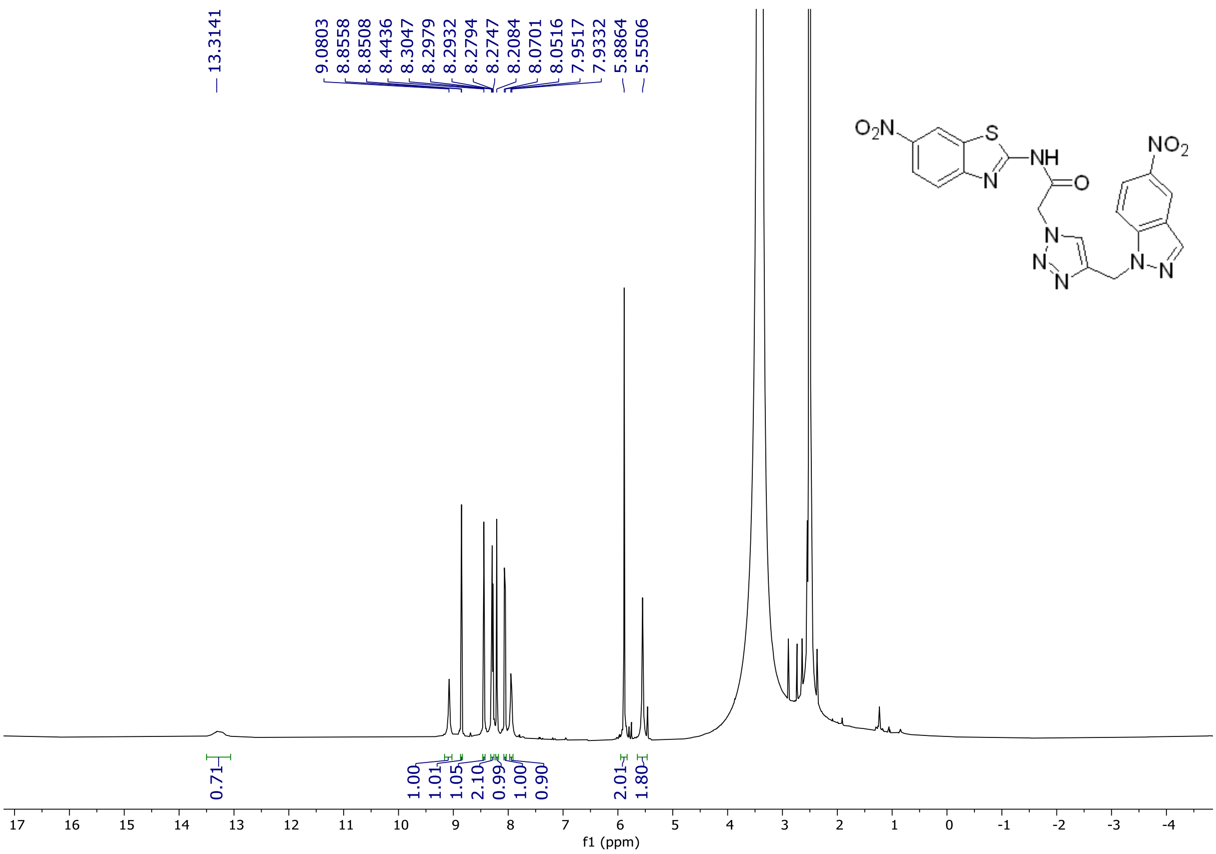

Figure S59. ^{1}H NMR spectrum of compound **5h** (500.13 MHz, DMSO-d_6).

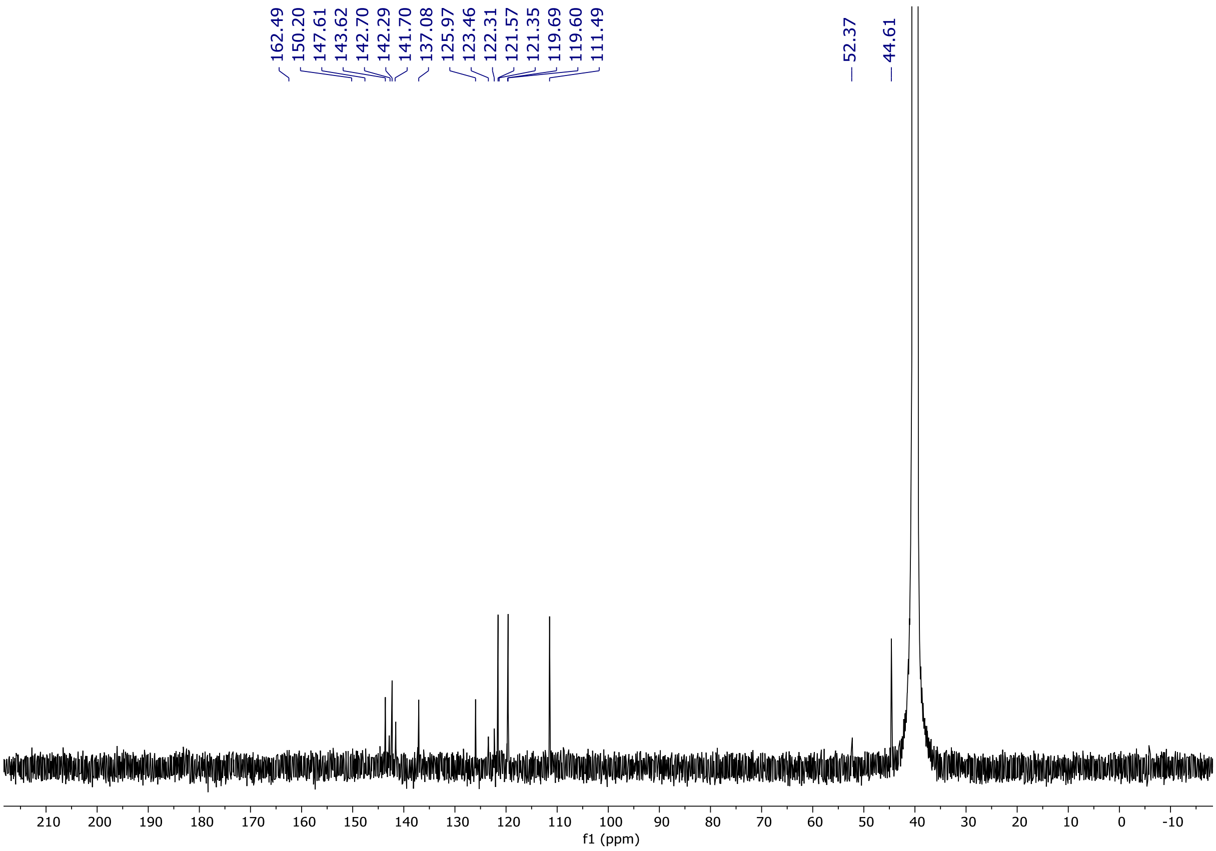

Figure S60. ^{13}C NMR spectrum of compound **5h** (125.76 MHz, DMSO-d_6).

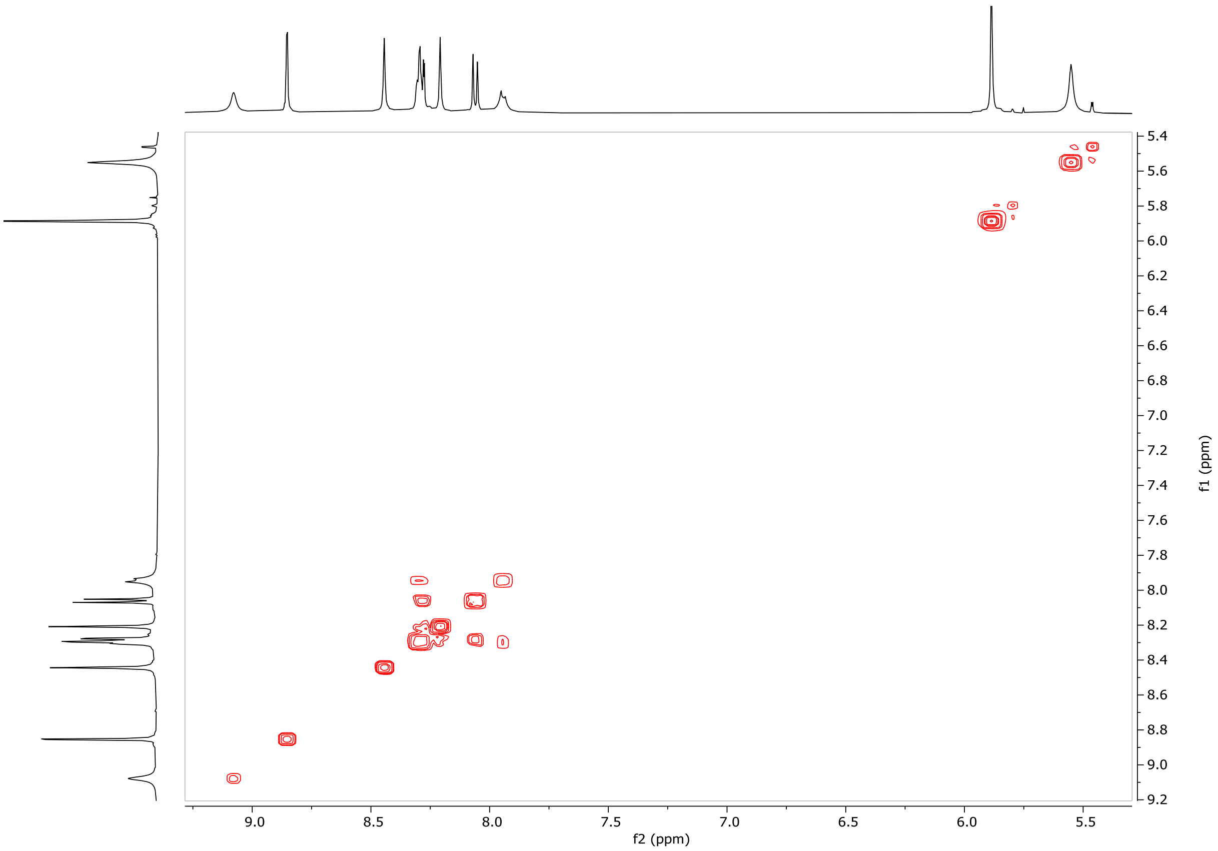

Figure S61. COSY($^1H/^1H$) spectrum of compound **5h** (500.13 MHz, DMSO-*d*$_6$).

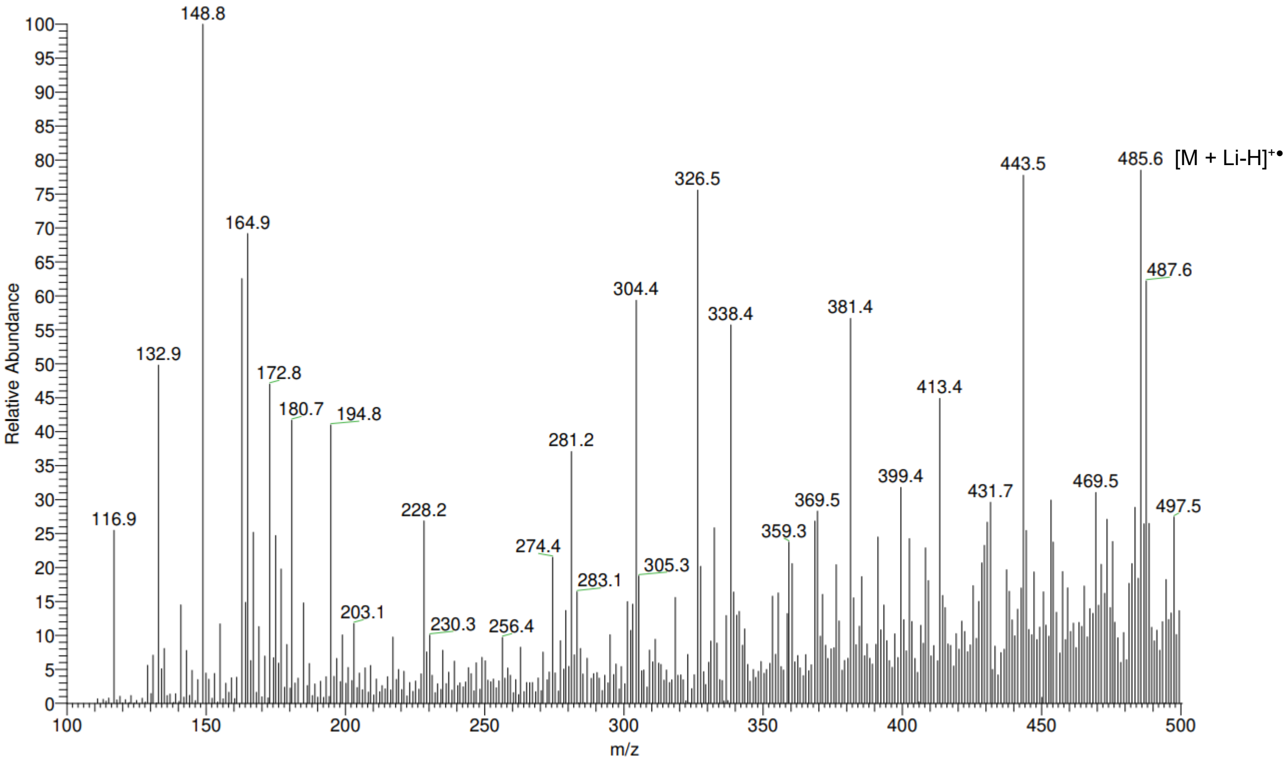

Figure S62. MS-ESI(+) spectrum of compound **5h**.

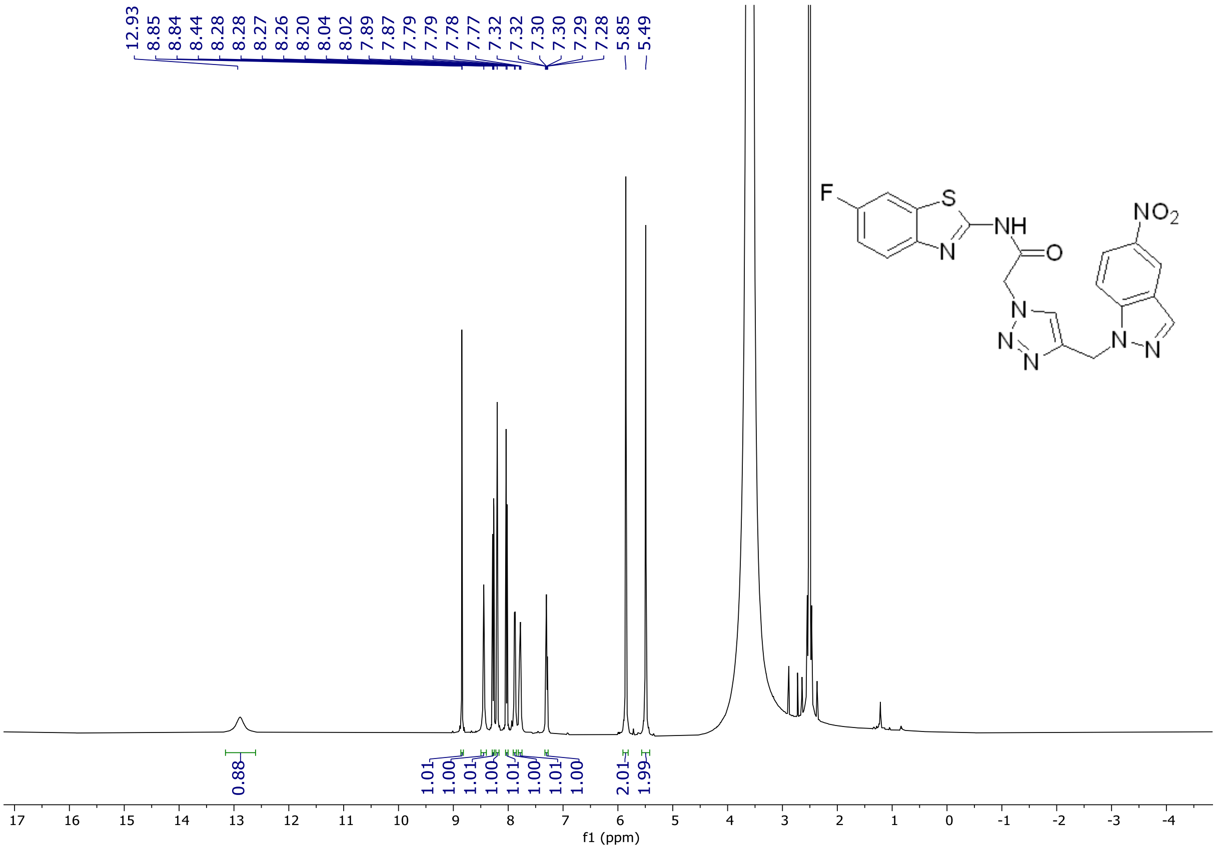

Figure S63. ^{1}H NMR spectrum of compound **5i** (500.13 MHz, DMSO-d_6).

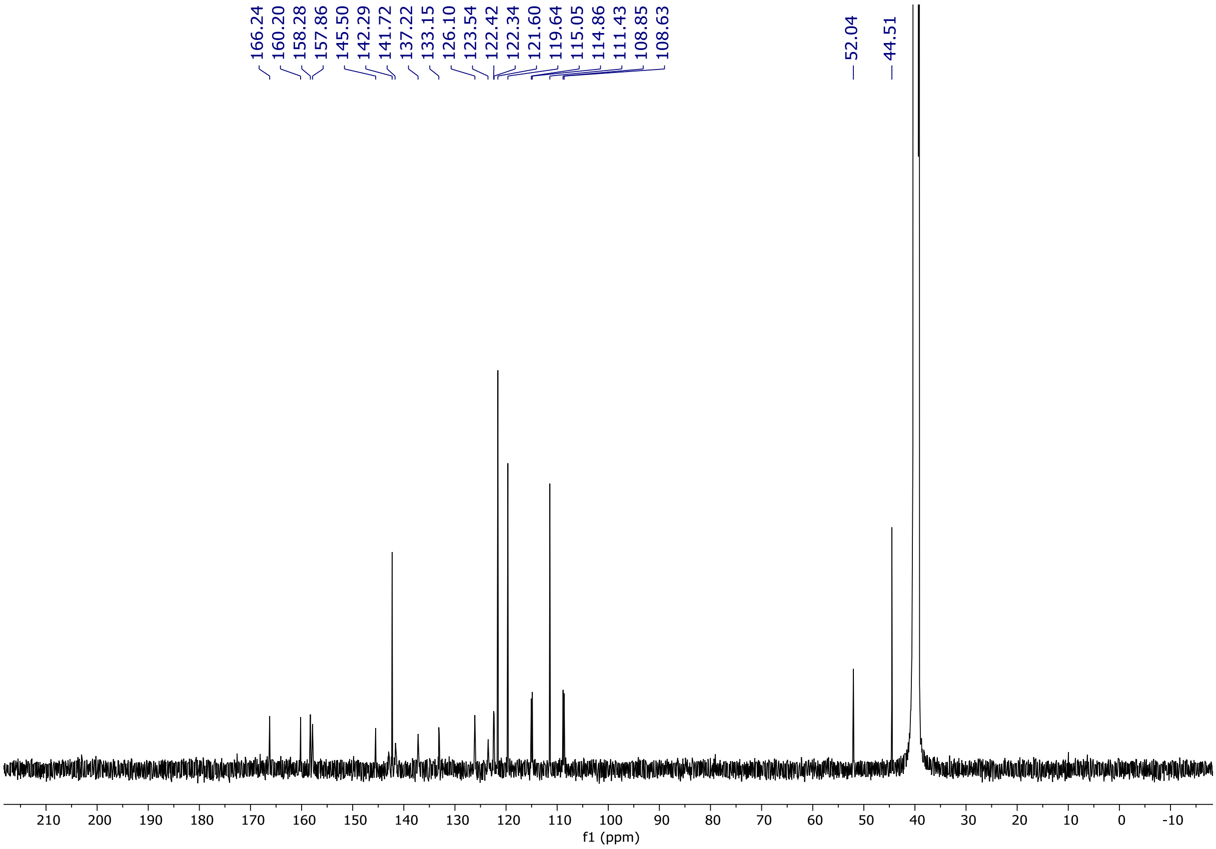

Figure S64. ^{13}C NMR spectrum of compound **5i** (125.76 MHz, DMSO-d_6).

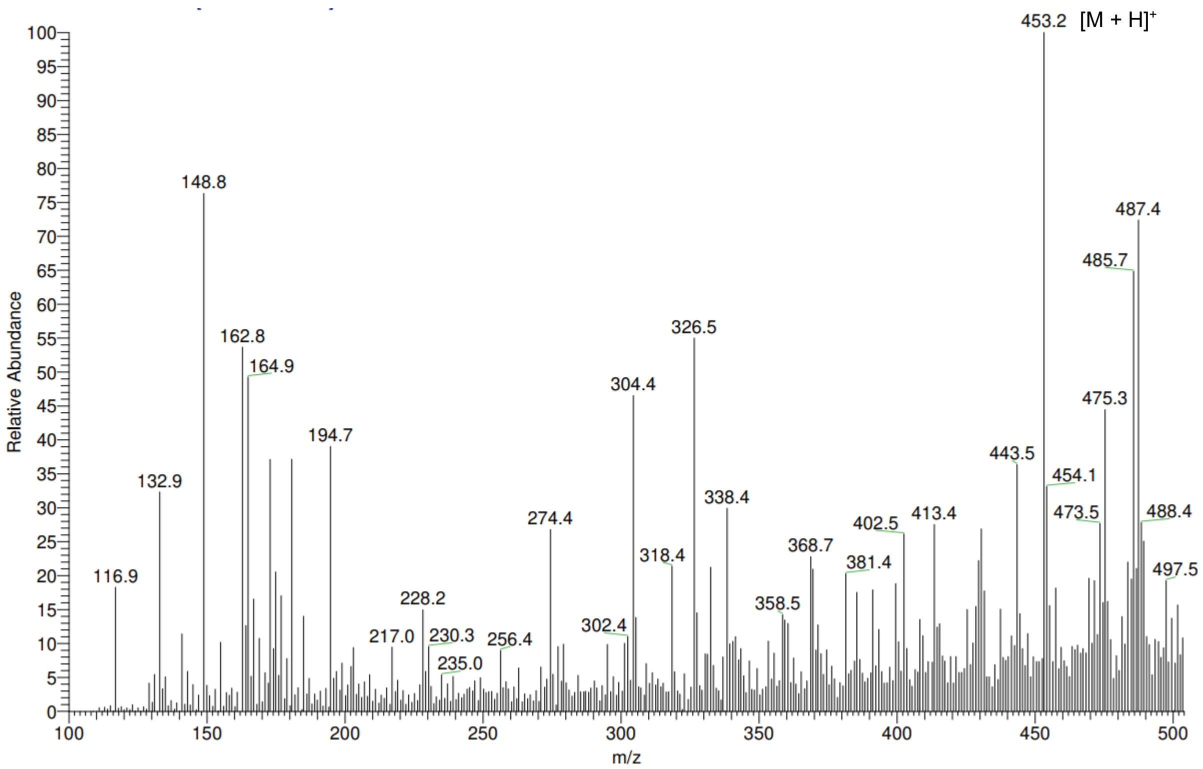

Figure S65. MS-ESI(+) spectrum of compound **5i**.

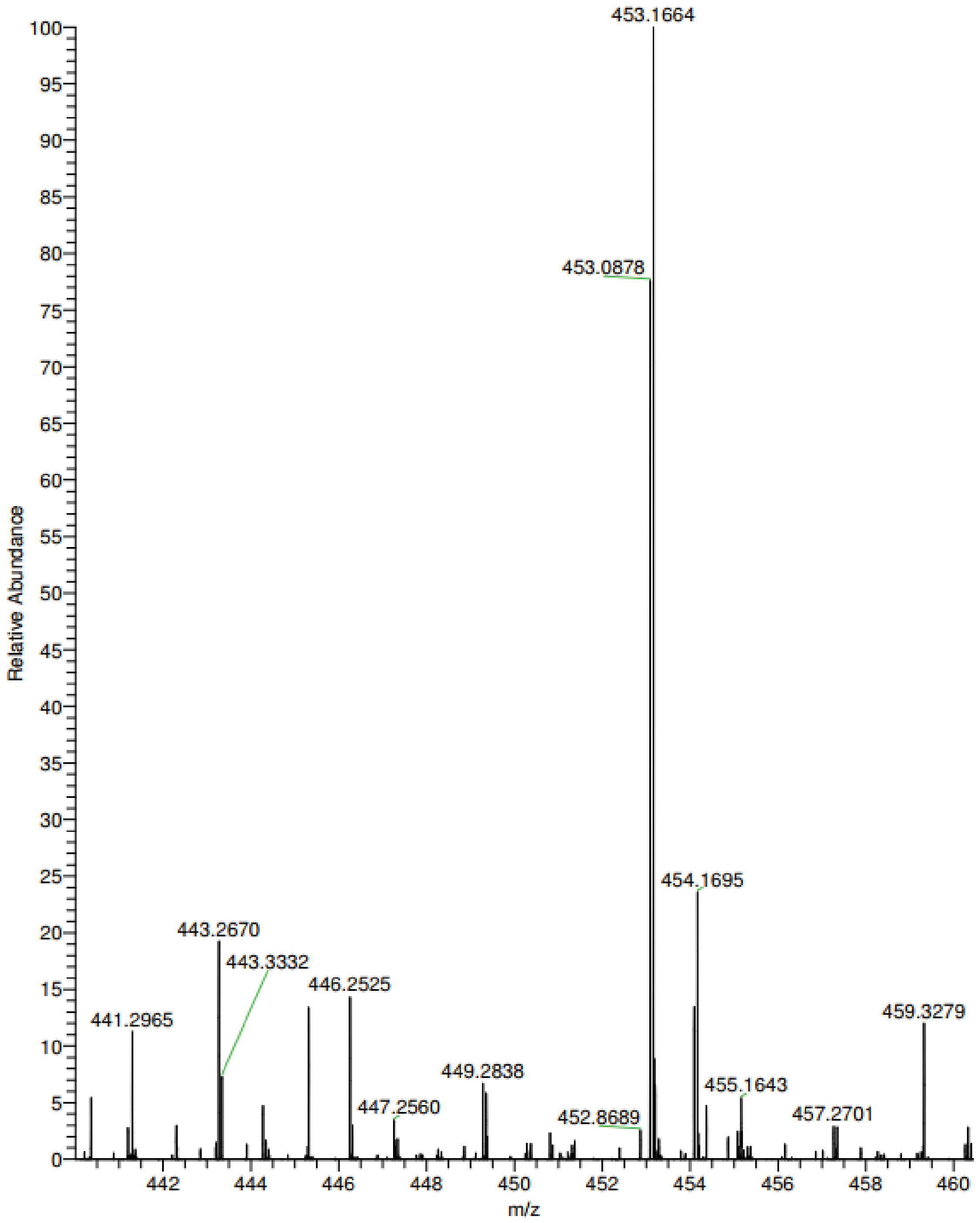

Figure S66. HRMS-ESI(+) spectrum of compound **5i**.

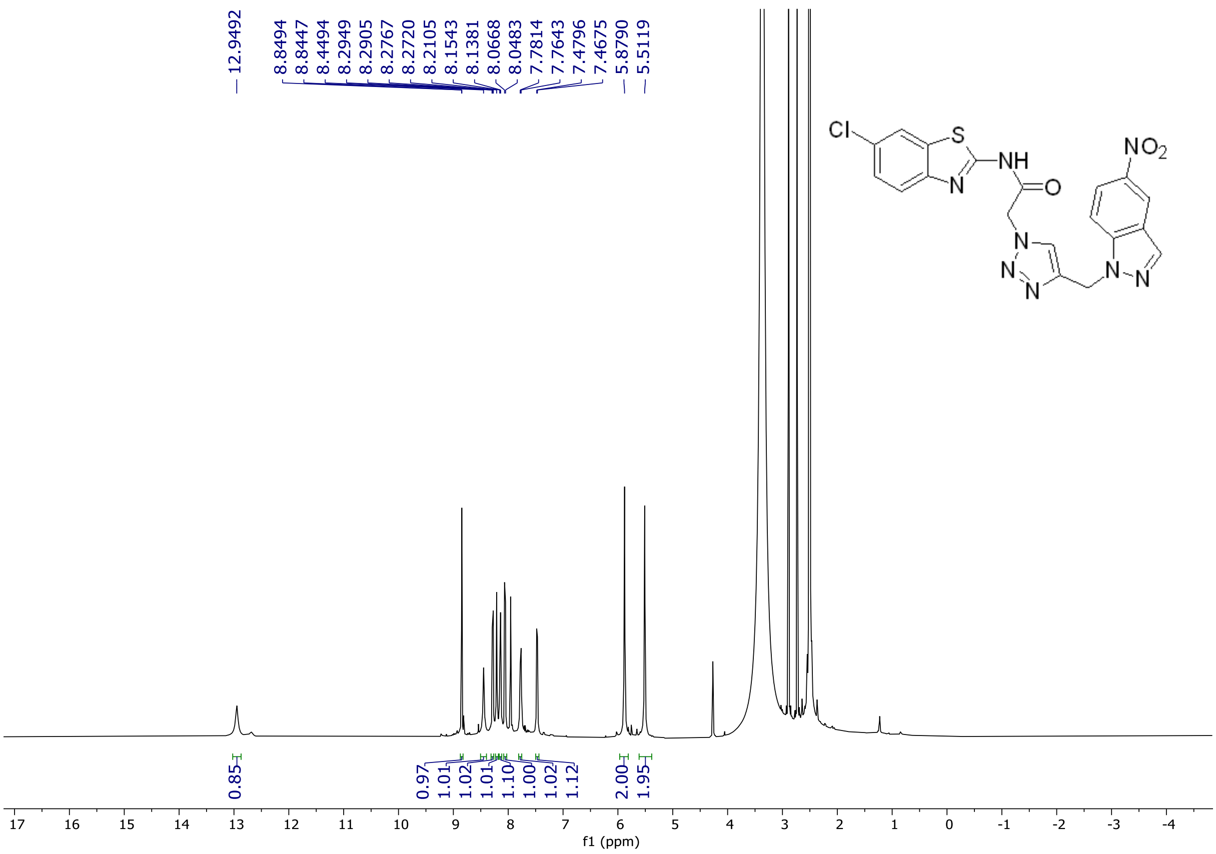

Figure S67. ^{1}H NMR spectrum of compound **5j** (500.13 MHz, DMSO-*d*$_6$).

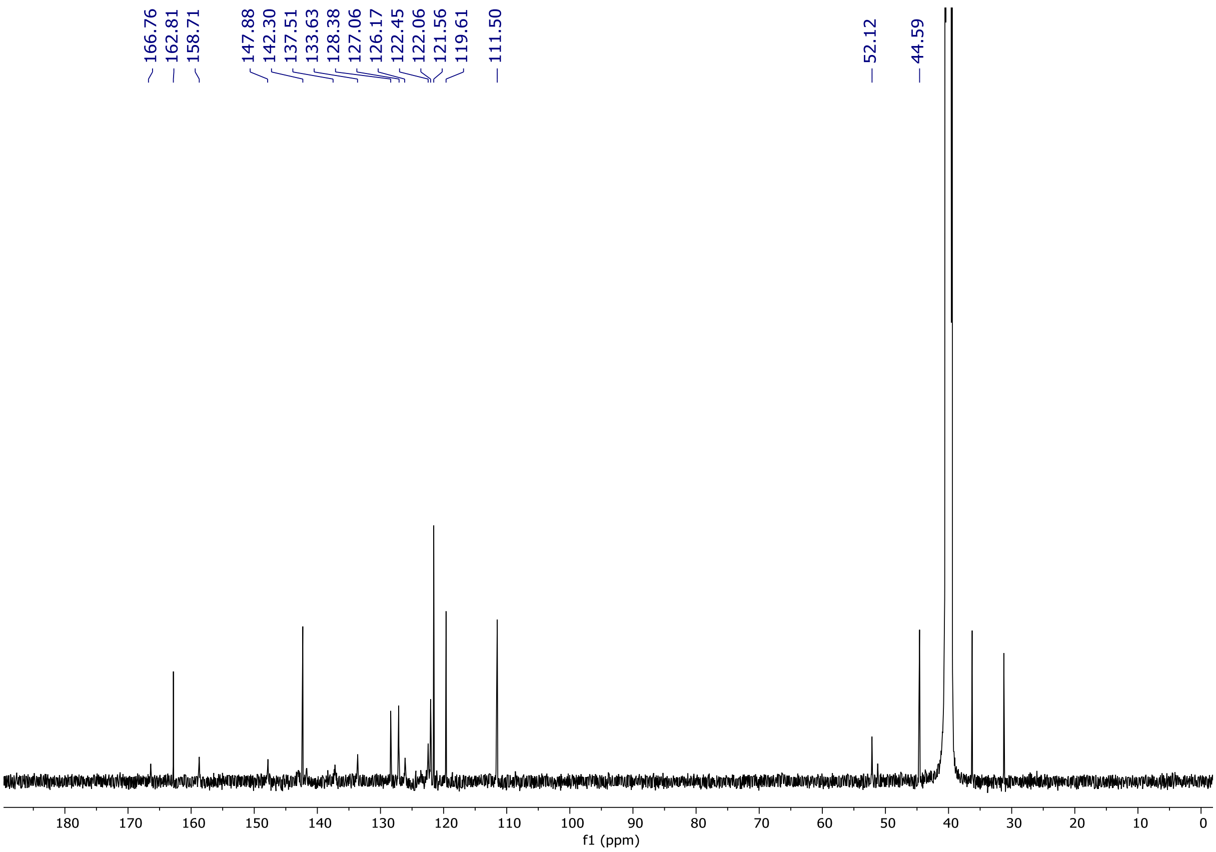

Figure S68. ^{13}C NMR spectrum of compound **5j** (125.76 MHz, DMSO-*d*$_6$).

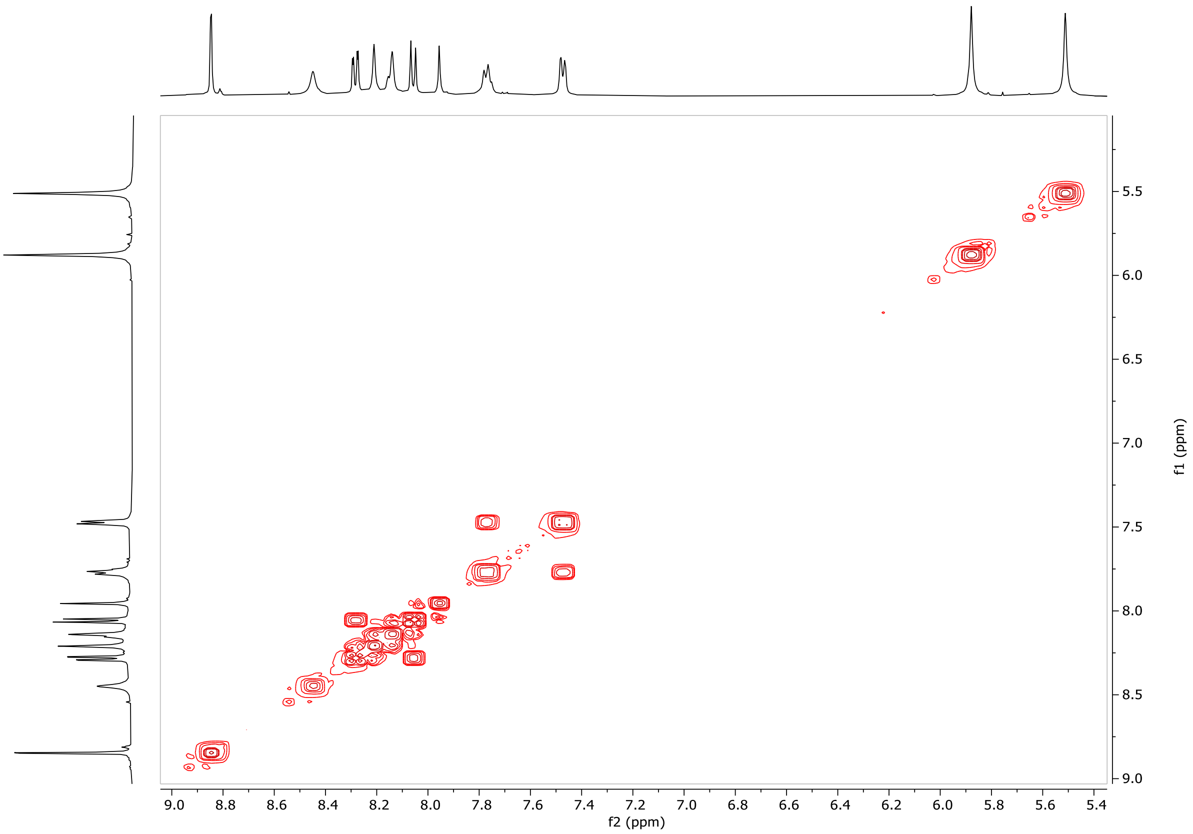

Figure S69. COSY($^1H/^1H$) spectrum of compound **5j** (500.13 MHz, DMSO-d_6).

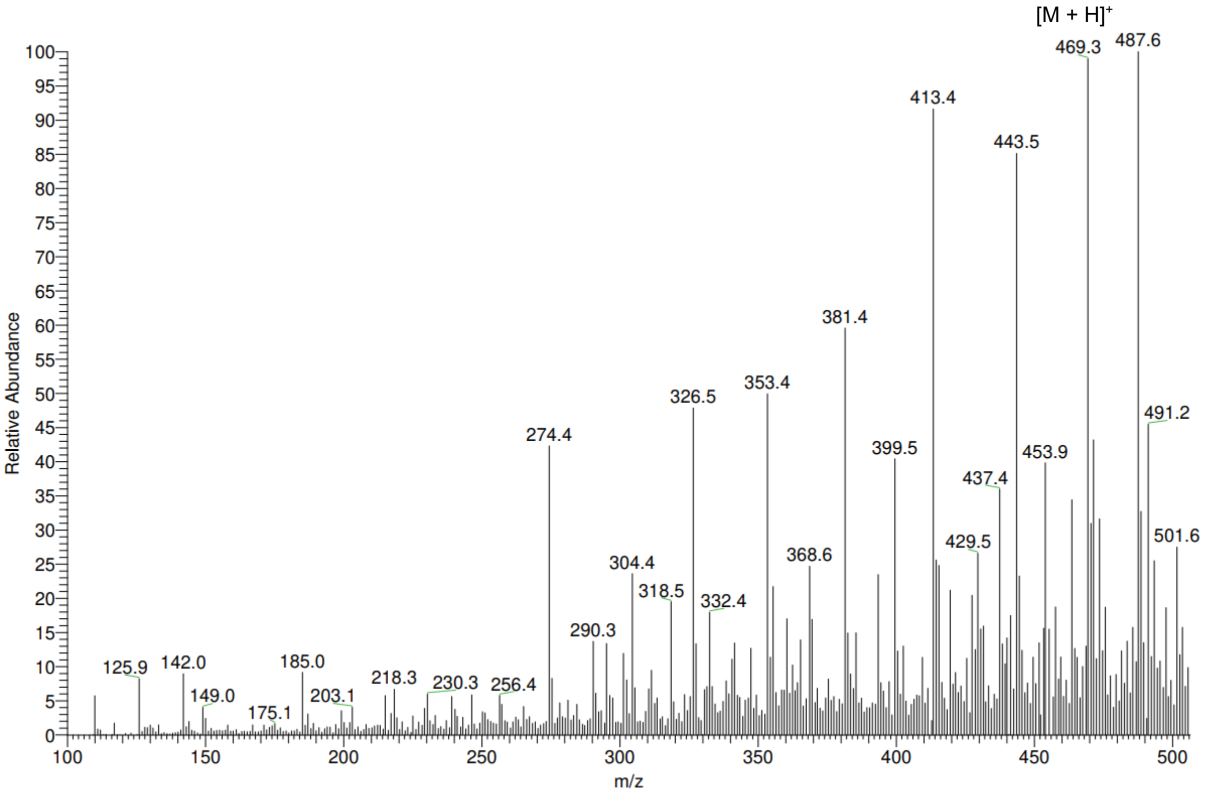

Figure S70. MS-ESI(+) spectrum of compound **5j**.

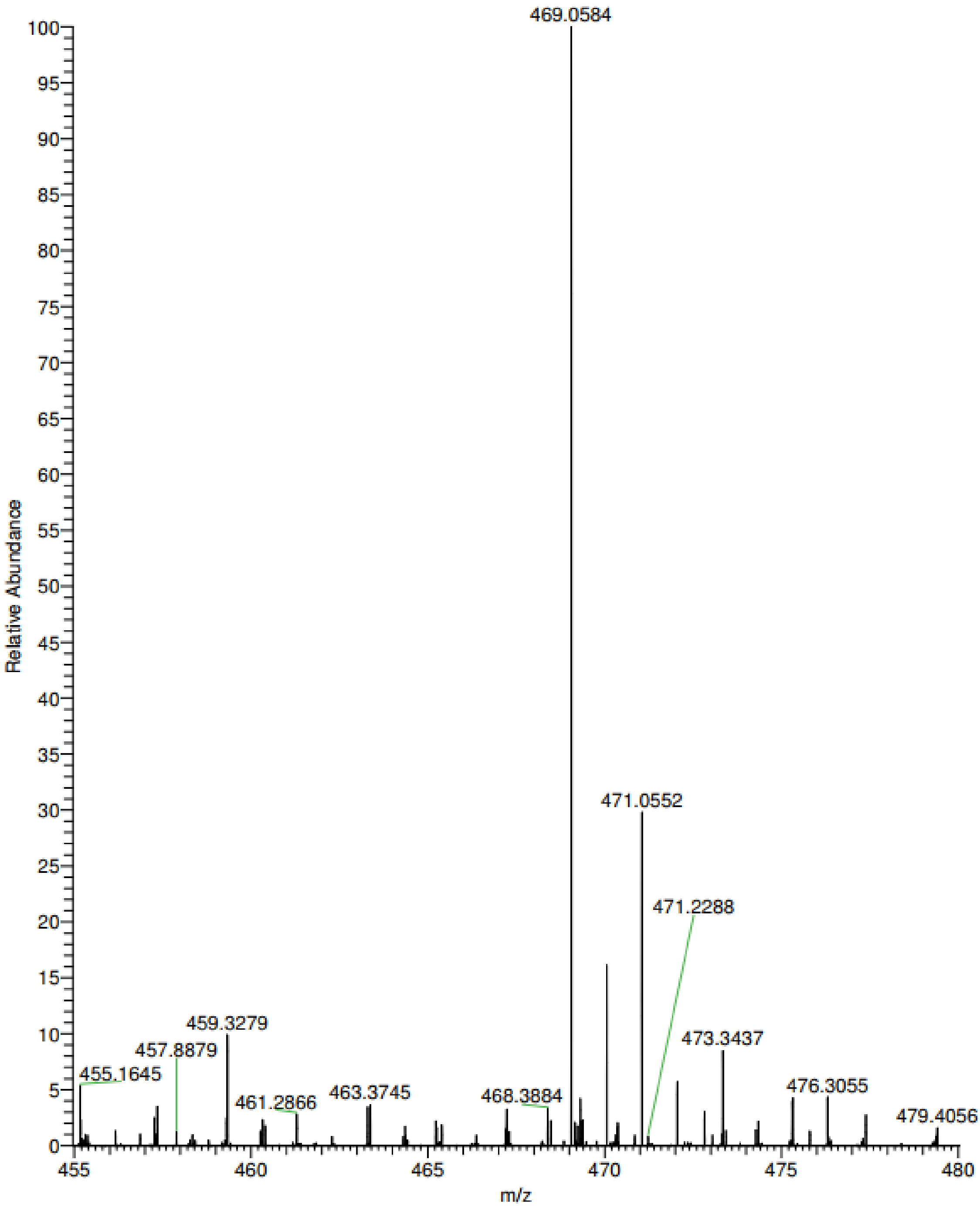

Figure S71. HRMS-ESI(+) spectrum of compound **5j**.

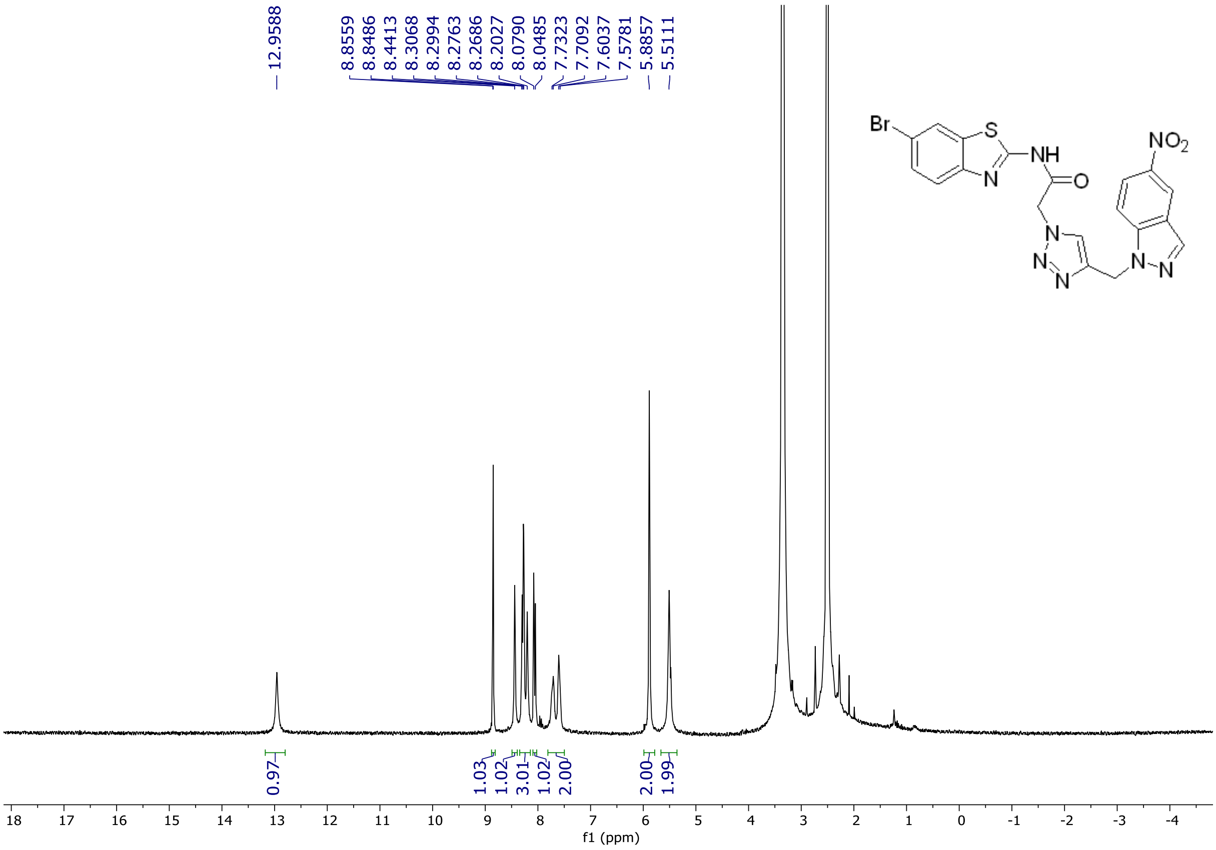

Figure S72. ^{1}H NMR spectrum of compound **5k** (300.13 MHz, DMSO-*d*$_6$).

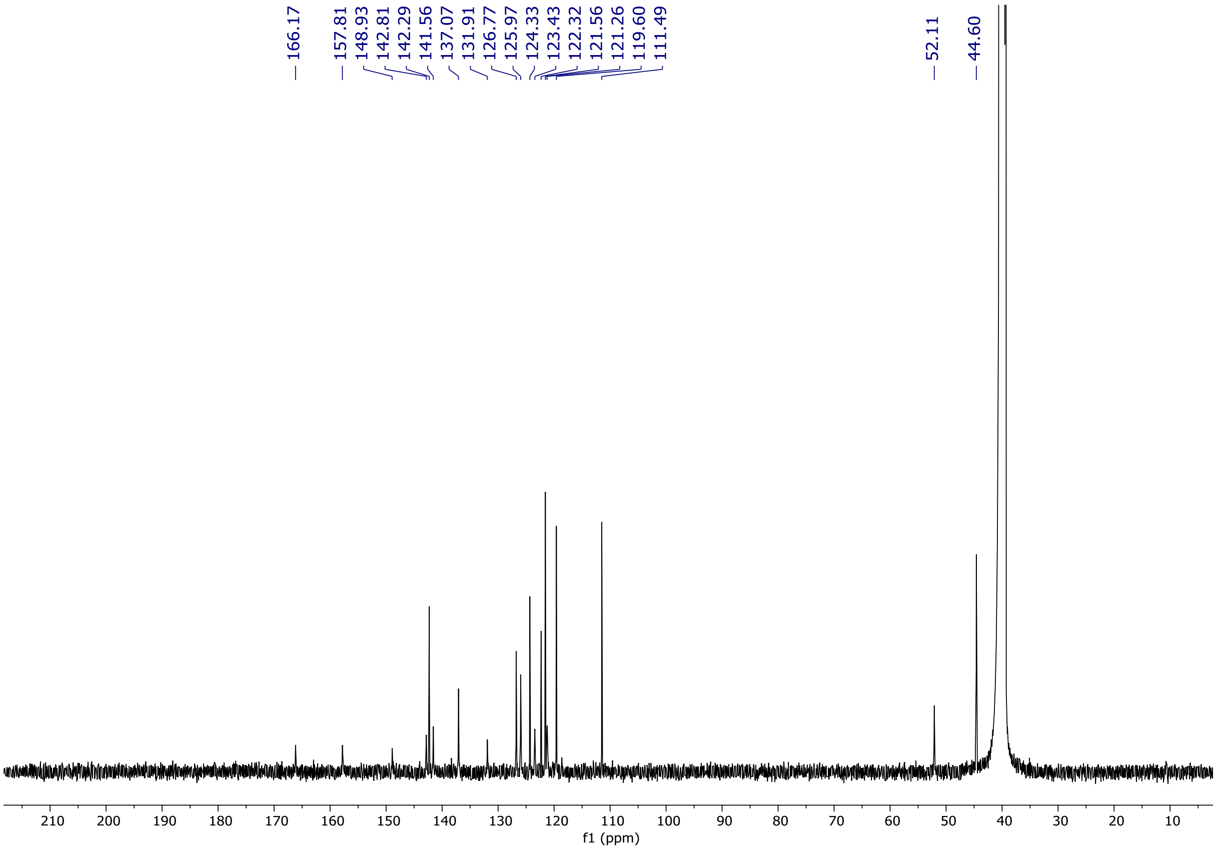

Figure S73. ^{13}C NMR spectrum of compound **5k** (125.76 MHz, DMSO-*d*$_6$).

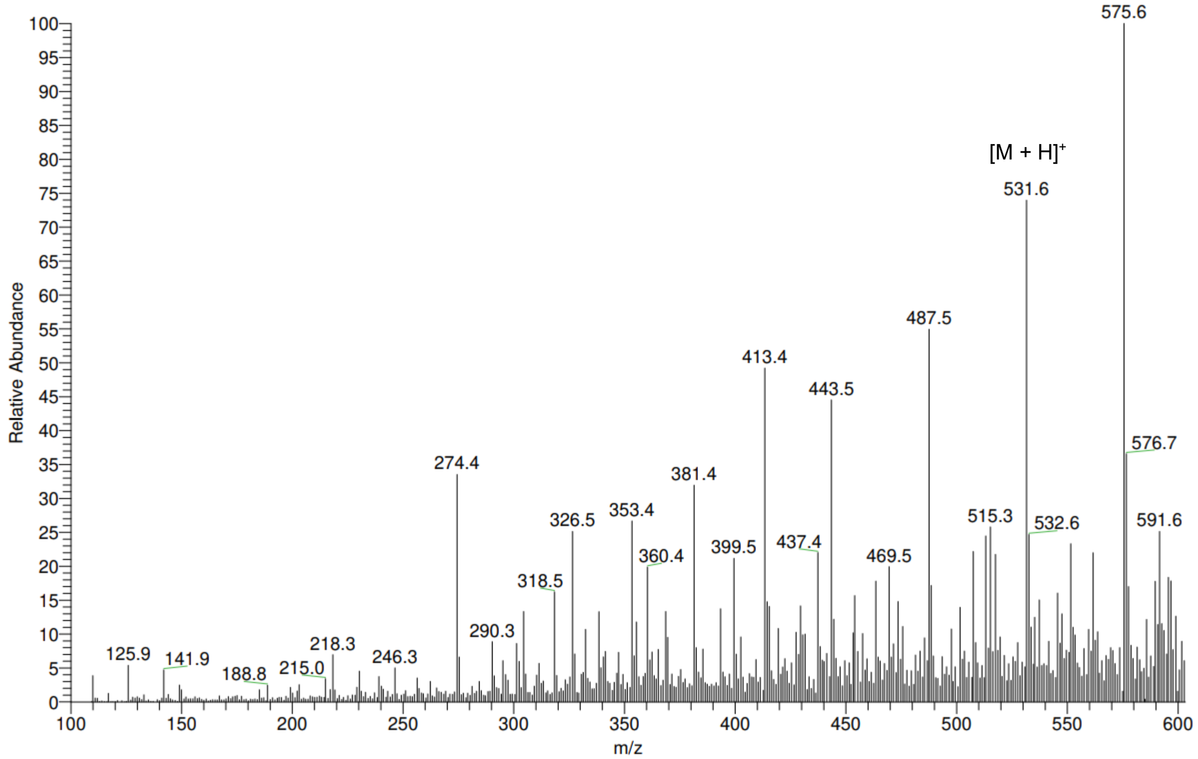

Figure S74. MS-ESI(+) spectrum of compound **5k**.

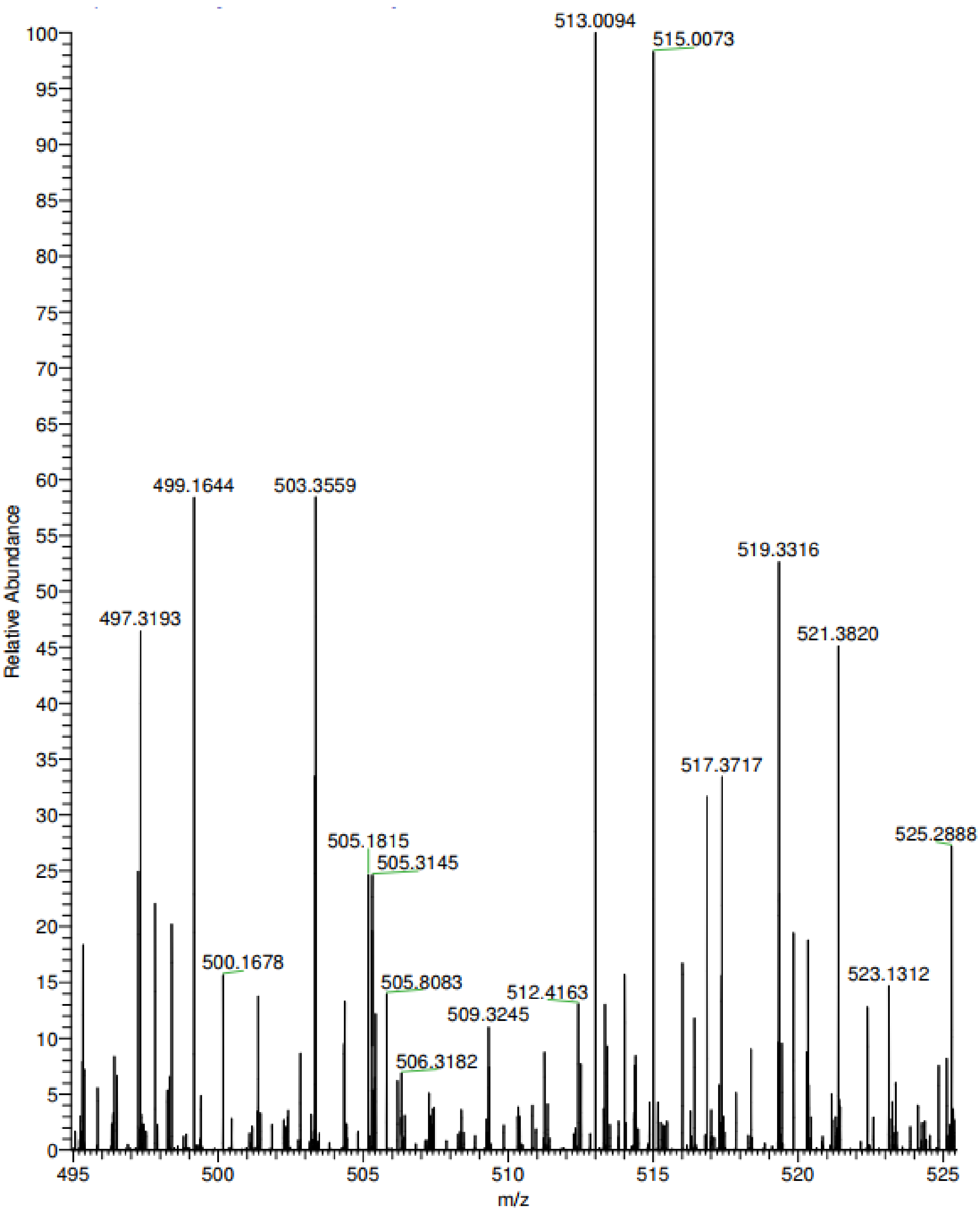

Figure S75. HRMS-ESI(+) spectrum of compound **5k**.

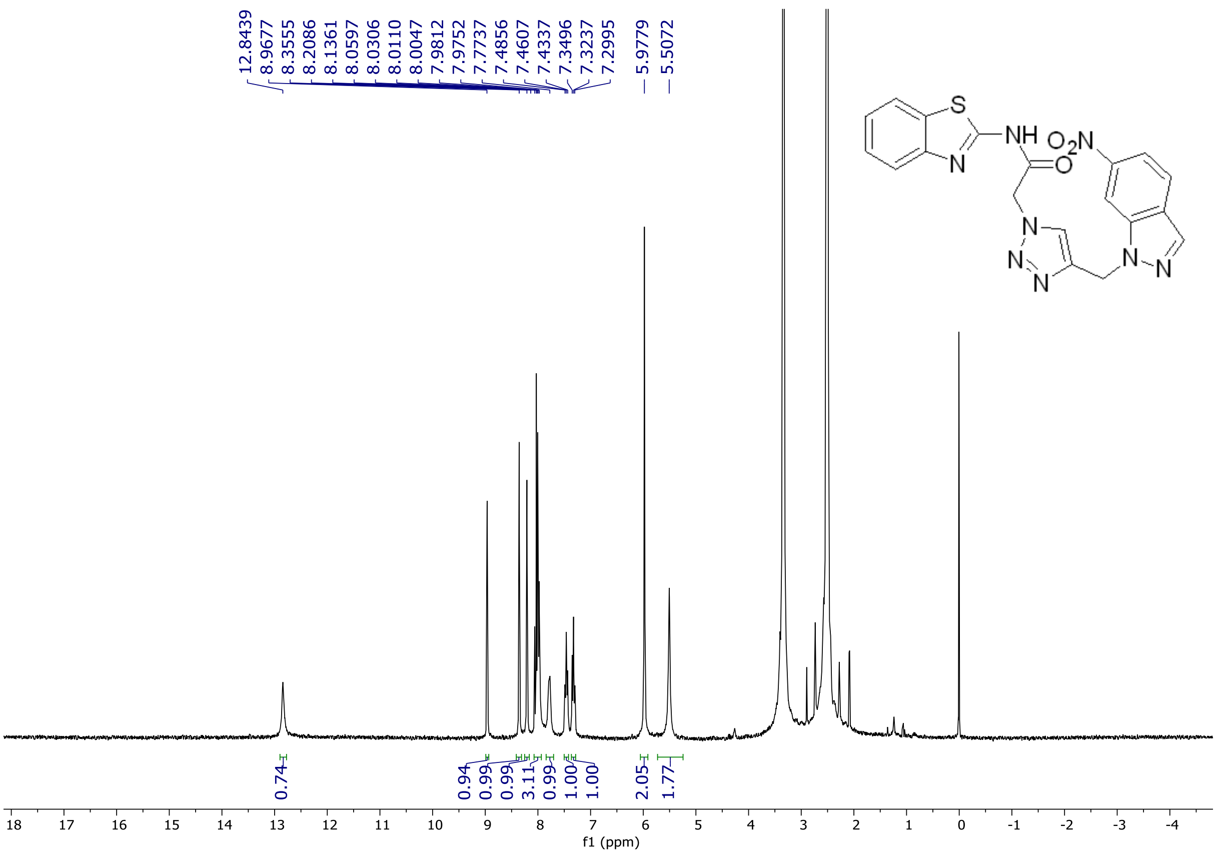

Figure S76. ^{1}H NMR spectrum of compound **5l** (300.13 MHz, DMSO-d_6).

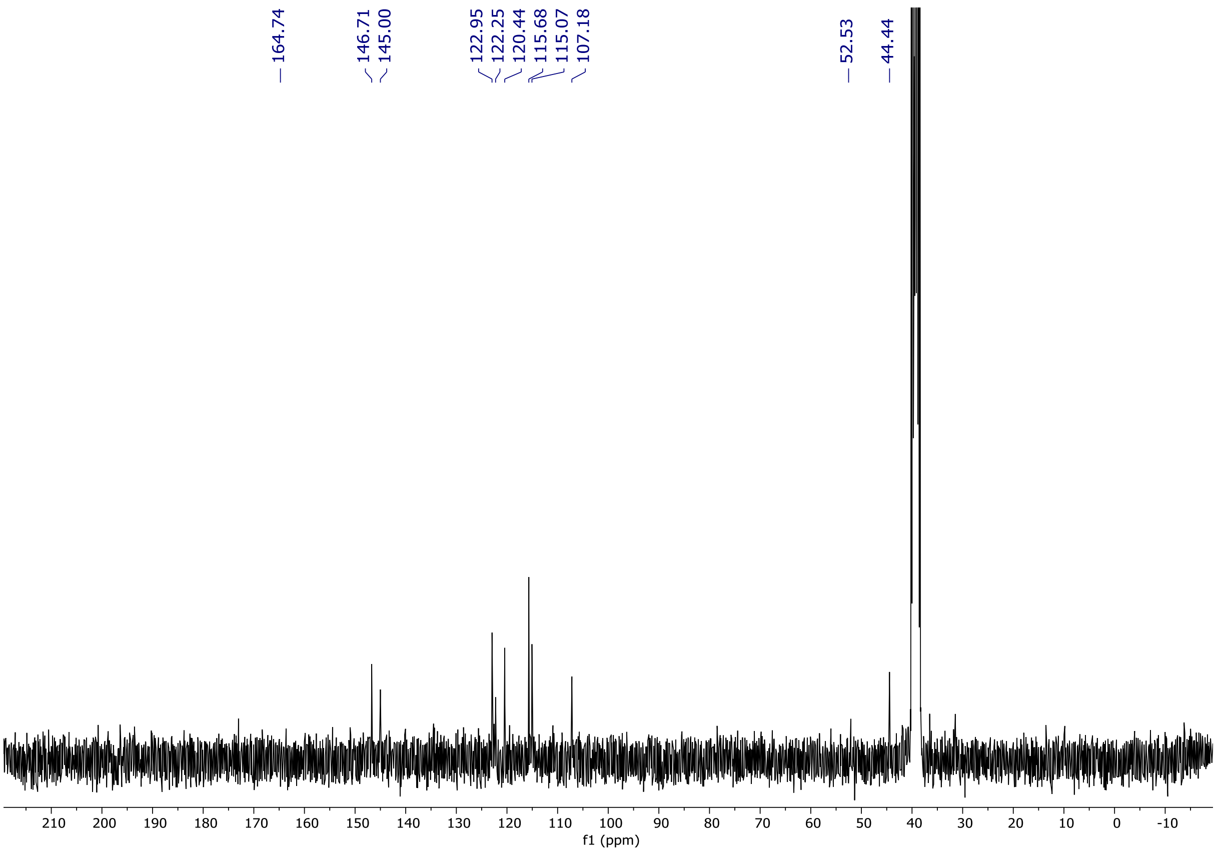

Figure S77. ^{13}C NMR spectrum of compound **5l** (75.47 MHz, DMSO-d_6).

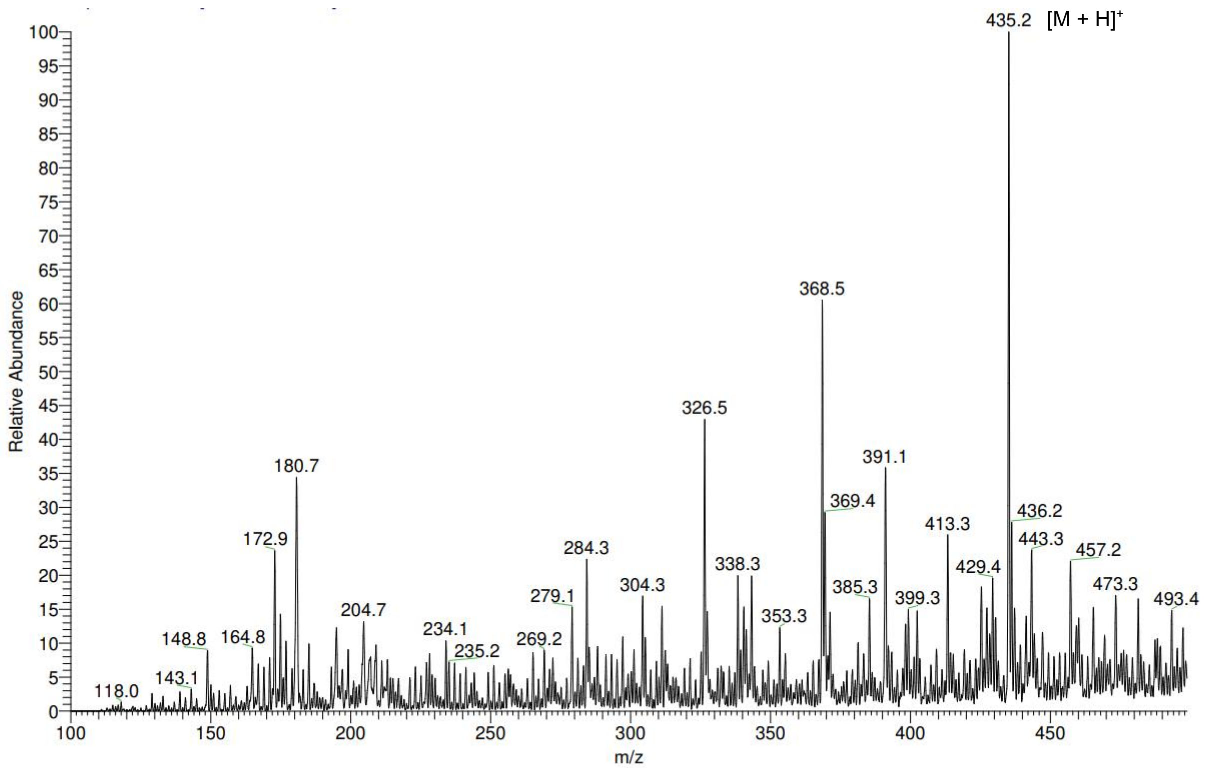

Figure S78. MS-ESI(+) spectrum of compound **5l**.

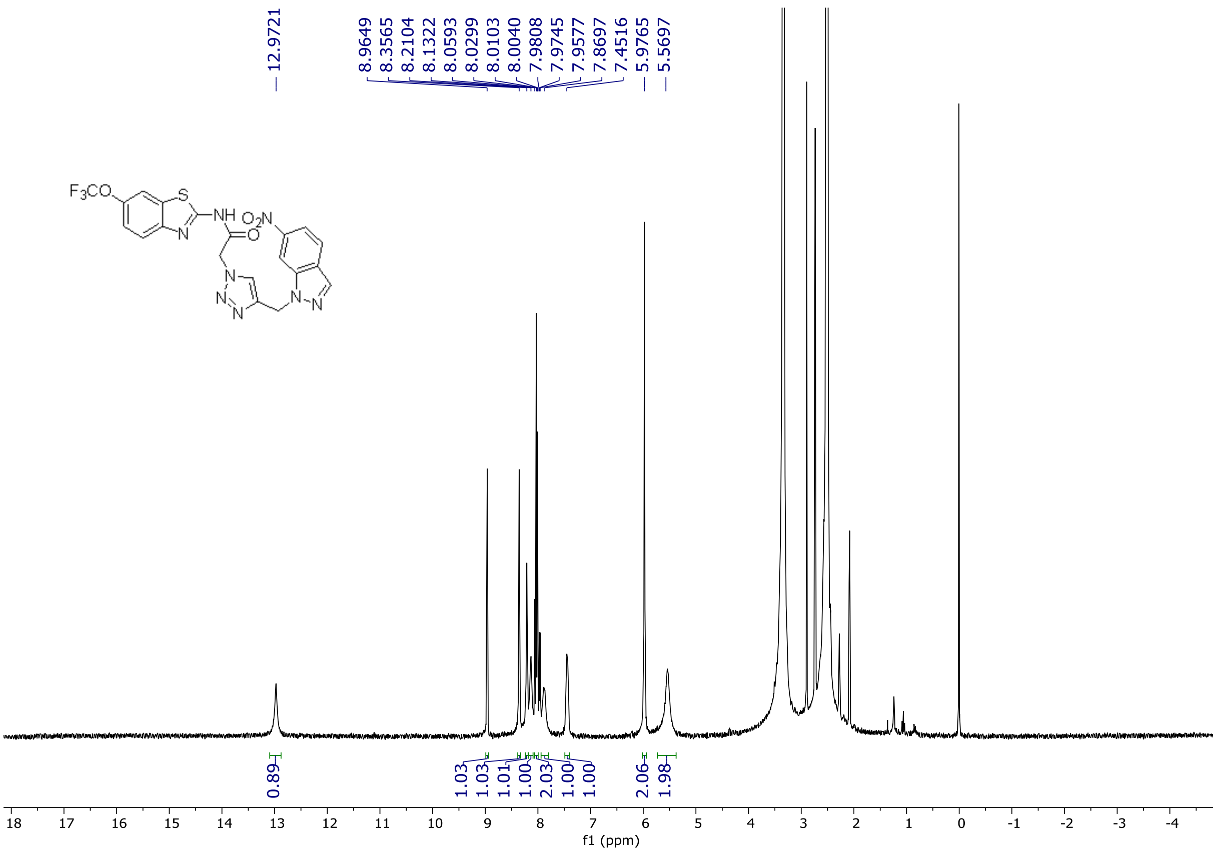

Figure S79. [11]H NMR spectrum of compound **5m** (300.13 MHz, DMSO-d_6).

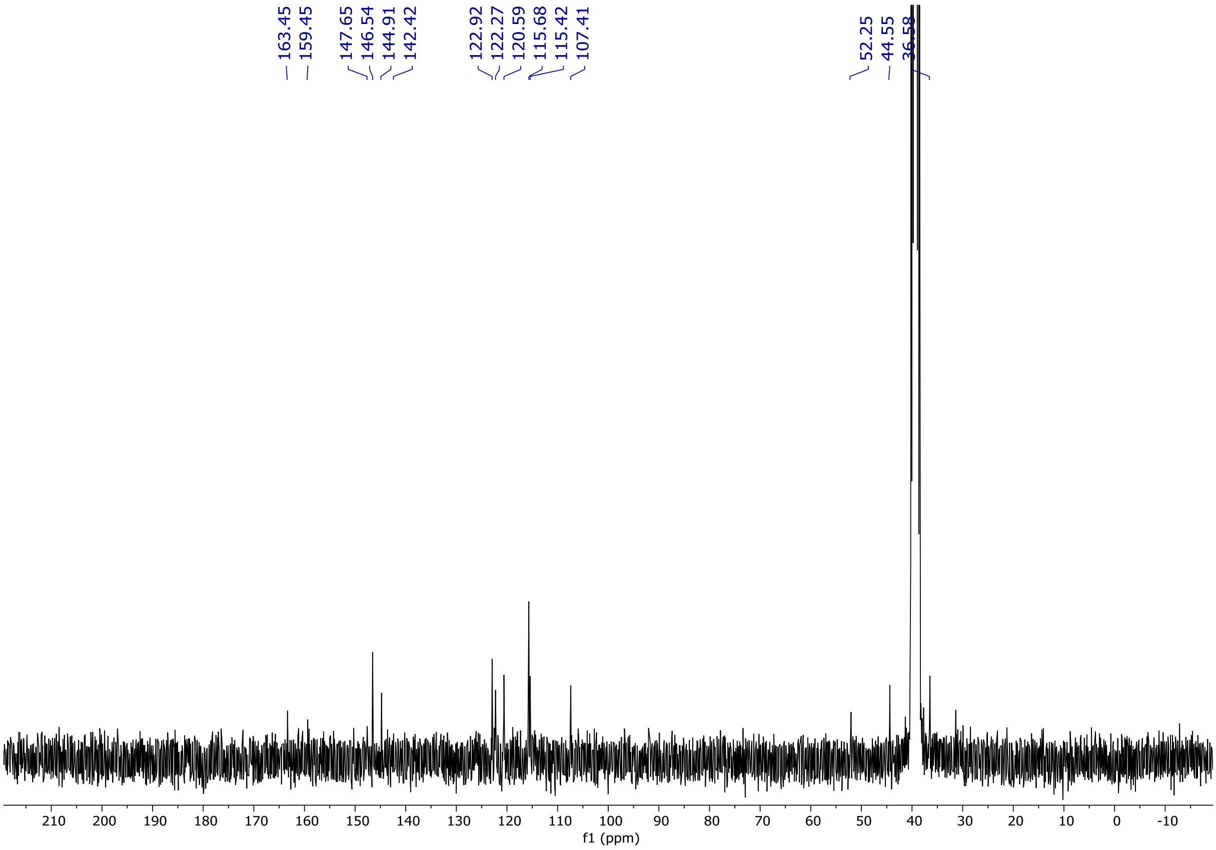

Figure S80. ^{13}C NMR spectrum of compound **5m** (75.47 MHz, DMSO-d_6).

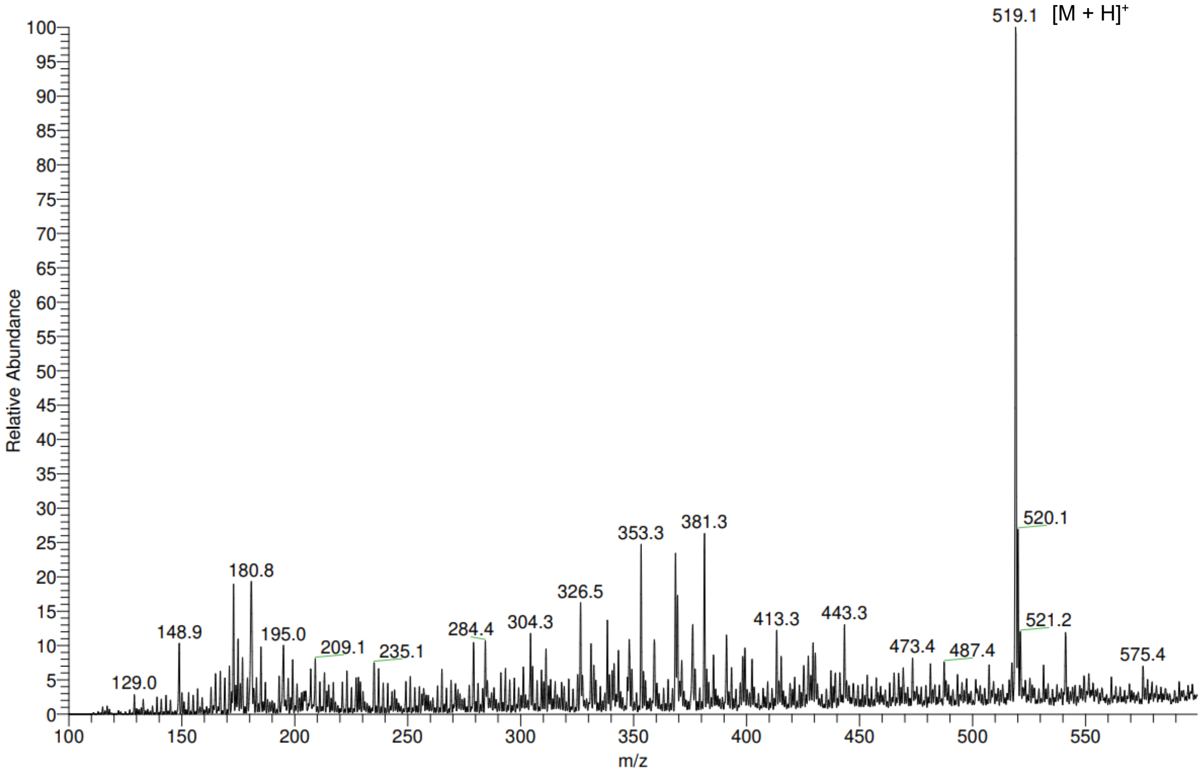

Figure S81. MS-ESI(+) spectrum of compound **5m**.

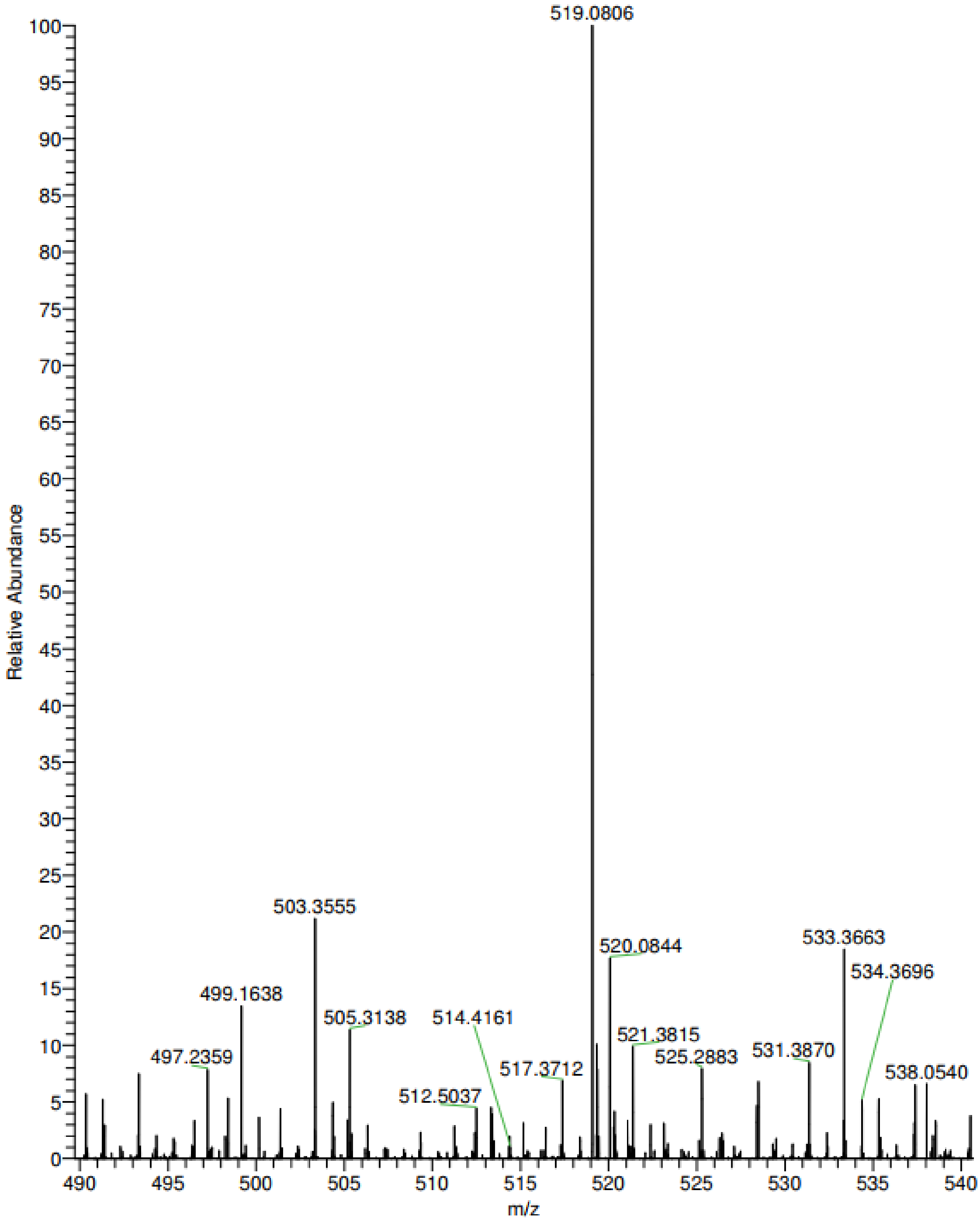

Figure S82. HRMS-ESI(+) spectrum of compound **5m**.

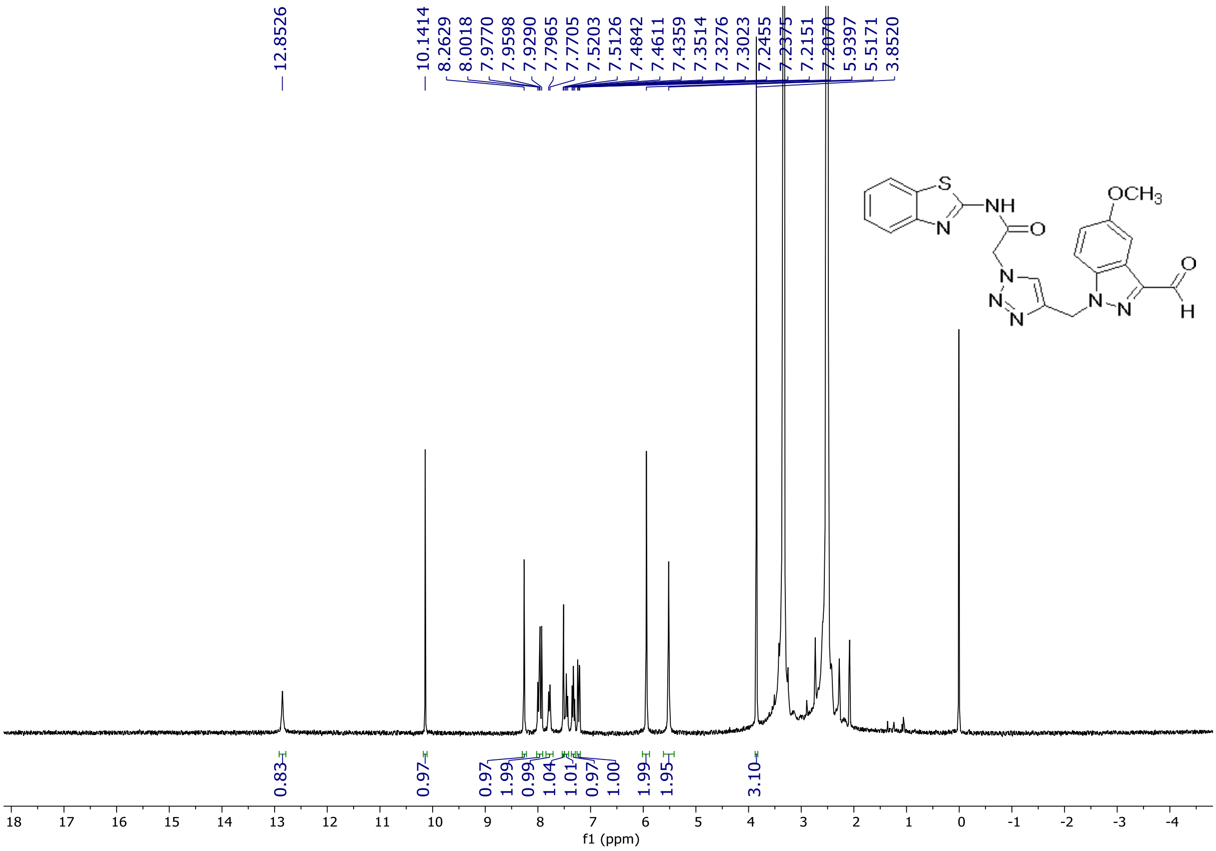

Figure S83. ^{1}H NMR spectrum of compound **5n** (300.13 MHz, DMSO-d_6).

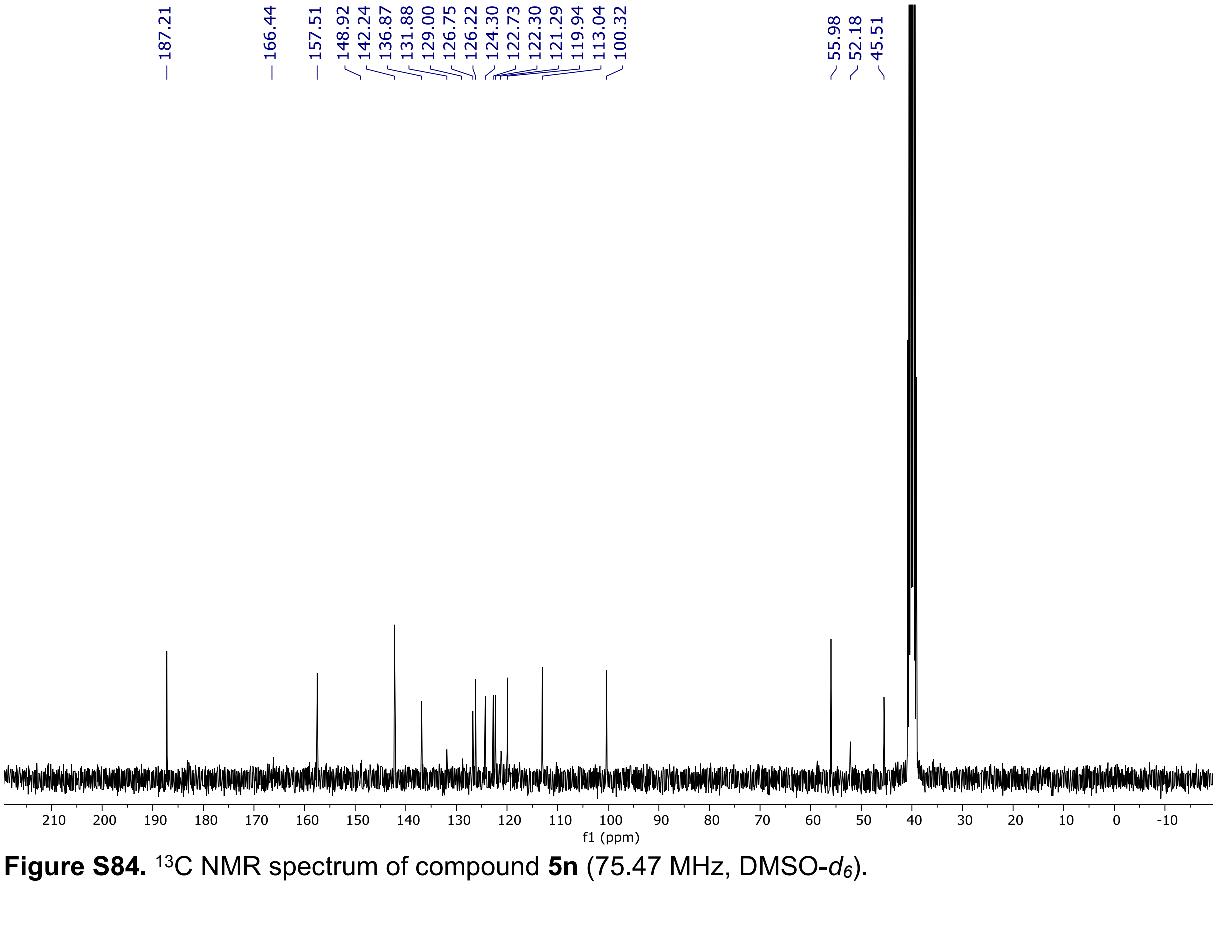

Figure S84. ^{13}C NMR spectrum of compound **5n** (75.47 MHz, DMSO-d_6).

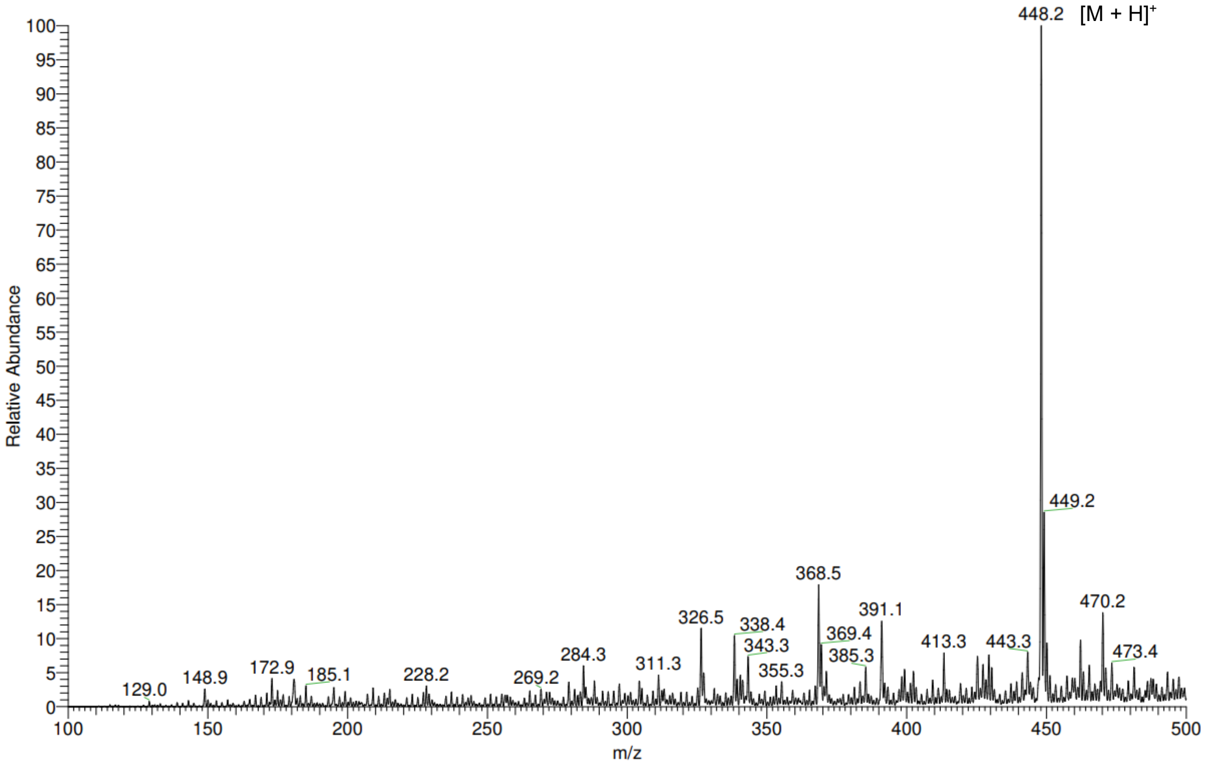

Figure S85. MS-ESI(+) spectrum of compound **5n**.

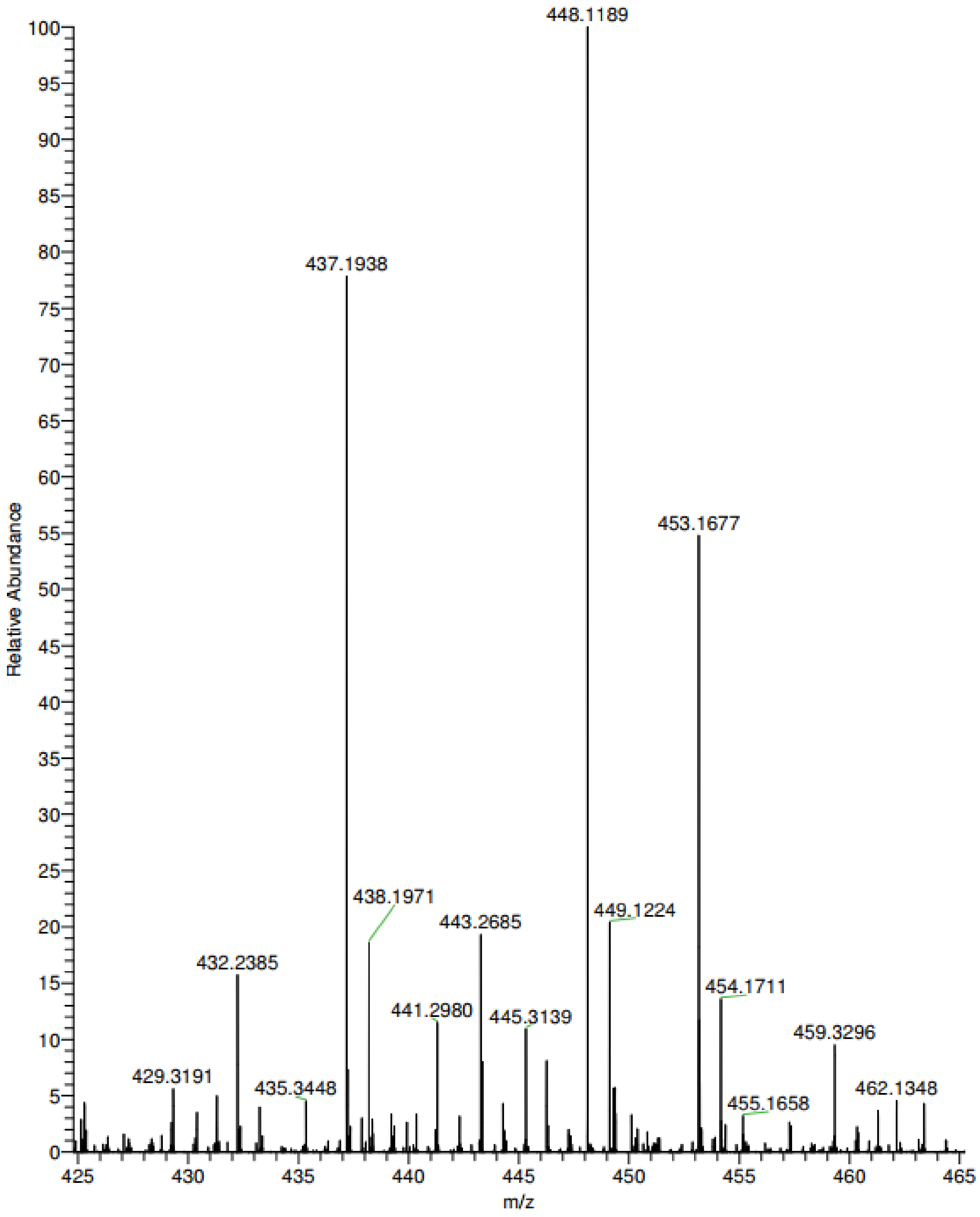

Figure S86. HRMS-ESI(+) spectrum of compound **5n**.

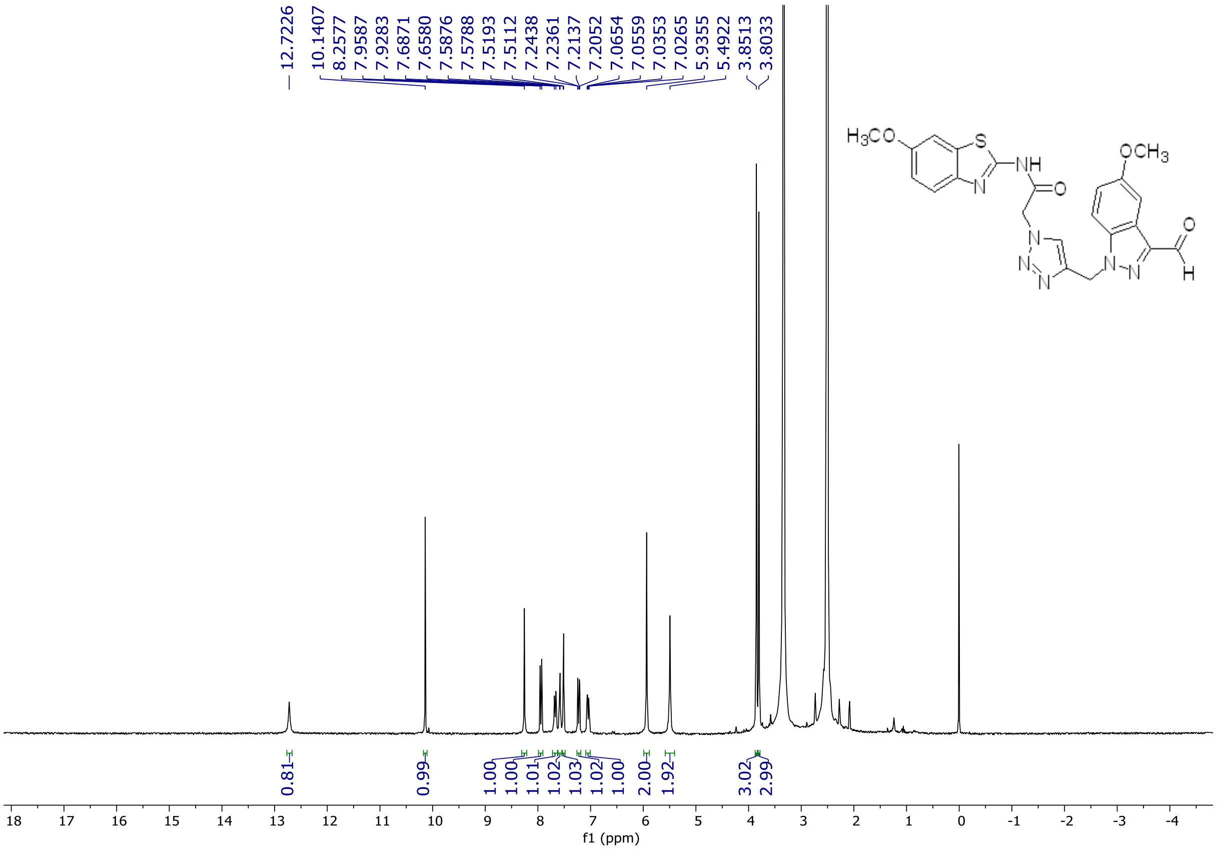

Figure S87. ^{1}H NMR spectrum of compound **5o** (300.13 MHz, DMSO-d_6).

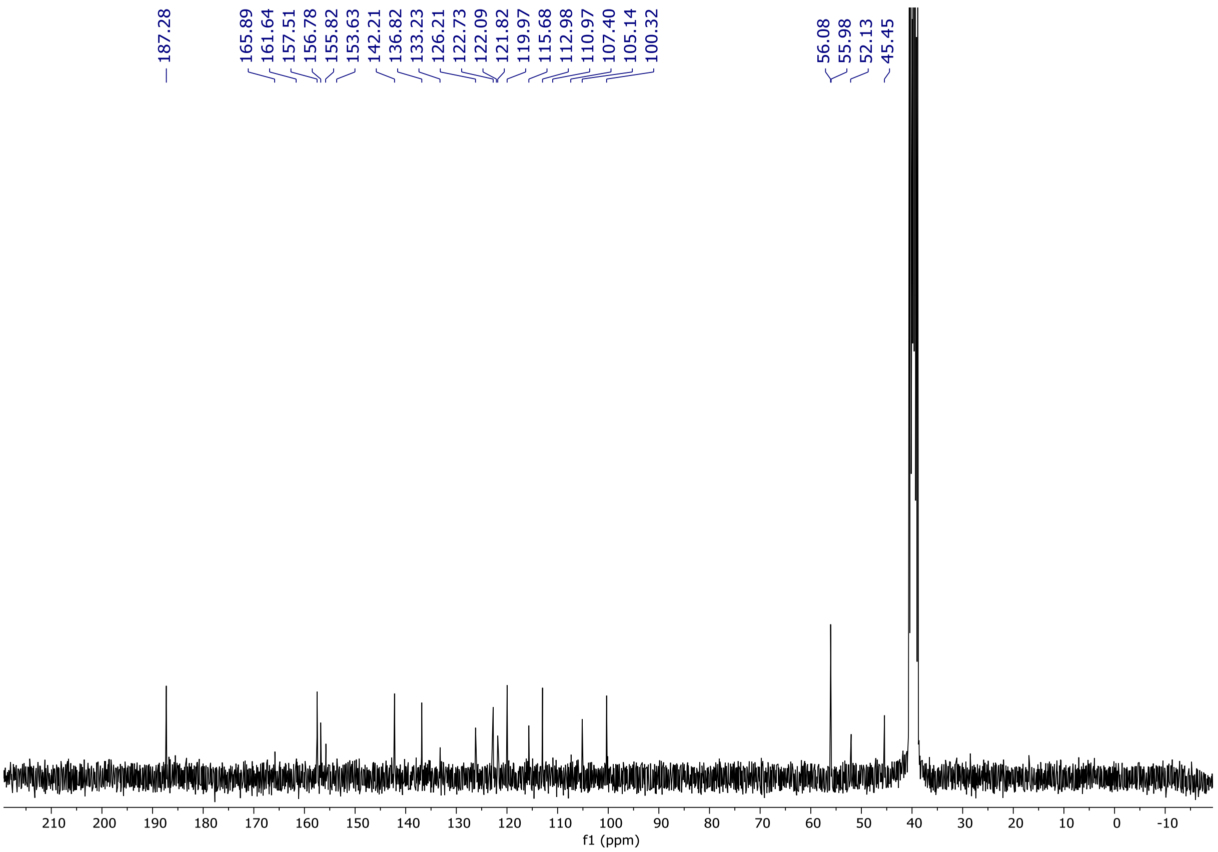

Figure S88. ^{13}C NMR spectrum of compound **5o** (75.47 MHz, DMSO-d_6).

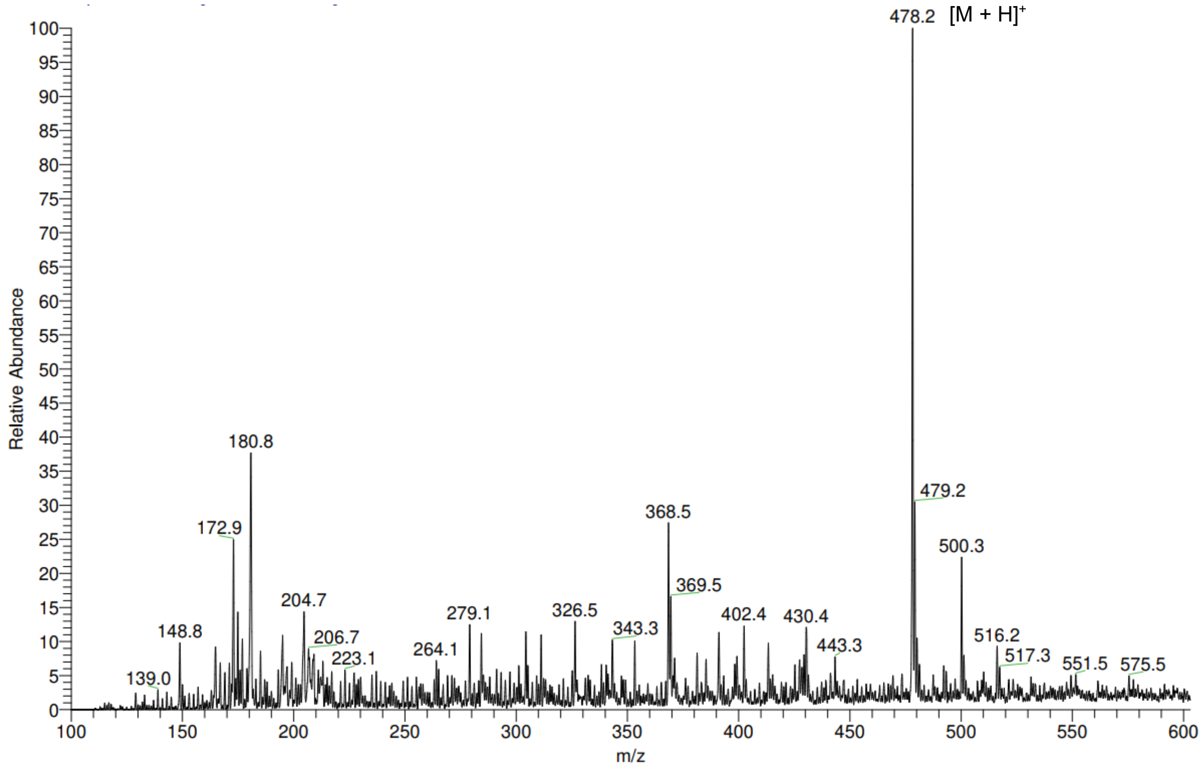

Figure S89. MS-ESI(+) spectrum of compound **5o**.

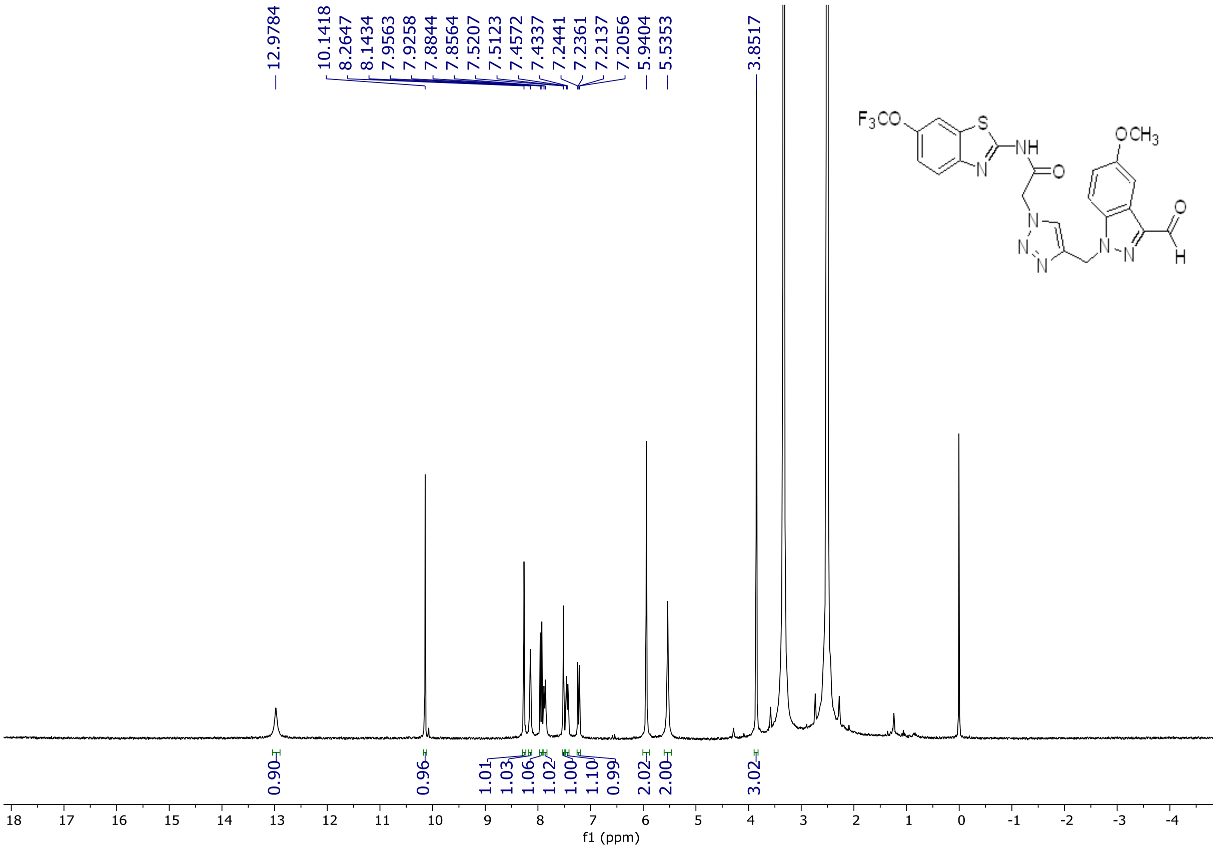

Figure S90. ^{1}H NMR spectrum of compound **5p** (300.13 MHz, DMSO-d_6).

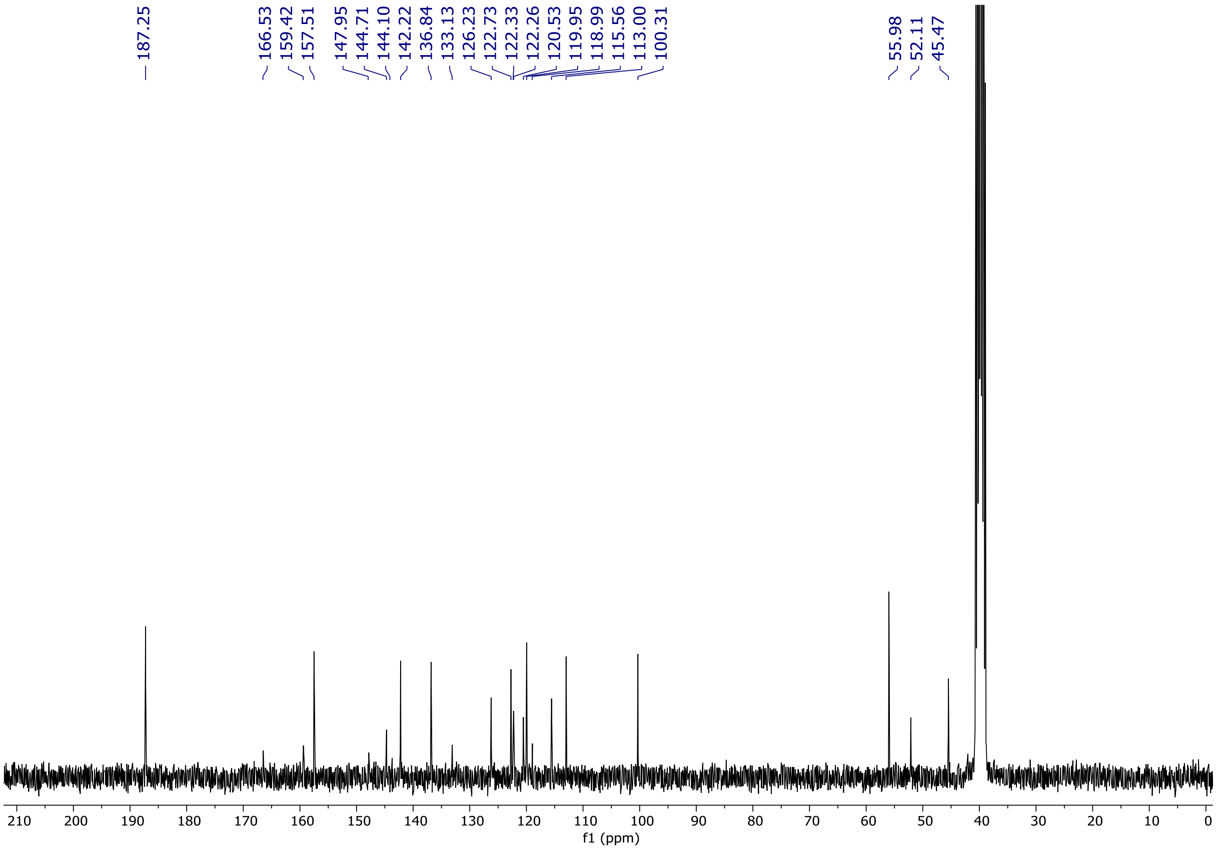

Figure S91. ^{13}C NMR spectrum of compound **5p** (75.47 MHz, DMSO-*d*$_6$).

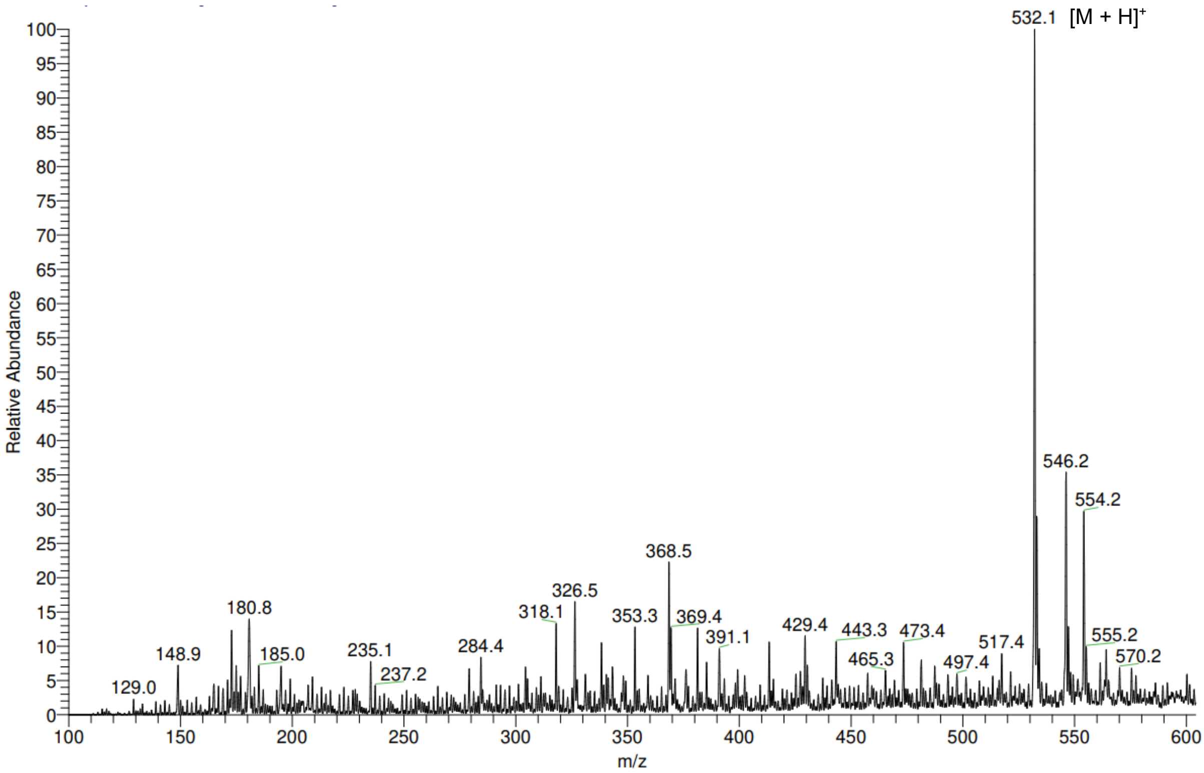

Figure S92. MS-ESI(+) spectrum of compound **5p**.

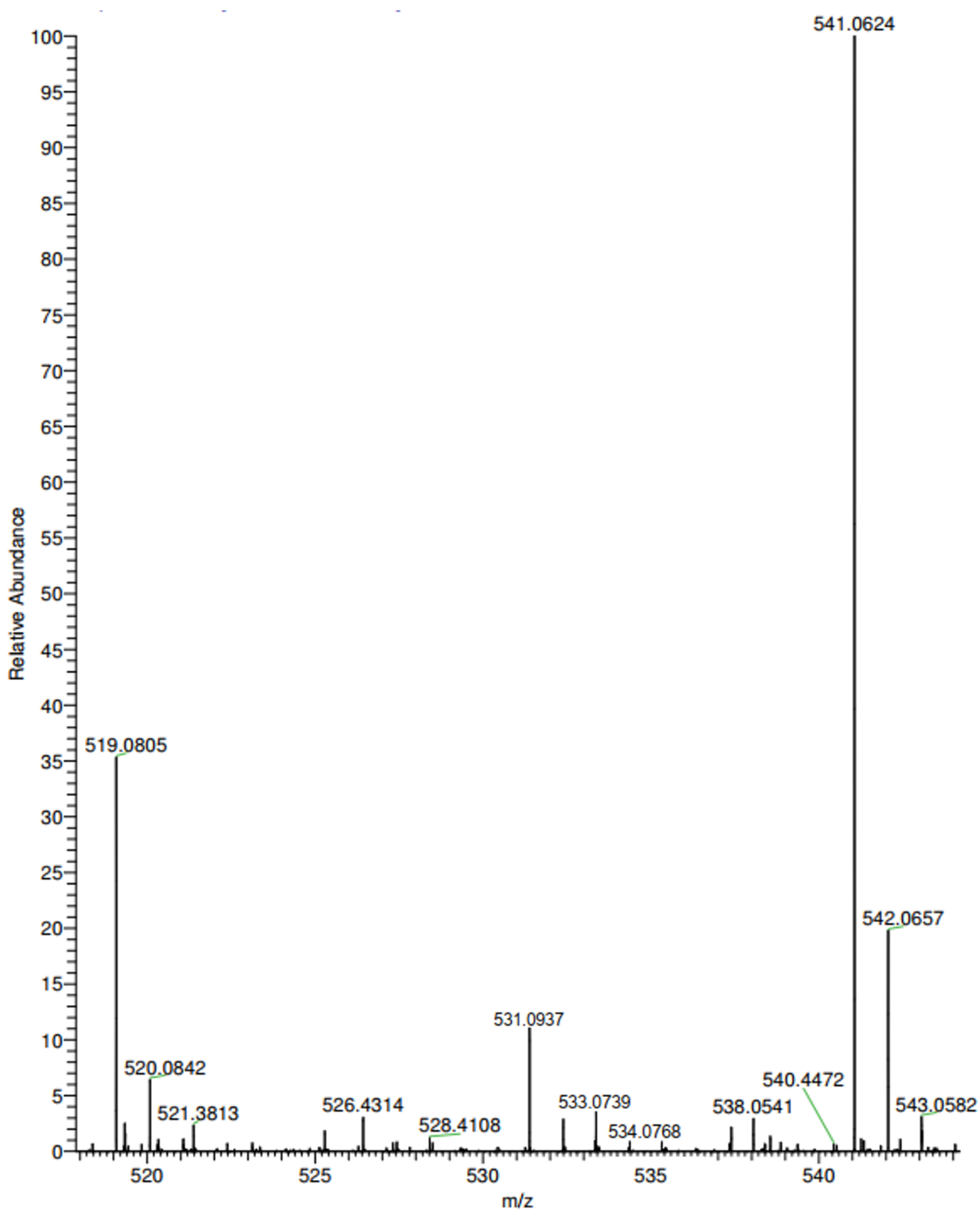

Figure S93. HRMS-ESI(+) spectrum of compound **5p**.

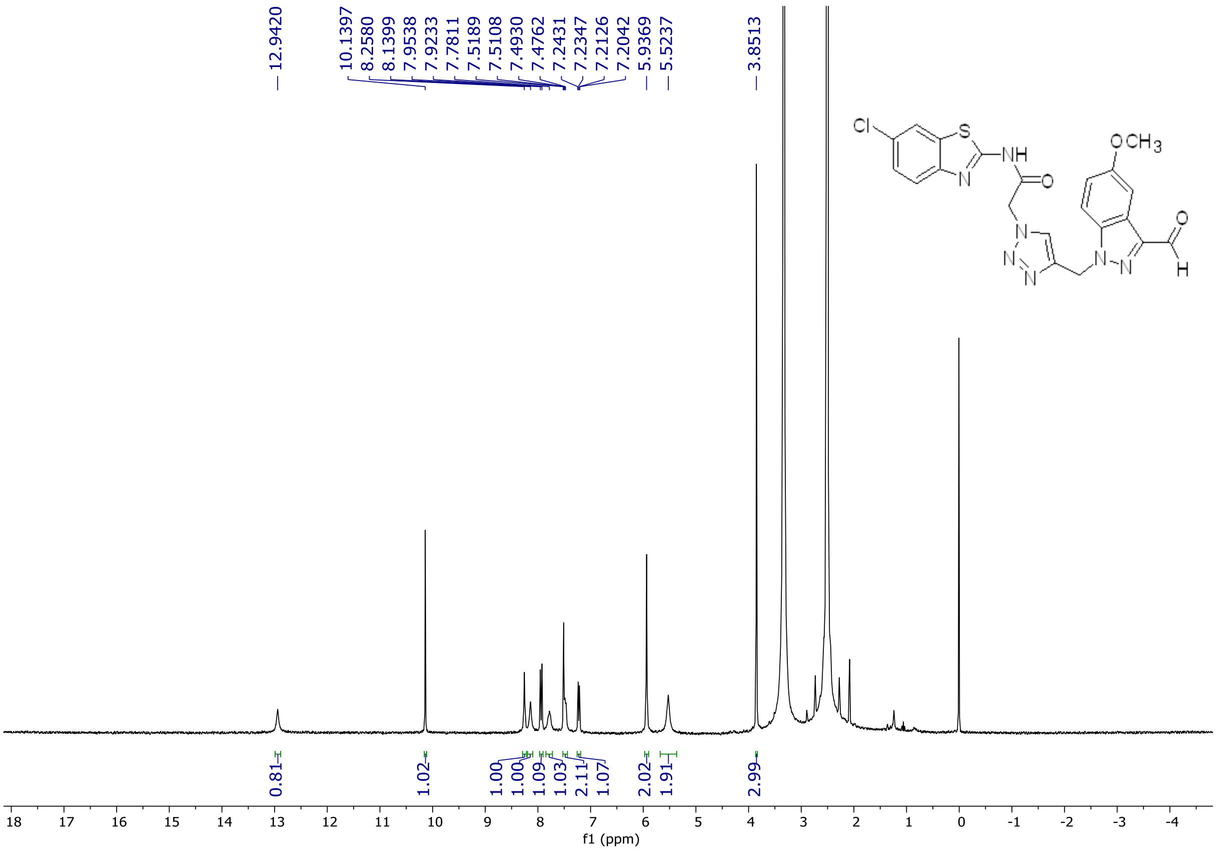

Figure S94. ^{11}H NMR spectrum of compound **5q** (300.13 MHz, DMSO-d_6).

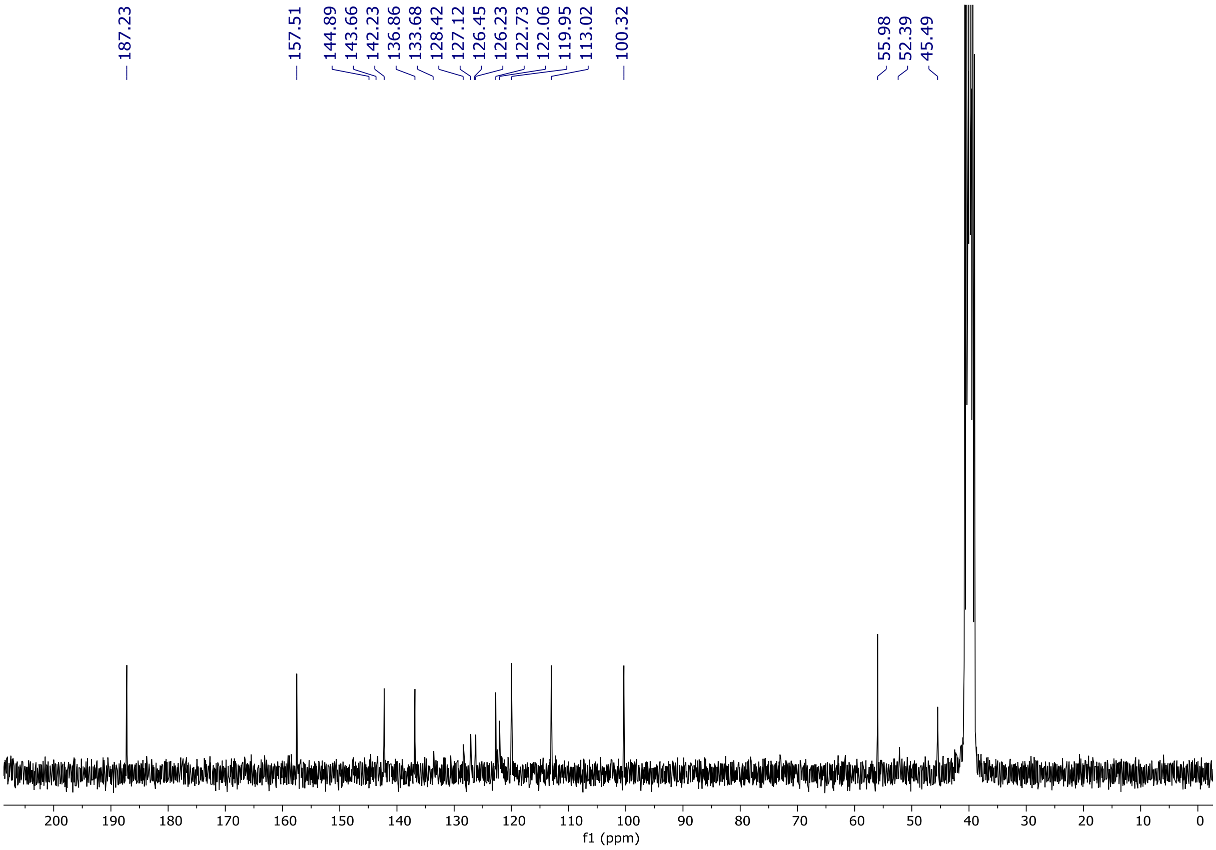

Figure S95. ^{13}C NMR spectrum of compound **5q** (75.47 MHz, DMSO-d_6).

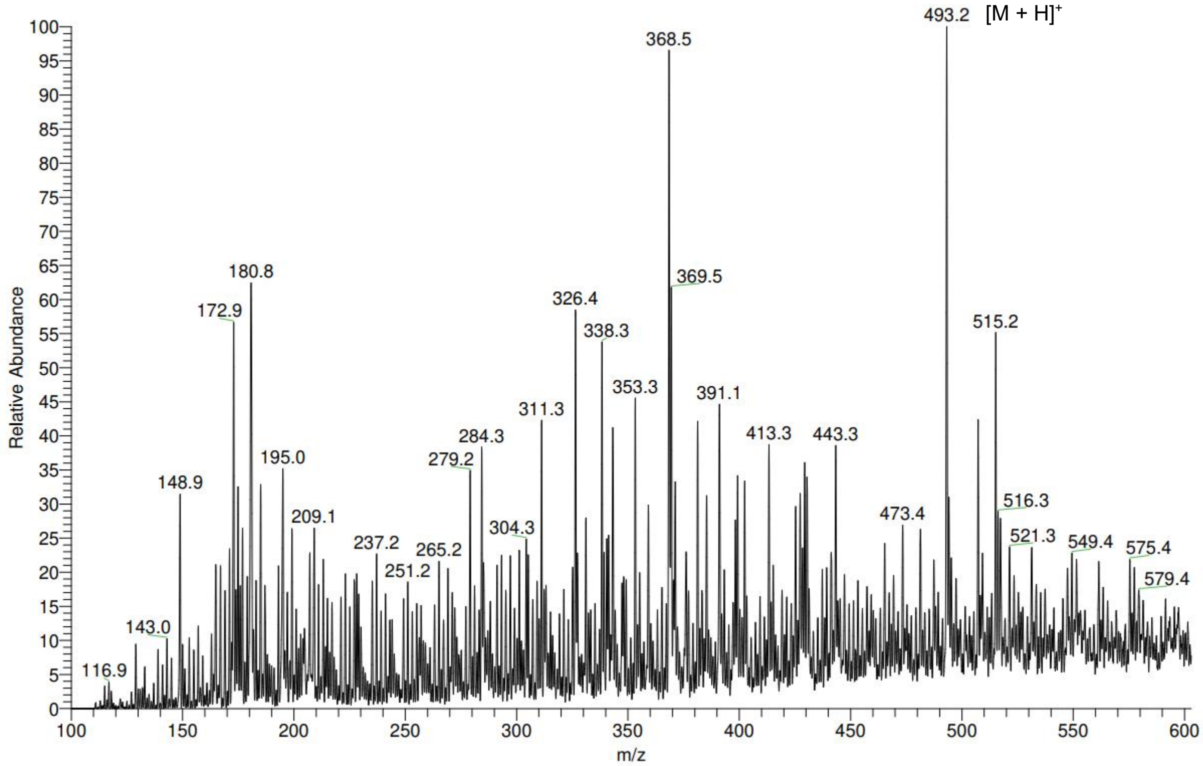

Figure S96. MS-ESI(+) spectrum of compound **5q**.

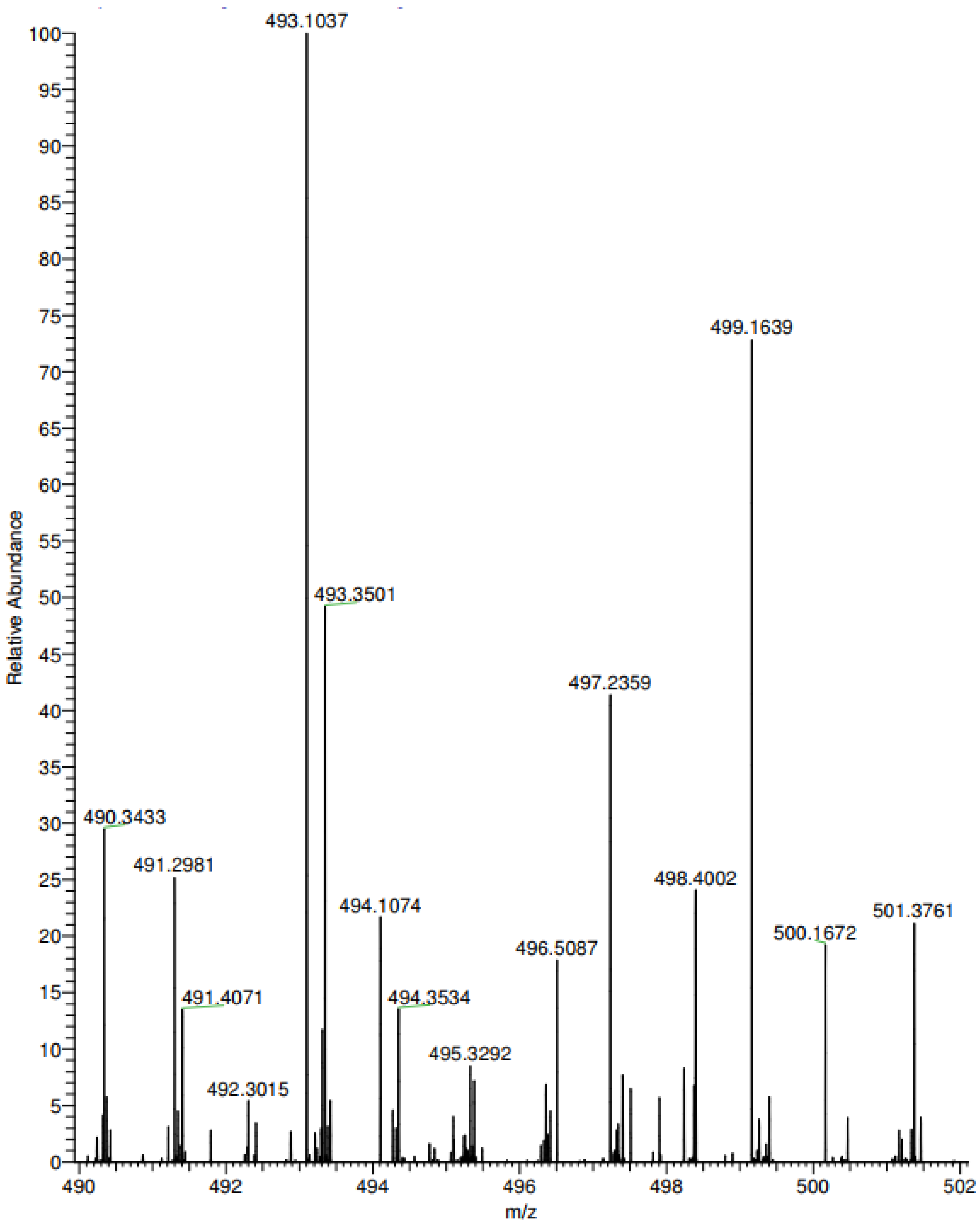

Figure S97. HRMS-ESI(+) spectrum of compound **5q**.

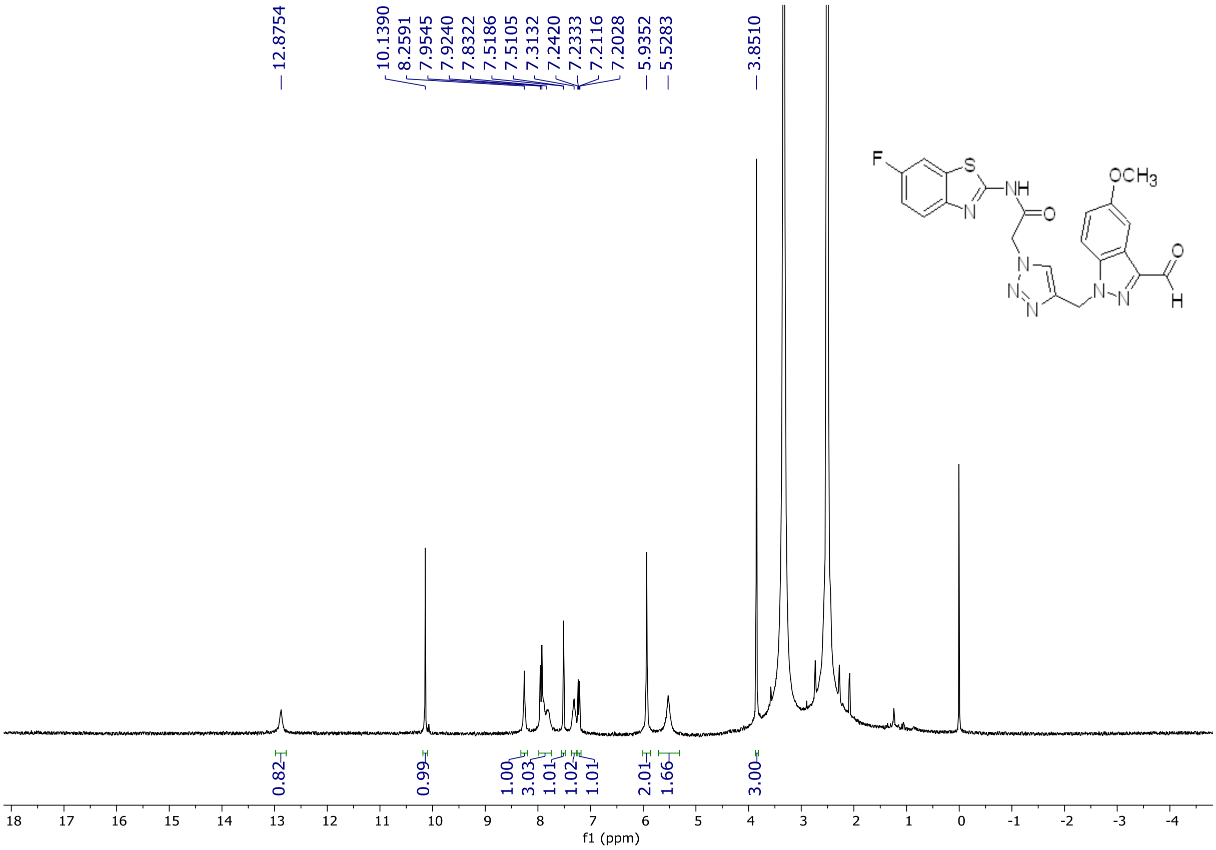

Figure S98. ^{1}H NMR spectrum of compound **5r** (300.13 MHz, DMSO-d_6).

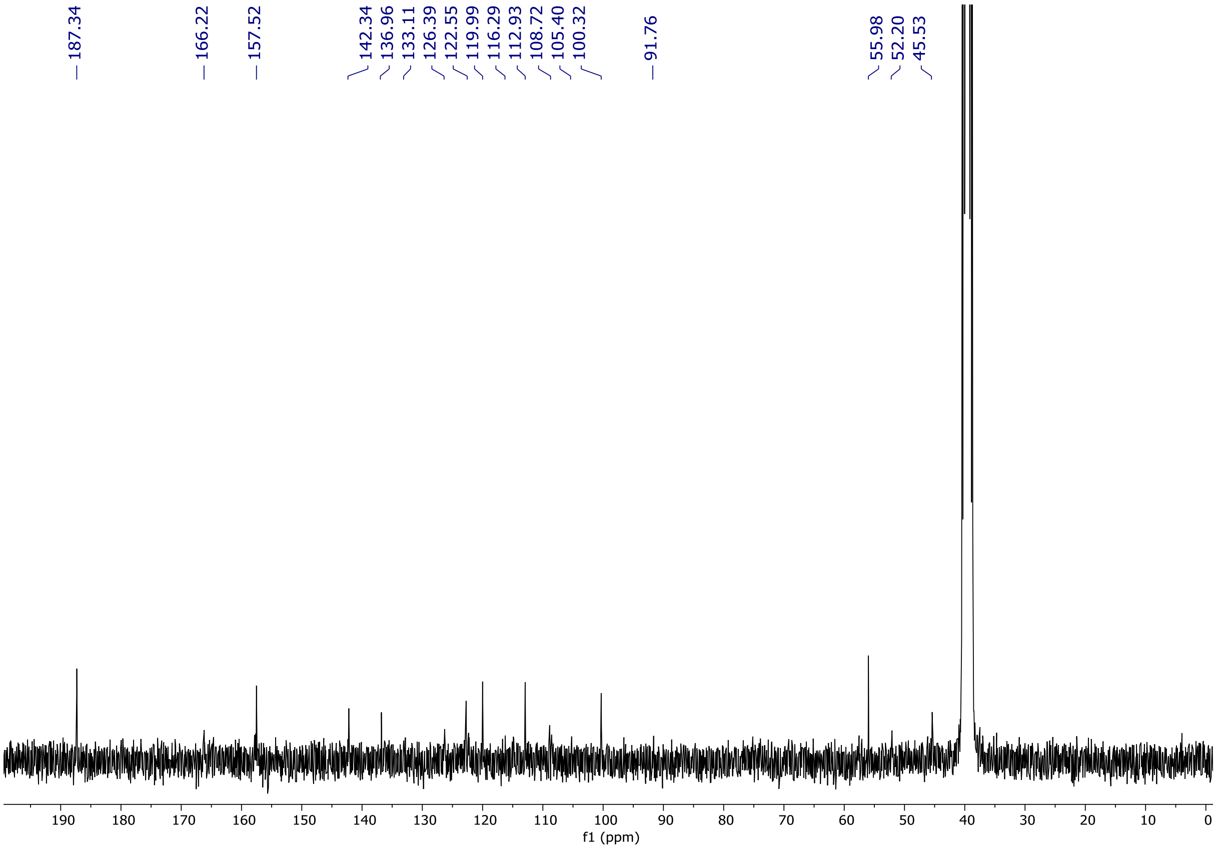

Figure S99. ^{13}C NMR spectrum of compound **5r** (75.47 MHz, DMSO-d_6).

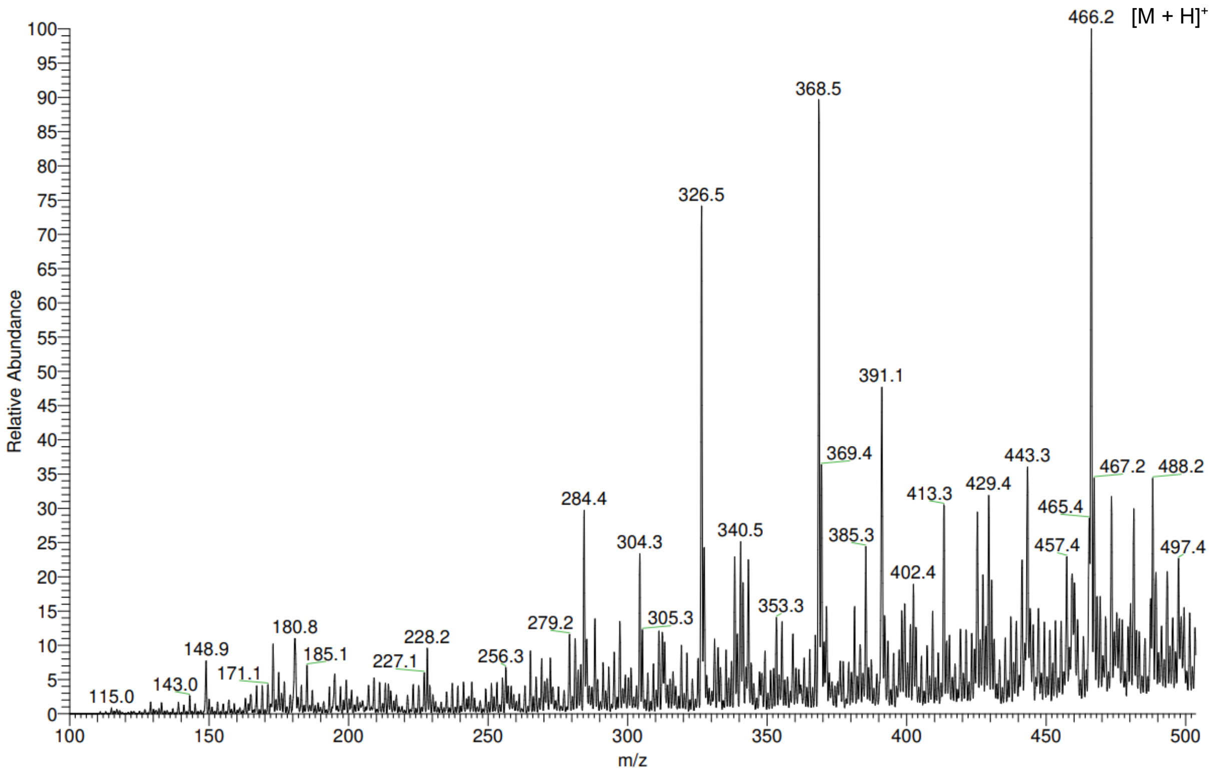

Figure S100. MS-ESI(+) spectrum of compound **5r**.

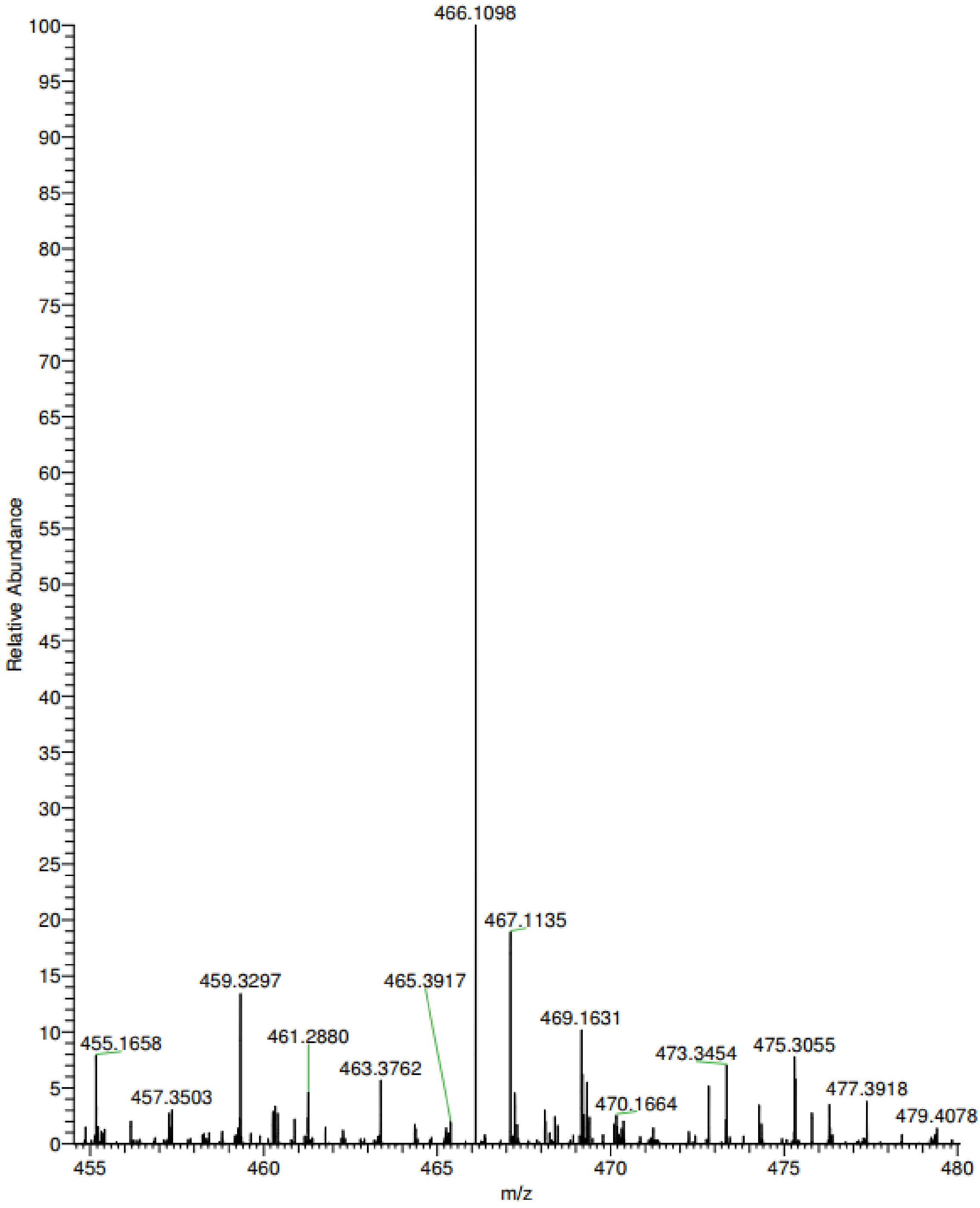

Figure S101. HRMS-ESI(+) spectrum of compound **5r**.

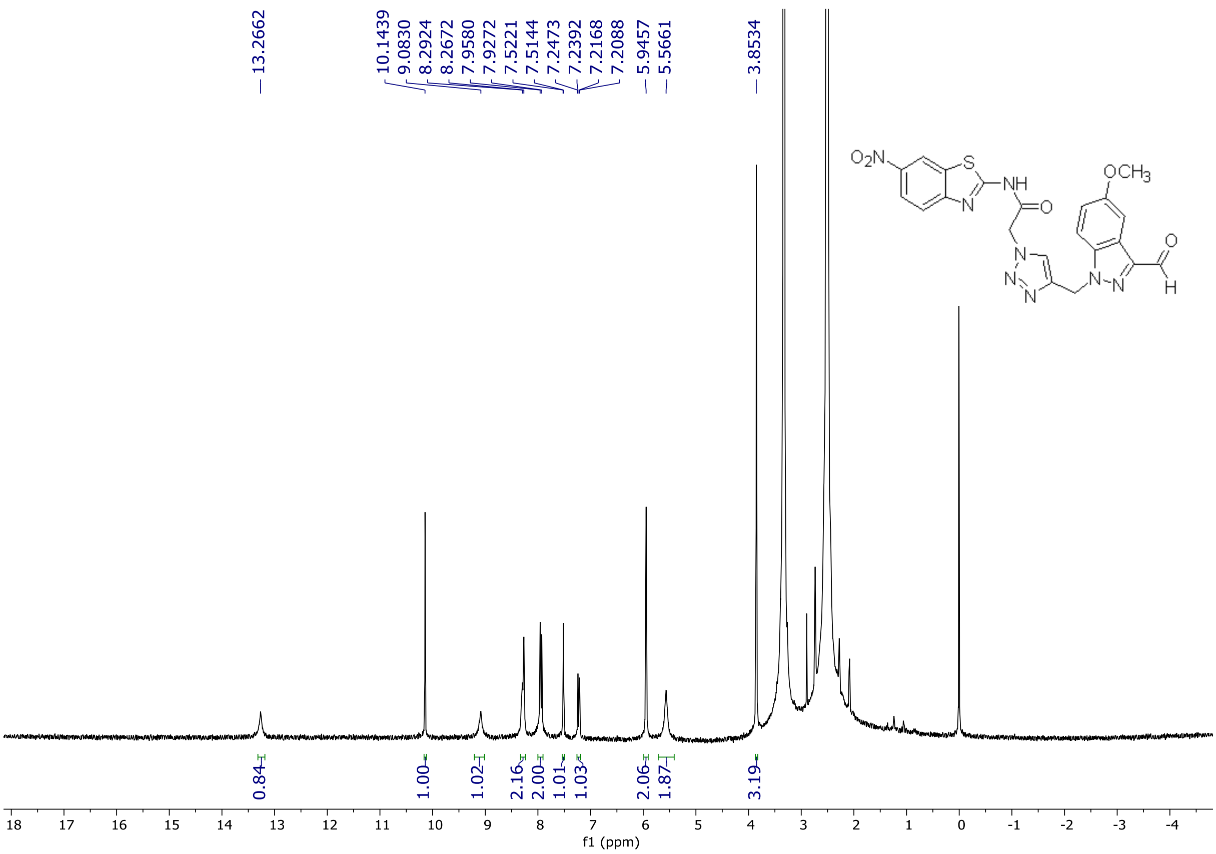

Figure S102. ^{1}H NMR spectrum of compound **5s** (300.13 MHz, DMSO-d_6).

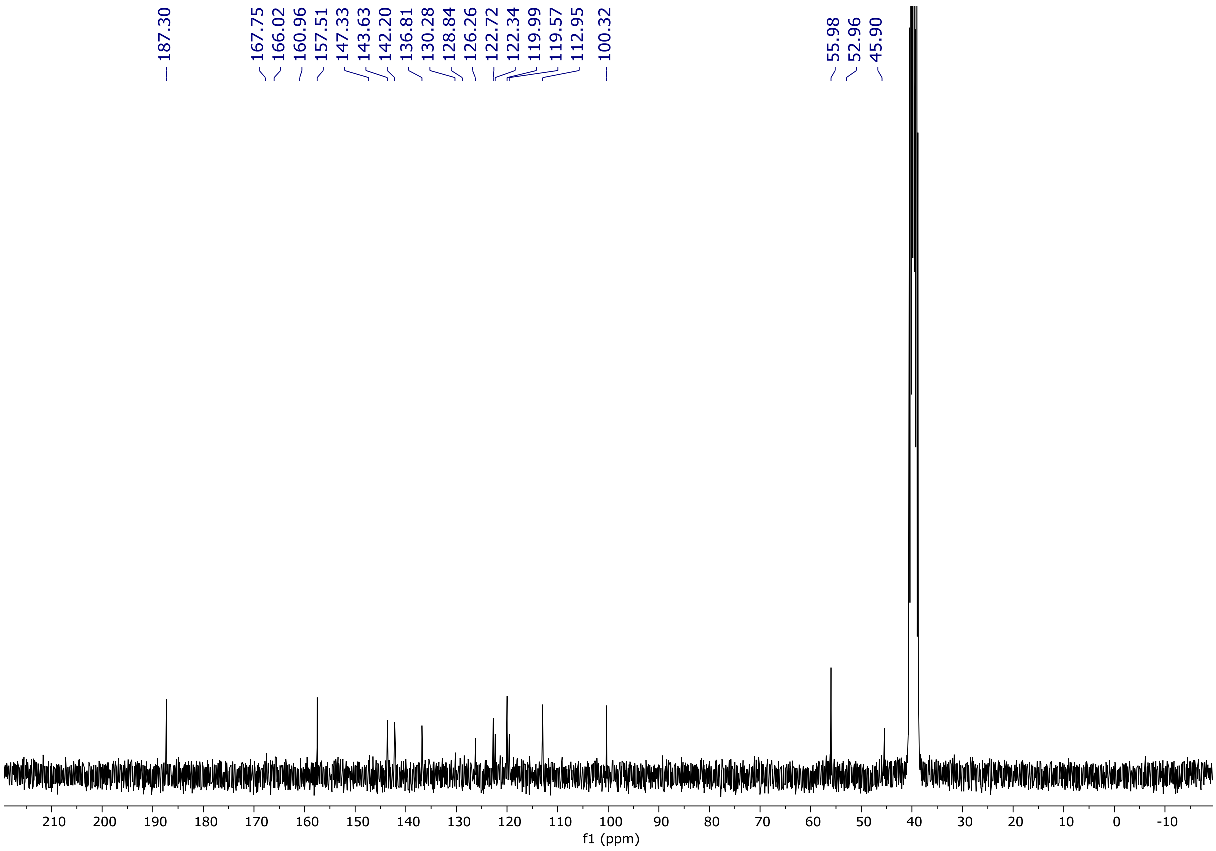

Figure S103. ^{13}C NMR spectrum of compound **5s** (75.47 MHz, DMSO-d_6).

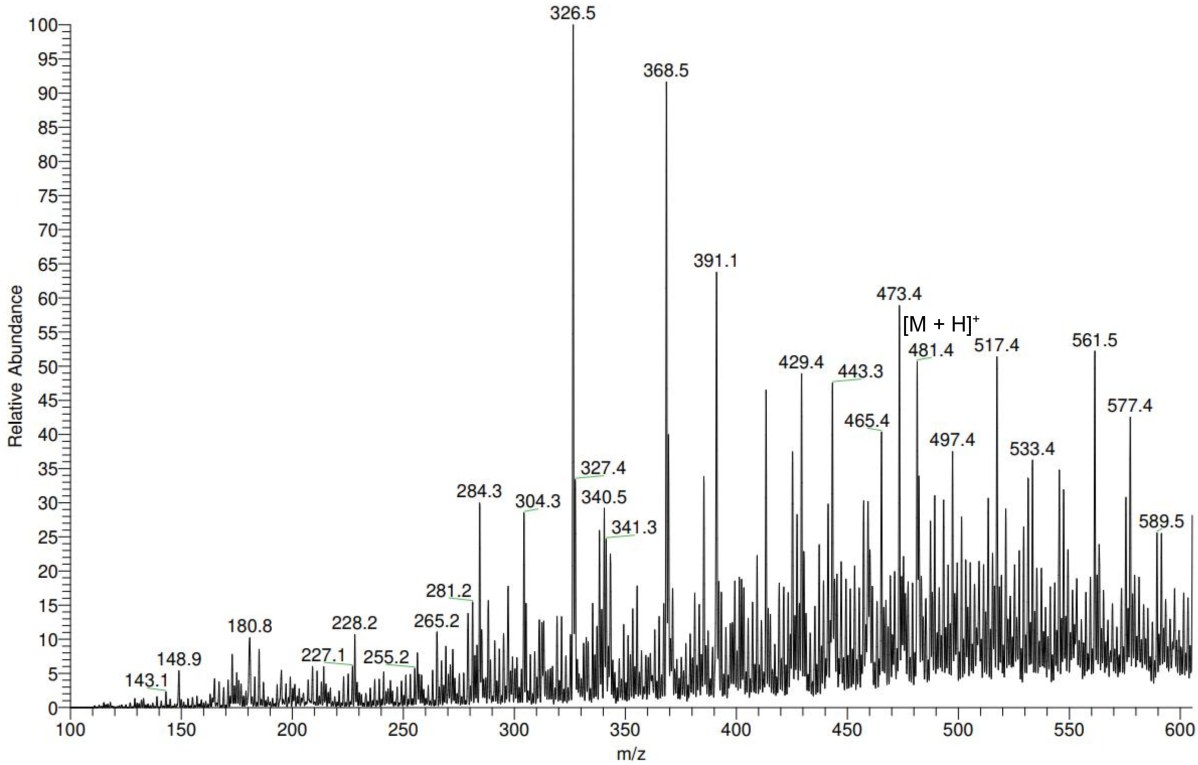

Figure S104. MS-ESI(+) spectrum of compound **5s**.

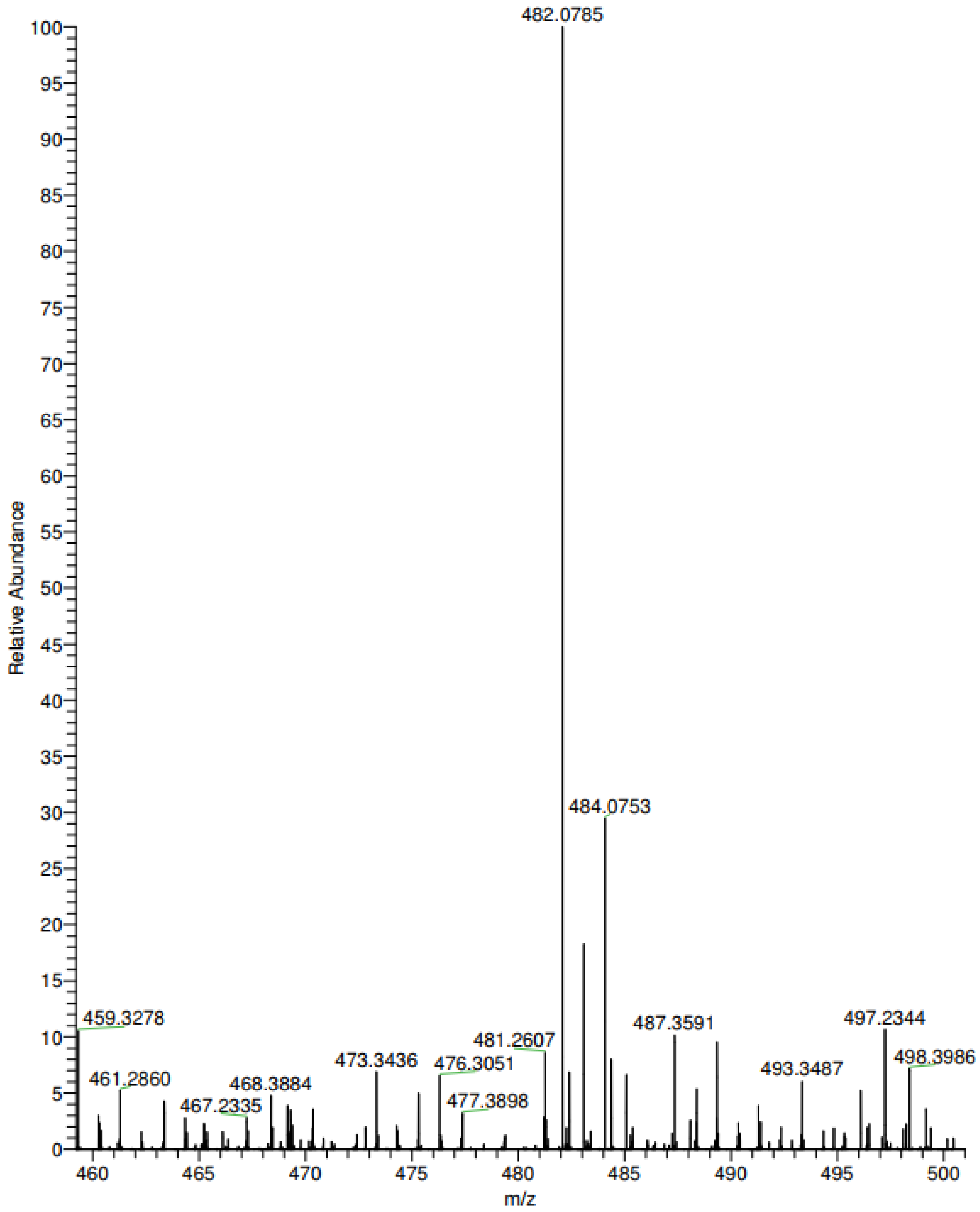

Figure S105. HRMS-ESI(+) spectrum of compound **5s**.

Table S1. Physicochemical properties, lipophilicity, water solubility, and pharmacokinetics, drug likeness and medicinal chemistry characteristics of compounds obtained in the online platform SwissADME.

Compd	Physicochemical Properties									Lipophilicity		Water Solubility	Pharmacokinetics								Druglikeness		Medicinal Chemistry		
	Formula	Molecular weight (g/mol)	Num. heavy atoms	Num. arom. heavy atoms	Fraction Csp3	Num. rotatable bonds	Num. H-bond acceptors	Num. H-bond donors	TPSA (Å²)	Log Po/w (XLOGP3)	Consensus Log Po/w		GI absorption	BBB permeant	P-gp substrate	CYP1A2 inhibitor	CYP2C19 inhibitor	CYP2C9 inhibitor	CYP2D6 inhibitor	CYP3A4 inhibitor	Lipinski	Bioavailability Score	PAINS	Leadlikeness	Synthetic accessibility
**Riluzole**	$C_8H_5F_3N_2OS$	234.20	15	9	0.12	2	5	1	76.38	3.61	2.81	Soluble	**High**	**Yes**	**No**	**Yes**	**Yes**	**No**	**No**	**No**	**Yes**; 0 violation	0.55	0 alert	**No**; 2 violations: MW<250, XLOGP3>3.5	2.24
**2b**	$C_{10}H_6ClF_3N_2O_2S$	310.68	19	9	0.20	5	6	1	79.46	3.69	3.00	Moderately soluble	**High**	**No**	**No**	**Yes**	**Yes**	**Yes**	**No**	**No**	**Yes**; 0 violation	0.55	0 alert	**No**; 1 violation: XLOGP3>3.5	2.54
**4b**	$C_{10}H_6F_3N_5O_2S$	317.25	21	9	0.20	6	9	1	129.21	4.17	2.60	Moderately soluble	**Low**	**No**	**No**	**Yes**	**No**	**Yes**	**No**	**No**	**Yes**; 0 violation	0.55	1 alert: azo_A	**No**; 1 violation: XLOGP3>3.5	2.69
**5a**	$C_{19}H_{14}N_8O_3S$	434.43	31	23	0.11	7	7	1	164.58	2.59	1.78	Moderately soluble	**Low**	**No**	**Yes**	**No**	**Yes**	**Yes**	**No**	**Yes**	**Yes**; 1 violation: NorO>10	0.55	0 alert	**No**; 1 violation: MW>350	3.49
**5b**	$C_{20}H_{16}N_8O_4S$	464.46	33	23	0.15	8	8	1	173.81	2.56	1.71	Moderately soluble	**Low**	**No**	**Yes**	**No**	**Yes**	**Yes**	**No**	**Yes**	**Yes**; 1 violation: NorO>10	0.55	0 alert	**No**; 2 violations: MW>350, Rotors>7	3.65
**5c**	$C_{20}H_{13}F_3N_8O_4S$	518.43	36	23	0.15	9	11	1	173.81	3.77	2.68	Moderately soluble	**Low**	**No**	**No**	**Yes**	**Yes**	**Yes**	**No**	**Yes**	**No**; 2 violations: MW>500, NorO>10	0.17	0 alert	**No**; 3 violations: MW>350, Rotors>7, XLOGP3>3.5	3.67
**5d**	$C_{19}H_{13}FN_8O_3S$	452.42	32	23	0.11	7	8	1	164.58	2.69	2.08	Moderately soluble	**Low**	**No**	**Yes**	**No**	**Yes**	**Yes**	**No**	**Yes**	**Yes**; 1 violation: NorO>10	0.55	0 alert	**No**; 1 violation: MW>350	3.54
**5e**	$C_{19}H_{13}ClN_8O_3S$	468.88	32	23	0.11	7	7	1	164.58	3.22	2.30	Moderately soluble	**Low**	**No**	**No**	**No**	**Yes**	**Yes**	**No**	**Yes**	**Yes**; 1 violation: NorO>10	0.55	0 alert	**No**; 1 violation: MW>350	3.54
**5f**	$C_{20}H_{16}N_8O_4S$	464.46	33	23	0.15	8	8	1	173.81	2.56	1.67	Moderately soluble	**Low**	**No**	**Yes**	**No**	**Yes**	**Yes**	**No**	**Yes**	**Yes**; 1 violation: NorO>10	0.55	0 alert	**No**; 2 violations: MW>350, Rotors>7	3.64
**5g**	$C_{20}H_{13}F_3N_8O4S$	518.43	36	23	0.15	9	11	1	173.81	3.77	2.69	Moderately soluble	**Low**	**No**	**No**	**Yes**	**Yes**	**Yes**	**No**	**Yes**	**No**; 2 violations: MW>500, NorO>10	0.17	0 alert	**No**; 3 violations: MW>350, Rotors>7, XLOGP3>3.5	3.66
**5h**	$C_{19}H_{13}N_9O_5S$	479.43	34	23	0.11	8	9	1	210.40	2.42	0.91	Moderately soluble	**Low**	**No**	**Yes**	**No**	**Yes**	**No**	**No**	**Yes**	**Yes**; 1 violation: NorO>10	0.55	0 alert	**No**; 2 violations: MW>350, Rotors>7	3.62
**5i**	$C_{19}H_{13}FN_8O_3S$	452.42	32	23	0.11	7	8	1	164.58	2.69	1.92	Moderately soluble	**Low**	**No**	**Yes**	**No**	**Yes**	**No**	**No**	**Yes**	**Yes**; 1 violation: NorO>10	0.55	0 alert	**No**; 1 violation: MW>350	3.52
**5j**	$C_{19}H_{13}ClN_8O_3S$	468.88	32	23	0.11	7	7	1	164.58	3.22	2.15	Moderately soluble	**Low**	**No**	**No**	**No**	**Yes**	**No**	**No**	**Yes**	**Yes**; 1 violation: NorO>10	0.55	0 alert	**No**; 1 violation: MW>350	3.52
**5k**	$C_{19}H_{13}BrN_8O_3S$	513.33	32	23	0.11	7	7	1	164.58	3.28	2.33	Moderately soluble	**Low**	**No**	**No**	**No**	**Yes**	**Yes**	**No**	**Yes**	**No**; 2 violations: MW>500, NorO>10	0.17	0 alert	**No**; 1 violation: MW>350	3.51
**5l**	$C_{19}H_{14}N_8O_3S$	434.43	31	23	0.11	7	7	1	164.58	2.59	1.79	Moderately soluble	**Low**	**No**	**Yes**	**No**	**Yes**	**Yes**	**No**	**Yes**	**Yes**; 1 violation: NorO>10	0.55	0 alert	**No**; 1 violation: MW>350	3.48
**5m**	$C_{20}H_{13}F_3N_8O_4S$	518.43	36	23	0.15	9	11	1	173.81	3.77	2.67	Moderately soluble	**Low**	**No**	**No**	**Yes**	**Yes**	**Yes**	**No**	**Yes**	**No**; 2 violations: MW>500, NorO>10	0.17	0 alert	2 alerts: nitro_group, oxygen-nitrogen_single_bond	3.66
**5n**	$C_{21}H_{17}N_7O_3S$	447.47	32	23	0.14	8	7	1	145.06	2.53	2.08	Moderately soluble	**Low**	**No**	**Yes**	**No**	**Yes**	**Yes**	**No**	**Yes**	**Yes**; 0 violation	0.55	0 alert	**No**; 2 violations: MW>350, Rotors>7	3.49
**5o**	$C_{22}H_{19}N_7O_4S$	477.50	34	23	0.18	9	8	1	154.29	2.50	2.16	Moderately soluble	**Low**	**No**	**Yes**	**No**	**Yes**	**Yes**	**No**	**Yes**	**Yes**; 1 violation: NorO>10	0.55	0 alert	**No**; 2 violations: MW>350, Rotors>7	3.67
**5p**	$C_{22}H_{16}F_3N_7O_4S$	531.47	37	23	0.18	10	11	1	154.29	3.71	3.02	Poorly soluble	**Low**	**No**	**No**	**No**	**Yes**	**Yes**	**No**	**Yes**	**No**; 2 violations: MW>500, NorO>10	0.17	0 alert	**No**; 3 violations: MW>350, Rotors>7, XLOGP3>3.5	3.69
**5q**	$C_{21}H_{16}N_8O_5S$	492.47	35	23	0.14	9	9	1	190.88	2.36	1.32	Moderately soluble	**Low**	**No**	**Yes**	**No**	**Yes**	**No**	**No**	**Yes**	**Yes**; 1 violation: NorO>10	0.55	0 alert	**No**; 2 violations: MW>350, Rotors>7	3.66
**5r**	$C_{21}H_{16}FN_7O_3S$	465.46	33	23	0.14	8	8	1	145.06	2.63	2.40	Moderately soluble	**Low**	**No**	**Yes**	**No**	**Yes**	**Yes**	**No**	**Yes**	**Yes**; 0 violation	0.55	0 alert	No; 2 violations: MW>350, Rotors>7	3.54
**5s**	$C_{21}H_{16}ClN_7O_3S$	481.91	33	23	0.14	8	7	1	145.06	3.16	2.62	Moderately soluble	**Low**	**No**	**No**	**No**	**Yes**	**Yes**	**No**	**Yes**	**Yes**; 0 violation	0.55	0 alert	No; 2 violations: MW>350, Rotors>7	3.54

Table S2. Percent proliferation of A2780, A549 and MDA-MB-231 cells treated with 30 μM of each compound **5a–s**. Values are expressed as percentage of untreated control (100%) ± standard deviation.

Compd	Cell line		
	A2780	A549	MDA-MB-231
**5a**	39.0 ± 4.3	72.1 ±3.2	87.2 ± 7.8
**5b**	58.3 ± 5.5	82.2 ±5.7	43.1 ± 2.2
**5c**	39.9 ± 7.5	99.8 ± 13.5	89.8 ± 8.5
**5d**	44.0 ± 5.1	74.5 ± 7.3	76.1 ± 7.3
**5e**	31.2 ± 9.0	68.2 ± 10.9	69.3 ± 8.0
**5f**	85.9 ± 5.8	99.4 ± 6.9	91.4 ± 9.8
**5g**	76.5 ± 5.2	93.5 ± 8.9	84.1 ± 8.0
**5h**	75.6 ± 10.0	97.4 ± 6.2	97.6 ± 4.0
**5i**	76.5 ± 6.3	93.9 ± 3.0	67.4 ± 4.6
**5j**	71.1 ± 6.6	89.5 ± 5.9	93.1 ± 8.5
**5k**	61.3 ± 4.6	79.0 ± 5.9	80.4 ± 2.6
**5l**	28.9 ± 8.2	73.5 ± 11.1	70.7 ± 9.9
**5m**	33.3 ± 9.33	90.7 ± 6.6	70.2 ± 7.2
**5n**	55.7 ± 5.4	72.5 ± 5.6	77.3 ± 2.3
**5o**	51.4 ± 9.10	79.4 ± 13.3	77.5 ± 11.7
**5p**	29.2 ± 10.0	81.1 ± 4.1	89.0 ± 9.6
**5q**	39.7 ± 6.8	94.5±8.1	88.9 ± 10.5
**5r**	31.1 ± 9.3	74.6±3.1	88.2 ± 5.1
**5s**	62.4 ± 10.5	87.5 ± 13.1	82.3 ± 14.1

Table S3. Dose-response effect of compounds **5a–e**, **5l** and **5p–5r**, on cell viability in A2780 ovarian carcinoma and MDA-MB-231 triple negative breast carcinoma cancer cells. Cells were treated with varying concentrations of the appropriate compound (0.096 to 60 μM) for 72 h. The values represent the percent cell proliferation referred to the untreated control (set to 100%). Data represent the mean ± standard deviation (SD) from independent experiments.

		**Applied concentration (uM)**							
**Compd**	**Cell line**	**0**	**60**	**30**	**12**	**2.4**	**0.48**	**0.096**	No exp.
**5a**	A2780	100	23.4 ± 6.4	24.4 ± 1.8	74.6 ± 11.1	80.5 ± 8.9	81.6 ± 7.6	90.8 ± 10.8	7
**5b**	MDA-MB-231	100	31.6 ± 3.1	29.4 ± 2.0	52.0 ± 0.2	84.4 ± 1.2	85.6 ± 0.6	97.6 ± 6.1	4
**5c**	A2780	100	26.4 ± 1.6		48.3 ± 0.9	68.6 ± 1.6	85.7 ± 7.9	91.2 ± 9.6	4
**5d**	A2780	100	11.5 ± 2.9	37.0 ± 4.9	56.7 ± 13.3	83.5 ± 6.0	88.9 ± 5.0	89.3 ± 6.5	6
**5e**	A2780	100	13.5 ± 7.6	15.5 ± 4.5	60.8 ± 17.3	88.2 ± 5.7	90.6 ± 7.8	91.3 ± 7.3	7
**5l**	A2780	100	28.2 ± 0.8		44.7 ± 2.5	65.4 ± 6.9	80.3 ± 13.9	85.4 ± 16.7	4
**5p**	A2780	100	13.3 ± 2.3		50.6 ± 1.7	63.9 ± 1.7	74.7 ± 1.7	88.3 ± 0.6	4
**5q**[a]	A2780	100	35.6 ± 1.0		52.0 ± 1.3	71.7 ± 2.2	89.7 ± 1.8	98.5 ± 3.4	4
**5r**	A2780	100	12.6 ± 1.7		29.7 ± 2.0	48.5 ± 3.4	73.1 ± 5.2	92.7 ± 5.5	4

[a] – IC_{50} = 33.9 ± 1.7 - A little above the limit to define the molecule as active.
